# Combining silver- and organocatalysis: an enantioselective sequential catalytic approach towards pyrano-annulated pyrazoles†
†Electronic supplementary information (ESI) available. CCDC 1035443. For ESI and crystallographic data in CIF or other electronic format see DOI: 10.1039/c4cc09495f



**DOI:** 10.1039/c4cc09495f

**Published:** 2015-02-11

**Authors:** Daniel Hack, Pankaj Chauhan, Kristina Deckers, Yusuke Mizutani, Gerhard Raabe, Dieter Enders

**Affiliations:** a Institute of Organic Chemistry , RWTH Aachen University , Landoltweg 1 , 52074 Aachen , Germany . Email: enders@rwth-aachen.de; b Nara Institute of Science and Technology (NAIST) , Ikoma , Japan

## Abstract

A one-pot asymmetric Michael addition/hydroalkoxylation sequence, catalyzed by a sequential catalytic system consisting of a squaramide and a silver salt, provides a new series of chiral pyrano-annulated pyrazole derivatives in excellent yields (up to 95%) and high enantioselectivities (up to 97% ee).

## Abstract 2

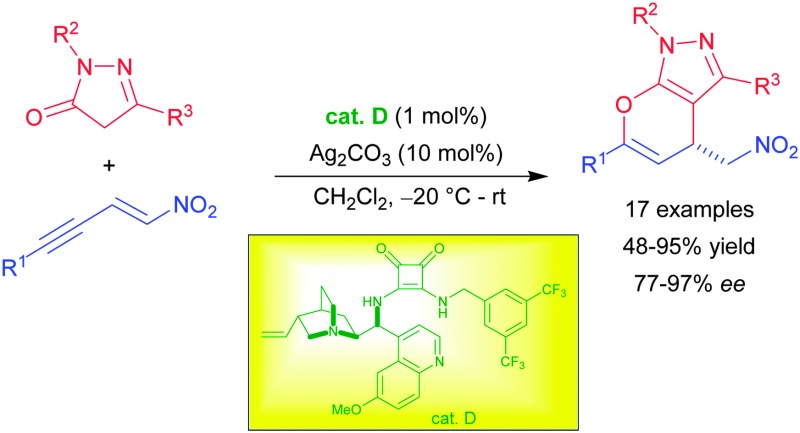
A one-pot sequential bicatalytic asymmetric Michael addition/hydroalkoxylation provides a new series of pyrano-annulated enantioenriched pyrazole derivatives.

Pyrazoles and their derivatives represent a versatile class of organic molecules found widely in pharmaceuticals and agrochemicals.^1^ Their remarkable bioactivity makes these N-heterocycles desirable targets in various fields of research, *e.g.* contributing to the success of blockbuster drugs like Viagra and Celebrex. In addition, pyrano-annulated pyrazoles also exhibit a broad spectrum of such bioactive properties, including antifungal (**I**), antibacterial (**II**) and anti-inflammatory behaviour (**III**) (Fig. 1).^2^

**Fig. 1:**
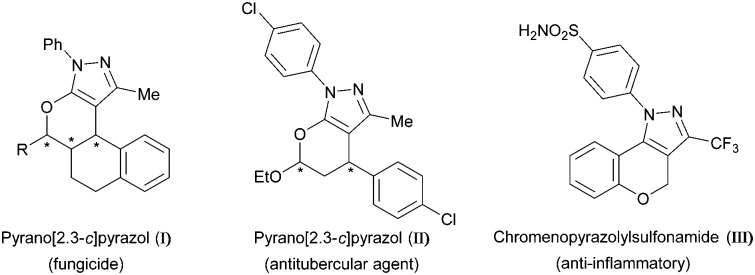
Examples of bioactive pyrano-annulated pyrazoles.

With the advent of organocatalysis, the exploitation of organocatalytic asymmetric Michael additions using pyrazolinones as nucleophiles has been an ongoing field of research.^3^ As pyrazolinones provide an activated methylene group, which allows for consecutive functionalization, more demanding domino reactions have been developed, generating quaternary carbon stereocenters and spirocyclic pyrazolinones.^4^ However, less attention has been given to the development of domino reactions which rely on the ambident nucleophilicity of pyrazolinones, and examples in which the enol oxygen acts as a nucleophile remain scarce. In 2012 our group synthesized annulated pyrazoles *via* a one-pot cascade sequence catalyzed by a secondary amine,^2*b*^ and recently Ye and co-workers have generated similar structures *via* NHC organocatalysis (Scheme 1).^5^

**Scheme 1:**
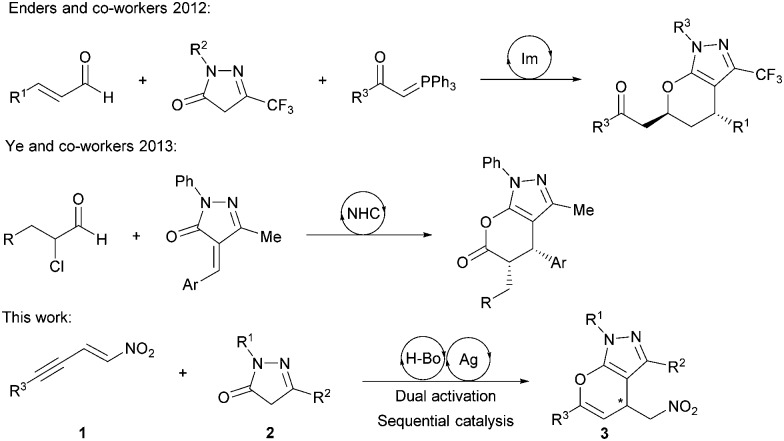
Former and current approaches for the asymmetric synthesis of chiral pyrano-annulated pyrazoles.

Lately, our group became interested in the merger of transition metal catalysis and organocatalysis.^6^ Both catalytic fields offer various advantages providing complemental activation modes for different functional groups. The combination is especially appealing, if the reaction can be performed as a one-pot procedure, because the purification of the intermediates can be omitted for waste minimization. On the other hand, common problems like mutual catalyst deactivation and solvent compatibility have to be addressed in order to carry for efficient sequential or relay catalysis.

While in the field of alkyne activation initial credit was given to expensive metals, we have recently demonstrated that low-priced silver salts can act as efficient catalysts in the presence of primary amines for the synthesis of chiral furano- and pyrano-annulated hydroxycoumarins.^7^ In this case, silver circumvents the initial drawbacks of the gold catalyst providing higher selectivity with better catalyst compatibility at lower catalyst costs.^8,9^

Considering the impact of bioactive pyrazoles and the recent developments in sequential catalysis, we envisioned the synthesis of enantiopure pyrano-annulated pyrazoles using an orthogonal activation strategy. Herein we present the synthesis of the desired compounds using alkyne-tethered nitroolefins **1** and pyrazolinones **2** in the presence of cinchona-derived squaramides and silver carbonate. The reaction proceeds *via* an asymmetric Michael addition, followed by a subsequent selective 6-*endo* dig hydroalkoxylation under mild conditions and low catalyst loading (1 mol% of organocatalyst) without a notable catalyst deactivation. This allows for an easy one-pot protocol under sequential catalytic conditions giving the pyrano-annulated pyrazoles in good yields and enantioselectivities.

Initially, we focused on the asymmetric Michael addition. Thus, the alkyne-tethered nitroolefin **1a** was treated with pyrazolinone **2a** in dichloromethane in the presence of different squaramide and thiourea catalysts at room temperature (Scheme 2).^10^ Gratifyingly, all catalysts provided the Michael adduct **3a** in good to excellent yields (up to 92%), with the exception of the thiourea catalyst **B** for which an extended reaction time was required. This indicates that the reaction proceeds faster under basic conditions, as catalyst **B** does not provide a basic tertiary amine that can catalyze the tautomerization of the pyrazolinones. Compared to thioureas or squaramide **C**, the cinchona-derived squaramides gave better results in terms of enantioselectivity with up to 77% ee (**D–H**). Since there was no significant difference in terms of selectivity among the different quinine- and cinchonidine-derived squaramides, we decided to use catalyst **D** for further optimization.

**Scheme 2:**
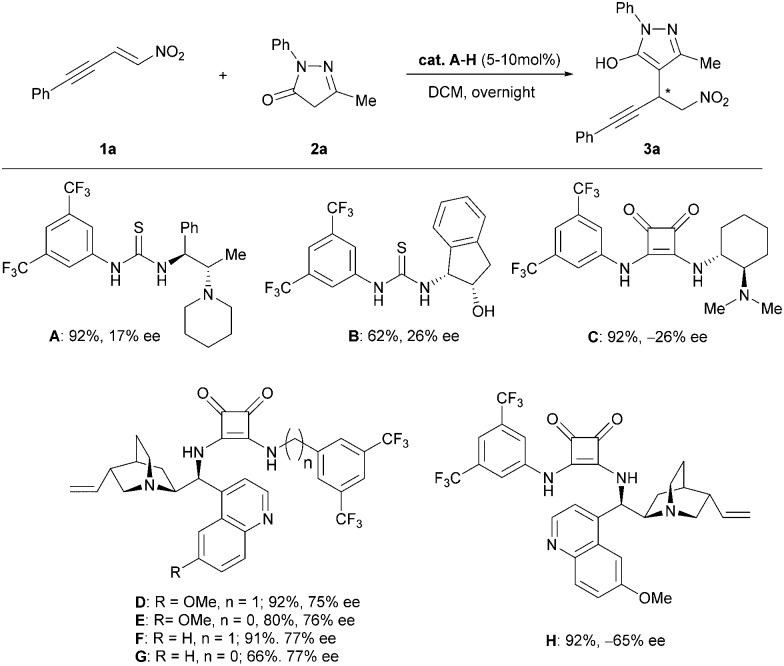
Catalyst screening for the asymmetric Michael addition of pyrazolinone **2a** to the alkyne-tethered nitroolefin **1a**.

Further optimization studies revealed that the choice of the solvent did not have a pivotal effect on the outcome of the reaction (Table 1). While satisfactory yields were obtained with all solvents tested, it turned out that the reaction proceeded with higher enantiomeric excess in dichloromethane (entries 1–4). However, we envisioned that higher enantioselectivity could be achieved at lower temperature, given that the reaction progress was fast at room temperature. Indeed, by decreasing the temperature to 0 °C, it became possible to obtain **3a** with a slight increase in enantiomeric excess without any loss of yield (entry 5). Further lowering of the temperature still had a beneficial effect on the outcome of the reaction, however temperatures below –20 °C only led to a deteriorated reaction rate (entries 7–9). Interestingly, further studies on the effect of the catalyst loading have shown that it is possible to reduce the catalyst loading to 1 mol% yielding the Michael adduct **3a** in 92% and 92% ee within six hours (entries 10–12).

**Table 1:** Optimization of the asymmetric Michael addition^*a*^

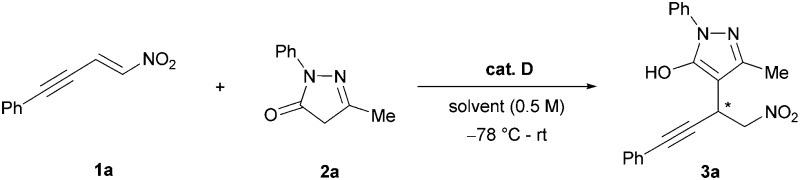						
Entry	Solvent	Cat. load (mol%)	*T* (°C)	*t* (h)	Yield^*b*^ (%)	ee^*c*^ (%)
1	CH_2_Cl_2_	5	rt	12	92	75
2	Toluene	5	rt	12	90	73
3	CHCl_3_	5	rt	12	86	61
4	Et_2_O	5	rt	12	88	63
5	CH_2_Cl_2_	5	0	0.5	93	89
6	Toluene	5	0	6	79	83
7	CH_2_Cl_2_	5	–20	2	93	92
8	CH_2_Cl_2_	5	–40	6	92	92
9	CH_2_Cl_2_	5	–78	6	92	92
10	CH_2_Cl_2_	7	–20	2	93	91
11	CH_2_Cl_2_	3	–20	2	93	92
12	CH_2_Cl_2_	1	–20	6	92	92

^*a*^Reaction conditions: 0.28 mmol (1.1 equiv.) of **1a**, 0.25 mmol (1.0 equiv.) of **2a**, 1–7 mol% of catalyst **D**, 0.5 mL solvent.

^*b*^Yield of isolated **3a** after flash chromatography.

^*c*^The enantiomeric excess was determined *via* the *O*-acetylated derivative **3a′** by HPLC analysis on a chiral stationary phase.

Having optimized the organocatalytic step, we turned our attention to the subsequent hydroalkoxylation. We anticipated that the π-activation of the alkyne by various late transition metals would enable the hydroalkoxylation of the enol tautomer of the pyrazolinone to either give a five- or six-membered ring-annulated pyrazole. To our surprise, the Michael adduct **3a** cleanly underwent cyclization to form the 6-*endo* dig-derived product **4a** in the presence of 10 mol% of an unactivated gold(i) catalyst in quantitative yields within 15 minutes in toluene (Table 2, entry 1). Similar results were obtained with various silver salts in dichloromethane, allowing for an easy one-pot procedure later on (entries 2–6). Recently, we have reported on a similar silver-catalyzed hydroalkoxylation which led to 5-*exo*-derived annulated hydroxycoumarins with minor amounts of 6-*endo*-derived products.^7^ Because of these results, we were intrigued by the inversed regioselectivity and the lack of side products. Presuming that the addition mainly underlies electronic effects, it is possible that the close proximity of the strongly electron withdrawing nitro group diminishes the original + I-effect of the methylene group next to the alkyne. Thus, the 6-*endo* addition seems more favourable because the aromatic substituent is less electron withdrawing and can further provide extra stabilization by mesomeric effects if free rotation of the aromatic ring is possible. Using silver salts is more attractive, because in general the more expensive gold catalysts have to be activated with equal amounts of silver salts and based on our previous experiences silver catalysts appear to be less prone to a deactivation by Lewis basic organocatalysts.^7^ Other π-acidic transition metals, including copper and platinum, and Brønsted acids like TFA failed to promote the reaction (entries 7–9). As anticipated, it was possible to combine both catalytic reactions for the desired one-pot protocol. With both catalysts being present from the beginning, we obtained the cyclized product **4a** with 88% yield and 94% ee (Table 3, entry 1).

**Table 2:** Optimization of the transition metal-catalyzed hydroalkoxylation of the Michael adduct **3a** ^*a*^

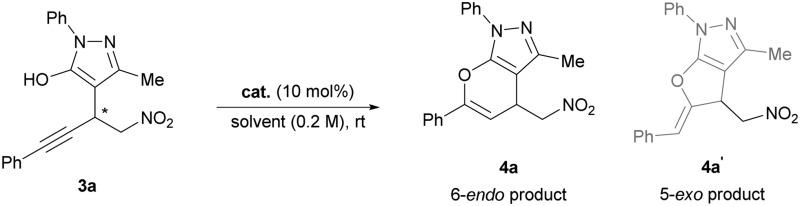				
Entry	Catalyst	Solvent	*t* (h)	Yield^*b*^ (%)
1	PPh_3_AuCl/AgNTf_2_	Toluene	0.25	99
2	AgNO_3_	CH_2_Cl_2_	3.5	97
3	AgNTf_2_	CH_2_Cl_2_	5	99
4	AgBF_4_	CH_2_Cl_2_	3	97
5	Ag_2_O	CH_2_Cl_2_	>96	69
6	Ag_2_CO_3_	CH_2_Cl_2_	2	99
7	TFA	CH_2_Cl_2_	>24	Traces
8	CuI	CH_2_Cl_2_	>24	Traces
9	PtCl_2_	CH_2_Cl_2_	>24	Traces

^*a*^Reaction conditions: 0.10 mmol of **3a**, 10 mol% of catalyst, solvent 0.5 mL, rt.

^*b*^Yield of isolated **4a** after flash chromatography.

**Table 3:** Scope of the investigated reaction^*a*^

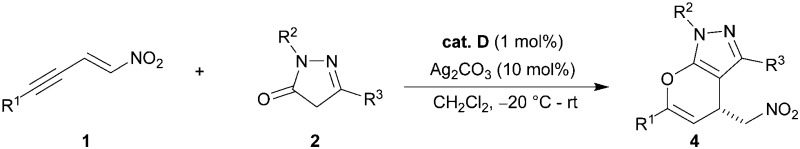					
**4**	R^1^	R^2^	R^3^	Yield^*b*^ (%)	ee^*c*^ ^,^ ^*d*^ (%)
**a**	Ph	Ph	Me	88	94 (99)
**b**	2-Br-C_6_H_4_	Ph	Me	77	95
**c**	4-CF_3_-C_6_H_4_	Ph	Me	89	85 (99)
**d**	2-Cl-C_6_H_4_	Ph	Me	74	88 (99)
**e**	3-Me-C_6_H_4_	Ph	Me	88	86 (99)
**f**	3-MeO-C_6_H_4_	Ph	Me	93	84 (99)^*d*^
**g**	3,4-OCH_2_O-C_6_H_3_	Ph	Me	93	89
**h**	1-Naphthyl	Ph	Me	77	93
**i**	2-Naphthyl	Ph	Me	91	91
**j**	1-Furanyl	Ph	Me	93	82 (99)
**k**	1-Thienyl	Ph	Me	92	89 (98)
**l**	Ph	2-Cl-C_6_H_4_	Me	87	91
**m**	Ph	4-Cl-C_6_H_4_	Me	93	89 (99)
**n**	Ph	Me	CF_3_	90	77 (99)
**o** ^*e*^	Ph	Me	Me	48	85 (99)
**p**	*n*-Butyl	Ph	Me	95	77 (99)
**q**	Cyclopentyl	Ph	Me	91	85 (99)

^*a*^Reaction conditions: 0.55 mmol (1.1 equiv.) of **1a**, 0.5 mmol (1.0 equiv.) of **2a**, 1 mol% **D**, 10 mol% Ag_2_CO_3_, 0.5 mL CH_2_Cl_2_, 12 h at –20 °C, 2 h at rt.

^*b*^Yield of isolated **4** after flash chromatography.

^*c*^The enantiomeric excess was determined by HPLC analysis on a chiral stationary phase.

^*d*^Values in brackets after one recrystallization from ethyl acetate–*n*-pentane.

^*e*^The reaction was performed at room temperature.

With the optimized conditions in hand, we focused on the general applicability of the developed one-pot protocol by screening substrates with different substituents. A broad scope of different internal alkynes was tested, and the annulated pyrazoles (**4a–k**, **4p**, **q**) were obtained in good to excellent yields and enantioselectivities, irrespective of the steric or electronic nature of the substituents tolerating various aromatic, hetero-aromatic and aliphatic groups. Only the substrates with bulky substituents on the 2-position gave slightly lower yields (**4b**, **4d**, **4h**). Interestingly, in all examples a clean cyclization to the 6-*endo*-derived products was observed. In addition, the use of different pyrazolinones led to similar results and only the reaction for 2,5-dimethyl pyrazolinone had to be performed at room temperature because of its limited solubility at lower temperatures (**4o**).

The absolute configuration was unambiguously determined by X-ray crystal structure analysis of compound **4a** and by analogy of all other products was assigned accordingly (Fig. 2).

**Fig. 2:**
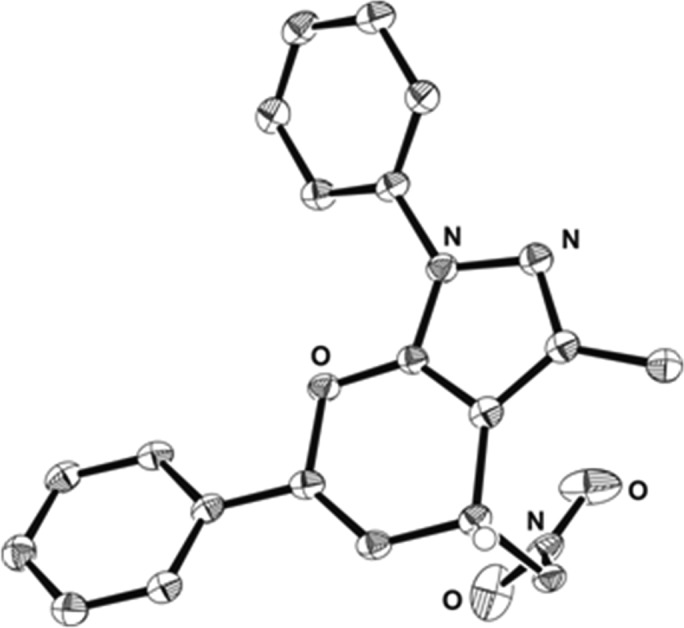
X-ray crystal structure of (*R*)-**4a**.^11^

To demonstrate the applicability of our newly developed protocol, we conducted a larger scale synthesis of compound **4i**. To our satisfaction, **4i** was obtained in 95% and 97% ee giving even better results than on small scale.

The proposed mechanism consists of two distinct catalytic cycles (Scheme 3). The squaramide acts as bifunctional catalyst responsible for the preorientation and the activation of the substrates. While the nitro alkene moiety gets activated upon the formation of hydrogen bonds between the nitro group and the squaramide, the pyrazolinone undergoes nucleophilic activation by hydrogen bonding with the quinuclidine moiety of the catalyst. Simultaneously, the nucleophile will be directed to the Si-face of the nitroolefin, thus accounting for the observed enantioselectivity. After the asymmetric Michael addition, the silver-catalyzed electrophilic activation of the internal alkyne triggers the subsequent hydroalkoxylation. The 6-*endo*-dig cyclization proceeds *via* stereoselective anti-addition of the enol to the alkyne, accounting for the generally observed *Z*-products. The generated vinylsilver intermediates are not stable under the applied conditions, and undergo fast protodeargentation yielding the pyrano-annulated pyrazoles and releasing the catalyst for the next cycle.

**Scheme 3:**
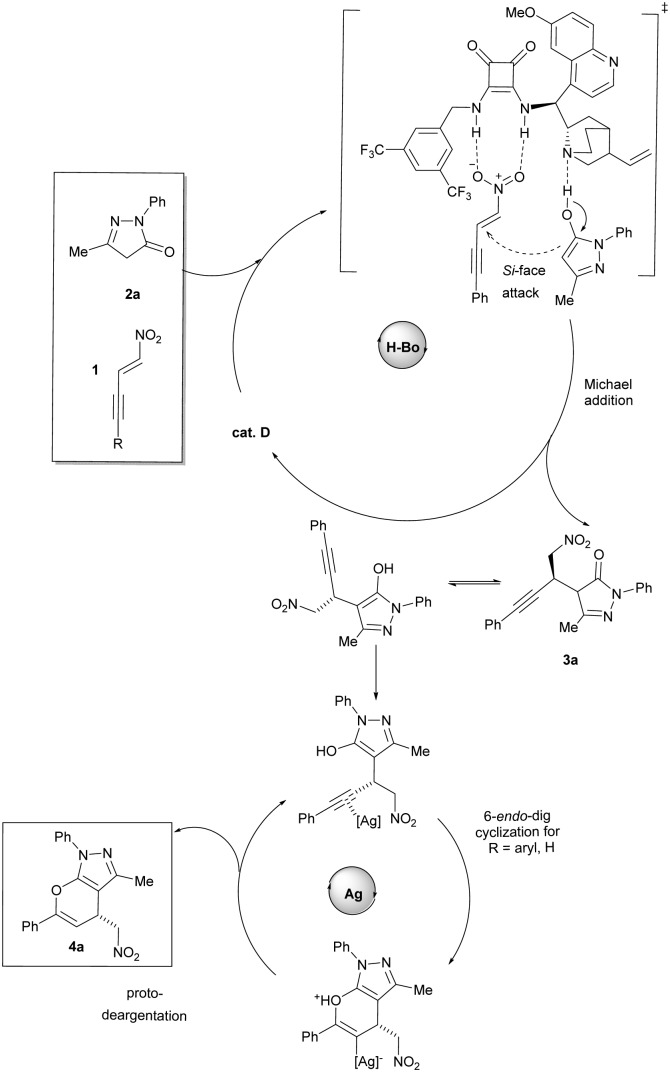
Proposed mechanism for the sequential catalytic domino reaction.

In summary, we have developed a novel synthesis of chiral pyrano-annulated pyrazoles by the combination of squaramide and silver catalysis. The sequential catalytic system consisting of an asymmetric Michael addition and a subsequent hydroalkoxylation gave the potentially bioactive pyrano-annulated pyrazoles under mild reaction conditions and with low catalyst loading in excellent yields and enantioselectivities. Further investigations towards the synthesis of heterocycles following similar orthogonal activation strategies are in progress in our laboratories.

Support from the European Research Council (ERC Advanced Grant 320493 “DOMINOCAT”) and the donation of chemicals from the BASF SE is gratefully acknowledged.
