# Supplementary material for: Combining silver- and organocatalysis: an enantioselective sequential catalytic approach towards pyrano-annulated pyrazoles
Source: Chem Commun (Camb). 2015 Feb 11;51(12):2266–9. doi: 10.1039/c4cc09495f (PMC4611987; doi:10.1039/c4cc09495f)
Supplement: Supplementary file 1 [file CC-051-C4CC09495F-s001.pdf]

Electronic Supplementary Material (ESI) for Chemical Communications.
This journal is © The Royal Society of Chemistry 2015

Sample Name: YM 26-1 rac
Data file: D:\GONZO\YM\26-1R2AD.D
Sample Info: Laufmittel: n-Heptan/IP 9:1;
Die Probe ist in EtOH/DCM/LM gelöst.

HEWLETT PACKARD

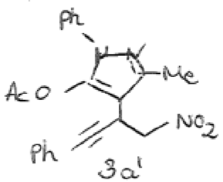

3a'

Säule: DAICELAD.M
Säuleninfo: Chiralpak AD (250x4,6)mm
Operator: Analytik Labor AKEN

Injektion Time: 11:06:50
Injektion Date: 18.03.2014

Instrument Conditions:	At Start	At Stop
Temperature in°C:	30.0°C	30.0°C
Pressure in bar:	16.8	16.9
Flow in ml/min:	0.70	0.70

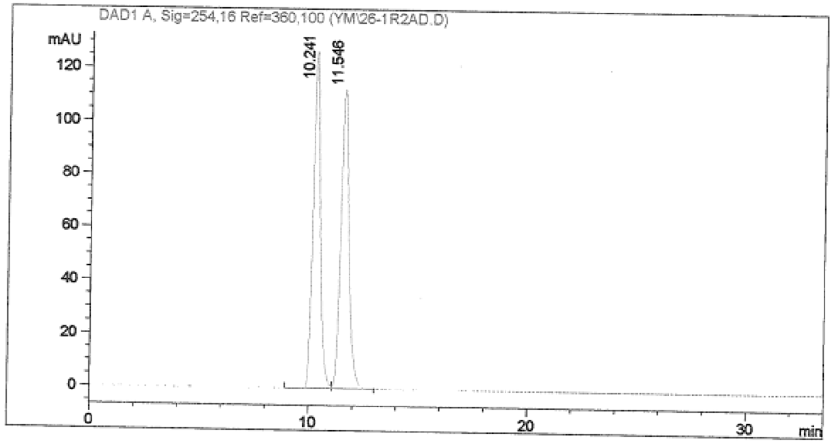

#	Ret. Time (min)	Width	Height (mAU)	Area (mAU*s)	Area %
1	10.24	0.35	126.55	2922.80	49.94
2	11.55	0.39	112.50	2929.73	50.06
Total				5852.53	100.00

Sample Name: YM 78-1
Data file: D:\GONZO\YM\78-1AD.D
Sample Info: Laufmittel: n-Heptan/IP 9:1;
Die Probe ist in LM/DCM gelöst

HEWLETT PACKARD

97

Säule: DAICELAD.M
Säuleninfo: Chiralpak AD (250x4,6)mm
Operator: Analytik Labor AKEN

Injektion Time: 11:44:29
Injektion Date: 21.05.2014

Instrument Conditions:	At Start	At Stop
Temperature in°C:	30.0°C	30.0°C
Pressure in bar:	15.9	15.9
Flow in ml/min:	0.70	0.70

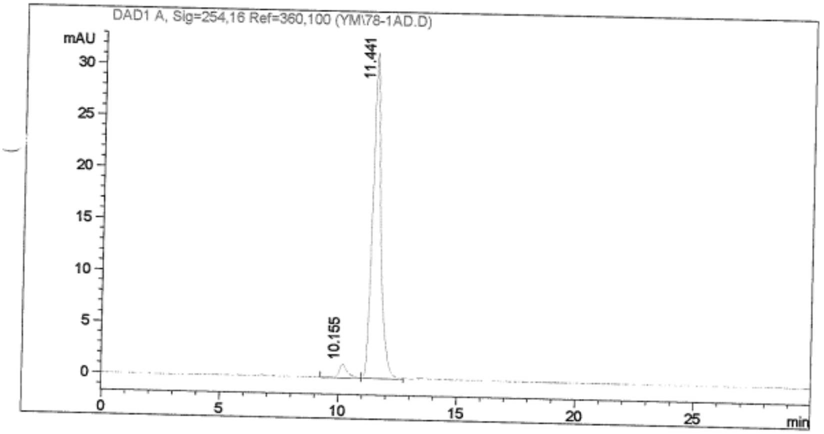

#	Ret. Time (min)	Width	Height (mAU)	Area (mAU*s)	Area %
1	10.15	0.35	1.33	32.34	3.99
2	11.44	0.36	31.51	777.20	96.01
Total				809.54	100.00

Sample Name: YM ~~21~~ 31-2 rac
Data file: D:\GONZO\PC\31-2RAD.D
Sample Info: Laufmittel: n-Heptan/IP 97:3;
Die Probe ist in LM/DCM gelöst

HEWLETT PACKARD

Säule: DAICELAD.M
Säuleninfo: Chiralpak AD (250x4,6)mm
Operator: Analytik Labor AKEN

Injektion Time: 10:51:52
Injektion Date: 24.03.2014

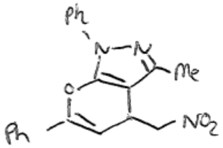

4a

Instrument Conditions:	At Start	At Stop
Temperature in°C:	30.0°C	30.0°C
Pressure in bar:	15.8	16.3
Flow in ml/min:	0.70	0.70

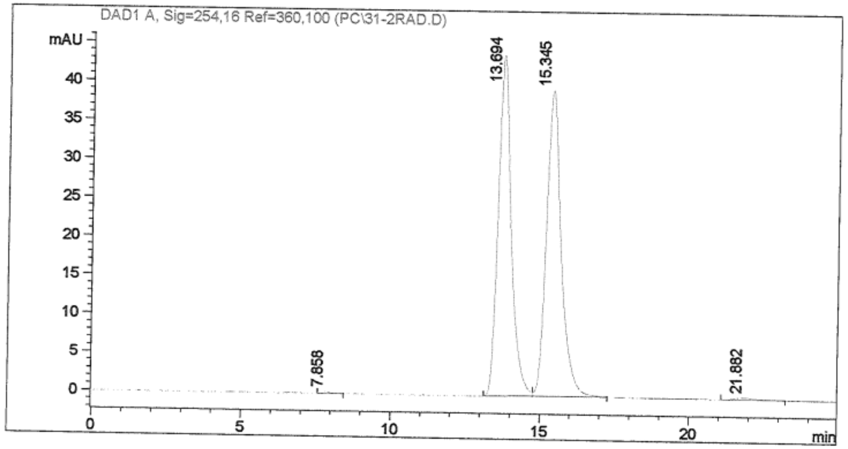

#	Ret. Time (min)	Width	Height (mAU)	Area (mAU*s)	Area %
1	7.86	0.25	0.25	4.62	0.17
2	13.69	0.46	43.92	1343.13	49.26
3	15.34	0.52	39.40	1365.03	50.06
4	21.88	0.64	0.27	13.92	0.51
Total				2726.69	100.00

Sample Name: KD 973 A1
Data file: D:\GONZO\KD\973A1AD.D
Sample Info: Laufmittel: n-Heptan/iPrOH 97:3;
Die Probe ist in DCM/LM gelöst.

HEWLETT PACKARD

Säule: DAICELAD.M
Säuleninfo: Chiralpak AD (250x4,6)mm
Operator: Analytik Labor AKEN

Injektion Time: 11:45:56
Injektion Date: 13.11.2014

Instrument Conditions:	At Start	At Stop
Temperature in°C:	30.0°C	30.0°C
Pressure in bar:	15.9	16.3
Flow in ml/min:	0.70	0.70

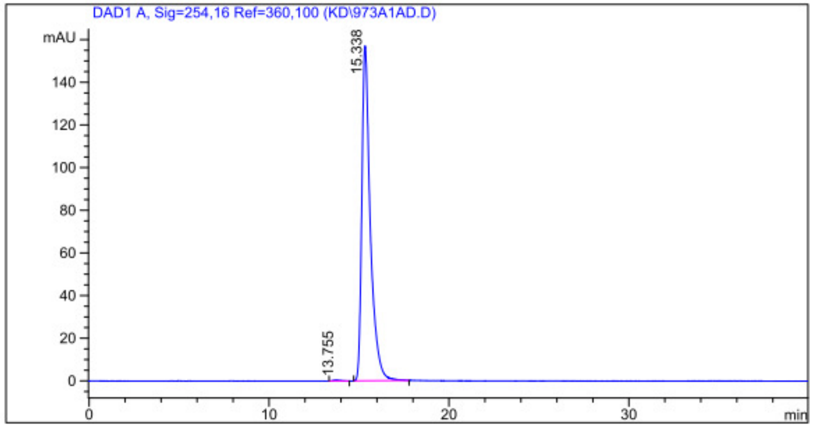

#	Ret. Time (min)	Width	Height (mAU)	Area (mAU*s)	Area %
1	13.75	0.32	0.39	9.97	0.19
2	15.34	0.48	157.03	5150.56	99.81
Total				5160.53	100.00

Sample Name: KD 959 A rac
Data file: D:\GONZO\KD\959AROD.D
Sample Info: Laufmittel: n-Heptan/EtOH 8:2;
Die Probe ist in LM gelöst

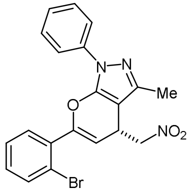

Säule: DAICELOD.M
Säuleninfo: Chiralcel OD (250x4,6)mm
Operator: Analytik Labor AKEN

Injektion Time: 12:32:12
Injektion Date: 24.09.2014

Instrument Conditions:	At Start	At Stop
Temperature in°C:	30.0°C	30.0°C
Pressure in bar:	33.2	34.1
Flow in ml/min:	1.00	1.00

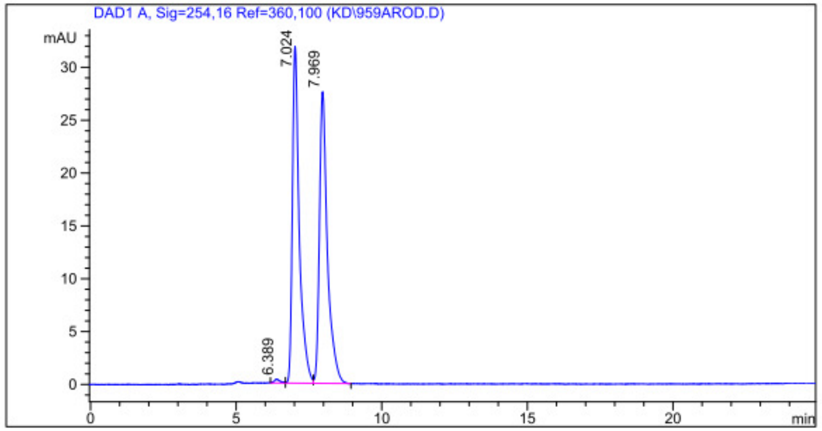

#	Ret. Time (min)	Width	Height (mAU)	Area (mAU*s)	Area %
1	6.39	0.21	0.36	5.29	0.50
2	7.02	0.24	31.85	524.86	49.66
3	7.97	0.28	27.59	526.67	49.84
Total				1056.81	100.00

Sample Name: KD 982 A
Data file: D:\GONZO\KD\982AOD.D
Sample Info: Laufmittel: n-Heptan/EtOH 8:2;
Die Probe ist in DCM/LM gelöst

Säule: DAICELOD.M
Säuleninfo: Chiralcel OD (250x4,6)mm
Operator: Analytik Labor AKEN

Injektion Time: 11:57:00
Injektion Date: 31.10.2014

Instrument Conditions:	At Start	At Stop
Temperature in°C:	30.0°C	30.0°C
Pressure in bar:	33.6	34.5
Flow in ml/min:	1.00	1.00

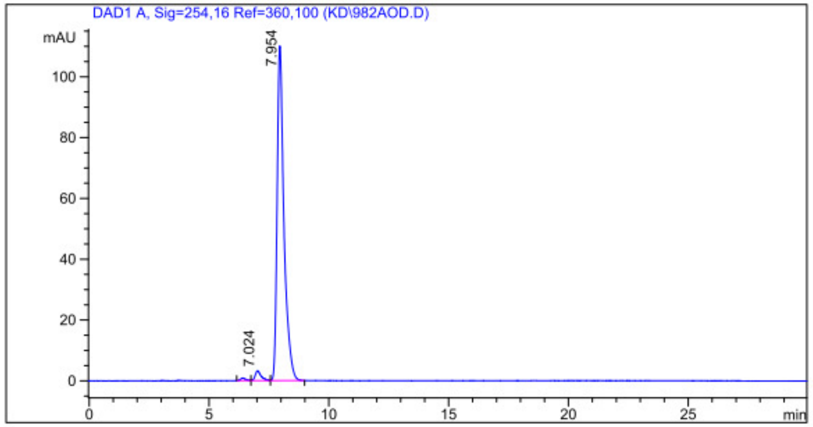

#	Ret. Time (min)	Width	Height (mAU)	Area (mAU*s)	Area %
1	6.40	0.22	0.87	13.17	0.59
2	7.02	0.26	3.22	56.34	2.51
3	7.95	0.29	110.00	2178.70	96.91
Total				2248.21	100.00

Sample Name: KD 929 A rac
Data file: D:\GONZO\KD\929ARAD.D
Sample Info: Laufmittel: n-Heptan/iPrOH 9:1;
Die Probe ist in DCM/LM gelöst.

Säule: DAICELAD.M
Säuleninfo: Chiralpak AD (250x4,6)mm
Operator: Analytik Labor AKEN

Injektion Time: 11:14:07
Injektion Date: 07.10.2014

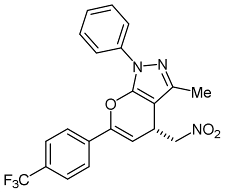

Instrument Conditions:	At Start	At Stop
Temperature in°C:	30.0°C	30.0°C
Pressure in bar:	25.7	26.4
Flow in ml/min:	1.00	1.00

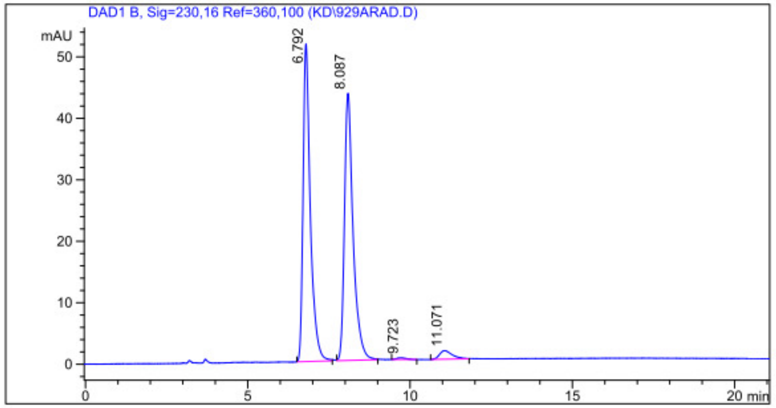

#	Ret. Time (min)	Width	Height (mAU)	Area (mAU*s)	Area %
1	6.79	0.23	51.66	802.86	48.56
2	8.09	0.27	43.47	806.23	48.76
3	9.72	0.29	0.27	5.44	0.33
4	11.07	0.40	1.40	38.95	2.36
Total				1653.47	100.00

Sample Name: KD 976 A1
Data file: D:\GONZO\KD\976A1AD.D
Sample Info: Laufmittel: n-Heptan/iPrOH 9:1;
Die Probe ist in DCM/LM gelöst.

Säule: DAICELAD.M
Säuleninfo: Chiralpak AD (250x4,6)mm
Operator: Analytik Labor AKEN

Injektion Time: 10:28:16
Injektion Date: 14.11.2014

Instrument Conditions:	At Start	At Stop
Temperature in°C:	30.0°C	30.0°C
Pressure in bar:	26.4	27.0
Flow in ml/min:	1.00	1.00

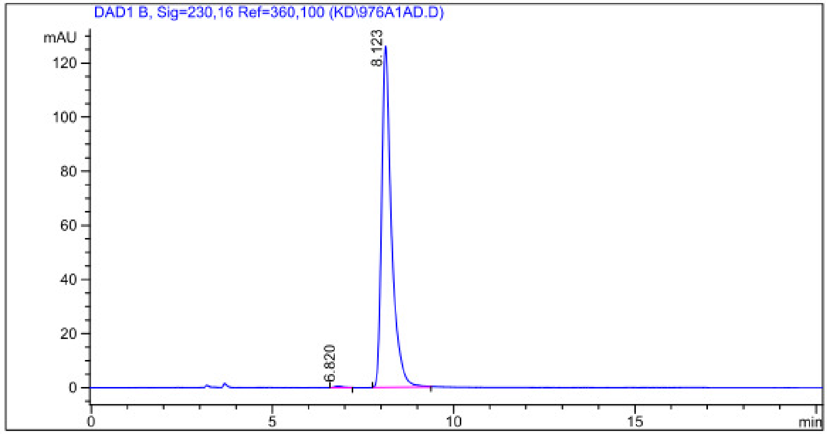

#	Ret. Time (min)	Width	Height (mAU)	Area (mAU*s)	Area %
1	6.82	0.22	0.47	7.29	0.31
2	8.12	0.28	126.14	2374.81	99.69
Total				2382.10	100.00

Sample Name: KD 955 A rac
Data file: D:\GONZO\KD\955ARAD.D
Sample Info: Laufmittel: n-Heptan/iPrOH 97:3;
Die Probe ist in DCM/LM gelöst.

HEWLETT PACKARD

Säule: DAICELAD.M
Säuleninfo: Chiralpak AD (250x4,6)mm
Operator: Analytik Labor AKEN

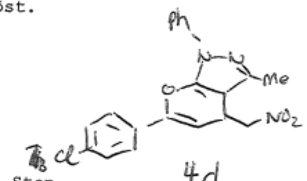

4d

Injektion Time: 11:15:21
Injektion Date: 22.09.2014

Instrument Conditions:	At Start	At Stop
Temperature in°C:	30.0°C	30.0°C
Pressure in bar:	24.5	24.7
Flow in ml/min:	1.00	1.00

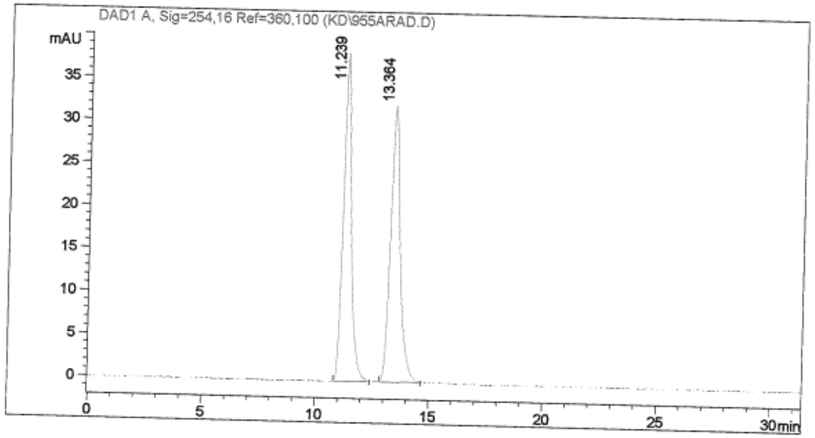

#	Ret. Time (min)	Width	Height (mAU)	Area (mAU*s)	Area %
1	11.24	0.38	38.23	963.71	49.95
2	13.36	0.45	32.18	965.62	50.05
Total				1929.33	100.00

Sample Name: KD 980 A1
Data file: D:\GONZO\KD\980A1AD.D
Sample Info: Laufmittel: n-Heptan/iPrOH 97:3;
Die Probe ist in DCM/LM gelöst.

HEWLETT PACKARD

Säule: DAICELAD.M
Säuleninfo: Chiralpak AD (250x4,6)mm
Operator: Analytik Labor AKEN

Injektion Time: 11:32:45
Injektion Date: 17.11.2014

Instrument Conditions:	At Start	At Stop
Temperature in°C:	30.0°C	30.0°C
Pressure in bar:	25.0	26.0
Flow in ml/min:	1.00	1.00

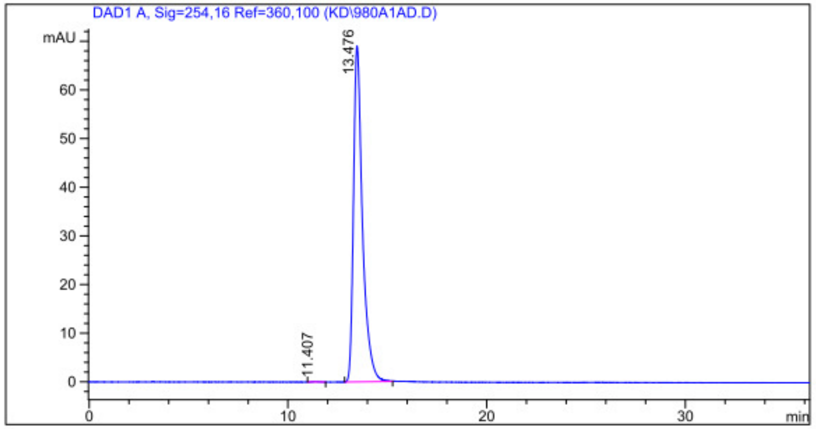

#	Ret. Time (min)	Width	Height (mAU)	Area (mAU*s)	Area %
1	11.41	0.39	0.08	1.94	0.09
2	13.48	0.47	69.00	2172.05	99.91
Total				2174.00	100.00

Sample Name: KD 956 A rac
Data file: D:\BERT\KD\956AROJ.D
Sample Info: Laufmittel: n-Heptan/EtOH 7:3;
Die Probe ist in DCM/LM gelöst

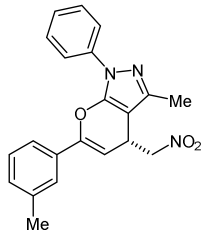

Säule: DAICELOJ.M
Säuleninfo: Chiralcel OJ (250x4,6)mm

Operator: Analytik Labor AKEN

Injektion Time: 12:14:51
Injektion Date: 24.09.2014

Instrument Conditions:	At Start	At Stop
Temperature in°C:	30.0	30.0
Pressure in bar:	49.0	47.6
Flow in ml/min:	1.0	1.0

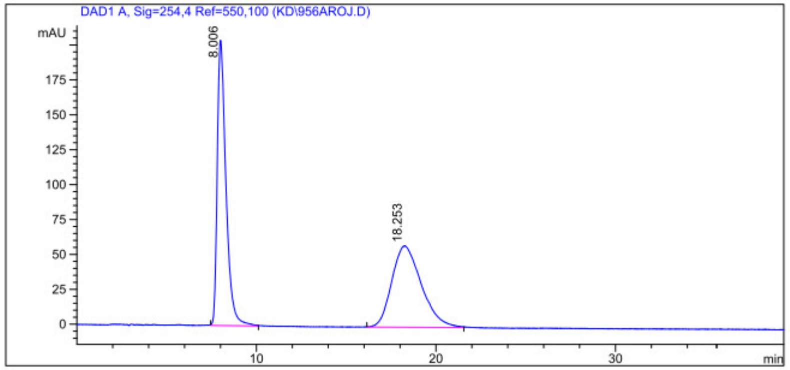

#	Ret. Time (min)	Width	Height (mAU)	Area (mAU*s)	Area %
1	8.01	0.51	204.11	6884.29	50.81
2	18.25	1.70	58.22	6665.27	49.19
Total				13549.57	100.00

Sample Name: KD 979 A2
Data file: D:\BERT\KD\979A2OJ.D
Sample Info: Laufmittel: n-Heptan/EtOH 7:3;
Die Probe ist in DCM/LM gelöst

Säule: DAICELOJ.M
Säuleninfo: Chiralcel OJ (250x4,6)mm

Operator: Analytik Labor AKEN

Injektion Time: 09:40:39
Injektion Date: 17.11.2014

Instrument Conditions:	At Start	At Stop
Temperature in°C:	30.0	30.0
Pressure in bar:	47.6	47.5
Flow in ml/min:	1.0	1.0

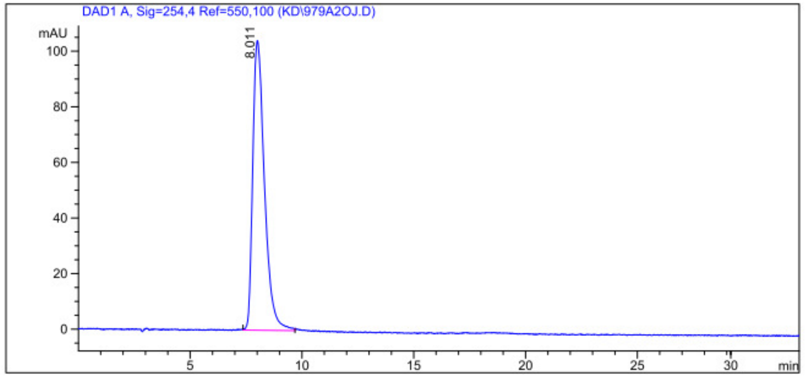

#	Ret. Time (min)	Width	Height (mAU)	Area (mAU*s)	Area %
1	8.01	0.56	104.13	3806.31	100.00
Total				3806.31	100.00

Sample Name: KD 926 A rac
Data file: D:\GONZO\KD\926AR1OD.D
Sample Info: Laufmittel: n-Heptan/iPrOH 9:1;
Die Probe ist in DCM/LM gelöst.

Säule: DAICELOD.M
Säuleninfo: Chiralcel OD (250x4,6)mm
Operator: Analytik Labor AKEN

Injektion Time: 15:11:57
Injektion Date: 06.10.2014

Instrument Conditions:	At Start	At Stop
Temperature in°C:	30.0°C	30.0°C
Pressure in bar:	29.2	29.6
Flow in ml/min:	1.00	1.00

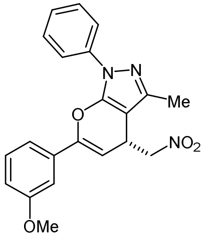

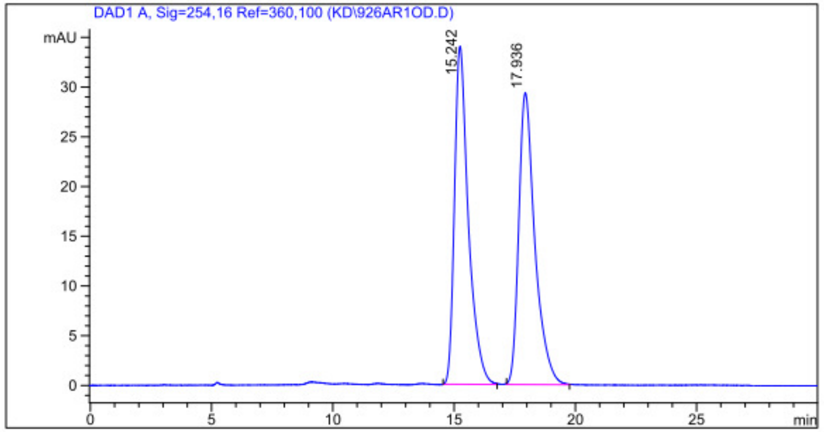

#	Ret. Time (min)	Width	Height (mAU)	Area (mAU*s)	Area %
1	15.24	0.59	33.98	1352.02	49.92
2	17.94	0.69	29.33	1356.46	50.08
Total				2708.48	100.00

Sample Name: KD 977 A1
Data file: D:\GONZO\KD\977A1OD.D
Sample Info: Laufmittel: n-Heptan/iPrOH 9:1;
Die Probe ist in DCM/LM gelöst.

Säule: DAICELOD.M
Säuleninfo: Chiralcel OD (250x4,6)mm
Operator: Analytik Labor AKEN

Injektion Time: 10:12:11
Injektion Date: 13.11.2014

Instrument Conditions:	At Start	At Stop
Temperature in°C:	30.0°C	30.0°C
Pressure in bar:	29.7	30.3
Flow in ml/min:	1.00	1.00

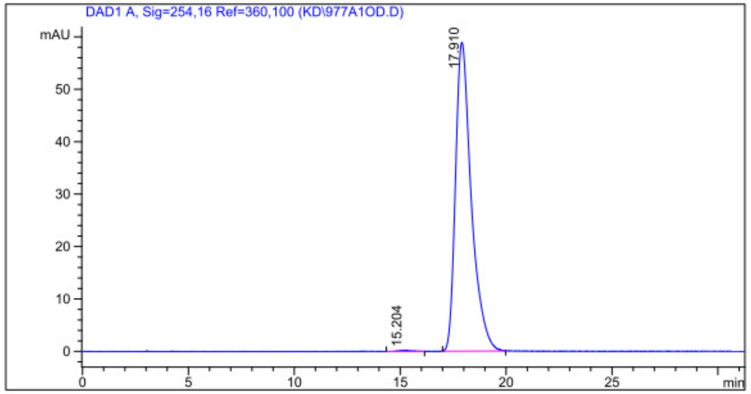

#	Ret. Time (min)	Width	Height (mAU)	Area (mAU*s)	Area %
1	15.20	0.85	0.21	10.75	0.35
2	17.91	0.78	58.87	3058.36	99.65
Total				3069.11	100.00

Sample Name: KD 923 A rac
Data file: D:\GONZO\KD\923AR1AD.D
Sample Info: Laufmittel: n-Heptan/EtOH 95:5;
Die Probe ist in DCM/LM gelöst.

Säule: DAICELAD.M
Säuleninfo: Chiralpak AD (250x4,6)mm
Operator: Analytik Labor AKEN

Injektion Time: 15:12:08
Injektion Date: 22.09.2014

Instrument Conditions:	At Start	At Stop
Temperature in°C:	30.0°C	30.0°C
Pressure in bar:	24.3	24.8
Flow in ml/min:	1.00	1.00

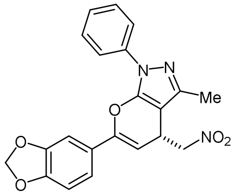

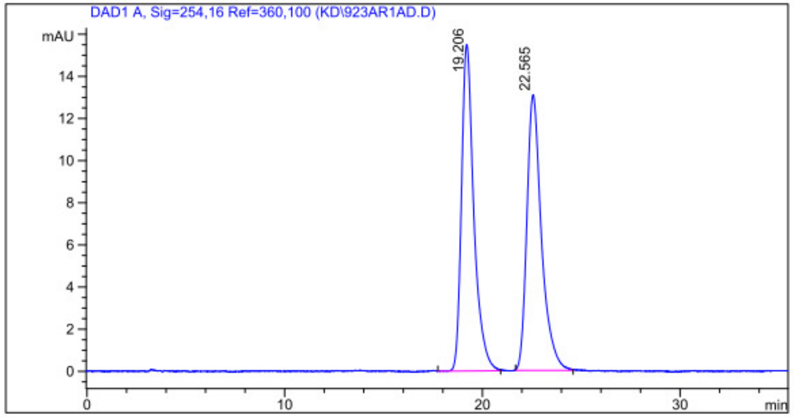

#	Ret. Time (min)	Width	Height (mAU)	Area (mAU*s)	Area %
1	19.21	0.65	15.49	674.02	50.12
2	22.56	0.77	13.08	670.80	49.88
Total				1344.81	100.00

Sample Name: KD 981 A
Data file: D:\GONZO\KD\981AAD.D
Sample Info: Laufmittel: n-Heptan/EtOH 95:5;
Die Probe ist in LM/DCM gelöst

Säule: DAICELAD.M
Säuleninfo: Chiralpak AD (250x4,6)mm
Operator: Analytik Labor AKEN

Injektion Time: 11:46:33
Injektion Date: 30.10.2014

Instrument Conditions:	At Start	At Stop
Temperature in°C:	30.0°C	30.0°C
Pressure in bar:	25.1	25.5
Flow in ml/min:	1.00	1.00

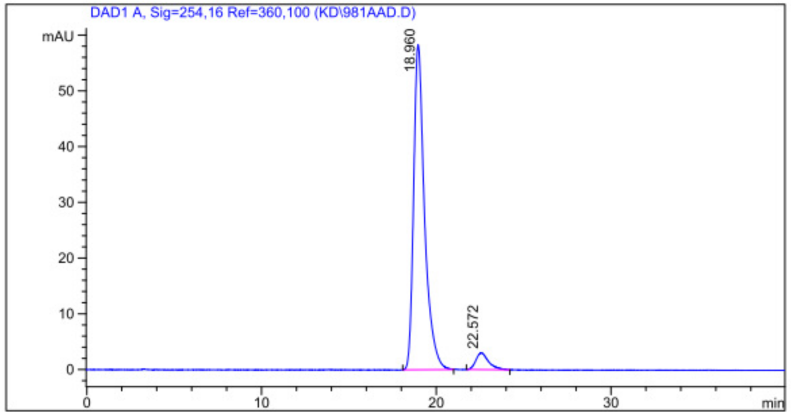

#	Ret. Time (min)	Width	Height (mAU)	Area (mAU*s)	Area %
1	18.96	0.65	58.32	2539.30	94.32
2	22.57	0.72	3.01	153.00	5.68
Total				2692.30	100.00

Sample Name: KD 958 A rac
Data file: D:\BERT\KD\958AR1OJ.D
Sample Info: Laufmittel: n-Heptan/EtOH 7:3;
Die Probe ist in DCM/LM gelöst

Agilent Technologies

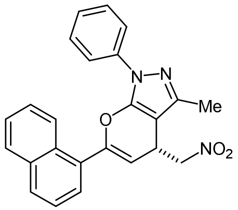

Säule: DAICELOJ.M
Säuleninfo: Chiralcel OJ (250x4,6)mm

Operator: Analytik Labor AKEN

Injektion Time: 09:02:19
Injektion Date: 10.10.2014

Instrument Conditions:	At Start	At Stop
Temperature in°C:	30.0	30.0
Pressure in bar:	47.4	47.3
Flow in ml/min:	1.0	1.0

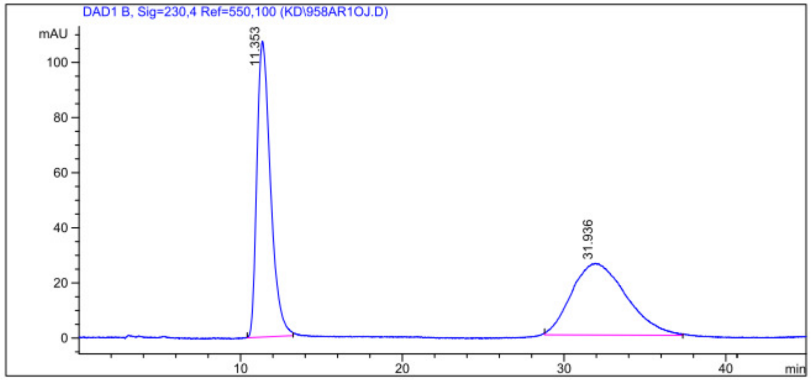

#	Ret. Time (min)	Width	Height (mAU)	Area (mAU*s)	Area %
1	11.35	0.85	107.33	6265.99	50.70
2	31.94	2.76	26.00	6092.04	49.30
Total				12358.03	100.00

Sample Name: KD 983 A
Data file: D:\BERT\KD\983AOJ.D
Sample Info: Laufmittel: n-Heptan/EtOH 7:3;
Die Probe ist in DCM/LM gelöst

Agilent Technologies

Säule: DAICELOJ.M
Säuleninfo: Chiralcel OJ (250x4,6)mm

Operator: Analytik Labor AKEN

Injektion Time: 08:36:44
Injektion Date: 31.10.2014

Instrument Conditions:	At Start	At Stop
Temperature in°C:	30.0	30.0
Pressure in bar:	47.9	47.6
Flow in ml/min:	1.0	1.0

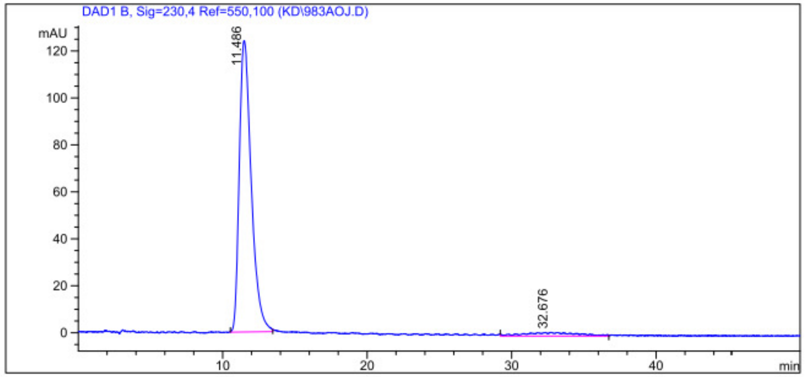

#	Ret. Time (min)	Width	Height (mAU)	Area (mAU*s)	Area %
1	11.49	0.85	124.03	7143.99	94.69
2	32.68	4.15	1.61	400.95	5.31
Total				7544.94	100.00

Sample Name: KD 961 A rac
Data file: D:\GONZO\KD\961AR2AD.D
Sample Info: Laufmittel: n-Heptan/EtOH 97:3;
Die Probe ist in DCM/LM gelöst.

HEWLETT PACKARD

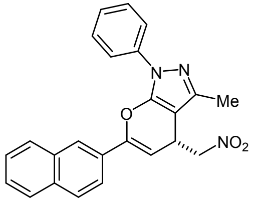

Säule: DAICELAD.M
Säuleninfo: Chiralpak AD (250x4,6)mm
Operator: Analytik Labor AKEN

Injektion Time: 10:11:15
Injektion Date: 08.10.2014

Instrument Conditions:	At Start	At Stop
Temperature in°C:	30.0°C	30.0°C
Pressure in bar:	24.2	24.8
Flow in ml/min:	1.00	1.00

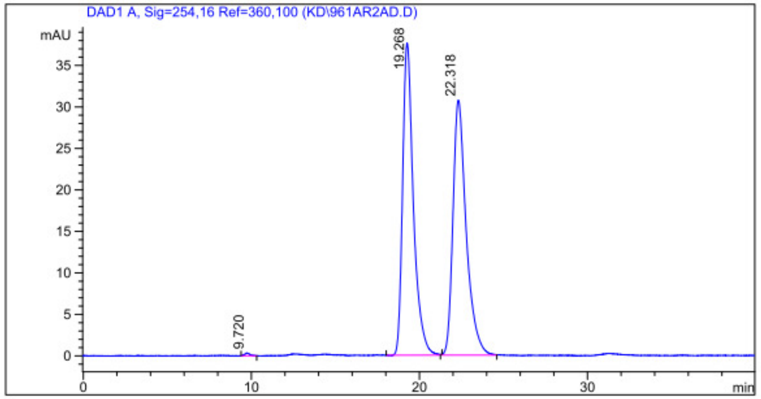

#	Ret. Time (min)	Width	Height (mAU)	Area (mAU*s)	Area %
1	9.72	0.30	0.33	6.98	0.21
2	19.27	0.67	37.61	1680.21	49.86
3	22.32	0.82	30.72	1682.39	49.93
Total				3369.59	100.00

Sample Name: KD 978 A
Data file: D:\GONZO\KD\978AAD.D
Sample Info: Laufmittel: n-Heptan/EtOH 97:3;
Die Probe ist in DCM/LM gelöst.

HEWLETT PACKARD

Säule: DAICELAD.M
Säuleninfo: Chiralpak AD (250x4,6)mm
Operator: Analytik Labor AKEN

Injektion Time: 12:53:46
Injektion Date: 29.10.2014

Instrument Conditions:	At Start	At Stop
Temperature in°C:	30.0°C	30.0°C
Pressure in bar:	24.9	25.3
Flow in ml/min:	1.00	1.00

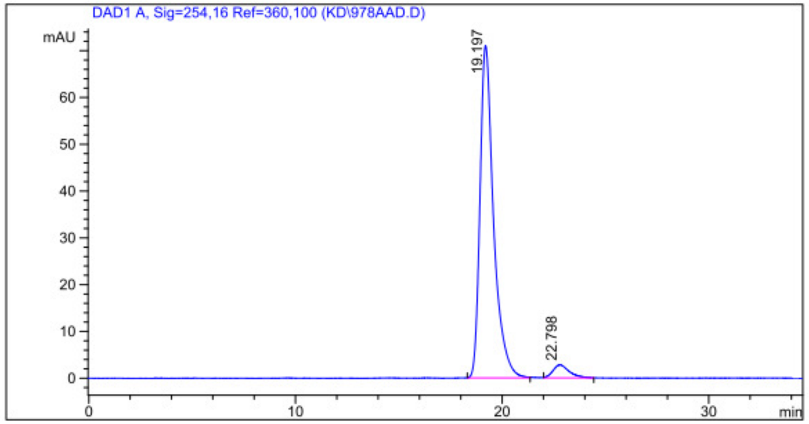

#	Ret. Time (min)	Width	Height (mAU)	Area (mAU*s)	Area %
1	19.20	0.68	71.02	3239.38	95.40
2	22.80	0.78	2.82	156.37	4.60
Total				3395.75	100.00

Sample Name: KD 997 A
Data file: D:\GONZO\KD\997AAD.D
Sample Info: Laufmittel: n-Heptan/EtOH 97:3;
Die Probe ist in DCM/LM gelöst.

Säule: DAICELAD.M
Säuleninfo: Chiralpak AD (250x4,6)mm
Operator: Analytik Labor AKEN

Injektion Time: 12:52:31
Injektion Date: 17.11.2014

Instrument Conditions:	At Start	At Stop
Temperature in°C:	30.0°C	30.0°C
Pressure in bar:	24.8	25.4
Flow in ml/min:	1.00	1.00

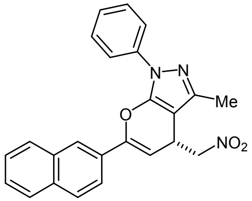

gram scale

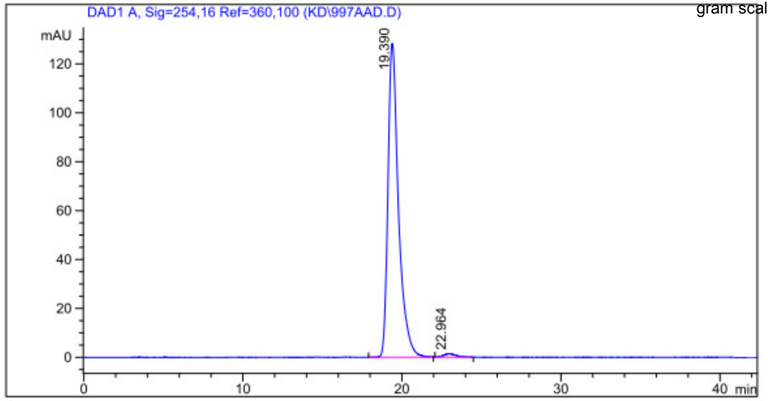

#	Ret. Time (min)	Width	Height (mAU)	Area (mAU*s)	Area %
1	19.39	0.69	128.34	5911.15	98.60
2	22.96	0.77	1.43	83.79	1.40
Total				5994.94	100.00

Sample Name: KD 964 A rac
Data file: D:\BERT\KD\964AROJ.D
Sample Info: Laufmittel: n-Heptan/EtOH 7:3;
Die Probe ist in DCM/LM gelöst

Agilent Technologies

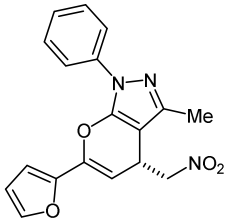

Säule: DAICELOJ.M
Säuleninfo: Chiralcel OJ (250x4,6)mm

Operator: Analytik Labor AKEN

Injektion Time: 14:10:35
Injektion Date: 22.10.2014

Instrument Conditions:	At Start	At Stop
Temperature in°C:	30.0	30.0
Pressure in bar:	48.2	47.6
Flow in ml/min:	1.0	1.0

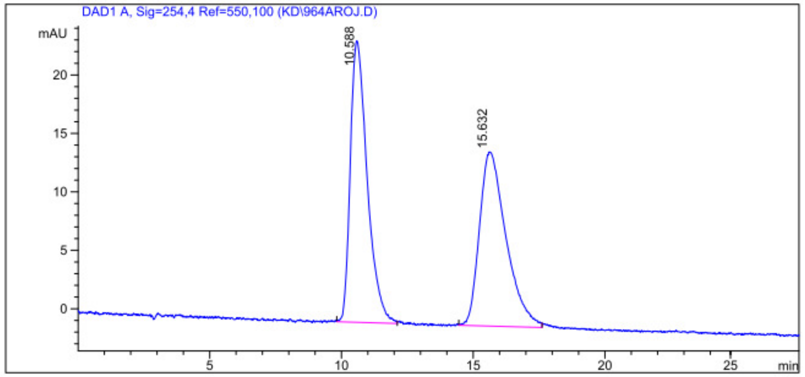

#	Ret. Time (min)	Width	Height (mAU)	Area (mAU*s)	Area %
1	10.59	0.70	24.06	1074.71	50.72
2	15.63	1.03	14.88	1044.25	49.28
Total				2118.95	100.00

Sample Name: KD 984 A1
Data file: D:\BERT\KD\984A1OJ.D
Sample Info: Laufmittel: n-Heptan/EtOH 7:3;
Die Probe ist in DCM/LM gelöst

Agilent Technologies

Säule: DAICELOJ.M
Säuleninfo: Chiralcel OJ (250x4,6)mm

Operator: Analytik Labor AKEN

Injektion Time: 10:25:52
Injektion Date: 13.11.2014

Instrument Conditions:	At Start	At Stop
Temperature in°C:	30.0	30.0
Pressure in bar:	47.6	47.4
Flow in ml/min:	1.0	1.0

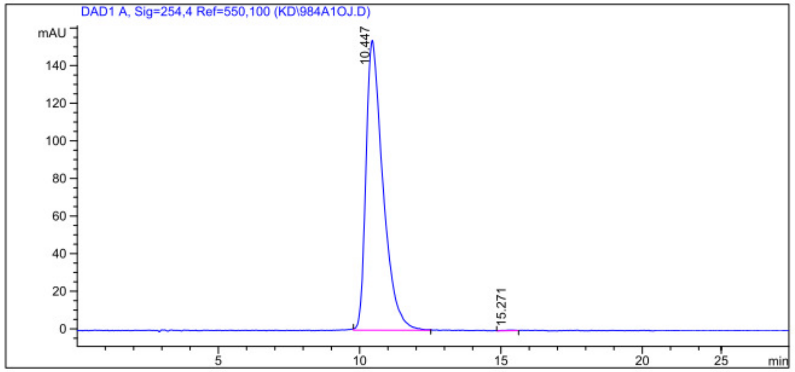

#	Ret. Time (min)	Width	Height (mAU)	Area (mAU*s)	Area %
1	10.45	0.63	154.09	6443.63	99.86
2	15.27	0.45	0.34	9.33	0.14
Total				6452.96	100.00

```
Sample Name:     KD 965 A rac
Data file:       D:\BERT\KD\965AROJ.D
Sample Info:     Laufmittel: n-Heptan/EtOH 7:3;
                 Die Probe ist in DCM/LM gelöst

Säule:           DAICELOJ.M
Säuleninfo:      Chiralcel OJ (250x4,6)mm

Operator:        Analytik Labor AKEN

Injektion Time:      14:39:40
Injektion Date:      22.10.2014

Instrument Conditions:    At Start          At Stop

Temperature in°C:         30.0              30.0
Pressure in bar:          47.7              47.4
Flow in ml/min:           1.0               1.0
```

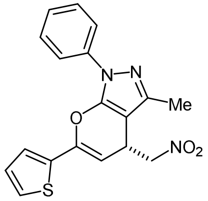

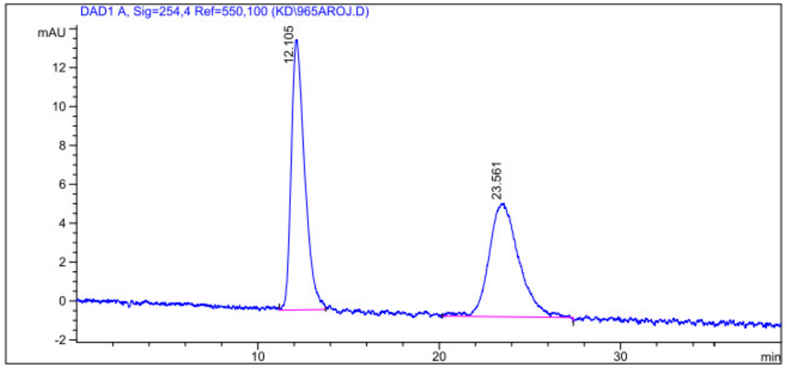

#	Ret. Time (min)	Width	Height (mAU)	Area (mAU*s)	Area %
1	12.11	0.86	13.92	714.58	51.59
2	23.56	1.92	5.83	670.65	48.41
Total				1385.23	100.00

```
Sample Name:     KD 991 A1
Data file:       D:\BERT\KD\991A1OJ.D
Sample Info:     Laufmittel: n-Heptan/EtOH 7:3;
                 Die Probe ist in DCM/LM gelöst

Säule:           DAICELOJ.M
Säuleninfo:      Chiralcel OJ (250x4,6)mm

Operator:        Analytik Labor AKEN

Injektion Time:      10:14:57
Injektion Date:      17.11.2014

Instrument Conditions:    At Start          At Stop

Temperature in°C:         30.0              30.0
Pressure in bar:          48.3              47.3
Flow in ml/min:           1.0               1.0
```

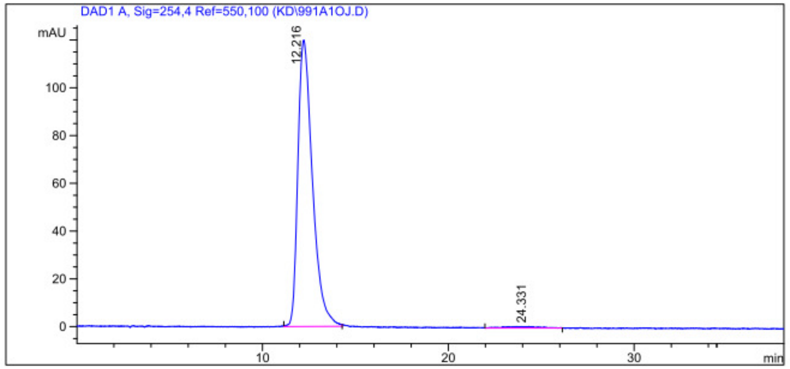

#	Ret. Time (min)	Width	Height (mAU)	Area (mAU*s)	Area %
1	12.22	0.82	119.87	6533.81	98.78
2	24.33	2.03	0.66	80.74	1.22
Total				6614.55	100.00

UNIVERSITY

Sample name: KD 966 A rac

Data file: C:\SNOOPY\KD\966ARXIA.D

Description: Laufmittel: n-Heptan/EtOH 9:1; Probe ist in LM/DCM gelöst.

Injection date: 10/24/2014 10:20:56 AM

Acq. Analysis method: CHIRALPAKIARNP.M

Column: Chiralpak IA, (250 x 4,6) mm, 5μ, SN: IA00CE-RC036

Pressure at start: 25 bar Start flow: 0.500 ml/min Column oven: 30 °C

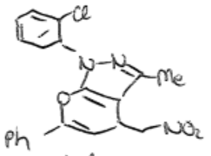

4l

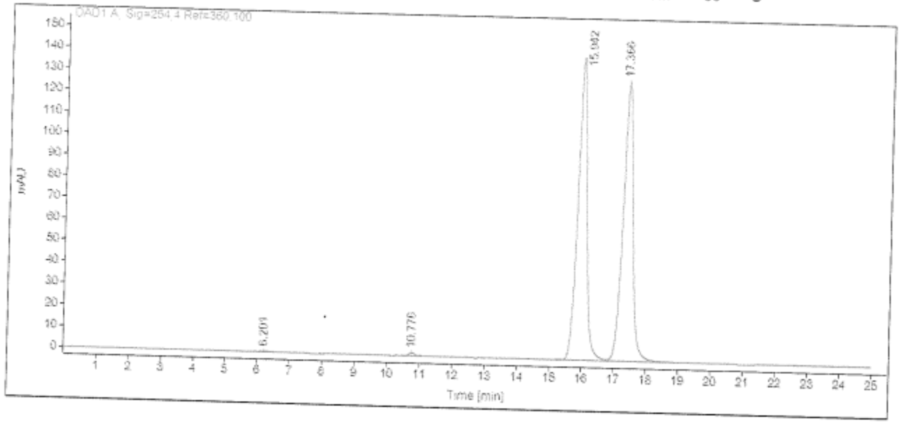

Name KD 966 A rac

RT [min]	Type	Area%	Area	Height	Width [min]
6.20	BB	0.08	4.30	0.48	0.14
10.78	BB	0.34	19.14	1.62	0.18
15.94	BV	49.67	2794.43	140.94	0.30
17.37	VB	49.91	2807.98	129.90	0.33
	Sum	100.00	5625.86		

UNIVERSITY

Sample name: KD 985 A

Data file: C:\SNOOPY\KD\KD 985 A IA.D

Description: Laufmittel: n-Heptan/EtOH 9:1 Die Probe ist DCM/LM gelöst.

Injection date: 11/3/2014 10:32:38 AM

Acq. Analysis method: CHIRALPAKIARNP.M

Column: Chiralpak IA, (250 x 4,6) mm, 5μ, SN: IA00CE-RC036

Pressure at start: 25 bar Start flow: 0.500 ml/min Column oven: 30.01 °C

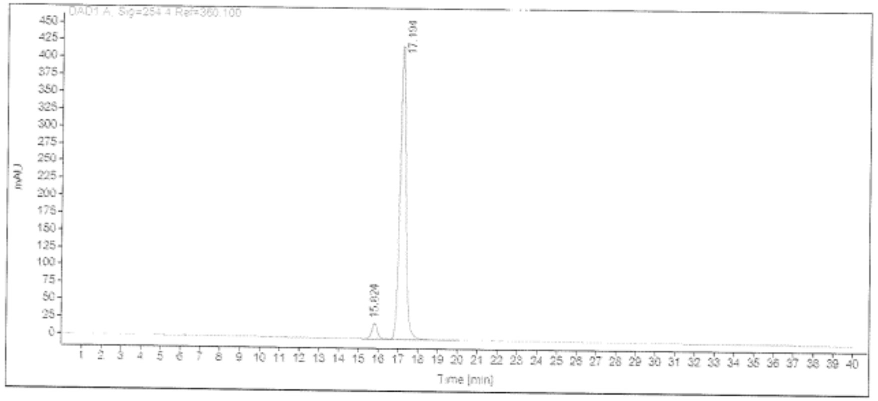

Name KD 985 A

RT [min]	Type	Area%	Area	Height	Width [min]
15.82	BV	4.68	443.87	22.66	0.30
17.19	VB	95.32	9039.40	423.52	0.33
	Sum	100.00	9483.26		

Sample Name: KD 970 A rac
Data file: D:\BERT\KD\970AR1OJ.D
Sample Info: Laufmittel: n-Heptan/EtOH 7:3;
Die Probe ist in DCM/LM gelöst

Säule: DAICELOJ.M
Säuleninfo: Chiralcel OJ (250x4,6)mm

Operator: Analytik Labor AKEN

Injektion Time: 10:35:54
Injektion Date: 29.10.2014

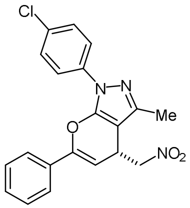

Instrument Conditions:	At Start	At Stop
Temperature in°C:	30.0	30.0
Pressure in bar:	33.0	33.1
Flow in ml/min:	0.7	0.7

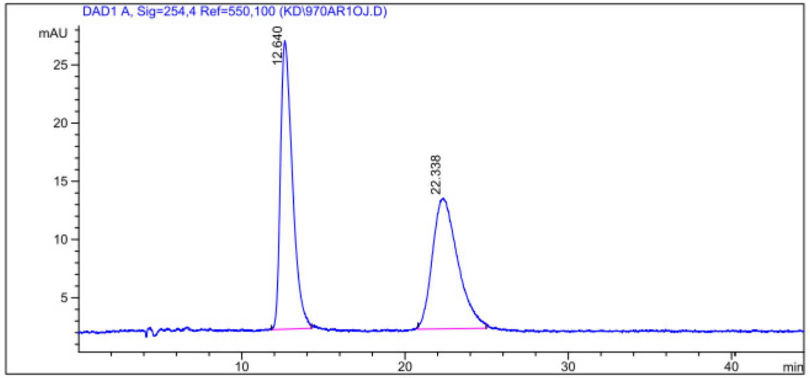

#	Ret. Time (min)	Width	Height (mAU)	Area (mAU*s)	Area %
1	12.64	0.74	24.79	1260.42	50.96
2	22.34	1.28	11.20	1212.93	49.04
Total				2473.35	100.00

Sample Name: KD 988 A1
Data file: D:\BERT\KD\988A1OJ.D
Sample Info: Laufmittel: n-Heptan/EtOH 7:3;
Die Probe ist in DCM/LM gelöst

Säule: DAICELOJ.M
Säuleninfo: Chiralcel OJ (250x4,6)mm

Operator: Analytik Labor AKEN

Injektion Time: 10:53:20
Injektion Date: 13.11.2014

Instrument Conditions:	At Start	At Stop
Temperature in°C:	30.0	30.0
Pressure in bar:	32.8	32.6
Flow in ml/min:	0.7	0.7

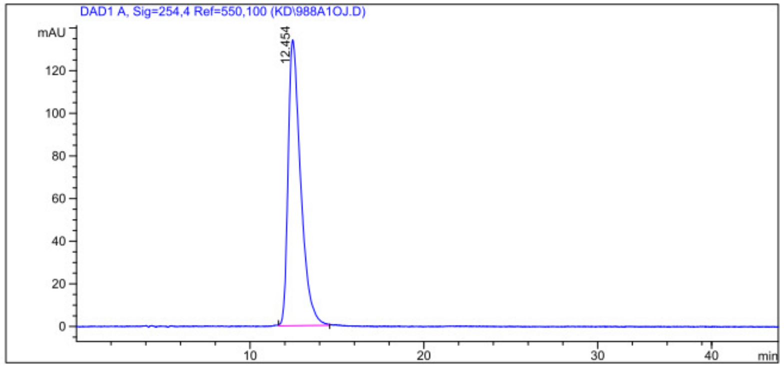

#	Ret. Time (min)	Width	Height (mAU)	Area (mAU*s)	Area %
1	12.45	0.74	134.08	6713.31	100.00
Total				6713.31	100.00

Sample Name: KD 963 A rac
Data file: D:\GONZO\KD\963ARAD.D
Sample Info: Laufmittel: n-Heptan/iPrOH 9:1;
Die Probe ist in DCM/LM gelöst.

HEWLETT PACKARD

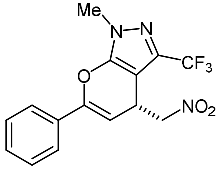

Säule: DAICELAD.M
Säuleninfo: Chiralpak AD (250x4,6)mm
Operator: Analytik Labor AKEN

Injektion Time: 10:02:04
Injektion Date: 27.10.2014

Instrument Conditions:	At Start	At Stop
Temperature in°C:	30.0°C	30.0°C
Pressure in bar:	26.0	26.3
Flow in ml/min:	1.00	1.00

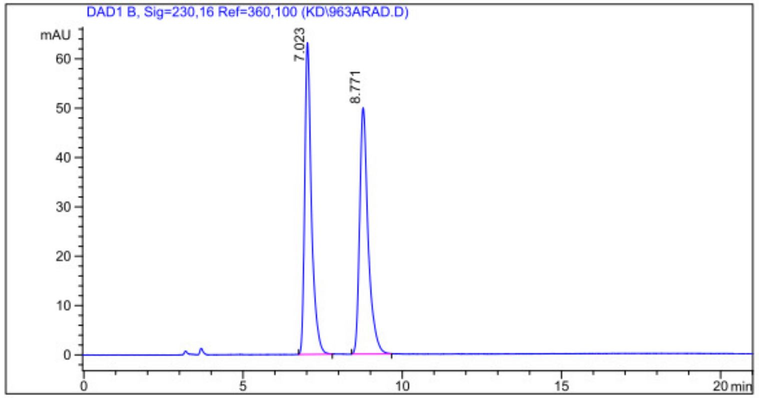

#	Ret. Time (min)	Width	Height (mAU)	Area (mAU*s)	Area %
1	7.02	0.22	63.15	931.13	50.01
2	8.77	0.28	49.90	930.94	49.99
Total				1862.07	100.00

Sample Name: KD 986 A1
Data file: D:\GONZO\KD\986A1AD.D
Sample Info: Laufmittel: n-Heptan/iPrOH 9:1;
Die Probe ist in DCM/LM gelöst.

HEWLETT PACKARD

Säule: DAICELAD.M
Säuleninfo: Chiralpak AD (250x4,6)mm
Operator: Analytik Labor AKEN

Injektion Time: 15:49:22
Injektion Date: 17.11.2014

Instrument Conditions:	At Start	At Stop
Temperature in°C:	30.0°C	30.0°C
Pressure in bar:	26.6	26.9
Flow in ml/min:	1.00	1.00

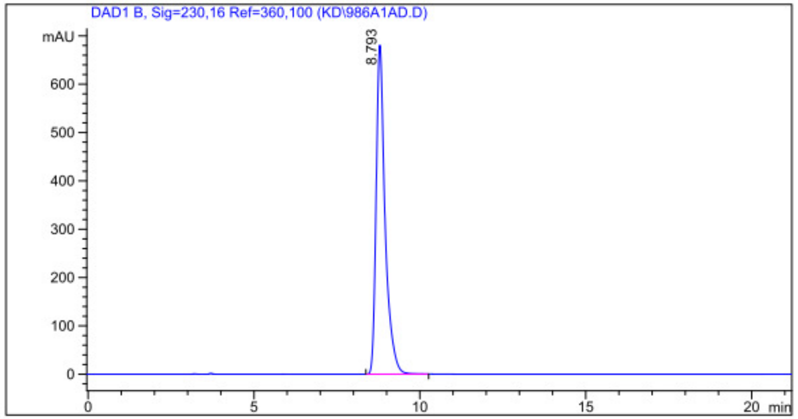

#	Ret. Time (min)	Width	Height (mAU)	Area (mAU*s)	Area %
1	8.79	0.28	680.67	12811.64	100.00
Total				12811.64	100.00

Sample name: KD 969 A rac

Data file: C:\SNOOPY\KD\KD 969 A RAC IA.D

Description: Laufmittel: n-Heptan/EtOH 9:1 Die Probe ist DCM/LM gelöst.

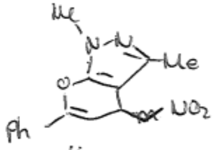

40

Injection date: 10/28/2014 10:12:08 AM

Acq. Analysis method: CHIRALPAKIARNP.M

Column: Chiralpak IA, (250 x 4,6) mm, 5µ, SN: IA00CE-RC036

Pressure at start: 51 bar Start flow: 1.000 ml/min Column oven: 29.99 °C

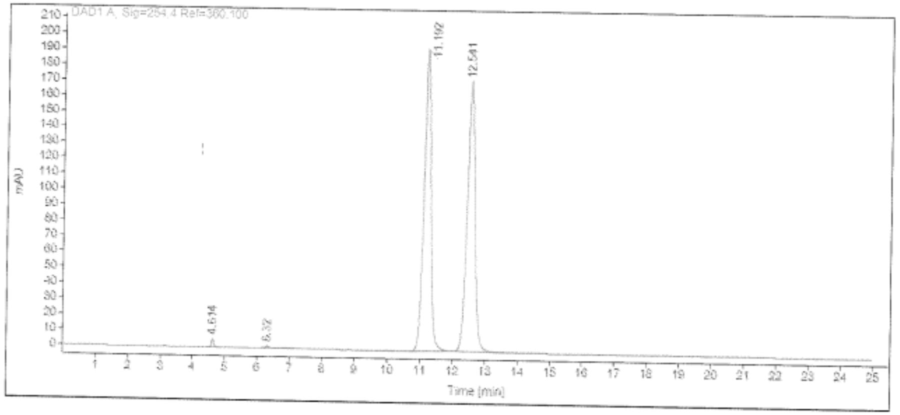

Name KD 969 A rac

RT [min]	Type	Area%	Area	Height	Width [min]
4.61	BV	0.48	27.86	5.25	0.08
6.32	BB	0.19	10.89	1.21	0.13
11.19	BB	49.64	2888.63	193.22	0.23
12.54	BB	49.69	2891.73	172.44	0.26
	Sum	100.00	5819.11		

Sample name: KD 996 A1

Data file: C:\SNOOPY\KD\KD 996 A1 IA.D

Description: Laufmittel: n-Heptan/EtOH 9:1 Die Probe ist DCM/LM gelöst.

Injection date: 11/18/2014 1:15:57 PM

Acq. Analysis method: CHIRALPAKIARNP.M

Column: Chiralpak IA, (250 x 4,6) mm, 5µ, SN: IA00CE-RC036

Pressure at start: 51 bar Start flow: 1.000 ml/min Column oven: 30 °C

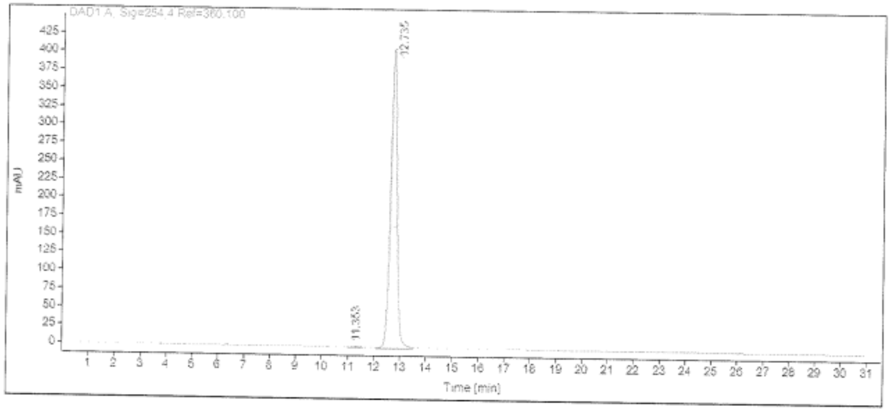

Name KD 996 A1

RT [min]	Type	Area%	Area	Height	Width [min]
11.35	MM	0.33	23.25	1.37	0.28
12.73	MM	99.67	7111.50	411.92	0.29
	Sum	100.00	7134.74		

Sample name: KD 968 A rac

Data file: C:\SNOOPY\KD\968ARXIA.D

Description: Laufmittel: n-Heptan/EtOH 9:1;
Probe ist in LM/DCM gelöst.

Injection date: 10/24/2014 10:47:02 AM

Acq. Analysis method: CHIRALPAKIARNP.M

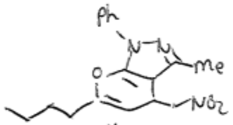

4p

Column: Chiralpak IA, (250 x 4,6) mm, 5μ, SN: IA00CE-RC036

Pressure at start: 26 bar Start flow: 0.500 ml/min Column oven: 29.99 °C

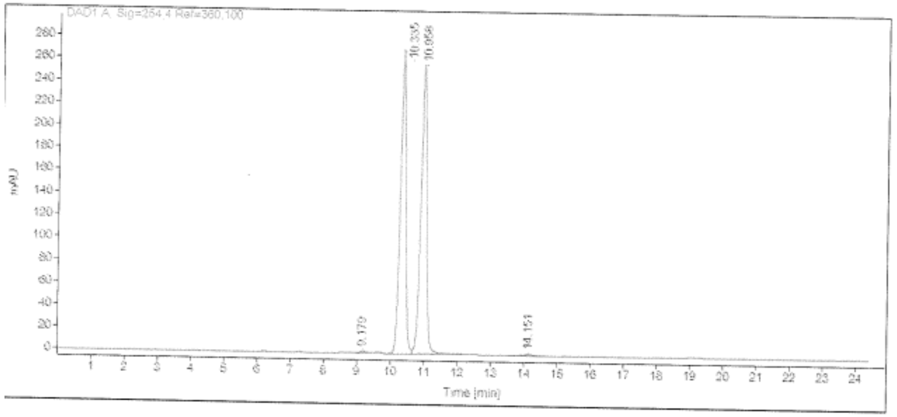

Name KD 968 A rac

RT [min]	Type	Area%	Area	Height	Width [min]
9.18	VB	0.29	18.18	1.69	0.17
10.33	BV	49.25	3106.08	270.62	0.18
10.96	VB	50.12	3161.09	256.32	0.19
14.15	BB	0.34	21.19	1.09	0.31
	Sum	100.00	6306.54		

Sample name: KD 995 A1

Data file: C:\SNOOPY\KD\KD 995 A1 IA.D

Description: Laufmittel: n-Heptan/EtOH 9:1 Die Probe ist DCM/LM gelöst.

Injection date: 11/19/2014 9:05:28 AM

Acq. Analysis method: CHIRALPAKIARNP.M

Column: Chiralpak IA, (250 x 4,6) mm, 5μ, SN: IA00CE-RC036

Pressure at start: 26 bar Start flow: 0.500 ml/min Column oven: 29.99 °C

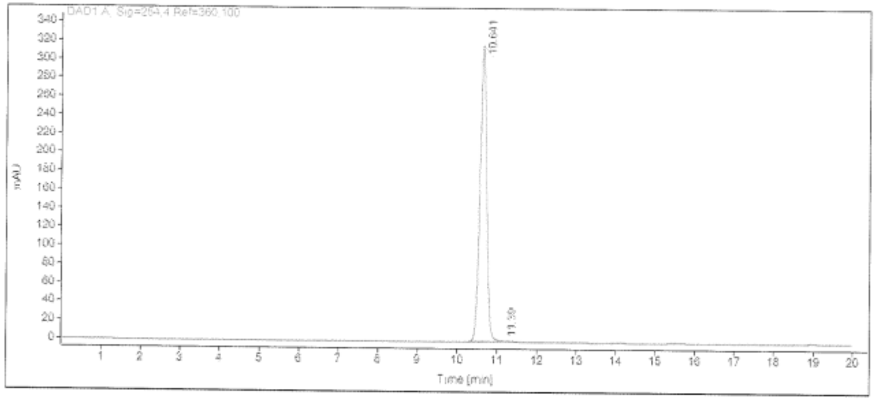

Name KD 995 A1

RT [min]	Type	Area%	Area	Height	Width [min]
10.64	MM	99.90	3713.02	316.73	0.20
11.39	MM	0.10	3.86	0.32	0.20
	Sum	100.00	3716.88		

Sample Name: KD 967 A rac
Data file: D:\GONZO\KD\967AR1OD.D
Sample Info: Laufmittel: n-Heptan/iPrOH 9:1;
Die Probe ist in DCM/LM gelöst.

Säule: DAICELOD.M
Säuleninfo: Chiralcel OD (250x4,6)mm
Operator: Analytik Labor AKEN

Injektion Time: 11:01:35
Injektion Date: 23.10.2014

Instrument Conditions:	At Start	At Stop
Temperature in°C:	30.0°C	30.0°C
Pressure in bar:	12.4	12.5
Flow in ml/min:	0.50	0.50

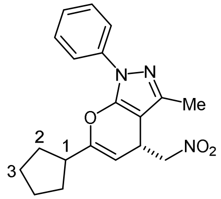

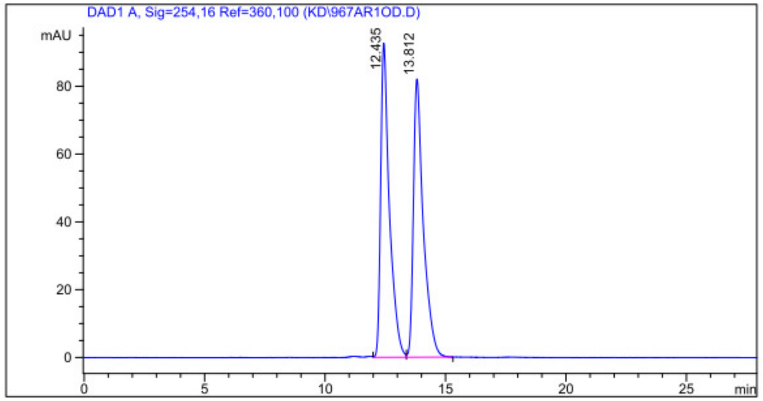

#	Ret. Time (min)	Width	Height (mAU)	Area (mAU*s)	Area %
1	12.43	0.36	92.56	2338.99	49.85
2	13.81	0.41	82.02	2353.53	50.15
Total				4692.52	100.00

Sample Name: KD 992 A1
Data file: D:\GONZO\KD\992A1OD.D
Sample Info: Laufmittel: n-Heptan/iPrOH 9:1;
Die Probe ist in DCM/LM gelöst

Säule: DAICELOD.M
Säuleninfo: Chiralcel OD (250x4,6)mm
Operator: Analytik Labor AKEN

Injektion Time: 16:46:06
Injektion Date: 17.11.2014

Instrument Conditions:	At Start	At Stop
Temperature in°C:	30.0°C	30.0°C
Pressure in bar:	12.7	12.9
Flow in ml/min:	0.50	0.50

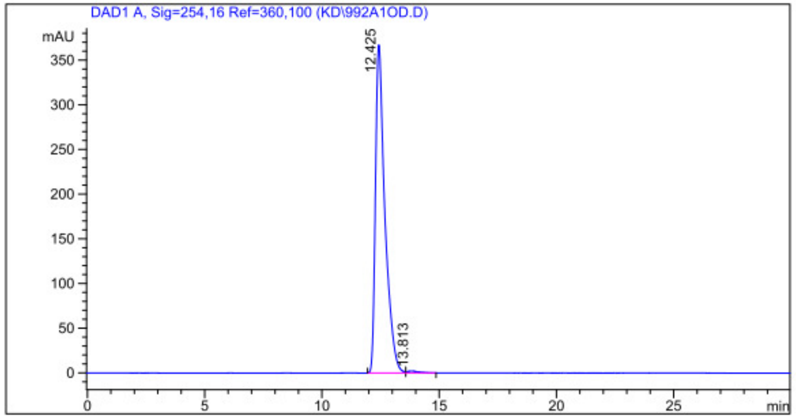

#	Ret. Time (min)	Width	Height (mAU)	Area (mAU*s)	Area %
1	12.43	0.41	367.05	10326.38	99.29
2	13.81	0.47	2.14	73.70	0.71
Total				10400.08	100.00
