# Supplementary material for: Combining silver- and organocatalysis: an enantioselective sequential catalytic approach towards pyrano-annulated pyrazoles
Source: Chem Commun (Camb). 2015 Feb 11;51(12):2266–9. doi: 10.1039/c4cc09495f (PMC4611987; doi:10.1039/c4cc09495f)
Supplement: Supplementary file 2 [file CC-051-C4CC09495F-s002.pdf]

Electronic Supplementary Material (ESI) for Chemical Communications.
This journal is © The Royal Society of Chemistry 2015

Combining silver- and organocatalysis: an enantioselective sequential catalytic approach towards pyrano-annulated pyrazoles

Daniel Hack, Pankaj Chauhan, Kristina Deckers, Yusuke Mizutani, Gerhard Raabe, Dieter Enders*

Institute of Organic Chemistry, RWTH Aachen University, Landoltweg 1, 52074 Aachen, Germany

Supporting Information

Content

General Information 2

General Procedures and Analytical Data 3

NMR spectra 25

General Information

Unless otherwise noted, all commercially available compounds were used without further purification. For preparative column chromatography SIL G-25 UV252 from Macherey-Nagel, particle size 0.040-0.063 nm (230-240 mesh. flash) was used. Visualization of the developed TLC plates was performed with UV irradiation (254 nm) or by staining with $KMnO_4$. Optical rotations were measured on a Perkin-Elmer 241 polarimeter. Mass spectra were recorded on a Finnigan SSQ7000 (EI 70 eV) spectrometer and high-resolution mass spectra on a Thermo Fisher Scientific Orbitrap XL spectrometer. IR spectra were recorded on a Perkin-Elmer FT-IR Spectrum 100 using ATR-Unit. ^{1}H, ^{13}C and ^{19}F spectra were recorded at ambient temperature on Varian Mercury 300, Inova 400, Varian VNMRS-400, or Varian VNMRS-600 spectrometers with TMS as an internal standard. Analytical HPLC was performed on a Hewlett-Packard 1100 Series instrument using chiral stationary phases (Daicel AD, Daicel AS, Daicel IA, Daicel OD, Daicel OJ or Chiralpak IC columns).

Abbreviations:

**OR**	optical rotation
**EA**	elemental analysis
**CH_{Ar}**	aromatic proton
**CH_{ol}**	olefinic proton
**{^{19}F}**	^{19}F decoupled spectra
**{^{1}H}**	^{1}H decoupled spectra

General Procedures and Analytical Data

Synthesis of nitrolefines:

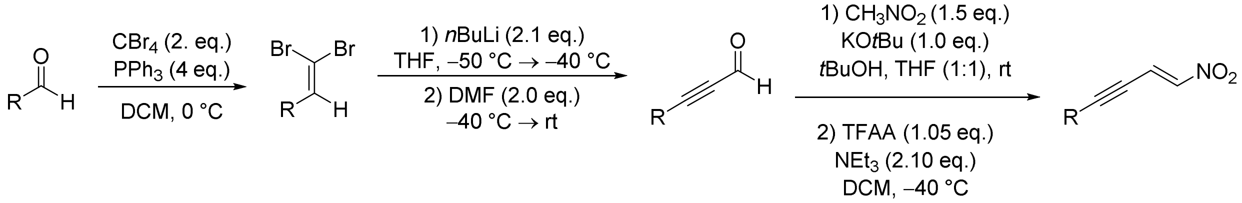

General procedure for the preparation of *gem*-dibromoolefines (first step):

Under an atmosphere of argon, a solution of triphenylphosphine (4 eq.) and tetrabromomethane (2 eq.) in abs. DCM (0.15 M) was stirred at 0 °C for 30 minutes. Then the aldehyde was added over a period of five minutes, and the mixture as stirred at 0 °C for one hour. After addition of water, the layers were separated, and the aqueous layer was extracted with DCM (three times). The combined organic layers were dried over $MgSO_4$ and the solvent was removed under reduced pressure. The crude product was dry-loaded on silica and subjected to flash chromatography (silica, *n*-pentane/DCM).

General procedure for the Corey-Fuchs homologisation (second step):

Under an atmosphere of argon, *n*-BuLi (2.1 eq., 1.6 M in *n*-hexane) was added over a period of 30 minutes *via* syringe pump to a solution of *gem*-dibromoolefine (1 eq.) in abs THF (0.4 M) at –50 °C, and the mixture was stirred at –40 °C for 15 minutes. After addition of DMF (2.0 eq.) at once, the mixture was allowed to warm to room temperature and stirred for one hour. The mixture was added to a stirring solution of NaH_2PO_4 (aq.)/MTBE (1:1). After five minutes, the layers were separated and the aqueous layer was extracted with MTBE. The combined organic layers were dried over $MgSO_4$, the solvent was removed under reduced pressure and the crude product was subjected to flash chromatography (silica, *n*-pentane/EtOAc).

General procedure for the preparation of nitro olefines (third step):

Under an atmosphere of argon, nitromethane (1.5 eq.) and KO*t*Bu (1 eq.) were added to a solution of aldehyde (1 eq.) in a solution of THF and *t*BuOH (1:1, 0.5 M), and the mixture was stirred overnight. Then a solution of NH_4Cl was added, the product was extracted twice with Et_2O, the combined organic phases were washed with brine and dried over $MgSO_4$. The solvent was removed under reduced pressure and the crude product was subjected to flash chromatography on silica (*n*-pentane/Et_2O 5:1 to 1:1).

Under an atmosphere of argon, TFAA (1.05 eq.) was added over a period of 30 min to a solution of nitroalcohol (1 eq.) in DCM (0.5 M) at –40 °C. Then triethylamine (2.1 eq.) was added, and the mixture was stirred at –0 °C for 45 min. The reaction was quenched with a solution of NH_4Cl was

added, the liquid phases were separated, and the aqueous phase was extracted twice with DCM. The combined organic phases were washed with a saturated solution of $NaHCO_3$ and brine, and dried over Na_2SO_4. The solvent was removed under reduced pressure and the product was purified by flash chromatography on silica (*n*-pentane/Et_2O 5:1).

R	yield (1st step) [%]	yield (2nd step) [%]	yield (3rd step)[1][%]
Ph (**1a**)	-	-	50
4-Br-C_6H_5 (**1b**)	96	-	32
4-F_3C-C_6H_5 (**1c**)	83	69	53
2-Cl-C_6H_5 (**1d**)	94	59	53
3-Me-C_6H_5 (**1e**)	99	87	41
3-MeO-C_6H_5 (**1f**)	94	79	52
3,4-OCH_2O-C_6H_3 (**1g**)	61	83	40
1-naphthyl (**1h**)	58	75	55
2-naphthyl (**1i**)	91	75	45
1-furanyl (**1j**)	95	23	52
2-thienyl (**1k**)	97	24	32
cyclopentyl (**2p**)	-	88	58
butyl (**2q**)	85	84	58

(*E*)-(4-Nitrobut-3-en-1-yn-1-yl)benzene [1a]

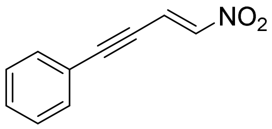

Compound **1a** was isolated after flash chromatography (SiO_2, *n*-pentane/Et_2O 5:1) as yellow solid (979 mg, 70%)[2]. **Molecular formula:** $C_{10}H_7NO_2$. **Molecular mass:** 173.17 g mol^{-1}. **R_f** (*n*-pentane /EtOAc: 50:1) = 0.23. **Mp:** 24-27 °C. **^{1}H NMR** (600 MHz, $CDCl_3$): δ = 7.35-7.37 (m, 2 H, CH_{ol}), 7.37-7.42 (m, 2 H, CH_{Ar}), 7.42-7.47 (m, 1 H, CH_{Ar}), 7.51-7.54 (m, 2 H, CH_{Ar}) ppm. **^{13}C NMR** (151 MHz, $CDCl_3$): δ = 82.0 (s), 105.2 (s), 121.2 (d), 121.3 (s), 128.8 (d, 2 C), 130.5 (d), 132.4 (d, 2 C), 145.7 (d) ppm. **IR** (ATR): $\tilde{\upsilon}$ = 3541, 3109, 3035, 2954, 2845, 2663, 2325, 2198, 2107, 1998, 1758, 1674, 1613, 1517, 1444, 1335, 1173, 1036, 934, 835, 757, 688 cm^{-1}. **MS (EI$^+$, 70 eV)** *m/z* (%): 174.2 (14), 173.2 $[M]^+$ = $[C_{10}H_7NO_2]^+$, 127.2 (19), 126.1 (100) $[M-HNO_2]^+$ = $[C_{10}H_6]^+$, 115.1 (12), 105.1 (29), 77.2 (20). **MS (CI$^+$, methane)** *m/z* (%): 202.3 (10) $[M+C_2H_5]^+$ = $[C_{12}H_{12}NO_2]^+$, 174.2 (88) $[M+H]^+$ = $[C_{10}H_8NO_2]^+$. **EA**: calcd. for $C_{10}H_7NO_2$: C: 69.36%, H: 4.07%, N: 8.09%; found: C: 69.26%, H: 4.32%, N: 8.34%.

---

[1] Yields given for both steps of the nitro olefine synthesis.
[2] Yield refers to the last step of the synthesis (elimination).

(*E*)-1-Bromo-2-(4-nitrobut-3-en-1-yn-1-yl)benzene [1b]

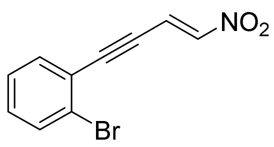

Compound **1b** was isolated after flash chromatography (SiO_2, *n*-pentane/Et_2O 5:1) as yellow solid (559 mg, 72%). **Molecular formula:** $C_{10}H_6BrNO_2$. **Molecular mass:** 252.07 g mol^{-1}. $\mathbf{R_f}$ (*n*-pentane /EtOAc: 50:1) = 0.18. **Mp:** 37-42 °C. ^{1}**H NMR** (600 MHz, $CDCl_3$): δ = 7.29 (dt, *J* = 1.7 Hz, *J* = 7.8 Hz, 1 H, CH_{Ar}), 7.34 (dt, *J* = 1.1 Hz, *J* = 7.6 Hz, 1 H, CH_{Ar}), 7.37-7.44 (m, 2 H, CH_{ol}), 7.54 (dd, *J* = 1.6 Hz, *J* = 7.7 Hz, 1 H, CH_{Ar}), 7.64 (dd, *J* = 0.9 Hz, *J* = 8.0 Hz, 1 H) ppm. ^{13}**C NMR** (151 MHz, $CDCl_3$): δ = 85.8 (s), 102.8 (s), 120.6 (d), 123.6 (s), 126.3 (s), 127.5 (d), 131.6 (d), 133.0 (d), 134.2 (d), 146.2 (d) ppm. **IR** (ATR): $\tilde{\nu}$ = 3533, 3104, 3032, 2921, 2836, 2577, 2486, 2299, 2194, 2120, 1988, 1940, 1858, 1741, 1687, 1612, 1580, 1555, 1510, 1427, 1335, 1269, 1160, 1117, 1057, 1020, 964, 964, 929, 929, 841, 755, 714 cm^{-1}. **MS (EI$^+$, 70 eV)** *m/z* (%): 254.0 (15), 253.0 (98) [M, ^{81}Br]$^+$ = $[C_{10}H_6BrNO_2]^+$, 252.0 (12), 251.0 (100) [M, ^{79}Br]$^+$ = $[C_{10}H_6BrNO_2]^+$, 206.0 (61) [M–HNO_2, ^{81}Br]$^+$ = $[C_{10}H_5Br]^+$, 204.0 (62) [M–HNO_2, ^{79}Br]$^+$ = $[C_{10}H_5Br]^+$, 185.0 (11), 183.0 (12), 126.1 (13). **MS (CI$^+$, methane)** *m/z* (%): 282.2 (12) [M+C_2H_5, ^{81}Br]$^+$ = $[C_{12}H_{11}BrNO_2]^+$, 280.0 (12) [M+C_2H_5, ^{79}Br]$^+$ = $[C_{12}H_{11}BrNO_2]^+$, 254.0 (96) [M+H, ^{81}Br]$^+$ = $[C_{10}H_7BrNO_2]^+$, 252.0 (96) [M+H, ^{79}Br]$^+$ = $[C_{10}H_7BrNO_2]^+$. **HR-MS (ESI$^+$)** *m/z* (%): calcd. for [M+Na, ^{79}Br]$^+$ = $[C_{10}H_6BrNaNO_2]^+$, 273.9474; found: 273.9474.

(*E*)-1-(4-Nitrobut-3-en-1-yn-1-yl)-4-(trifluoromethyl)benzene [1c]

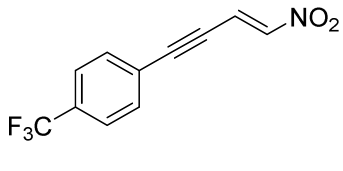

Compound **1c** was isolated after flash chromatography (SiO_2, *n*-pentane/Et_2O 5:1) as yellow solid (1.102 g, 74%). **Molecular formula:** $C_{11}H_6F_3NO_2$. **Molecular mass:** 241.17 g mol^{-1}. $\mathbf{R_f}$ (*n*-pentane /EtOAc: 50:1) = 0.23. **Mp:** 36-40 °C. ^{1}**H NMR** (400 MHz, $CDCl_3$): δ = 7.32-7.42 (m, 2 H, CH_{ol}), 7.59-7.68 (m, 4 H, CH_{Ar}) ppm. ^{13}**C{^{19}F} NMR** (100 MHz, $CDCl_3$): δ = 83.6 (s), 102.6 (s), 120.3 (d), 123.7 (s), 125.0 (s), 125.8 (d, 2 C), 132.0 (s), 132.6 (d, 2 C), 146.6 (d) ppm. ^{19}**F{^{1}H}** (376 MHz, $CDCl_3$): δ = −63.16 (s) ppm. **IR** (ATR): $\tilde{\nu}$ = 3559, 3102, 3040, 2928, 2832, 2204, 1928, 1799, 1677, 1615, 1615, 1510, 1405, 1313, 1227, 1163, 1110, 1065, 1025, 963, 936, 839, 752, 705 cm^{-1}. **MS (EI$^+$, 70 eV)** *m/z* (%): 241.3 (24) [M]$^+$ = $[C_{11}H_6F_3NO_2]^+$, 195.2 (15), 194.2 (100) [M–HNO_2]$^+$ = $[C_{11}H_5F_3]^+$, 193.1 (10), 175.1 (27) [M–$HFNO_2$]$^+$ = $[C_{11}H_5F_2]^+$, 173.1 (19), 145.1 (26) [M–$C_4H_2NO_2$]$^+$ = $[C_7H_4F_3]^+$. **MS (CI$^+$, methane)** *m/z* (%): 270.4 (26) [M+C_2H_5]$^+$ = $[C_{13}H_{11}F_3NO_2]^+$, 242.3 (100) [M+H]$^+$ = $[C_{11}H_7F_3NO_2]^+$. **EA**: calcd. for $C_{11}H_6F_3NO_{22}$: C: 54.78%, H: 2.51%, N: 5.81%; found: C: 54.59%, H: 2.42%, N: 5.54%.

(*E*)-1-Chloro-2-(4-nitrobut-3-en-1-yn-1-yl)benzene [1d]

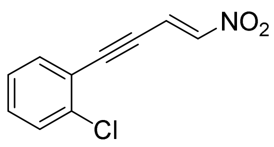

Compound **1d** was isolated after flash chromatography (SiO_2, *n*-pentane/Et_2O 5:1) as yellow solid (752 mg, 86%). **Molecular formula:** $C_{10}H_6ClNO_2$. **Molecular mass:** 207.61 g mol^{-1}. $\mathbf{R_f}$ (*n*-pentane /EtOAc: 50:1) = 0.20. **Mp:** 58-62 °C. ^{1}**H NMR** (600 MHz, $CDCl_3$): δ = 7.29 (dt, J = 1.1 Hz, J = 7.6 Hz, 1 H, CH_{Ar}), 7.37 (dt, J = 1.6 Hz, J = 7.8 Hz, 1 H, CH_{Ar}), 7.40 (s, 2 H, CH_{ol}), 7.46 (dd, J = 0.6 Hz, J = 8.1 Hz, 1 H, CH_{Ar}), 7.54 (dd, J = 1.5 Hz, J = 7.7 Hz, 1 H) ppm. ^{13}**C NMR** (151 MHz, $CDCl_3$): δ = 86.4 (s), 101.1 (s), 120.6 (d), 121.4 (s), 126.9 (d), 129.8 (d), 131.5 (d), 134.1 (d), 136.9 (s), 146.2 (d) ppm. **IR** (ATR): $\tilde{\upsilon}$ = 3896, 3855, 3749, 3667, 3539, 3221, 3168, 3107, 3036, 2947, 2834, 2667, 2578, 2485, 2406, 2324, 2236, 2197, 2117, 1987, 1941, 1908, 1859, 1859, 1813, 1740, 1581, 1502, 1431, 1336, 1270, 1226, 1159, 1124, 1067, 1023, 964, 929, 877, 838, 755, 728, 665 cm^{-1}. **MS (EI$^+$, 70 eV)** *m/z* (%): 209.2 (14) [M, $^{37}Cl]^+$ = $[C_{10}H_6ClNO_2]^+$, 207.2 (43) [M, $^{35}Cl]^+$ = $[C_{10}H_6ClNO_2]^+$, 162.1 (12), 161.1 (12), 160.1 (100), 139.1 (25), 126.1 (21), 125.1 (28), 113.1 (10), 111.1 (11), 99.2 (16), 75.2 (15). **MS (CI$^+$, methane)** *m/z* (%): 236.3 (28) $[M+C_2H_5$, $^{35}Cl]^+$ = $[C_{12}H_{11}ClNO_2]^+$, 208.3 (100) $[M+H]^+$ = $[C_{10}H_7ClNO_2]^+$. **EA**: calcd. for $C_{10}H_6ClNO_2$: C: 57.85%, H: 2.91%, N: 6.75%; found: C: 57.53%, H: 2.87%, N: 6.56%.

(*E*)-1-Methyl-3-(4-nitrobut-3-en-1-yn-1-yl)benzene [1e]

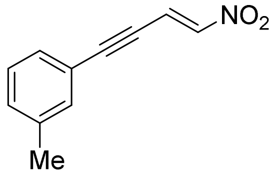

Compound **1e** was isolated after flash chromatography (SiO_2, *n*-pentane/Et_2O 5:1) as yellow solid (1.452 g, 81%). **Molecular formula:** $C_{11}H_9NO_2$. **Molecular mass:** 187.20 g mol^{-1}. $\mathbf{R_f}$ (*n*-pentane /EtOAc: 50:1) = 0.22. **Mp:** 30-35 °C. ^{1}**H NMR** (600 MHz, $CDCl_3$): δ = 2.36 (s, 3 H, CH_3), 7.25 (d, J = 7.8 Hz, 1 H, CH_{Ar}), 7.28 (t, J = 7.5 Hz, 1 H, CH_{Ar}), 7.38-7.31 (m, 4 H, CH_{Ar}, CH_{ol}) ppm. ^{13}**C NMR** (151 MHz, $CDCl_3$): δ = 21.3 (q), 81.8 (s), 105.7 (s), 121.1 (s), 121.3 (d), 128.7 (d), 129.6 (d), 131.5 (d), 132.9 (d), 138.7 (s), 145.6 (d) ppm. **IR** (ATR): $\tilde{\upsilon}$ = 3803, 3536, 3102, 3034, 2920, 2841, 2660, 2307, 2196, 1832, 1608, 1502, 1322, 1180, 1090, 1044, 932, 830, 777, 685, 532 cm^{-1}. **MS (EI$^+$, 70 eV)** *m/z* (%): 188.1 (25), 187.1 (95) $[M]^+$ = $[C_{11}H_9NO_2]^+$, 141.2 (18), 140.1 (100) $[M-HNO_2]^+$ = $[C_{11}H_8]^+$, 139.1 (64), 119.1 (37), 115.1 (31) $[M-C_2H_2NO_2]^+$ = $[C_9H_7]^+$, 91.1 (35) $[M-C_4H_2NO_2]^+$ = $[C_7H_7]^+$. **MS (CI$^+$, methane)** *m/z* (%): 216.2 (11) $[M+C_2H_5]^+$ = $[C_{13}H_{14}NO_2]^+$, 188.0 (100) $[M+H]^+$ = $[C_{11}H_{10}NO_2]^+$. **EA**: calcd. for $C_{11}H_9NO_2$: C: 70.58%, H: 4.85%, N: 7.48%; found: C: 70.32%, H: 5.08%, N: 7.48%.

(*E*)-1-Methoxy-3-(4-nitrobut-3-en-1-yn-1-yl)benzene [1f]

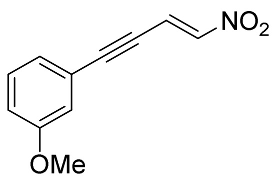

Compound **1f** was isolated after flash chromatography (SiO_2, *n*-pentane/Et_2O 5:1) as yellow solid (1.301 g, 71%). **Molecular formula:** $C_{11}H_9NO_3$. **Molecular mass:** 203.2 g mol^{-1}. **R$_f$** (*n*-pentane /EtOAc: 50:1) = 0.16. **Mp:** 41-46 °C. **^{1}H NMR** (600 MHz, $CDCl_3$): δ = 3.83 (s, 3 H, OCH_3), 6.98-7.01 (m, 1 H, CH_{Ar}), 7.02-7.04 (m, 1 H, CH_{Ar}), 7.10-7.13 (m, 1 H, CH_{Ar}), 7.30 (t, J = 8.0 Hz, 1 H, CH_{Ar}), 7.36 (s, 2 H, CH_{ol}) ppm. **^{13}C NMR** (151 MHz, $CDCl_3$): δ = 55.5 (q), 81.8 (s), 105.1 (s), 117.1 (d, 2 C), 121.1 (d), 122.2 (s), 125.0 (d), 129.9 (d), 145.8 (d), 159.6 (s) ppm. **IR** (ATR): $\tilde{\upsilon}$ = 3534, 3176, 3098, 2938, 2840, 2488, 2325, 2195, 1935, 1767, 1663, 1611, 1569, 1504, 1325, 1179, 1037, 932, 867, 774, 684 cm^{-1}. **MS (EI$^+$, 70 eV)** *m/z* (%): 204.0 (14), 203.0 (89) $[M]^+$ = $[C_{11}H_9NO_3]^+$, 157.0 (13), 156.0 (100) $[M{-}HNO_2]^+$ = $[C_{11}H_8O]^+$, 135.0 (20), 126.0 (23), 114.1 (14), 113.0 (18), 107.0 (12), 77.1 (11). **MS (CI$^+$, methane)** *m/z* (%): 232.4 (17) $[M+C_2H_5]^+$ = $[C_{13}H_{14}NO_3]^+$, 204.3 (69) $[M+H]^+$ = $[C_{11}H_{10}NO_3]^+$. **EA**: calcd. for $C_{11}H_9NO_3$: C: 65.02%, H: 4.46%, N: 6.89%; found: C: 64.78%, H: 4.34%, N: 6.47%.

(*E*)-5-(4-Nitrobut-3-en-1-yn-1-yl)benzo[*d*][1,3]dioxole [1g]

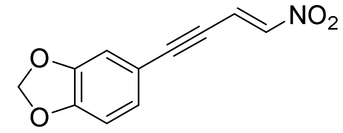

Compound **1g**was isolated after flash chromatography (SiO_2, *n*-pentane/Et_2O 5:1) as orange solid (979 mg, 66%). **Molecular formula:** $C_{11}H_7NO_4$. **Molecular mass:** 217.18 g mol^{-1}. **R$_f$** (*n*-pentane /EtOAc: 50:1) = 0.14. **Mp:** 105-110 °C. **^{1}H NMR** (600 MHz, $CDCl_3$): δ = 6.03 (d, J = 2.2 Hz, 2 H, CH_2), 6.82 (d, J = 8.1 Hz, 1 H, CH_{Ar}), 6.94 (d, J = 1.5 Hz, 1 H, CH_{Ar}), 7.08 (dd, J = 1.6 Hz, J = 8.1 Hz, 1 H, CH_{Ar}), 7.30-7.38 (m, 2 H, CH_{ol}) ppm. **^{13}C NMR** (151 MHz, $CDCl_3$): δ = 81.2 (s), 101.9 (t), 106.0 (s), 109.0 (d), 112.0 (d), 114.4 (s), 121.3 (d), 128.2 (d), 145.1 (d), 147.9 (s), 150.0 (s) ppm. **IR** (ATR): $\tilde{\upsilon}$ = 3789, 3521, 3211, 3110, 3026, 2922, 2848, 2788, 2638, 2579, 2499, 2399, 2299, 2187, 1988, 1942, 1872, 1738, 1653, 1597, 1490, 1438, 1325, 1231, 1150, 1108, 1028, 968, 940, 902, 868, 816, 758, 725, 703 cm^{-1}. **MS (EI$^+$, 70 eV)** *m/z* (%): 218.1 (14), 217.1 (100) $[M]^+$ = $[C_{11}H_7NO_4]^+$, 170.1 (59), 169.0 (22) $[M{-}HNO_2]^+$ = $[C_{11}H_6O_2]^+$, 149.0 (20), 121.1 (13), 113.1 (16). **MS (CI$^+$, methane)** *m/z* (%): 246.9 (4) $[M+C_2H_5]^+$ = $[C_{13}H_{12}NO_4]^+$, 218.3 (61) $[M+H]^+$ = $[C_{11}H_8NO_4]^+$. **EA**: calcd. for $C_{11}H_7NO_4$: C: 60.83%, H: 3.25%, N: 6.45%; found: C: 60.36%, H: 3.25%, N: 6.24%.

(*E*)-1-(4-Nitrobut-3-en-1-yn-1-yl)naphthalene [1h]

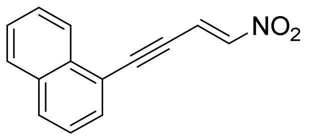

Compound YM-15 was isolated after flash chromatography (SiO_2, *n*-pentane/Et_2O 5:1) as white solid (729 mg, 81%). **Molecular formula:** $C_{14}H_9NO_2$. **Molecular mass:** 223.23 g mol^{-1}. **R$_f$** (*n*-pentane /EtOAc: 50:1) = 0.18. **Mp:** 72-75 °C. **^{1}H NMR** (600 MHz, $CDCl_3$): δ = 7.44-7.54 (m, 3 H, CH_{ol}, CH_{Ar}), 7.56-7.60 (m, 1 H, CH_{Ar}), 7.61-7.66 (m, 1 H, CH_{Ar}), 7.78 (dd, J = 0.9 Hz, J = 7.2 Hz, 1 H, CH_{Ar}), 7.90 (d, J = 8.2 Hz, 1 H, CH_{Ar}), 7.95 (d, J = 8.3 Hz, 1 H, CH_{Ar}), 8.24 (d, J = 8.4 Hz, 1 H, CH_{Ar}) ppm. **^{13}C NMR** (151 MHz, $CDCl_3$): δ = 86.7 (s), 103.6 (s), 118.9 (d), 121.1 (s) 125.4 (d), 125.7 (d), 127.1 (d), 127.8 (d), 128.8 (d), 131.4 (d), 132.4 (d), 133.2 (s, 2 C), 145.5 (d) ppm. **IR** (ATR): $\tilde{\nu}$ = 3855, 3750, 3517, 3106, 3025, 2944, 2838, 2664, 2391, 2300, 2177, 1904, 1737, 1607, 1505, 1320, 1110, 1017, 967, 924, 765 cm^{-1}. **MS (EI$^+$, 70 eV)** *m/z* (%): 224.1 (43), 223.1 (86) $[M]^+ = [C_{14}H_9NO_2]^+$, 206.1 (12), 177.1 (42), 176.0 (100) $[M-HNO_2]^+ = [C_{14}H_8]^+$, 175.0 (20), 170.1 (14), 165.1 (16), 164.1 (13), 162.1 (17), 155.1 (15) $[M-C_2H_2NO_2]^+ = [C_{12}H_7]^+$, 152.1 (23), 151.1 (22), 150.1 (33), 141.1 (19), 140.1 (12), 139.1 (17), 127.1 (40) $[M-C_4H_2NO_2]^+ = [C_{10}H_7]^+$. **MS (CI$^+$, methane)** *m/z* (%): 252.2 (20) $[M+C_2H_5]^+ = [C_{16}H_{14}NO_2]^+$, 224.1 (100) $[M+H]^+ = [C_{14}H_{10}NO_2]^+$. **HR-MS (ESI$^+$)** *m/z* (%): calcd. for $[M+Na]^+ = [C_{14}H_9NNaO_2]^+$, 246.0526; found: 246.0526.

(*E*)-2-(4-Nitrobut-3-en-1-yn-1-yl)naphthalene [1i]

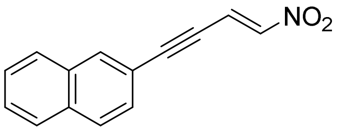

Compound **1i** was isolated after flash chromatography (SiO_2, *n*-pentane/Et_2O 5:1) as white solid (1.501 g, 63%). **Molecular formula:** $C_{14}H_9NO_2$. **Molecular mass:** 223.23 g mol^{-1}. **R$_f$** (*n*-pentane /EtOAc: 50:1) = 0.17. **Mp:** 95-98 °C. **^{1}H NMR** (600 MHz, $CDCl_3$): δ = 7.37-7.43 (m, 2 H, CH_{ol}), 7.50-7.58 (m, 3 H, CH_{Ar}), 7.80-7.89 (m, 3 H, CH_{Ar}), 8.07 (s, 1 H, CH_{Ar}) ppm. **^{13}C NMR** (151 MHz, $CDCl_3$): δ = 82.4 (s), 105.8 (s), 116.5 (d), 118.2 (d), 121.2 (s), 127.2 (d), 128.0 (d), 128.1 (d), 128.3 (d), 128.6 (d), 132.9 (s), 133.4 (d), 133.8 (s), 145.6 (d) ppm. **IR** (ATR): $\tilde{\nu}$ = 3528, 3107, 3034, 2940, 2837, 2648, 2484, 2400, 2300, 2190, 1993, 1934, 1723, 1657, 1609, 1514, 1337, 1264, 1149, 1084, 1032, 965, 935, 907, 873, 823, 748 cm^{-1}. **MS (EI$^+$, 70 eV)** *m/z* (%): 224.1 (17), 223.1 (83) $[M]^+ = [C_{14}H_9NO_2]^+$, 177.1 (15), 176.0 (100) $[M-HNO_2]^+ = [C_{14}H_8]^+$, 165.1 (11), 163.1 (11), 150.1 (16), 127.1 (40) $[M-C_4H_2NO_2]^+ = [C_{10}H_7]^+$. **MS (CI$^+$, methane)** *m/z* (%): 252.0 (9) $[M+C_2H_5]^+ = [C_{16}H_{14}NO_2]^+$, 224.1 (100) $[M+H]^+ = [C_{14}H_{10}NO_2]^+$. **EA**: calcd. for $C_{14}H_9NO_2$.: C: 75.33%, H: 4.06%, N: 6.27%; found: C: 75.42%, H: 4.25%, N: 6.07%.

(*E*)-2-(4-Nitrobut-3-en-1-yn-1-yl)furan [1j]

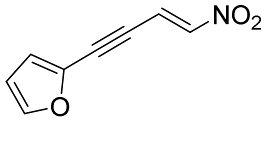

Compound **1j** was isolated after flash chromatography (SiO_2, *n*-pentane/Et_2O 5:1) as yellow solid (487 mg, 77%). **Molecular formula:** $C_8H_5NO_3$. **Molecular mass:** 163.13 g mol^{-1}. $\mathbf{R_f}$ (*n*-pentane /EtOAc: 50:1) = 0.16. **Mp:** 97-101 °C. ^{1}**H NMR** (600 MHz, $CDCl_3$): δ = 6.50 (dd, *J* = 1.8 Hz, *J* = 3.5 Hz, 1 H, CH_{Ar}), 6.87 (d, *J* = 3.5 Hz, 1 H, CH_{Ar}), 7.32-7.40 (m, 2 H, CH_{ol}), 7.53-7.54 (m, 1 H, CH_{Ar}) ppm. ^{13}**C NMR** (151 MHz, $CDCl_3$): δ = 87.5 (s), 94.9 (s), 112.1 (d), 120.0 (d, 2 C), 135.6 (s), 144.9 (d), 146.3 (d) ppm. **IR** (ATR): $\tilde{\upsilon}$ = 3804, 3745, 3647, 3522, 3333, 3190, 3109, 3037, 2958, 2843, 2892, 2761, 2672, 2614, 2539, 2423, 2301, 2178, 2133, 1990, 1938, 1781, 1725, 1663, 1618, 1557, 1505, 1460, 1377, 1326, 1209, 1075, 1044, 1001, 964, 934, 827, 760, 703 cm^{-1}. **MS (EI^+, 70 eV)** *m/z* (%): 163.0 (86) $[M]^+$ = $[C_8H_5NO_3]^+$, 116.0 (100) $[M–HNO_2]^+$ = $[C_8H_4O]^+$, 95.1 (21), 93.0 (18), 89.2 (13), 88.1 (33), 87.1 (40), 86.1 (32), 85.1 (11), 79.1 (15), 77.1 (12), 74.1 (16), 63.2 (71), 62.1 (13), 51.2 (25), 50.1 (16), 46.2 (20). **MS (CI^+, methane)** *m/z* (%): 164.1 (100) $[M+H]^+$ = $[C_8H_6NO_3]^+$. **EA**: calcd. for $C_8H_5NO_3$: C: 58.90%, H: 3.09%, N: 8.59%; found: C: 59.35%, H: 3.33%, N: 8.55%.

(*E*)-2-(4-Nitrobut-3-en-1-yn-1-yl)thiophene [1k]

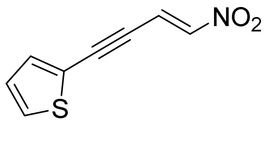

Compound **1k** was isolated after flash chromatography (SiO_2, *n*-pentane/Et_2O 5:1) as yellow solid (276 mg, 56%). **Molecular formula:** $C_8H_5NO_2S$. **Molecular mass:** 179.19 g mol^{-1}. $\mathbf{R_f}$ (*n*-pentane /EtOAc: 50:1) = 0.11. **Mp:** 89-93 °C. ^{1}**H NMR** (600 MHz, $CDCl_3$): δ = 7.08 (dd, *J* = 3.7 Hz, *J* = 5.1 Hz, 1 H, CH_{Ar}), 7.34 (d, *J* = 13.4 Hz, 1 H, CH_{ol}), 7.38 (d, *J* = 13.4 Hz, 1 H, CH_{ol}), 7.41 (dd, *J* = 1.1 Hz, *J* = 3.7 Hz, 1 H, CH_{Ar}), 7.48 (dd, *J* = 1.1 Hz, *J* = 5.1 Hz, 1 H, CH_{Ar}) ppm. ^{13}**C NMR** (151 MHz, $CDCl_3$): δ = 86.6 (s), 98.9 (s), 120.8 (d), 121.1 (s), 127.9 (d), 131.1 (d), 135.3 (d), 144.9 (d) ppm. **IR** (ATR): $\tilde{\upsilon}$ = 3595, 3510, 3163, 3104, 3026, 2929, 2822, 2748, 2405, 2241, 2174, 1992, 1948, 1836, 1755, 1717, 1639, 1607, 1515, 1491, 1412, 1323, 1253, 1190, 1140, 1078, 1047, 968, 928, 844, 721 cm^{-1}. **MS (EI^+, 70 eV)** *m/z* (%): 179.0 (86) $[M]^+$ = $[C_8H_5NO_2S]^+$, 133.1 (10), 132.0 (100) $[M–HNO_2]^+$ = $[C_8H_4S]^+$, 121.0 (11), 111.0 (59), 95.1 (18), 93.0 (14), 89.1 (40), 87.1 (12), 75.1 (10), 74.1 (14), 63.1 (20), 63.1 (20), 62.1 (10). **MS (CI^+, methane)** *m/z* (%): 208.5 (1) $[M+C_2H_5]^+$ = $[C_{10}H_{10}NO_2S]^+$, 208.5 (1) $[M+H]^+$ = $[C_8H_6NO_2S]^+$. **EA**: calcd. for $C_8H_5NO_2S_2$: C: 53.62%, H: 2.81%, N: 7.82%; found: C: 53.76%, H: 3.14%, N: 7.63%.

(*E*)-1-Nitrooct-1-en-3-yne [1p]

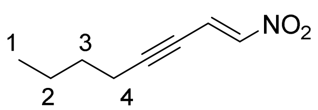

Compound **1p** was isolated after flash chromatography (SiO_2, *n*-pentane/Et_2O 5:1) as yellow solution (1.3580 g, 84%). **Molecular formula:** $C_{11}H_8NO_2$. **Molecular mass:** 153.18 g mol^{-1}. **R$_f$** (*n*-pentane /EtOAc: 50:1) = 0.32. **^{1}H NMR** (600 MHz, $CDCl_3$): δ = 0.93 (t, J = 7.4 Hz, 3 H, 1-CH_3), 1.39-1.47 (m, 2 H, 2-CH_2), 1.53-1.60 (m, 2 H, 3-CH_2), 2.44 (dt, J = 2.4 Hz, J = 7.1 Hz, 2 H, 4-CH_2), 7.13 (dt, J = 2.3 Hz, J = 13.4 Hz, 1 H, CH_{ol}), 7.21 (d, J = 13.4 Hz, 1 H, CH_{ol}) ppm. **^{13}C NMR** (151 MHz, $CDCl_3$): δ = 13.6 (q), 19.3 (t), 22.1 (t), 30.1 (t), 73.8 (t), 108.7 (t), 122.1 (t), 145.7 (t) ppm. **IR** (ATR): $\tilde{\nu}$ = 3836, 3112, 3030, 2934, 2665, 2342, 2216, 2111, 1934, 1741, 1667, 1618, 1523, 1461, 1340, 1233, 1168, 1086, 1042, 939, 836, 720, 699 cm^{-1}. **MS (CI$^+$, methane)** *m/z* (%): 182.0 (18) $[M+C_2H_5]^+$ = $[C_{13}H_{13}NO_2]^+$, 153.9 (69) $[M+H]^+$ = $[C_{11}H_9NO_2]^+$. **EA**: calcd. for $C_{11}H_8NO_2$: C: 62.73%, H: 7.24%, N: 9.14%; found: C: 962.38%, H: 6.87%, N: 9.69%.

(*E*)-(4-Nitrobut-3-en-1-yn-1-yl)cyclopentane [1q]

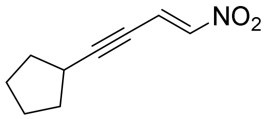

Compound **1q** was isolated after flash chromatography (SiO_2, *n*-pentane/Et_2O 5:1) as yellow solution (743 mg, 81%). **Molecular formula:** $C_9H_{11}NO_2$. **Molecular mass:** 165.19 g mol^{-1}. **R$_f$** (*n*-pentane/EtOAc: 50:1) = 0.30. **^{1}H NMR** (600 MHz, $CDCl_3$): δ = 1.56-1.71 (m, 4 H, CH_2), 1.71-1.81 (m, 2 H, CH_2), 1.94-2.05 (m, 2 H, CH_2), 2.84 (dp, J = 2.2 Hz, J = 7.6 Hz, 1 H, CH), 7.14 (dd, J = 2.3 Hz, J = 13.4 Hz, 1 H, CH_{ol}), 7.20 (d, J = 13.6 Hz, 1 H, CH_{ol}) ppm. **^{13}C NMR** (151 MHz, $CDCl_3$): δ = 25.3 (t, 2 C), 31.4 (d), 33.6 (t, 2 C), 73.4 (s), 112.9 (s), 122.3 (d), 145.5 (d) ppm. **IR** (ATR): $\tilde{\nu}$ = 3112, 3033, 2958, 2871, 2310, 2209, 2986, 1992, 1940, 1616, 1521, 1452, 1338, 1237, 1180, 1126, 966, 939, 835, 735 cm^{-1}. **MS (EI$^+$, 70 eV)** *m/z* (%): 166.1 (28), 165.1 (45) $[M]^+$ = $[C_9H_{11}NO_2]^+$, 120.1 (14), 119.2 (19) $[M-HNO_2]^+$ = $[C_9H_{10}]^+$, 117.1 (24), 115.1 (15), 109.1 (22), 91.1 (43), 89.1 (29), 81.2 (23), 79.2 (27), 78.2 (12), 77.2 (34), 76.2 (33), 75.2 (24), 74.1 (22), 65.2 (30), 63.2 (100), 62.1 (34), 53.2 (20), 52.3 (14), 51.2 (51), 50.2 (37), 46.2 (59). **MS (CI$^+$, methane)** *m/z* (%): 333.1 (100) $[2M+H]^+$ = $[C_{18}H_{23}N_2O_4]^+$. **EA**: calcd. for $C_9H_{11}NO_2$: C: 65.44%, H: 6.71%, N: 8.48%; found: C: 65.28%, H: 6.55 %, N: 8.89%.

Exceptions from general procedures:

Corey-Fuchs-homologisation of 2-bromobenzaldehyde[3]:

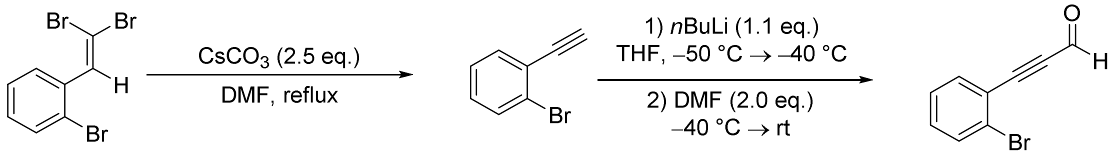

A suspension of *gem*-dibromoolefine (7.36 g, 21.6 mmol) and Cs_2CO_3 (17.6 g, 54.0 mmol) in DMSO (216 mL, 0.1 M) was stirred at 115 °C for 18 hours. Then, a saturated solution of NaCl was added, the product was extracted with diethyl ether and dried over $MgSO_4$. The crude product was subjected to flash chromatography on silica (*n*-pentane: Et_2O 95:5) to yield 4-bromophenylacetylene (3.91 g, 84%). Under an atmosphere of argon, *n*-BuLi (6.9mL, 11.0 mmol 1.6 M in *n*-hexane) was added over a period of 30 minutes *via* syringe pump to a solution of 2-bromophenylacetylene (1.80 g, 10.0 mmol) in abs. THF (25 mL) at –50 °C, and the mixture was stirred at –40 °C for 15 minutes. After addition of DMF (1.5 mL, 20.4 mmol), the mixture was allowed to warm to room temperature and stirred for one hour. The mixture was added to a stirring solution of NaH_2PO_4 (aq.)/MTBE (1:1). After five minutes, the layers were separated and the aqueous layer was extracted with MTBE. The combined organic layers were dried over $MgSO_4$, the solvent was removed under reduced pressure, and the crude product was subjected to flash chromatography on silica (*n*-pentane/Et_2O 95:5) to yield the desired aldehyde (1.01 g, 48%).

General procedure for the sequential catalysis:

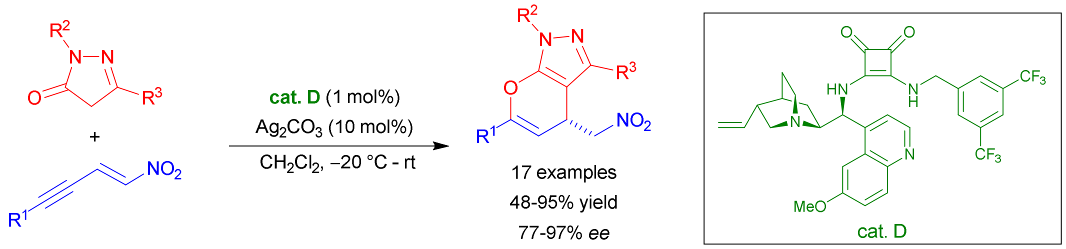

A suspension of a nitroolefin (0.55 mmol, 1.1 eq.), a pyrazolinone (0.5 mmol, 1.0 eq.), catalyst **D** (3.2 mg, 1 mol%) and Ag_2CO_3 (13.8 mg, 0.1 eq.) in CH_2Cl_2 was stirred at –20 °C for 12 hours, until TLC indicated full conversion of the starting material. The reaction was allowed to warm to room temperature, and stirred for further two hours. The crude product was subjected flash chromatography on silica (*n*-pentane/Et_2O) to afford the afforded products.

[3] Zhao, M.; Kuang, C.; Yang, Q.; Cheng, X. *Tetrahedron Letters* **2011**, *52*, 992-994.

(*R*)-3-methyl-4-(1-nitro-4-phenylbut-3-yn-2-yl)-1-phenyl-1H-pyrazol-5-ol [3a]

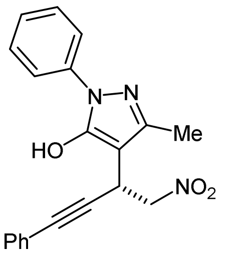

Compound **3a** was isolated after flash chromatography (SiO_2, *n*-pentane/EtOAc 3:1 to 1:1) as brown solid (162 mg, 93%). **Molecular formula:** $C_{20}H_{17}N_3O_3$. **Molecular mass:** 347.37 g mol^{-1}. $\mathbf{R_f}$(*n*-pentane/EtOAc 3:1) = 0.10. ^{1}**H NMR** (600 MHz, $CDCl_3$, main tautomer): δ = 2.19 (s, 3 H, C*H₃*), 4.42-4.60 (m, 3 H, C*H*, C*H₂*), 7.07 (t, *J* = 7.4 Hz, 1 H, CH_{Ar}), 7.14-7.21 (m, 2 H, CH_{Ar}), 7.22-7.28 (m, 3 H, CH_{Ar}), 7.30-7.36 (m, 4 H, CH_{Ar}) ppm. ^{13}**C NMR** (150 MHz, $CDCl_3$, all signals, no assignment): δ = 11.4, 15.4, 26.3, 30.1, 30.4, 52.3, 66.0, 73.4, 76.8, 80.5, 84.1, 84.7, 86.4, 99.2, 119.3, 121.1, 122.4, 125.7, 126.5, 128.5, 128.7, 129.3, 131.7, 135.7, 146.0, 157.1, 170.6 ppm. **IR** (ATR): $\tilde{\nu}$ = 3066, 2916, 2702, 2319, 2102, 1893, 1804, 1723, 1552, 1495, 1410, 1373, 1308, 1191, 1123, 1070, 1017, 911, 834, 749, 686, 588 cm^{-1}. **MS (EI$^+$, 70 eV)** *m/z* (%): 346.7 (24) $[M]^+$ = $[C_{20}H_{17}N_3O_3]^+$, 300.1 (17) $[M-HNO_2]^+$ = $[C_{20}H_{16}N_2O]^+$, 298.3 (15), 286.2 (26), 285.2 (24), 284.1 (19), 173.1 (41), 171.6 (100), 128.3 (17), 127.6 (54), 126.2 (37), 114.3 (12), 104.9 (51), 77.6 (23) $[C_6H_5]^+$, 77.0 (32). **MS (CI$^+$, methane)** *m/z* (%): 376.1 (11) $[M+C_2H_5]^+$ = $[C_{22}H_{22}N_3O_3]^+$, 348.0 (45) $[M+H]^+$ = $[C_{20}H_{18}N_3O_3]^+$. **EA:** calcd. for $C_{20}H_{17}N_3O_3$: C 69.15%, H 4.93%, N 12.10%; found: C 69.57%, H 4.96%, N 11.80%.

(*R*)-3-Methyl-4-(1-nitro-4-phenylbut-3-yn-2-yl)-1-phenyl-1*H*-pyrazol-5-yl acetate [3a']

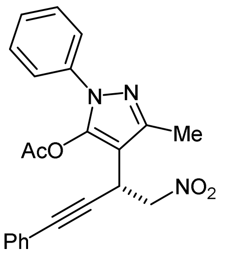

Molecular formula: $C_{22}H_{19}N_3O_4$. **Molecular mass:** 389.41 g mol^{-1}. $\mathbf{R_f}$(*n*-pentane/EtOAc 5:1) = 0.39. **HPLC:** AD, 9/1 *n*-heptane/*i*PrOH, 0.7 ml/min, λ = 254 nm, τ_{minor} = 10.4 min, τ_{major} = 11.6 min. ^{1}**H NMR** (600 MHz, $CDCl_3$): δ = 2.18 (s, 3 H, C*H₃*), 2.43 (s, 3 H, C*H₃*), 4.61 (dd, *J* = 6.2 Hz, *J* = 11.8 Hz, 1 H, C*H*), 4.66 (dd, *J* = 6.2 Hz, *J* = 8.7 Hz, 1 H, C*H₂*), 4.76 (dd, *J* = 8.8 Hz, *J* = 11.8 Hz, 1 H, C*H₂*), 7.28-7.38 (m, 4 H, CH_{Ar}), 7.39-7.43 (m, 2 H, CH_{Ar}), 7.45 (d, *J* = 7.4 Hz, 2 H, CH_{Ar}), 7.48 (d, *J* = 7.7 Hz, 2H, CH_{Ar}) ppm. ^{13}**C NMR** (150 MHz, $CDCl_3$): δ = 13.3 (q), 20.5 (q), 26.8 (d), 77.7 (t), 83.7 (s), 84.5 (s), 103.1 (s), 122.2 (s), 123.2 (d, 2 C), 127.9 (d), 128.5 (d, 2 C), 128.8 (d), 129.4 (d, 2 C), 131.8 (d, 2 C), 137.6 (s), 142.0 (s), 147.2 (s), 167.5 (s) ppm. **IR** (ATR): $\tilde{\nu}$ = 3291, 2063, 2925, 2338, 1964, 1887, 1790, 1728, 1661, 1594, 1553, 1501, 1435, 1374, 1321, 1244, 1165, 1053, 1006, 960, 914, 882,

814, 756, 690 cm^{-1}. **MS (EI$^+$, 70 eV)** *m/z* (%): 389.7 (3) $[M]^+ = [C_{22}H_{19}N_3O_4]^+$ 342.4 (20), 340.9 (32), 328.5 (28), 301.4 (16), 300.4 (39), 299.5 (30), 298.3 (46), 288.2 (13), 287.2 (60), 286.2 (100), 285.3 (30), 284.0 (26), 257.0 (10), 256.0 (12), 214.4 (15), 198.8 (15), 197.7 (17), 188.9 (11), 188.0 (13), 186.7 (15), 180.1 (11), 179.2 (10), 164.7 (21), 163.1 (19), 153.2 (13), 152.1 (25), 151.0 (29), 149.9 (11), 139.3 (12), 128.4 (10), 126.4 (13), 118.4 (14), 117.3 (26), 105.4 (24), 104.6 (71), 77.7 (23), 76.9 (22). **MS (CI$^+$, methane)** *m/z* (%): 418.2 (3) $[M+C_2H_5]^+ = [C_{24}H_{24}N_3O_4]^+$, 390.1 (49) $[M+H]^+ = [C_{19}H_{20}N_3O_4]^+$.]$^+$. **HR-MS (ESI$^+$)** *m/z* (%): calcd. for $[M+H]^+ = [C_{222}H_{20}N_3O_4]^+$: 390.1448; found: 390.1448.

(*R*)-3-Methyl-4-(nitromethyl)-1,6-diphenyl-1,4-dihydropyrano[2,3-*c*]pyrazole [4a]

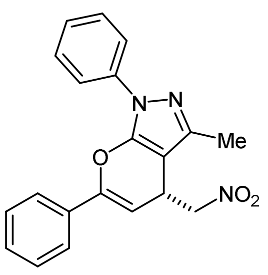

Compound **4a** was isolated after flash chromatography (SiO_2, *n*-pentane/EtOAc 3:1) as white solid (153 mg, 88%). **Molecular formula:** $C_{20}H_{17}N_3O_3$. **Molecular mass:** 347.37 mol^{-1}. **R**$_f$(*n*-pentane/EtOAc 5:1) = 0.38. **Mp:** 163-165 °C. **HPLC:** AD, 97/3 *n*-heptane/*i*PrOH, 0.7 ml/min, $\lambda = 254$ nm, $\tau_{minor} = 13.8$ min, $\tau_{major} = 15.3$ min. **OR:** $[\alpha]_D^{20} = -124.7$ ($c = 0.6$, $CHCl_3$, 99% ee). **^{1}H NMR** (600 MHz, $CDCl_3$): $\delta = 2.33$ (s, 3 H, CH_3), 4.47 (ddd, $J = 4.0$ Hz, $J = 4.4$ Hz, $J = 8.5$ Hz, 1 H, C*H*), 4.53 (dd, $J = 8.5$ Hz, $J = 11.7$ Hz, 1 H, CH_2), 4.68 (dd, $J = 4.4$ Hz, $J = 11.7$ Hz, 1 H, CH_2), 5.61 (d, $J = 4.0$ Hz, 1 H, CH_{ol}), 7.28-7.34 (m, 1 H, CH_{Ar}), 7.37-7.45 (m, 3 H, CH_{Ar}), 7.45-7.52 (m, 2 H, CH_{Ar}), 7.55-7.66 (m, 2 H, CH_{Ar}), 7.74-7.82 (m, 2 H, CH_{Ar}) ppm. **^{13}C NMR** (151 MHz, $CDCl_3$): $\delta = 13.4$ (q), 31.4 (d), 80.3 (t), 93.6 (s), 97.6 (d), 121.1 (d, 2 C), 125.3 (d, 2 C), 126.6 (s), 128.8 (d, 2 C), 129.4 (d, 2 C), 129.7 (d), 132.6 (d), 138.1 (s), 145.6 (s), 147.6 (s), 150.9 (s) ppm. **IR** (ATR): $\tilde{\nu} = 3055$, 2918, 2845, 2655, 2317, 2195, 2069, 2037, 1980, 1952, 1740, 1663, 1598, 1539, 1510, 1440, 1400, 1378, 1322, 1280, 1280, 1246, 1191, 1119, 1076, 1030, 996, 923, 884, 847, 801, 762, 695, 661 cm^{-1}. **MS (EI$^+$, 70 eV)** *m/z* (%): 301.3 (26) $[M-NO_2]^+ = [C_{20}H_{17}N_2O]^+$, 300.3 (92) $[M-HNO_2]^+ = [C_{20}H_{16}N_2O]^+$, 288.2 (21), 287.3 (100) $[M-CH_2NO_2]^+ = [C_{19}H_{15}N_2O]^+$, 188.1 (12), 77.1 $[C_6H_5]^+$. **MS (CI$^+$, methane)** *m/z* (%): 376.5 (13) $[M+C_2H_5]^+ = [C_{22}H_{22}N_3O_3]^+$, 348.4 (56) $[M+H]^+ = [C_{20}H_{18}N_3O_3]^+$. **EA:** calcd. for $C_{20}H_{17}N_3O_3$: C 69.15%, H 4.93%, N 12.10%; found: C 69.07%, H 5.05%, N 11.68%.

(*R*)-6-(2-Bromophenyl)-3-methyl-4-(nitromethyl)-1-phenyl-1,4-dihydropyrano[2,3-*c*]pyrazole [4b]

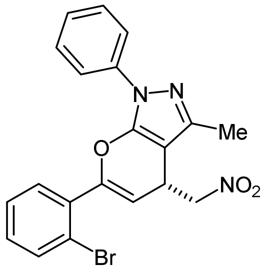

Compound **4b** was isolated after flash chromatography (SiO_2, *n*-pentane/EtOAc 3:1 to 1:1) as off-white solid (164 mg, 77%). **Molecular formula:** $C_{20}H_{16}BrN_3O_3$. **Molecular mass:** 426.27 mol^{-1}. $\mathbf{R_f}$(*n*-pentane/EtOAc 5:1) = 0.34. **Mp:** 140-143 °C. **HPLC:** OD, 8/2 *n*-heptane/EtOH, 1.0 ml/min, λ = 254 nm, τ_{minor} = 7.0 min, τ_{major} = 8.0 min. **OR:** $[\alpha]_D^{20}$ = −141.2 (*c* = 0.6, $CHCl_3$, 95% ee). **^{1}H NMR** (400 MHz, $CDCl_3$): δ = 2.34 (m, 3 H, CH_3), 4.48 (ddd, *J* = 3.9 Hz, *J* = 4.6 Hz, *J* = 8.1 Hz, 1 H, C*H*), 4.59 (dd, *J* = 8.1 Hz, *J* = 11.8 Hz, 1 H, CH_2), 4.69 (dd, *J* = 4.6 Hz, *J* = 11.8 Hz, 1 H, CH_2), 5.33 (d, *J* = 3.9 Hz, 1 H, CH_{ol}), 7.21-7.26 (m, 1 H, CH_{Ar}), 7.27-7.42 (m, 4 H, CH_{Ar}), 7.44 (dd, *J* = 1.8 Hz, *J* =7.5 Hz, 1 H CH_{Ar}), 7.65 (dd, *J* = 1.2 Hz, *J* = 7.9 Hz, 1 H, CH_{Ar}), 7.72-7.79 (m, 2 H, CH_{Ar}) ppm. **^{13}C NMR** (100 MHz, $CDCl_3$): δ = 13.4 (q), 31.6 (d), 80.2 (t), 93.4 (s), 102.8 (d), 120.9 (d, 2 C), 122.7 (s), 126.5 (d), 127.6 (d), 129.3 (d, 2 C), 131.2 (d), 131.4 (d), 133.6 (d), 134.9 (s), 138.0 (s), 145.4 (s), 147.4 (s), 150.9 (s) ppm. **IR** (ATR): $\tilde{\nu}$ = 3829, 2675, 3342, 3116, 2916, 2706, 2495, 2288, 2242, 2064, 1993, 1903, 1741, 1678, 1595, 1544, 1514, 1436, 1384, 1331, 1280, 1238, 1207, 1122, 1084, 1030, 991, 946, 904, 823, 756, 689, 663 cm^{-1}. **MS (EI$^+$, 70 eV)** *m/z* (%): 381.0 (20), 379.9 (100) [M−HNO_2, $^{81}Br]^+$ = $[C_{20}H_{15}BrN_2O]^+$, 379.0 (12), 377.9 (87) [M−HNO_2, $^{79}Br]^+$ = $[C_{20}H_{15}BrN_2O]^+$, 368.0 (15), 366.9 (79) [M−CH_2NO_2, $^{81}Br]^+$ = $[C_{19}H_{14}BrN_2O]^+$, 366.0 (14), 364.9 (71) [M−CH_2NO_2, $^{79}Br]^+$ = $[C_{19}H_{14}BrN_2O]^+$, 117.9 (12), 76.9 (26) $[C_6H_5]^+$. **MS (CI$^+$, methane)** *m/z* (%): 456.0 (14) [M+C_2H_5, $^{81}Br]^+$ = $[C_{22}H_{21}BrN_3O_3]^+$, 454.0 (14) [M+C_2H_5, $^{79}Br]^+$ = $[C_{22}H_{21}BrN_3O_3]^+$, 427.9 (52) [M+H, $^{81}Br]^+$ = $[C_{20}H_{17}BrN_3O_3]^+$, 425.9 (54) [M+H, $^{79}Br]^+$ = $[C_{20}H_{17}BrN_3O_3]^+$. **HR-MS (ESI$^+$)** *m/z* (%): calcd. for [M+H, $^{79}Br]^+$ = $[C_{20}H_{17}BrN_3O_3]^+$: 426.0448; found: 426.0448.

(*R*)-3-Methyl-4-(nitromethyl)-1-phenyl-6-(4-(trifluoromethyl)phenyl)-1,4-dihydropyrano[2,3-*c*]pyrazole [4c]

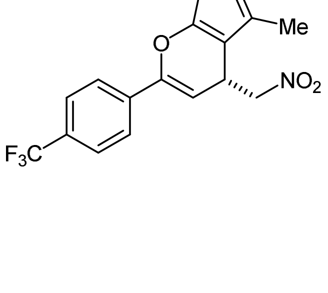

Compound **4c** was isolated after flash chromatography (SiO_2, *n*-pentane/EtOAc 3:1 to 1:1) as off-white solid (185 mg, 89%). **Molecular formula:** $C_{21}H_{16}F_3N_3O_3$. **Molecular mass:** 415.11 g mol^{-1}. $\mathbf{R_f}$(*n*-pentane/EtOAc 5:1) = 0.18. **Mp:** 180-182 °C. **HPLC:** AD, 9/1 *n*-heptane/*i*PrOH, 1.0 ml/min, λ = 230 nm, τ_{minor} = 6.8 min, τ_{major} = 8.1 min. **OR:** $[\alpha]_D^{20}$ = −109.6 (*c* = 0.6, $CHCl_3$, 99% ee). **^{1}H NMR** (400 MHz, $CDCl_3$): δ = 2.34 (s, 3 H, C*H$_3$*), 4.49 (ddd, *J* = 4.0 Hz, *J* = 4.2 Hz, *J* = 8.4 Hz, 1 H, C*H*), 4.56 (dd, *J* = 8.4 Hz, *J* = 11.7 Hz, 1 H, C*H$_2$*), 4.70 (dd, *J* = 4.2 Hz, *J* = 11.7 Hz, 1 H, C*H$_2$*), 5.72 (d, *J* = 4.0 Hz, 1 H, CH_{ol}), 7.32 (dt, *J* = 1.1 Hz, *J* = 7.4 Hz, 1 H, CH_{Ar}), 7.45-7.52 (m, 2 H, CH_{Ar}), 7.65-7.70 (m, 2 H, CH_{Ar}), 7.71-7.70 (m, 4 H, CH_{Ar}) ppm. **^{13}C{^{19}F} NMR** (100 MHz, $CDCl_3$): δ = 13.4 (q), 31.5 (d), 80.0 (t), 93.3 (s), 99.8 (d), 121.1 (d, 2 C), 123.9 (s), 125.6 (d, 2 C), 129.9 (d, 2 C), 126.8 (d), 129.5 (d, 2 C), 131.6 (s), 136.0 (s), 138.0 (s), 145.6 (s), 147.2 (s), 149.7 (s) ppm. **^{19}F{^{1}H}** (376 MHz, $CDCl_3$): δ = −62.9 (s) ppm. **IR** (ATR): $\tilde{\nu}$ = 3843, 3449, 2937, 2655, 2458, 2289, 2069, 1929, 1738, 1607, 1519, 1406, 1316, 1110, 1001, 835, 743 cm^{-1}. **MS (EI$^+$, 70 eV)** *m/z* (%): 369.0 (13) $[M-NO_2]^+$ = $[C_{21}H_{16}F_3N_2O]^+$, 368.0 (52) $[M-HNO_2]^+$ = $[C_{21}H_{15}F_3N_2O]^+$, 356.0 (13), 355.0 (62) $[M-CH_2NO_2]^+$ = $[C_{20}H_{14}F_3N_2O]^+$, 182.9 (15), 172.9 (11), 144.8 (18), 117.9 (24), 76.9 (100) $[C_6H_5]^+$, 51.0 (19), . **MS (CI$^+$, methane)** *m/z* (%): 444.3 (13) $[M+C_2H_5]^+$ = $[C_{23}H_{21}F_3N_3O_3]^+$, 416.3 (49) $[M+H]^+$ = $[C_{21}H_{17}F_3N_3O_3]^+$. **EA:** calcd. for $C_{21}H_{16}F_3N_3O_3$: C 60.72%, H 3.88%, N 10.12%; found: C 61.06%, H 4.27%, N 9.98%.

(*R*)-6-(2-Chlorophenyl)-3-methyl-4-(nitromethyl)-1-phenyl-1,4-dihydropyrano[2,3-*c*]pyrazole [4d]

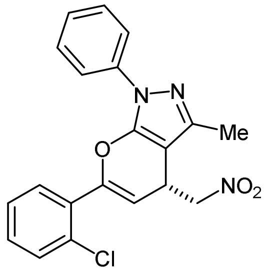

Compound **4d** was isolated after flash chromatography (SiO_2, *n*-pentane/EtOAc 3:1 to 1:1) as off-white solid (142 mg, 74%). **Molecular formula:** $C_{20}H_{16}ClN_3O_3$. **Molecular mass:** 381.82 mol^{-1}. $\mathbf{R_f}$(*n*-pentane/EtOAc 5:1) = 0.43. **Mp:** 130-134 °C. **HPLC:** AD, 97/3 *n*-heptane/*i*PrOH, 1.0 ml/min, λ = 254 nm, τ_{minor} = 11.4 min, τ_{major} = 13.5 min. **OR:** $[\alpha]_D^{20}$ = −154.7 (*c* = 0.7, $CHCl_3$, 99% ee). **^{1}H NMR** (400 MHz, $CDCl_3$): δ = 2.33 (m, 3 H, C*H$_3$*), 4.49 (ddd, *J* = 3.9 Hz, *J* = 4.6 Hz, *J* = 8.1 Hz, 1 H, C*H*), 4.59 (dd, *J* = 8.1 Hz, *J* = 11.8 Hz, 1 H, C*H$_2$*), 4.69 (dd, *J* = 4.6 Hz, *J* = 11.8 Hz, 1 H, C*H$_2$*), 5.38 (d, *J* = 3.9 Hz, 1 H, CH_{ol}), 7.23 (dt, *J* = 1.1 Hz, *J* = 7.5 Hz, 1 H, CH_{Ar}), 7.28-7.43 (m, 4 H, CH_{Ar}), 7.44-7.49 (m, 2 H, CH_{Ar}), 7.71-7.77 (m, 2 H, CH_{Ar}) ppm. **^{13}C NMR** (100 MHz, $CDCl_3$): δ = 13.4 (q), 31.6 (d), 80.2 (t) 93.4 (s), 103.0 (d), 120.9 (d, 2 C), 126.5 (d), 127.1 (d), 129.3 (d, 2 C), 130.4 (d), 131.0 (d), 131.1 (d), 132.7 (s), 133.3 (s), 138.0 (s), 145.5 (s), 147.5 (s), 149.6 (s) ppm. **IR** (ATR): $\tilde{\nu}$ = 3815, 3345, 3063, 2918, 2711, 2465, 2168, 2115, 1942, 1869, 1737, 1679, 1599, 1529, 1435, 1386, 1321, 1279, 1232,

1188, 1122, 1090, 1031, 997, 939, 898, 852, 800, 752, 689, 659 cm^{-1}. **MS (EI$^+$, 70 eV)** *m/z* (%): 336.1 (15) [M−HNO_2, ^{37}Cl]$^+$ = [$C_{20}H_{15}ClN_2O$]$^+$, 334.1 (36) [M−HNO_2, ^{35}Cl]$^+$ = [$C_{20}H_{15}ClN_2O$]$^+$, 323.1 (13) [M−CH_2NO_2, ^{37}Cl]$^+$ = [$C_{19}H_{14}ClN_2O$]$^+$, 321.1 (39) [M−CH_2NO_2, ^{35}Cl]$^+$ = [$C_{19}H_{14}ClN_2O$]$^+$, 118.0 (23), 76.9 (21) [C_6H_5]$^+$, 65.0 (11), 51.1 (21). **MS (CI$^+$, methane)** *m/z* (%): 411.7 (9) [M+C_2H_5, ^{37}Cl]$^+$ = [$C_{22}H_{21}ClN_3O_3$]$^+$, 410.0 (20) [M+C_2H_5, ^{35}Cl]$^+$ = [$C_{22}H_{21}ClN_3O_3$]$^+$, 384.0 (31) [M+H, ^{37}Cl]$^+$ = [$C_{20}H_{17}ClN_3O_3$]$^+$, 382.2 (94) [M+H, ^{35}Cl]$^+$ = [$C_{20}H_{17}ClN_3O_3$]$^+$. **HR-MS (ESI$^+$)** *m/z* (%): calcd. for [M+H, ^{35}Cl]$^+$ = [$C_{20}H_{17}ClN_3O_3$]$^+$, 382.0953; found: 382.0953.

(*R*)-3-Methyl-4-(nitromethyl)-1-phenyl-6-(m-tolyl)-1,4-dihydropyrano[2,3-*c*]pyrazole [4e]

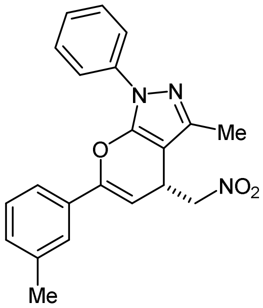

Compound **4e**was isolated after flash chromatography (SiO_2, *n*-pentane/EtOAc 3:1 to 1:1) as off-white solid (160 mg, 88%). **Molecular formula:** $C_{21}H_{19}N_3O_3$. **Molecular mass:** 361.42 g mol^{-1}. **R$_f$**(*n*-pentane/EtOAc 5:1) = 0.38. **Mp:** 124-127 °C. **HPLC:** OJ, 7/3 *n*-heptane/EtOH, 1.0 ml/min, λ = 254 nm, τ_{minor} = 18.3 min, τ_{major} = 8.0 min. **OR:** $[\alpha]_D^{20}$ = −110.7 (*c* = 0.6, $CHCl_3$, 99% ee). **^{1}H NMR** (400 MHz, $CDCl_3$): δ = 2.33 (s, 3 H, CH_3), 2.39 (s, 3 H, CH_3), 4.46 (ddd, *J* = 3.9 Hz, *J* = 4.2 Hz, *J* = 8.5 Hz, 1 H, C*H*), 4.52 (dd, *J* = 8.5 Hz, *J* = 11.4 Hz, 1 H, CH_2), 4.68 (dd, *J* = 4.2 Hz, *J* = 11.4 Hz, 1 H, CH_2), 5.59 (d, *J* = 3.9 Hz, 1 H, CH_{ol}), 7.19-7.24 (m, 1 H, CH_{Ar}), 7.27-7.35 (m, 2 H, CH_{Ar}), 7.39-7.45 (m, 2 H, CH_{Ar}), 7.45-7.52 (m, 2 H, CH_{Ar}), 7.74-7.82 (m, 2 H, CH_{Ar}) ppm. **^{13}C NMR** (100 MHz, $CDCl_3$): δ = 13.4 (q), 21.7 (q), 31.4 (d), 80.3 (t), 93.6 (s), 97.4 (d), 121.0 (d, 2 C), 122.4 (d), 126.0 (d), 126.6 (d), 128.7 (d), 129.4 (d, 2 C), 130.5 (d), 132.6 (d), 138.1 (s), 138.5 (s), 145.6 (s), 147.6 (s), 151.0 (s) ppm. **IR** (ATR): $\tilde{\nu}$ = 3829, 3320, 3076, 2916, 2727, 2467, 2312, 2154, 2064, 1985, 1933, 1794, 1724, 1659, 1600, 1518, 1439, 1378, 1318, 1277, 1230, 1180, 1125, 1078, 1033, 989, 932, 900, 865, 792, 751, 716, 688 cm^{-1}. **MS (EI$^+$, 70 eV)** *m/z* (%): 361.0 (3) [M]$^+$ = [$C_{21}H_{19}N_3O_3$]$^+$, 315.1 (28) [M−NO_2]$^+$ = [$C_{21}H_{19}N_2O$]$^+$, 314.0 (93) [M−HNO_2]$^+$ = [$C_{21}H_{18}N_2O$]$^+$, 302.1 (30), 301.0 (100) [M−CH_2NO_2]$^+$ = [$C_{20}H_{17}N_2O$]$^+$. **MS (CI$^+$, methane)** *m/z* (%): 362.1 (35) [M+H]$^+$ = [$C_{21}H_{20}N_3O_3$]$^+$. **HR-MS (ESI$^+$)** *m/z* (%): calcd. for [M+H]$^+$ = [$C_{21}H_{20}N_3O_3$]$^+$: 362.1499; found: 362.1498.

(*R*)-6-(3-methoxyphenyl)-3-methyl-4-(nitromethyl)-1-phenyl-1,4-dihydropyrano[2,3-*c*]pyrazole [4f]

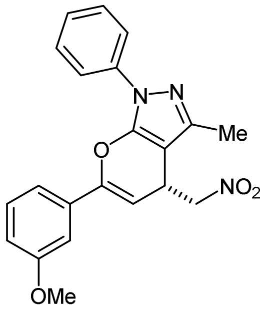

Compound **4f** was isolated after flash chromatography (SiO_2, *n*-pentane/EtOAc 3:1 to 1:1) as off-white solid (173 mg, 93%). **Molecular formula:** $C_{21}H_{19}N_3O_4$. **Molecular mass:** 377.40 mol^{-1}. $\mathbf{R_f}$(*n*-pentane/EtOAc 5:1) = 0.18. **Mp:** 115-118 °C. **HPLC:** OD, 9/1 *n*-heptane/*i*PrOH, 1.0 ml/min, λ = 254 nm, τ_{minor} = 15.2 min, τ_{major} = 17.9 min. **OR:** $[\alpha]_D^{20}$ = −111.5 (*c* = 0.6, $CHCl_3$, 99% ee). **^{1}H NMR** (400MHz, $CDCl_3$): δ = 2.33 (s, 3 H, C*H₃*), 3.84 (s, 3 H, OCH_3), 4.46 (ddd, *J* = 4.0 Hz, *J* = 4.3 Hz, *J* = 8.4 Hz, 1 H, C*H*), 4.52 (dd, *J* = 8.4 Hz, *J* = 11.5 Hz, 1 H, CH_2), 4.68 (dd, *J* = 4.3 Hz, *J* = 11.5 Hz, 1 H, CH_2), 5.61 (d, *J* = 4.0 Hz, 1 H, CH_{ol}), 6.95 46 (ddd, *J* = 0.8 Hz, *J* = 2.5 Hz, *J* = 8.2 Hz, 1 H, CH_{Ar}), 7.13-7.18 (m, 1 H, CH_{Ar}), 7.19-7.24 (m, 1 H, CH_{Ar}), 7.27-7.36 (m, 2 H, CH_{Ar}), 7.44-7.51 (m, 2 H, CH_{Ar}), 7.74-7.82 (m, 2 H, CH_{Ar}) ppm. **^{13}C NMR** (151 MHz, $CDCl_3$): δ = 13.4 (q), 31.4 (d), 55.4 (q), 80.3 (t), 93.6 (s), 97.8 (d), 110.9 (d), 115.3 (d), 117.7 (d), 121.0 (d, 2 C), 126.6 (d), 129.4 (d, 2 C), 129.9 (d), 134.0 (s), 138.1 (s), 145.6 (s), 147.6 (s), 150.7 (s), 160.0 (s) ppm. **IR** (ATR): $\tilde{\nu}$ = 3839, 3617, 3324, 3070, 2920, 2841, 2727, 2491, 233, 2078, 1948, 1782, 1720, 1662, 1596, 1546, 1498, 1445, 1375, 1279, 1210, 1126, 1077, 1039, 911, 861, 787, 757, 691 cm^{-1}. **MS (EI$^+$, 70 eV)** *m/z* (%): 377.4 (1) $[M]^+$ = $[C_{21}H_{19}N_3O_4]^+$, 331.3 (19) $[M{-}NO_2]^+$ = $[C_{21}H_{19}NO_2]^+$, 330.3 (68) $[M{-}HNO_2]^+$ = $[C_{21}H_{18}N_2O_2]^+$, 318.3 (21), 317.2 (100) $[M{-}CH_2NO_2]^+$ = $[C_{20}H_{17}NO_2]^+$, 118.1 (16), 77.1 (26) $[C_6H_5]^+$. **MS (CI$^+$, methane)** *m/z* (%): 406.4 (6) $[M+C_2H_5]^+$ = $[C_{23}H_{22}N_3O_4]^+$, 378.3 (35) $[M+H]^+$ = $[C_{21}H_{20}N_3O_4]^+$. 362.1 (35) $[M+H]^+$ = $[C_{21}H_{20}N_3O_3]^+$. **HR-MS (ESI$^+$)** *m/z* (%): calcd. for $[M+H]^+$ = $[C_{21}H_{20}N_3O_4]^+$: 378.1448; found: 378.1455.

(*R*)-6-(Benzo[d][1,3]dioxol-5-yl)-3-methyl-4-(nitromethyl)-1-phenyl-1,4-dihydropyrano[2,3-*c*]pyrazole [4g]

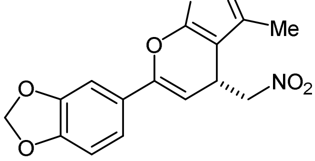

Compound **4g** was isolated after flash chromatography (SiO_2, *n*-pentane/EtOAc 3:1 to 1:1) as white solid (182 mg, 93%). **Molecular formula:** $C_{21}H_{17}N_3O_5$. **Molecular mass:** 391.38 g mol^{-1}. **R**$_f$(*n*-pentane/EtOAc 5:1) = 0.18. **Mp:** 148-151 °C. **HPLC:** AD, 95/5 *n*-heptane/EtOH, 1.0 ml/min, λ = 254 nm, τ_{minor} = 22.6 min, τ_{major} = 19.0 min. **OR:** $[\alpha]_D^{20}$ = −94.8 (*c* = 0.5, $CHCl_3$, 89% ee). **^{1}H NMR** (400 MHz, $CDCl_3$): δ = 2.32 (s, 3 H, C*H$_3$*), 4.43 (ddd, *J* = 4.0 Hz, *J* = 4.4 Hz, *J* = 8.4 Hz, 1 H, C*H*), 4.51 (dd, *J* = 8.4 Hz, *J* = 11.6 Hz, 1 H, C*H$_2$*), 4.66 (dd, *J* = 4.4 Hz, *J* = 11.7 Hz, 1 H, C*H$_2$*), 5.45 (d, *J* = 4.0 Hz, 1 H, C*H$_{ol}$*), 6.01 (s, 2 H, OCH_2O), 6.83 (d, *J* = 8.2 Hz, 1 H, C*H$_{Ar}$*), 7.06 (d, *J* = 1.7 Hz, 1 H, C*H$_{Ar}$*), 7.14 (dd, *J* = 1.8 Hz, *J* = 8.2 Hz, 1 H, C*H$_{Ar}$*), 7.27-7.34 (m, 1 H, C*H$_{Ar}$*), 7.44-7.51 (m, 2 H, C*H$_{Ar}$*), 7.72-7.77 (m, 2 H, C*H$_{Ar}$*) ppm. **^{13}C NMR** (100 MHz, $CDCl_3$): δ = 13.4 (q), 31.4 (d), 80.3 (t), 93.6 (s), 96.4 (d), 101.7 (t), 105.8 (d), 108.5 (d), 119.7 (d), 121.1 (d, 2 C), 126.0 (d), 126.8 (s), 129.4 (d, 2 C), 138.0 (s), 145.6 (s), 147.5 (s), 148.1 (s), 148.9 (s) 150.6 (s) ppm. **IR** (ATR): $\tilde{\nu}$ = 3840, 3649, 3326, 3080, 2910, 2785, 2700, 2460, 2287, 2230, 2164, 2077, 2051, 2007, 1962, 1856, 1715, 1662, 1600, 1546, 1497, 1439, 1380, 1360, 1284, 1251, 1188, 1108, 1071, 1036, 1000, 932, 853, 806, 757, 719, 691, 661 cm^{-1}. **MS (EI$^+$, 70 eV)** *m/z* (%): 391.4 (3) $[M]^+$ = $[C_{21}H_{17}N_3O_5]^+$, 345.3 (12) $[M-NO_2]^+$ = $[C_{21}H_{17}N_2O_3]^+$, 344.3 (43) $[M-HNO_2]^+$ = $[C_{21}H_{16}N_2O_3]^+$, 332.3 (21), 331.3 (100) $[M-CH_2NO_2]^+$ = $[C_{20}H_{15}N_2O_3]^+$, 172.1 (30), 165.3 (11), 159.1 (16), 149.0 (17), 118.1 (32), 77.1 (45) $[C_6H_5]^+$. **MS (CI$^+$, methane)** *m/z* (%): 420.4 (13) $[M+C_2H_5]^+$ = $[C_{23}H_{22}N_3O_5]^+$, 392.4 (71) $[M+H]^+$ = $[C_{21}H_{18}N_3O_5]^+$. **HR-MS (ESI$^+$)** *m/z* (%): calcd. for $[M+H]^+$ = $[C_{21}H_{18}N_3O_4]^+$: 392.1234; found: 392.1241.

(*R*)-3-Methyl-6-(naphthalen-1-yl)-4-(nitromethyl)-1-phenyl-1,3a,4,7a-tetrahydropyrano[2,3-*c*]pyrazole [4h]

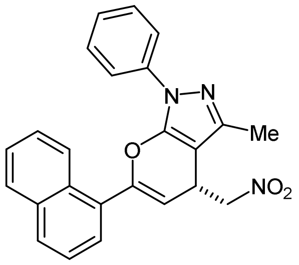

Compound **4h** was isolated after flash chromatography (SiO_2, *n*-pentane/EtOAc 3:1 to 1:1) as off-white solid (153 mg, 77%). **Molecular formula:** $C_{24}H_{19}N_3O_3$. **Molecular mass:** 397.43 g mol^{-1}. **R**$_f$(*n*-pentane/EtOAc 5:1) = 0.30. **Mp:** 150-153 °C. **HPLC:** OJ, 7/3 *n*-heptane/EtOH, 1.0 ml/min, λ = 230 nm, τ_{minor} = 32.7 min, τ_{major} = 11.5 min. **OR:** $[\alpha]_D^{20}$ = −169.9 (*c* = 0.7, $CHCl_3$, 93% ee). **^{1}H NMR** (400 MHz, $CDCl_3$): δ = 2.38 (s, 3 H, C*H$_3$*), 4.54 (ddd, *J* = 3.9 Hz, *J* = 4.6 Hz, *J* = 7.8 Hz, 1 H, C*H*), 4.67 (dd, *J* = 7.8 Hz, *J* = 11.6 Hz, 1 H, C*H$_2$*), 4.75 (dd, *J* = 4.6 Hz, *J* = 11.6 Hz, 1 H, C*H$_2$*), 5.42 (d, *J* = 3.9 Hz, 1 H, C*H$_{ol}$*), 7.14-7.20 (m, 1 H, C*H$_{Ar}$*), 7.28-7.34 (m, 2 H, C*H$_{Ar}$*), 7.47-7.56 (m, 3 H, C*H$_{Ar}$*), 7.61 (dd, *J* = 1.2 Hz, *J* = 7.1 Hz, 1 H, C*H$_{Ar}$*), 7.67-7.72 (m, 2 H, C*H$_{Ar}$*), 7.88-7.92 (m, 1 H, C*H$_{Ar}$*), 7.94 (d, *J* = 8.5 Hz, 1 H, C*H$_{Ar}$*), 8.10-8.16 (m, 1 H, C*H$_{Ar}$*) ppm. **^{13}C NMR** (100 MHz, $CDCl_3$): δ = 13.5 (q), 31.9 (d), 80.4 (t), 93.6 (s), 102.6 (d), 120.5 (d, 2 C), 125.3 (d, 2 C), 126.3 (d), 126.5 (d), 127.0 (d), 127.9 (d),

128.7 (d), 129.3 (d, 2 C), 130.5 (d), 131.2 (s), 131.4 (s), 133.8 (s), 138.1 (s), 145.6 (s), 147.9 (s), 151.7 (s) ppm. **IR** (ATR): $\tilde{\nu}$ = 3843, 3342, 3046, 2913, 2861, 2704, 2463, 2319, 2166, 2070, 1981, 1933, 1729, 1685, 1600, 1519, 1538, 1385, 1317, 1277, 1238, 1185, 1113, 1065, 1015, 969, 929, 855, 776, 752, 710, 688, 658 cm^{-1}. **MS (EI^+, 70 eV)** *m/z* (%): 397.0 (5) $[M]^+ = [C_{24}H_{19}N_3O_3]^+$, 351.0 (18) $[M-NO_2]^+ = [C_{24}H_{17}N_2O]^+$, 350.0 (70) $[M-HNO_2]^+ = [C_{24}H_{16}N_2O]^+$, 338.0 (22), 336.9 (100) $[M-CH_2NO_2]^+ = [C_{23}H_{17}N_2O]^+$, 175.0 (12), 164.9 (18), 151.9 (25), 126.8 (10), 117.9 (25), 76.9 (89) $[C_6H_5]^+$. **MS (CI^+, methane)** *m/z* (%): 426.2 (5) $[M+C_2H_5]^+ = [C_{26}H_{24}N_3O_3]^+$, 398.1 (61) $[M+H]^+ = [C_{24}H_{20}N_3O_3]^+$. **HR-MS ($ESI^+$)** *m/z* (%): calcd. for $[M+H]^+ = [C_{24}H_{20}N_3O_3]^+$: 398.1493; found: 398.1499.

(*R*)-3-Methyl-6-(naphthalen-2-yl)-4-(nitromethyl)-1-phenyl-1,3a,4,7a-tetrahydropyrano[2,3-*c*]pyrazole [4i]

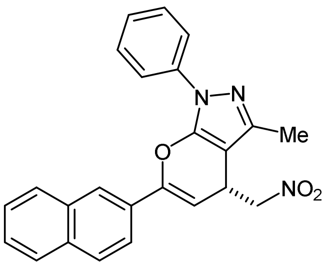

Compound **4i** was isolated after flash chromatography (SiO_2, *n*-pentane/EtOAc 3:1 to 1:1) as off-white solid (182 mg, 91%). **Molecular formula:** $C_{24}H_{19}N_3O_3$. **Molecular mass:** 397.43 g mol^{-1}. $\mathbf{R_f}$(*n*-pentane/EtOAc 5:1) = 0.26. **Mp:** 168-172 °C. **HPLC:** AD, 97/3 *n*-heptane/EtOH, 1.0 ml/min, λ = 250 nm, τ_{minor} = 22.8 min, τ_{major} = 19.2 min. **OR:** $[\alpha]_D^{20}$ = −52.4 (*c* = 0.7, $CHCl_3$, 91% ee). **^{1}H NMR** (600 MHz, $CDCl_3$): δ = 2.35 (s, 3 H, CH_3), 4.51 (ddd, *J* = 4.0 Hz, *J* = 4.4 Hz, *J* = 8.6 Hz, 1 H, C*H*), 4.57 (dd, *J* = 8.6 Hz, *J* = 11.8 Hz, 1 H, CH_2), 4.71 (dd, *J* = 4.4 Hz, *J* = 11.8 Hz, 1 H, CH_2), 5.75 (d, *J* = 4.0 Hz, 1 H, CH_{ol}), 7.31-7.37 (m, 1 H, CH_{Ar}), 7.49-7.56 (m, 4 H, CH_{Ar}), 7.68 (dd, *J* = 1.8 Hz, *J* = 8.7 Hz, 1 H, CH_{Ar}), 7.81-7.89 (m, 5 H, CH_{Ar}), 8.11 (d, *J* = 0.9 Hz, 1 H, CH_{Ar}) ppm. **^{13}C NMR** (150 MHz, $CDCl_3$): δ = 13.5 (q), 31.5 (d), 80.3 (t), 93.6 (s), 98.1 (d), 121.1 (d, 2 C), 122.6 (d), 124.9 (d), 126.6 (d), 126.9 (d), 127.(1), 127.9 (d), 128.6 (d), 128.7 (d), 129.4 (d, 2 C), 129.8 (s), 133.1 (s), 133.8 (s), 138.1 (s), 145.7 (s), 147.6 (s), 150.9 (s) ppm. **IR** (ATR): $\tilde{\nu}$ = 3837, 3394, 3060, 2918, 2720, 2457, 2290, 2194, 2071, 1993, 1933, 1734, 1661, 1601, 1541, 1443, 1380, 1285, 1240, 1190, 1126, 1075, 1006, 944, 896, 857, 812, 750, 690, 663 cm^{-1}. **MS (EI^+, 70 eV)** *m/z* (%): 351.0 (58) $[M-NO_2]^+ = [C_{24}H_{19}N_2O]^+$, 350.0 (16) $[M-HNO_2]^+ = [C_{24}H_{18}N_2O]^+$, 338.1 (21), 337.0 (92) $[M-CH_2NO_2]^+ = [C_{23}H_{17}N_2O]^+$, 174.9 (16), 168.4 (14), 164.9 (36), 154.9 (14), 151.9 (11), 126.9 (29), 117.9 (56), 90.9 (10), 76.9 (100) $[C_6H_5]^+$, 57.1 (11), 55.1 (10), 51.1 (17). **MS (CI^+, methane)** *m/z* (%): 426.0 (8) $[M+C_2H_5]^+ = [C_{26}H_{24}N_3O_3]^+$, 398.0 (46) $[M+H]^+ = [C_{24}H_{20}N_3O_3]^+$. $]^+$. **HR-MS (ESI^+)** *m/z* (%): calcd. for $[M+H]^+ = [C_{24}H_{20}N_3O_3]^+$: 398.1499; found: 398.1507.

(*R*)-6-(Furan-2-yl)-3-methyl-4-(nitromethyl)-1-phenyl-1,4-dihydropyrano[2,3-*c*]pyrazole [4j]

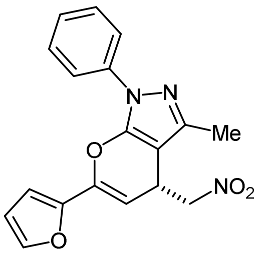

Compound **4j** was isolated after flash chromatography (SiO_2, *n*-pentane/EtOAc 3:1 to 1:1) as off-white solid (158 mg, 93%). **Molecular formula:** $C_{18}H_{15}N_3O_4$. **Molecular mass:** 337.33 mol^{-1}. **R_f**(*n*-pentane/EtOAc 5:1) = 0.30. **Mp:** 172-175 °C. **HPLC:** OJ, 7/3 *n*-heptane/EtOH, 1.0 ml/min, λ = 254 nm, τ_{minor} = 15.3 min, τ_{major} = 10.4 min. **OR:** $[\alpha]_D^{20}$ = −123.3 (*c* = 0.6, $CHCl_3$, 99% ee). **^{1}H NMR** (600 MHz, $CDCl_3$): δ = 2.31 (s, 3 H, CH_3), 4.45 (ddd, *J* = 4.1 Hz, *J* = 4.6 Hz, *J* = 8.3 Hz, 1 H, C*H*), 4.51 (dd, *J* = 8.3 Hz, *J* = 11.9 Hz, 1 H, CH_2), 4.66 (dd, *J* = 4.6 Hz, *J* = 11.9 Hz, 1 H, CH_2), 5.65 (d, *J* = 4.1 Hz, 1 H, CH_{ol}), 6.47 (dd, *J* = 1.3 Hz, *J* = 3.4 Hz, 1 H, CH_{Ar}), 6.59 (d, *J* = 3.3 H, 1 H, CH_{Ar}), 7.28-7.33 (m, 1 H, CH_{Ar}), 7.45 (d, *J* = 1.0 Hz, 1 H, CH_{Ar}), 7.46-7.50 (m, 2 H, CH_{Ar}), 7.73-7.77 (m, 2 H, CH_{Ar}) ppm. **^{13}C NMR** (151 MHz, $CDCl_3$): δ = 13.4 (q), 30.9 (d), 80.1 (t), 93.7 (s), 96.0 (d), 108.3 (d), 111.6 (d), 120.9 (d, 2 C), 126.6 (d), 129.3 (d, 2 C), 138.0 (s), 140.3 (s), 140.6 (s), 145.7 (s), 146.8 (s, 2 C) ppm. **IR** (ATR): $\tilde{\nu}$ = 3860, 3141, 3056, 2918, 2853, 2695, 2317, 2069, 1733, 1676, 1601, 1526, 1442, 1385, 1319, 1237, 1196, 1157, 1121, 1083, 1002, 925, 878, 831, 802, 754, 693, 664 cm^{-1}. **MS (EI$^+$, 70 eV)** *m*/*z* (%): 337.0 (5) $[M]^+$ = $[C_{18}H_{15}N_3O_4]^+$, 291.1 (31) $[M{-}NO_2]^+$ = $[C_{18}H_{15}N_2O_2]^+$, 290.0 (100) $[M{-}HNO_2]^+$ = $[C_{18}H_{14}N_2O_2]^+$, 278.0 (22), 277.0 (95) $[M{-}CH_2NO_2]^+$ = $[C_{17}H_{13}N_2O_2]^+$, 117.9 (13), 76.9 $[C_6H_5]^+$. **MS (CI$^+$, methane)** *m*/*z* (%): 337.9 (1) $[M+H]^+$ = $[C_{18}H_{16}N_3O_4]^+$. **HR-MS (ESI$^+$)** *m*/*z* (%): calcd. for $[M+H]^+$ = $[C_{18}H_{16}N_3O_4]^+$: 338.1135; found: 338.1136.

(*R*)-3-Methyl-4-(nitromethyl)-1-phenyl-6-(thiophen-2-yl)-1,4-dihydropyrano[2,3-*c*]pyrazole [4k]

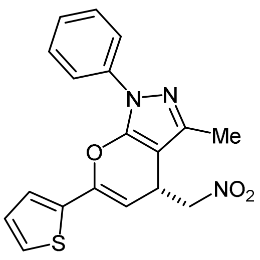

Compound **4k** was isolated after flash chromatography (SiO_2, *n*-pentane/EtOAc 3:1 to 1:1) as off-white solid (163 mg, 92%). **Molecular formula:** $C_{18}H_{15}N_3O_3S$. **Molecular mass:** 353.40 mol^{-1}. **R_f**(*n*-pentane/EtOAc 5:1) = 0.31. **Mp:** 175-180 °C. **HPLC:** OJ, 7/3 *n*-heptane/EtOH, 1.0 ml/min, λ = 254 nm, τ_{minor} = 24.3 min, τ_{major} = 12.2 min. **OR:** $[\alpha]_D^{20}$ = −111.9 (*c* = 0.8, $CHCl_3$, 98% ee). **^{1}H NMR** (600 MHz, $CDCl_3$): δ = 2.32 (s, 3 H, CH_3), 4.44 (ddd, *J* = 4.1 Hz, *J* = 4.5 Hz, *J* = 8.5 Hz, 1 H, C*H*), 4.52 (dd, *J* = 8.5 Hz, *J* = 12.0 Hz, 1 H, CH_2), 4.66 (dd, *J* = 4.5 Hz, *J* = 12.0 Hz, 1 H, CH_2), 5.54 (d, *J* = 4.1

Hz, 1 H, CH_{ol}), 7.06 (dd, J = 3.8 Hz, J = 4.9 Hz, 1 H, CH_{Ar}), 7.28-7.32 (m, 1 H, CH_{Ar}), 7.32-7.35 (m, 2 H, CH_{Ar}), 7.46-7.51 (m, 2 H, CH_{Ar}), 7.76-7.81 (m, 2 H, CH_{Ar}) ppm. **^{13}C NMR** (151 MHz, $CDCl_3$): δ = 13.4 (q), 31.3 (d), 80.1 (t), 93.5 (s), 96.5 (d), 120.9 (d, 2 C), 125.2 (d), 126.5 (d), 126.6 (d), 127.8 (d), 129.4 (d, 2 C), 135.8 (s), 138.0 (s), 145.6 (s), 146.5 (s), 147.1 (s) ppm. **IR** (ATR): $\tilde{\nu}$ = 3100, 2921, 2853, 2324, 2072, 1801, 1731, 1662, 1600, 1543, 1513, 1432, 1398, 1371, 1326, 1279, 1241, 1911, 1124, 1072, 1039, 978, 909, 851, 798, 753, 700, 659 cm^{-1}. **MS (EI$^+$, 70 eV)** *m*/*z* (%): 353.0 (2) $[M]^+$ = $[C_{18}H_{15}N_3O_3S]^+$, 307.0 (24) $[M{-}NO_2]^+$ = $[C_{18}H_{15}N_2OS]^+$, 306.0 (82) $[M{-}HNO_2]^+$ = $[C_{18}H_{14}N_2OS]^+$, 294.0 (24), 293.0 (95) $[M{-}CH_2NO_2]^+$ = $[C_{17}H_{13}N_2OS]^+$, 117.9 (14), 76.9 (17) $[C_6H_5]^+$. **MS (CI$^+$, methane)** *m*/*z* (%): 382.1 (10) $[M{+}C_2H_5]^+$ = $[C_{20}H_{20}N_3O_3S]^+$, 354.0 (44) $[M{+}H]^+$ = $[C_{18}H_{16}N_3O_3S]^+$. **HR-MS (ESI$^+$)** *m*/*z* (%): calcd. for $[C_{18}H_{16}N_3O_3S]^+$ = $[C_{18}H_{16}O_3N_3S]^+$: 354.0907; found: 354.0902.

(*R*)-1-(2-Chlorophenyl)-3-methyl-4-(nitromethyl)-6-phenyl-1,4-dihydropyrano[2,3-*c*]pyrazole [4l]

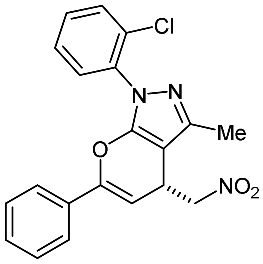

Compound **4l** was isolated after flash chromatography (SiO_2, *n*-pentane/EtOAc 3:1 to 1:1) as yellow solid (167 mg, 87%). **Molecular formula:** $C_{20}H_{16}ClN_3O_3$. **Molecular mass:** 381.82 mol^{-1}. **R$_f$**(*n*-pentane/EtOAc 5:1) = 0.43. **Mp:** 58-63 °C. **HPLC:** IA, 9/1 *n*-heptane/EtOH, 0.5 ml/min, λ = 254 nm, τ_{minor} = 15.8 min, τ_{major} = 17.2 min. **OR:** $[\alpha]_D^{20}$ = −67.9 (c = 0.6, $CHCl_3$, 91% ee). **^{1}H NMR** (600 MHz, $CDCl_3$): δ = 2.34 (m, 3 H, CH_3), 4.50 (ddd, J = 4.0 Hz, J = 4.3 Hz, J = 8.5 Hz, 1 H, CH), 4.55 (dd, J = 8.5 Hz, J = 11.7 Hz, 1 H, CH_2), 4.71 (dd, J = 4.3 Hz, J = 11.7 Hz, 1 H, CH_2), 5.58 (d, J = 4.0 Hz, 1 H, CH_{ol}), 7.32-7.37 (m, 3 H, CH_{Ar}), 7.38-7.44 (m, 2 H, CH_{Ar}), 7.48-7.52 (m, 1 H, CH_{Ar}), 7.52-7.55 (m, 2 H, CH_{Ar}), 7.55-7.59 (m, 1 H, CH_{Ar}) ppm. **^{13}C NMR** (151 MHz, $CDCl_3$): δ = 13.5 (q), 31.6 (d), 80.2 (t), 92.4 (s), 97.4 (d), 125.3 (d, 2 C), 127.7 (d), 128.7 (d, 2 C), 129.3 (d), 129.6 (d), 130.3 (d), 130.5 (d), 131.3 (s), 132.5 (s), 135.0 (s), 146.3 (s), 148.7 (s), 150.8 (s) ppm. **IR** (ATR): $\tilde{\nu}$ = 3066, 2920, 2737, 2323, 2193, 2069, 1978, 1811, 1730, 1662, 1602, 1544, 1489, 1441, 1376, 1324, 1280, 1250, 1191, 1094, 1060, 1034, 887, 914, 846, 813, 759, 727, 690 cm^{-1}. **MS (EI$^+$, 70 eV)** *m*/*z* (%): 337.0 (11) $[M{-}NO_2, {}^{37}Cl]^+$ = $[C_{20}H_{16}ClN_2O]^+$, 336.0 (47) $[M{-}HNO_2, {}^{37}Cl]^+$ = $[C_{20}H_{15}ClN_2O]^+$, 335.0 (31) $[M{-}NO_2, {}^{35}Cl]^+$ = $[C_{20}H_{16}ClN_2O]^+$, 334.0 (96) $[M{-}HNO_2, {}^{35}Cl]^+$ = $[C_{20}H_{15}ClN_2O]^+$, 323.1 (48) $[M{-}CH_2NO_2, {}^{37}Cl]^+$ = $[C_{19}H_{14}ClN_2O]^+$, 322.0 (29), 321.0 (100) $[M{-}CH_2NO_2, {}^{35}Cl]^+$ = $[C_{19}H_{14}ClN_2O]^+$, 151.9 (22), 114.9 (16), 110.8 (11). **MS (CI$^+$, methane)** *m*/*z* (%): 409.9 (6) $[M{+}C_2H_5, {}^{35}Cl]^+$ = $[C_{22}H_{21}ClN_3O_3]^+$, 384.0 (10) $[M{+}H, {}^{37}Cl]^+$ = $[C_{20}H_{17}ClN_3O_3]^+$, 382.0 (36) $[M{+}H, {}^{35}Cl]^+$ =

$[C_{20}H_{17}ClN_3O_3]^+$.]$^+$. **HR-MS (ESI$^+$)** *m/z* (%): calcd. for [M+H, $^{35}Cl]^+$ = $[C_{20}H_{17}ClO_3N_3]^+$: 382.0953; found: 382.0953.

(*R*)-1-(4-Chlorophenyl)-3-methyl-4-(nitromethyl)-6-phenyl-1,4-dihydropyrano[2,3-*c*]pyrazole [4m]

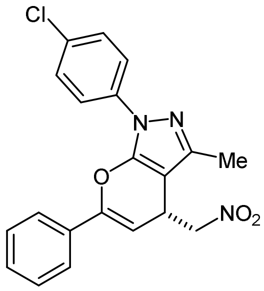

Compound **4m** was isolated after flash chromatography (SiO_2, *n*-pentane/EtOAc 3:1 to 1:1) as off-white solid (178 mg, 93%). **Molecular formula:** $C_{20}H_{16}ClN_3O_3$. **Molecular mass:** 381.82 mol^{-1}. **R$_f$**(*n*-pentane/EtOAc 5:1) = 0.39. **Mp:** 178-182 °C. **HPLC:** OJ, 7/3 *n*-heptane/EtOH, 0.7 ml/min, λ = 254 nm, τ_{minor} = 22.3 min, τ_{major} = 12.6 min. **OR:** $[\alpha]_D^{20}$ = −120.0 (c = 0.6, $CHCl_3$, 99% ee). **^{1}H NMR** (600 MHz, $CDCl_3$): δ = 2.32 (m, 3 H, C*H$_3$*), 4.46 (ddd, *J* = 4.0 Hz, *J* = 4.5 Hz, *J* = 8.4 Hz, 1 H, C*H*), 4.53 (dd, *J* = 8.4 Hz, *J* = 11.8 Hz, 1 H, C*H$_2$*), 4.67 (dd, *J* = 4.5 Hz, *J* = 11.8 Hz, 1 H, C*H$_2$*), 5.60 (d, *J* = 4.0 Hz, 1 H, CH_{ol}), 7.40-7.48 (m, 5 H, CH_{Ar}), 7.57-7.63 (m, 2 H, CH_{Ar}), 7.71-7.76 (m, 2 H, CH_{Ar}) ppm. **^{13}C NMR** (151 MHz, $CDCl_3$): δ = 13.4 (q), 31.4 (d), 80.2 (t), 93.8 (s), 97.7 (d), 122.0 (d, 2 C), 125.3 (d, 2 C), 128.9 (d, 2 C), 129.5 (d, 2 C), 129.9 (d), 132.0 (s), 132.5 (s), 136.6 (s), 146.0 (s), 147.6 (s), 150.9 (s) ppm. **IR** (ATR): $\tilde{\nu}$ = 3841, 3115, 2917, 2881, 2665, 2456, 2331, 2254, 2195, 2162, 2109, 2056, 1991, 1902, 1723, 1660, 1603, 1545, 1512, 1490, 1446, 1411, 1375, 1328, 1274, 1242, 1178, 1077, 1036, 999, 941, 911, 828, 759, 681 cm^{-1}. **MS (EI$^+$, 70 eV)** *m/z* (%): 336.0 (34) [M−HNO_2, $^{37}Cl]^+$ = $[C_{20}H_{15}ClN_2O]^+$, 335.0 (26) [M−NO_2, $^{35}Cl]^+$ = $[C_{20}H_{16}ClN_2O]^+$, 334.0 (90) [M−HNO_2, $^{35}Cl]^+$ = $[C_{20}H_{15}ClN_2O]^+$, 323.0 (37) [M−CH_2NO_2, $^{37}Cl]^+$ = $[C_{19}H_{14}ClN_2O]^+$, 322.0 (25), 321.0 (100) [M−CH_2NO_2, $^{35}Cl]^+$ = $[C_{19}H_{14}ClN_2O]^+$, 151.9 (12). **MS (CI$^+$, methane)** *m/z* (%): 410.0 (25) [M+C_2H_5, $^{35}Cl]^+$ = $[C_{22}H_{21}ClN_3O_3]^+$, 384.0 (30) [M+H, $^{37}Cl]^+$ = $[C_{20}H_{17}ClN_3O_3]^+$, 382.0 (100) [M+H, $^{35}Cl]^+$ = $[C_{20}H_{17}ClN_3O_3]^+$. **HR-MS (ESI$^+$)** *m/z* (%): calcd. for [M+H, $^{35}Cl]^+$ = $[C_{20}H_{17}ClO_3N_3]^+$: 382.0953; found: 382.0954.

(*R*)-1-Methyl-4-(nitromethyl)-6-phenyl-3-(trifluoromethyl)-1,4-dihydropyrano[2,3-*c*]pyrazole [4n]

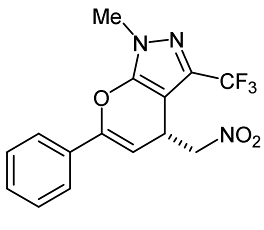

Compound **4n** was isolated after flash chromatography (SiO_2, *n*-pentane/EtOAc 3:1 to 1:1) as off-white solid (153 mg, 90%). **Molecular formula:** $C_{15}H_{12}F_3N_3O_3$. **Molecular mass:** 339.27 g mol^{-1}. $\mathbf{R_f}$(*n*-pentane/EtOAc 5:1) = 0.47. **Mp:** 122-125 °C. **HPLC:** AD, 9/1 *n*-heptane/*i*PrOH, 1.0 ml/min, λ = 230 nm, τ_{minor} = 7.0 min, τ_{major} = 8.8 min. **OR:** $[\alpha]_D^{20}$ = −99.4 (c = 0.7, $CHCl_3$, 99% ee). **^{1}H NMR** (400 MHz, $CDCl_3$): δ = 3.87 (s, 3 H, NCH_3), 4.47-4.56 (m, 2 H, C*H*, CH_2), 4.66-4.82 (m, 1 H, CH_2), 5.52-5.66 (m, 1 H, CH_{ol}), 7.39-7.47 (m, 3 H, CH_{Ar}), 7.58-7.66 (m, 2 H, CH_{Ar}) ppm. **^{13}C{^{19}F} NMR** (100 MHz, $CDCl_3$): δ = 31.0 (d), 34.8 (q), 80.0 (t), 92.4 (s), 97.4 (d), 121.3 (s), 125.3 (d, 2 C), 128.8 (d, 2 C), 130.0 (d), 132.2 (s), 136.8 (s), 148.7 (s), 150.6 (s) ppm. **^{19}F{^{1}H}** (376 MHz, $CDCl_3$): δ = −61.2 (s) ppm. **IR** (ATR): $\tilde{\nu}$ = 3083, 2950, 2854, 2204, 2138, 1980, 1664, 1591, 1546, 1501, 1432, 1400, 1378, 1332, 1271, 1240, 1214, 1162, 1116, 1072, 1042, 1016, 924, 847, 763, 722, 688 cm^{-1}. **MS (EI$^+$, 70 eV)** *m/z* (%): 293.1 (18) $[M-NO_2]^+$ = $[C_{15}H_{12}F_3N_2O]^+$, 292.1 (100) $[M-HNO_2]^+$ = $[C_{15}H_{11}F_3N_2O]^+$, 279.1 (100) $[M-CH_2NO_2]^+$ = $[C_{14}H_{10}F_3N_2O]^+$, 146.0 (11), 139.1 (15), 115.0 (19), 111.1 (11), 105.0 (22), 102.0 (19), 97.1 (15), 95.1 (10), 85.1 (12), 83.1 (15), 81.1 (11), 77.1 (26) $[C_6H_5]^+$, 71.2 (26), 69.2 (20), 57.2 (27), 55.2 (10). **MS (CI$^+$, methane)** *m/z* (%): 340.0 (1) $[M+H]^+$ = $[C_{15}H_{13}F_3N_3O_3]^+$. **EA:** calcd. for $C_{15}H_{12}F_3N_3O_3$: C 53.10%, H 3.57 %, N 12.30%; found: C 53.30%, H 3.66%, N 12.32%.

(*R*)-1,3-Dimethyl-4-(nitromethyl)-6-phenyl-1,4-dihydropyrano[2,3-*c*]pyrazole [o]

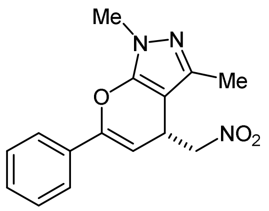

Compound **4o** was isolated after flash chromatography (SiO_2, DCM/EtOAc 5:1) as off-white solid (69 mg, 48%). **Molecular formula:** $C_{15}H_{15}N_3O_3$. **Molecular mass:** 285.30 mol^{-1}. $\mathbf{R_f}$(*n*-pentane/EtOAc 3:1) = 0.14. **Mp:** 132-135 °C. **HPLC:** IA, 9/1 *n*-heptane/EtOH, 0.7 ml/min, λ = 254 nm, τ_{minor} = 11.2 min, τ_{major} = 12.7 min. **OR:** $[\alpha]_D^{20}$ = −108.1 (c = 0.5, $CHCl_3$, 99% ee). **^{1}H NMR** (600 MHz, $CDCl_3$): δ = 2.23 (s, 3 H, CH_3), 3.74 (s, 3 H, NCH_3), 4.40 (ddd, J = 4.0 Hz, J = 4.4 Hz, J = 8.5 Hz, 1 H, C*H*), 4.45 (dd, J = 8.5 Hz, J = 11.6 Hz, 1 H, CH_2), 4.62 (dd, J = 4.4 Hz, J = 11.6 Hz, 1 H, CH_2), 5.53 (d, J = 4.0 Hz, 1 H, CH_{ol}), 7.37-7.47 (m, 3 H, CH_{Ar}), 7.59-7.68 (m, 2 H, CH_{Ar}) ppm. **^{13}C NMR** (151 MHz, $CDCl_3$): δ = 13.3 (q), 33.6 (d), 33.7 (q), 80.4 (t), 91.5 (s), 97.6 (d), 125.3 (d, 2 C), 128.7 (d, 2 C), 129.7 (d), 132.9 (s), 143.7 (s), 148.1 (s), 150.6 (s) ppm. **IR** (ATR): $\tilde{\nu}$ = 3133, 3064, 2932, 2860, 2728, 2513, 2459, 2327, 2199, 2167, 2082, 1996, 1919, 1719, 1623, 1574, 1536, 1489, 1462, 1441, 1351, 1307, 1277, 1228, 1160, 1102, 1077, 1018, 990, 933, 857, 785, 766, 723, 687 cm^{-1}. **MS (EI$^+$, 70 eV)** *m/z* (%): 239.1 (14), 238.0 (63) $[M-HNO_2]^+$ = $[C_{15}H_{14}N_2O]^+$, 226.0 (17), 225.0 (100) $[M-CH_2NO_2]^+$ = $[C_{14}H_{13}N_2O]^+$. **MS (CI$^+$, methane)** *m/z* (%): 314.0 (5) $[M+C_2H_5]^+$ = $[C_{17}H_{20}N_3O_3]^+$, 286.0 (29) $[M+H]^+$ = $[C_{15}H_{16}N_3O_3]^+$. **EA:** calcd. for $C_{15}H_{15}N_3O_3$: C 63.15%, H 5.30%, N 14.73%; found: C 63.41%, H 5.62%, N 14.20%.

(*R*)-6-Butyl-3-methyl-4-(nitromethyl)-1-phenyl-1,4-dihydropyrano[2,3-*c*]pyrazole [4p]

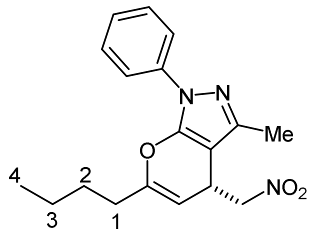

Compound **4p** was isolated after flash chromatography (SiO_2, *n*-pentane/EtOAc 3:1) as off-white solid (156 mg, 95%). **Molecular formula:** $C_{18}H_{21}N_3O_3$. **Molecular mass:** 327.38 mol^{-1}. $\mathbf{R_f}$(*n*-pentane/EtOAc 5:1) = 0.43. **Mp:** 87-91 °C. **HPLC:** IA, 9/1 *n*-heptane/EtOH, 0.5 ml/min, λ = 254 nm, τ_{minor} = 10.3 min, τ_{major} = 10.9 min. **OR:** $[\alpha]_D^{20}$ = −161.6 (c = 0.5, $CHCl_3$, 99% ee). **^{1}H NMR** (600 MHz, $CDCl_3$): δ = 0.93 (t, J = 7.4 Hz, 3 H, 4-CH_3), 1.34-1.43 (m, 2 H, 3-CH_2), 1.52-1.59 (m, 2 H, 2-CH_2), 2.27-2.32 (m, 5 H, 1-CH_2, CH_3), 4.25 (ddd, J = 3.8 Hz, J = 4.6 Hz, J = 8.4 Hz, 1 H, CH), 4.41 (dd, J = 8.4 Hz, J = 11.7 Hz, 1 H, CH_2), 4.57 (dd, J = 4.6 Hz, J = 11.7 Hz, 1 H, CH_2), 4.84 (d, J = 3.8 Hz, 1 H, CH_{ol}), 7.23-7.30 (m, 1 H, CH_{Ar}), 7.40-7.46 (m, 2 H, CH_{Ar}), 7.66-7.73 (m, 2 H, CH_{Ar}) ppm. **^{13}C NMR** (151 MHz, $CDCl_3$): δ = 13.4 (q), 13.9 (q), 22.0 (t), 28.9 (t), 31.0 (d), 32.7 (t), 80.5 (t), 93.6 (s), 96.7 (d), 120.7 (d, 2 C), 126.3 (d), 129.2 (d, 2 C), 138.1 (s), 145.6 (s), 147.9 (s), 153.6 (s) ppm. **IR** (ATR): $\tilde{\nu}$ = 3676, 3346, 3068, 2953, 2927, 2866, 2735, 2460, 2318, 2223, 2193, 1251, 2071, 1983, 1938, 1810, 1685, 1603, 1542, 1515, 1435, 1399, 1380, 1321, 1275, 1243, 1166, 1125, 1066, 1029, 988, 935, 904, 847, 795, 754, 721, 689, 661 cm^{-1}. **MS (EI$^+$, 70 eV)** m/z (%): 327.0 (4) $[M]^+$ = $[C_{18}H_{21}N_3O_3]^+$, 281.1 (18) $[M-NO_2]^+$ = $[C_{18}H_{21}N_2O]^+$, 280.1 (90) $[M-HNO_2]^+$ = $[C_{18}H_{20}N_2O]^+$, 268.1 (19), 267.1 (100) $[M-CH_2NO_2]^+$ = $[C_{17}H_{19}N_2O]^+$, 117.9 (14), 76.9 (21) $[C_6H_5]^+$. **MS (CI$^+$, methane)** m/z (%): 356.1 (14) $[M+C_2H_5]^+$ = $[C_{20}H_{26}N_3O_3]^+$, 328.0 (60) $[M+H]^+$ = $[C_{19}H_{22}N_3O_3]^+$. **EA:** calcd. for $C_{18}H_{21}N_3O_3$: C 66.04%, H 6.47%, N 12.84%; found: C 65.80%, H 6.63%, N 12.44%.

(*R*)-6-Cyclopentyl-3-methyl-4-(nitromethyl)-1-phenyl-1,4-dihydropyrano[2,3-*c*]pyrazole [4q]

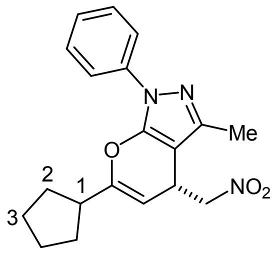

Compound **4q** was isolated after flash chromatography (SiO_2, *n*-pentane/EtOAc 3:1) as white solid (154mg, 91%). **Molecular formula:** $C_{19}H_{21}N_3O_3$. **Molecular mass:** 339.39 mol^{-1}. $\mathbf{R_f}$(*n*-pentane/EtOAc 5:1) = 0.42. **Mp:** 87-91 °C. **HPLC:** OD, 9/1 *n*-heptane/*i*PrOH, 0.5 ml/min, λ = 254 nm, τ_{minor} = 13.8 min, τ_{major} = 12.4 min. **OR:** $[\alpha]_D^{20}$ = −139.0 (c = 0.5, $CHCl_3$, 99% ee). **^{1}H NMR** (600 MHz, $CDCl_3$): δ = 1.55-1.68 (m, 4 H, 2-CH_2, 2-CH_2), 1.69-1.81 (m, 2 H, 3-CH_2), 1.86-1.98 (m, 2 H, 2-CH_2),

2.27 (s, 3 H, CH_3), 2.72 (p, J = 8.1 Hz, 1 H, 1-CH), 4.25 (ddd, J = 3.8 Hz, J = 4.6 Hz, J = 8.4 Hz, 1 H, CH), 4.41 (dd, J = 8.4 Hz, J = 11.7 Hz, 1 H, CH_2), 4.57 (dd, J = 4.6 Hz, J = 11.7 Hz, 1 H, CH_2), 4.87 (d, J = 3.8 Hz, 1 H, CH_{ol}), 7.24-7.29 (m, 1 H, CH_{Ar}), 7.40-7.47 (m, 2 H, CH_{Ar}), 7.67-7.73 (m, 2 H, CH_{Ar}) ppm. **^{13}C NMR** (151 MHz, $CDCl_3$): δ = 13.4 (q), 25.6 (t, 2 C), 30.7 (t, 2 C), 31.0 (d), 42.8 (d), 80.5 (t), 93.7 (s), 95.2 (d), 120.2 (d, 2 C), 126.3 (d), 129.2 (d, 2 C), 138.2 (s), 145.6 (s), 147.9 (s), 156.5 (s) ppm. **IR** (ATR): $\tilde{\nu}$ = 3064, 2961, 2869, 2322, 2242, 2168, 2072, 1995, 1802, 1685, 1600, 1514, 1439, 1377, 1315, 1267, 1250, 1185, 1123, 1066, 1030, 1009, 934, 907, 845, 793, 756, 688, 660 cm^{-1}. **MS (EI$^+$, 70 eV)** m/z (%): 339.1 (8) $[M]^+$ = $[C_{19}H_{21}N_3O_3]^+$, 293.1 (26) $[M-NO_2]^+$ = $[C_{19}H_{21}N_2O]^+$, 292.1 (97) $[M-HNO_2]^+$ = $[C_{19}H_{20}N_2O]^+$, 280.1 (23), 279.1 (100) $[M-CH_2NO_2]^+$ = $[C_{18}H_{19}N_2O]^+$, 76.9 (11) $[C_6H_5]^+$. **MS (CI$^+$, methane)** m/z (%): 340.1 (44) $[M+H]^+$ = $[C_{19}H_{22}N_3O_3]^+$. **EA:** calcd. for $C_{19}H_{21}N_3O_3$: C 67.24%, H 6.42%, N 12.38%; found: C 67.35%, H 5.91%, N 12.21%.

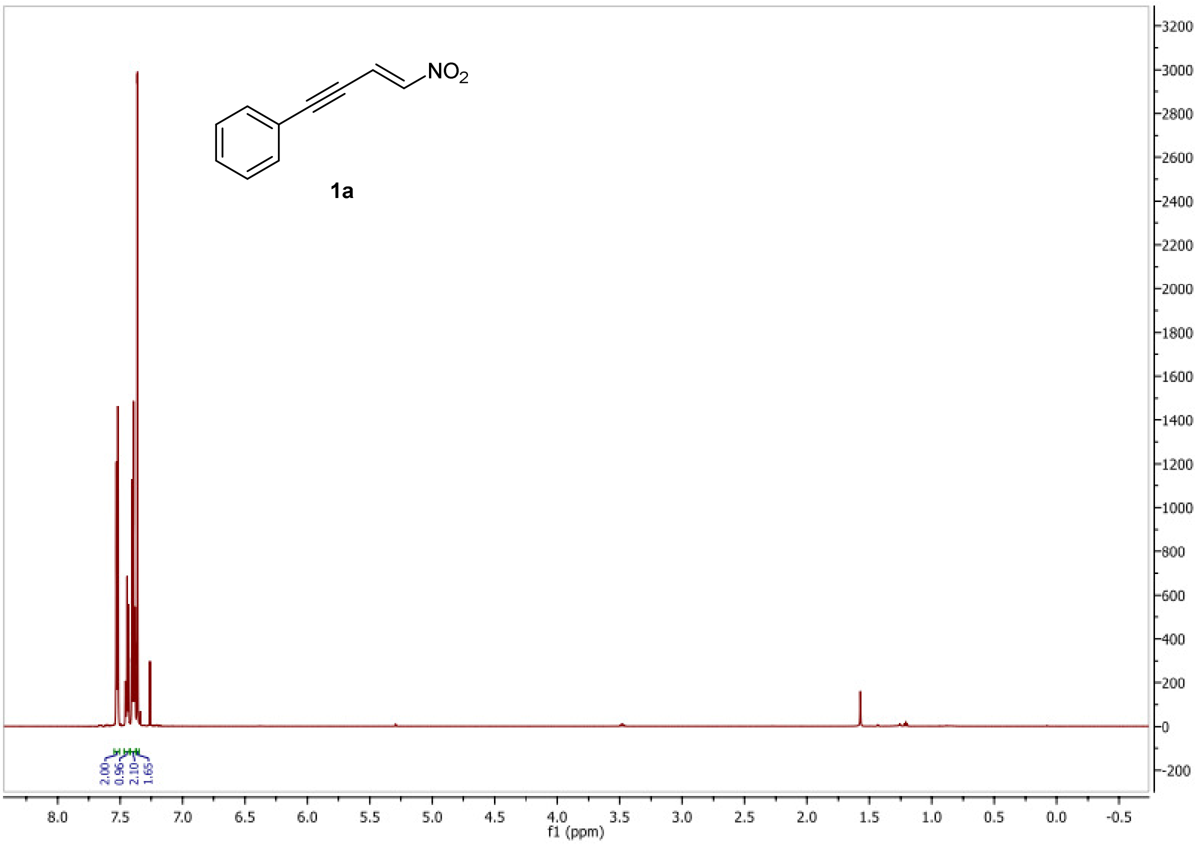

NO2
1a
2.00
0.96
2.10
1.65
f1 (ppm)

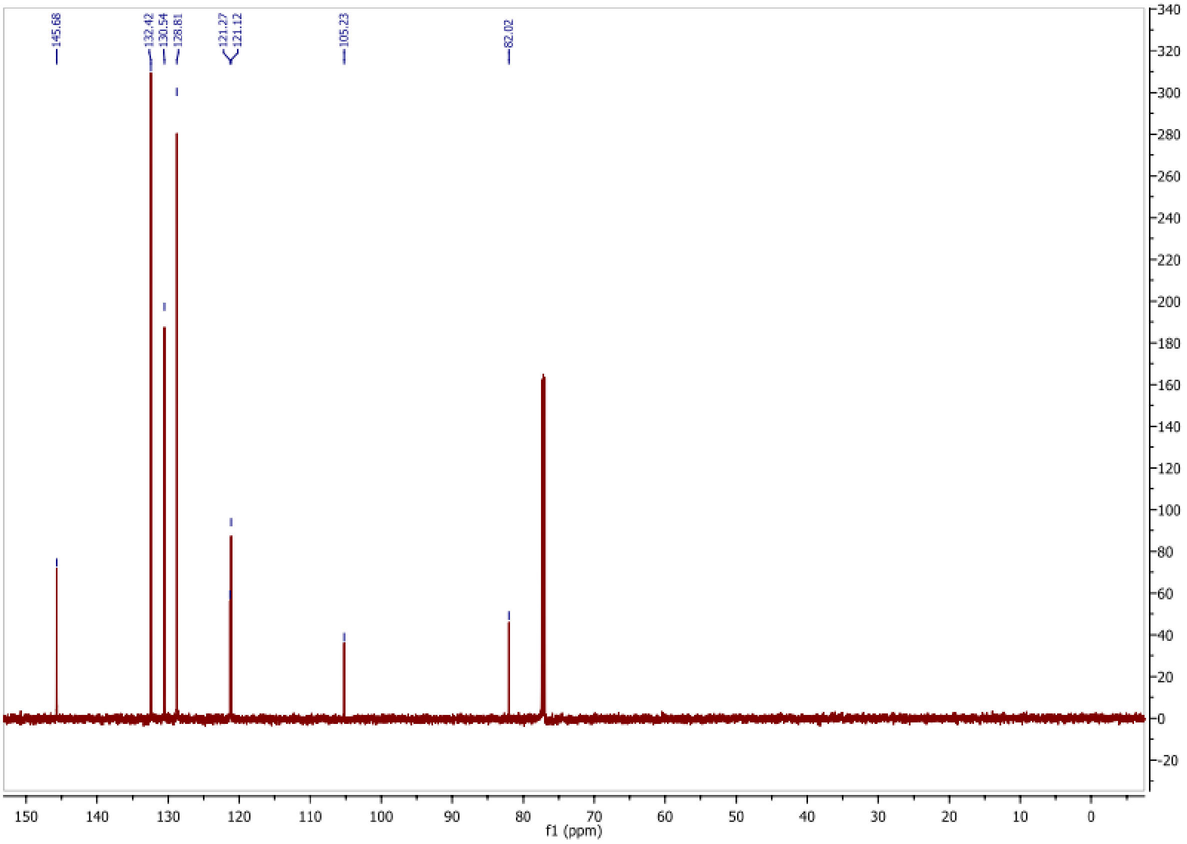

145.68
132.42
130.54
128.81
121.27
121.12
105.23
82.02
f1 (ppm)

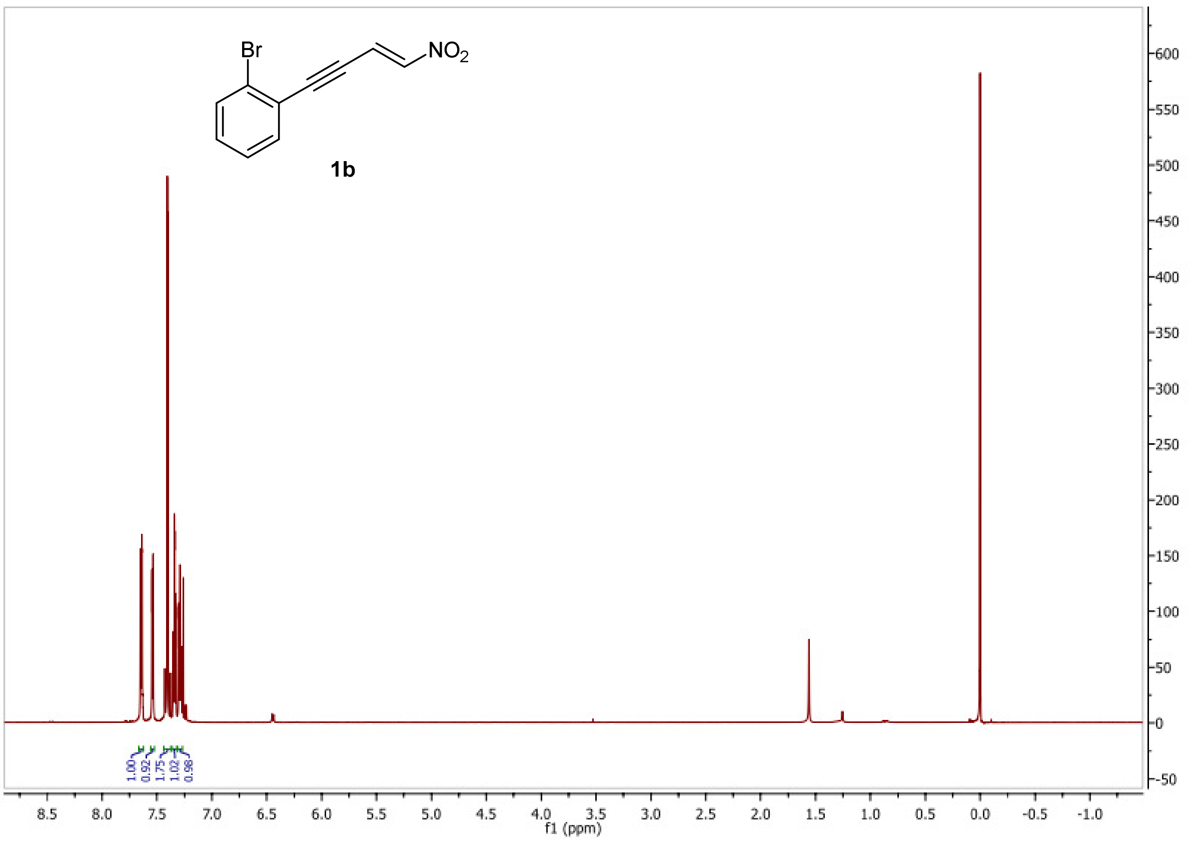

Br
NO2
1b
1.00
0.92
1.75
1.02
0.98
f1 (ppm)

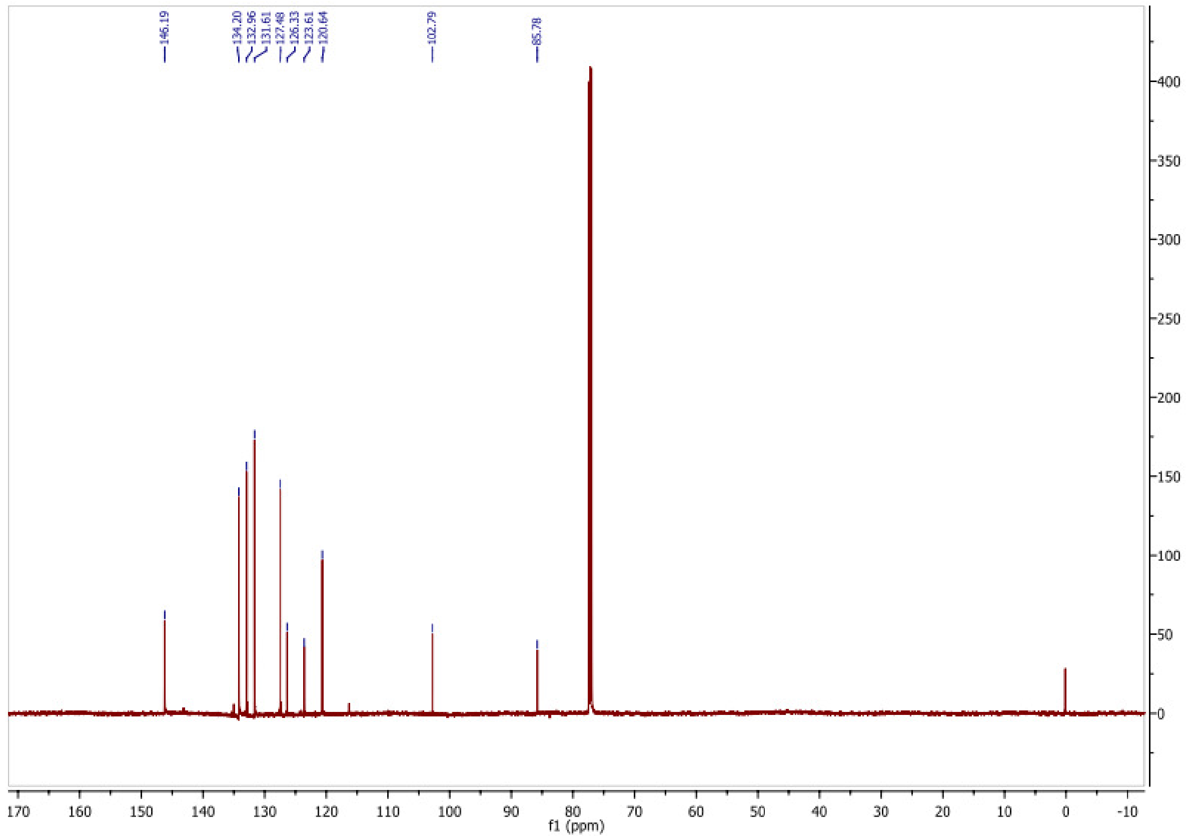

146.19
134.20
132.96
131.61
127.48
126.33
123.61
120.64
102.79
85.78
f1 (ppm)

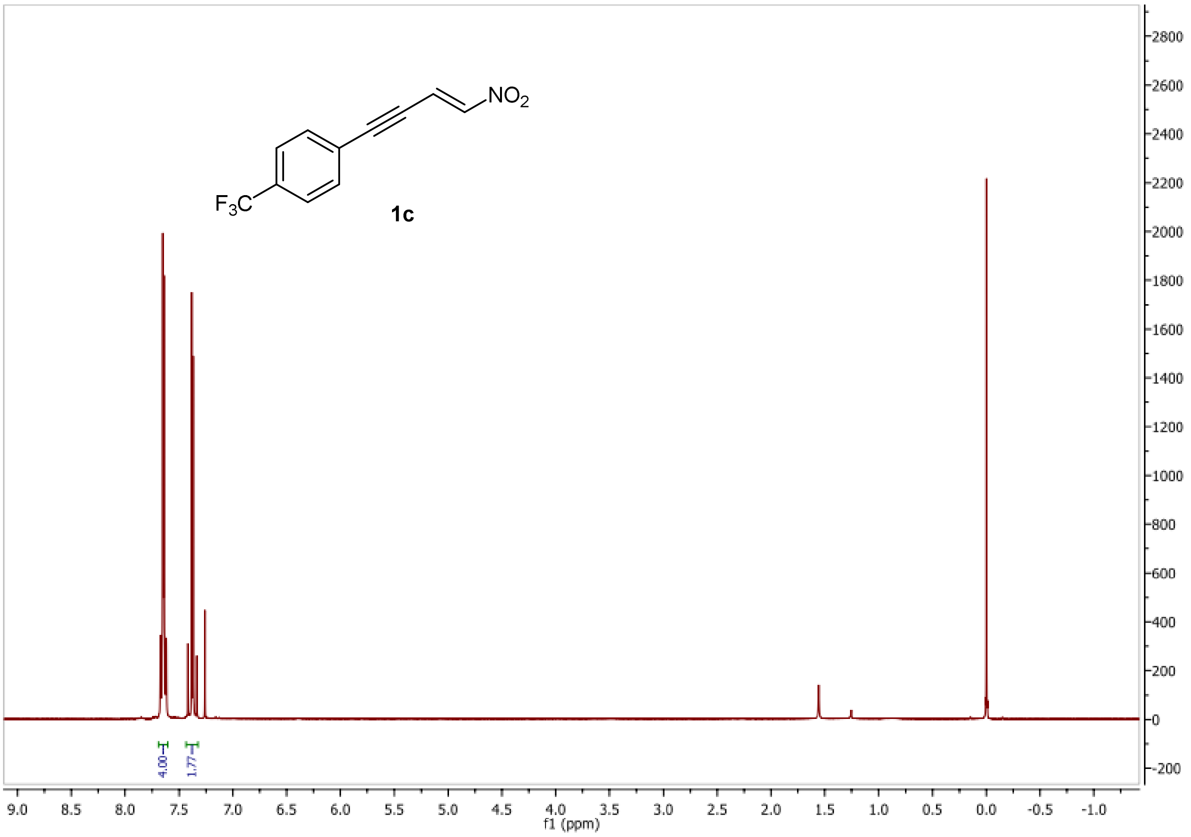
NO2
F3C
1c
4.00
1.77
f1 (ppm)

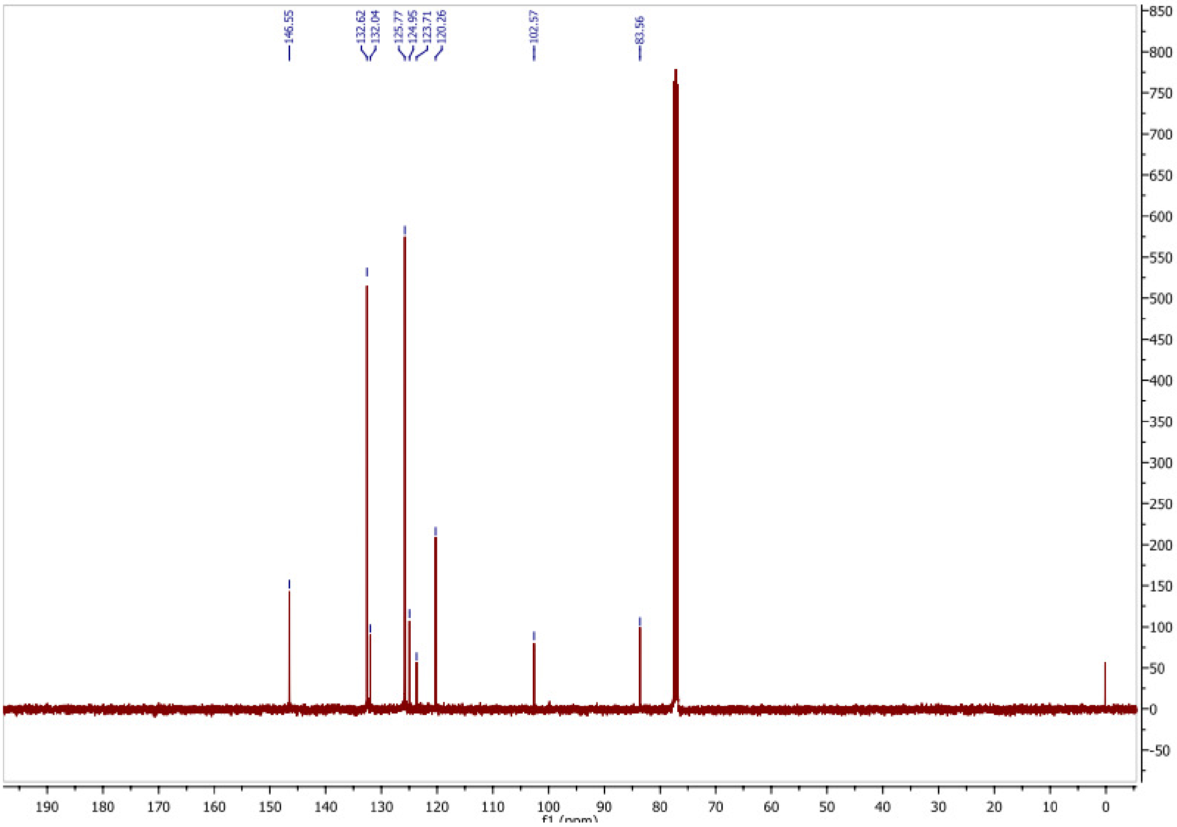
146.55
132.62
132.04
125.77
124.95
123.71
120.26
102.57
83.56

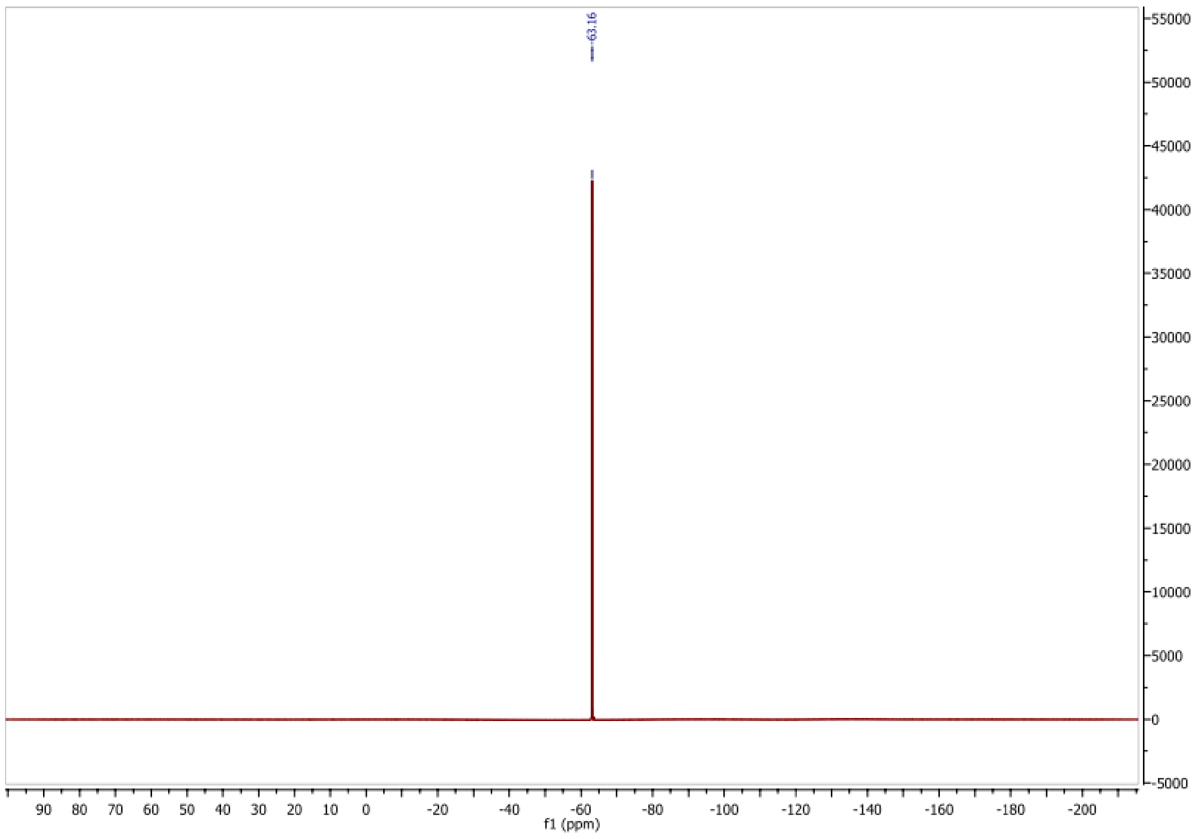
-63.16
f1 (ppm)

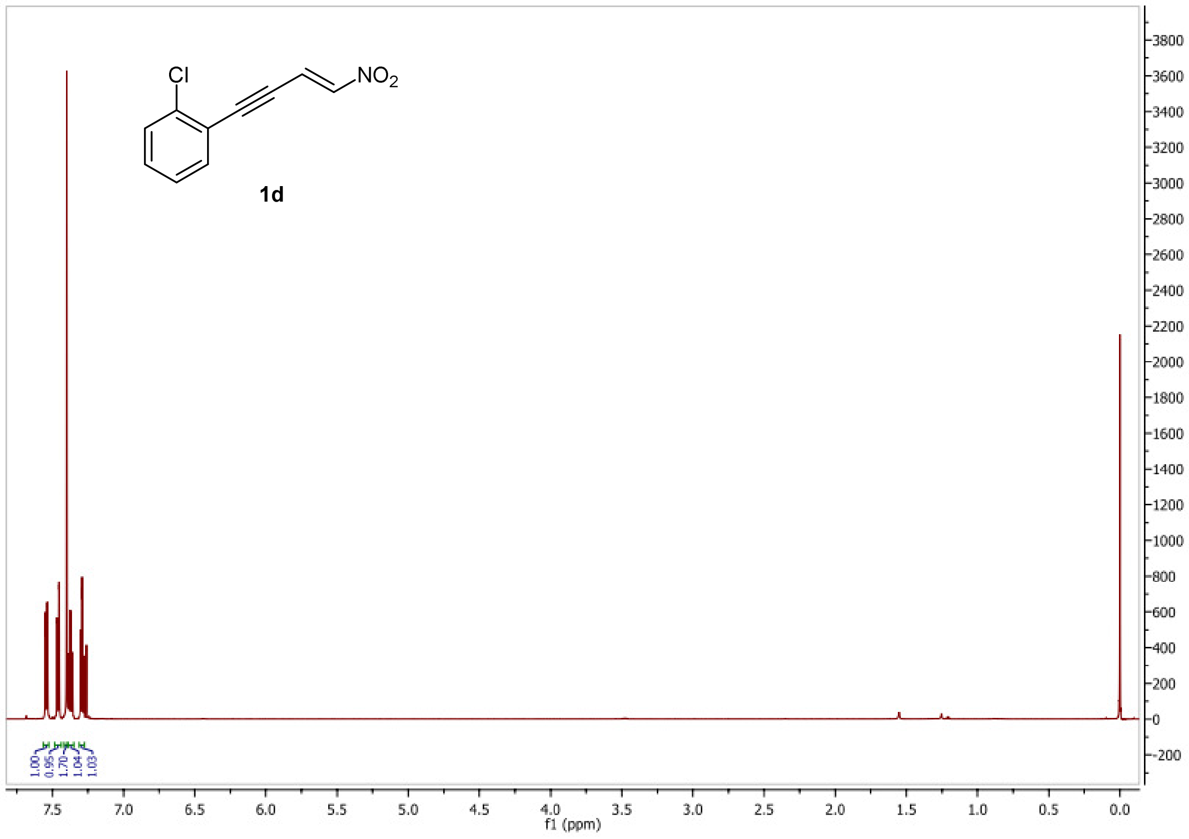
Cl
NO2
1d
1.00
0.95
1.70
1.04
1.03
f1 (ppm)

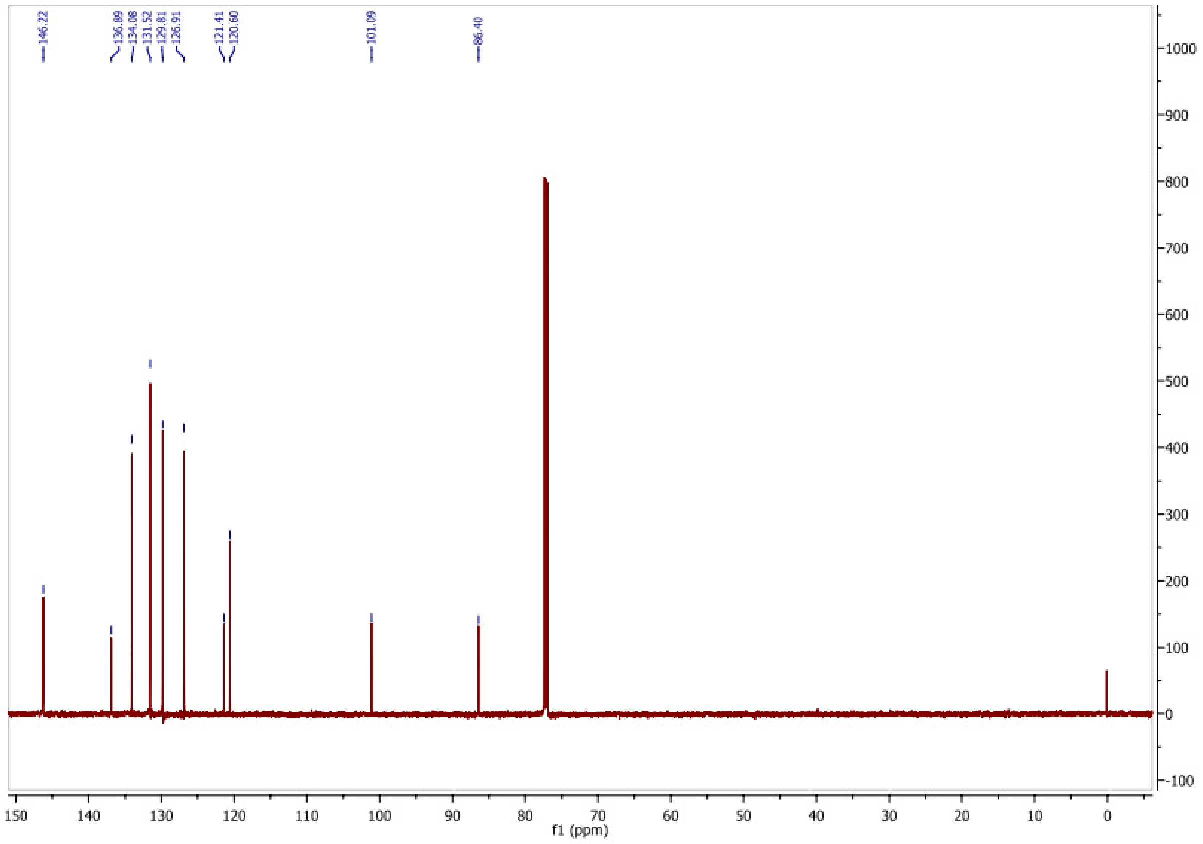
146.22
136.89
134.08
131.52
129.81
126.91
121.41
120.60
101.09
86.40
f1 (ppm)

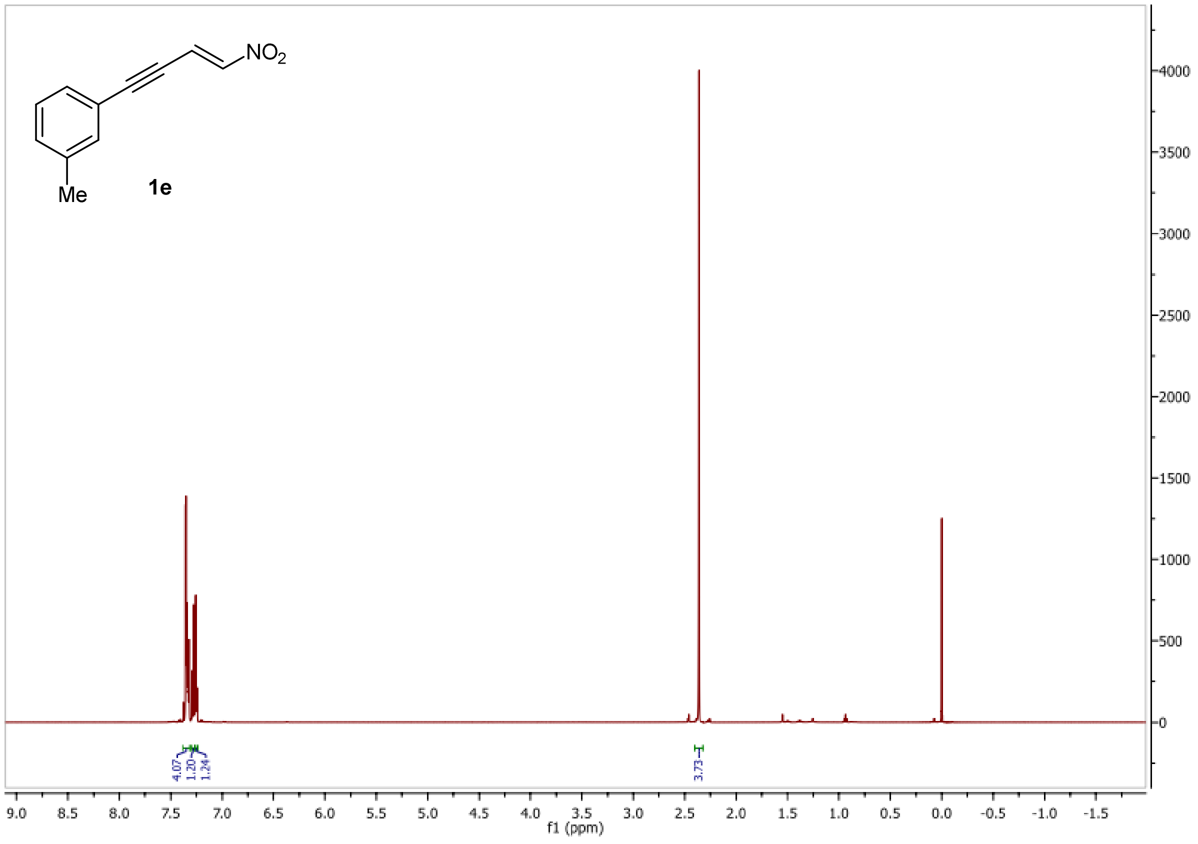
NO2
Me
1e
4.07
1.20
1.24
3.73
f1 (ppm)

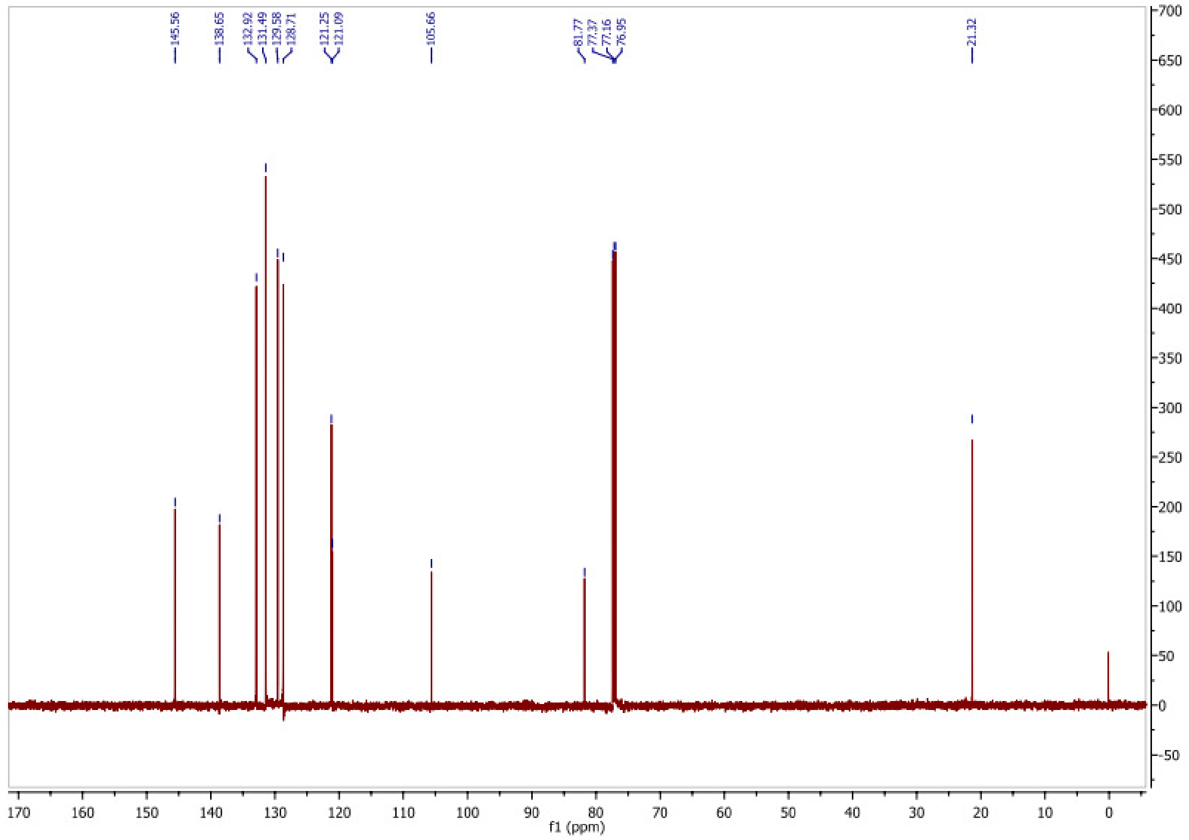
145.56
138.65
132.92
131.49
129.58
128.71
121.25
121.09
105.66
81.77
77.37
77.16
76.95
21.32
f1 (ppm)

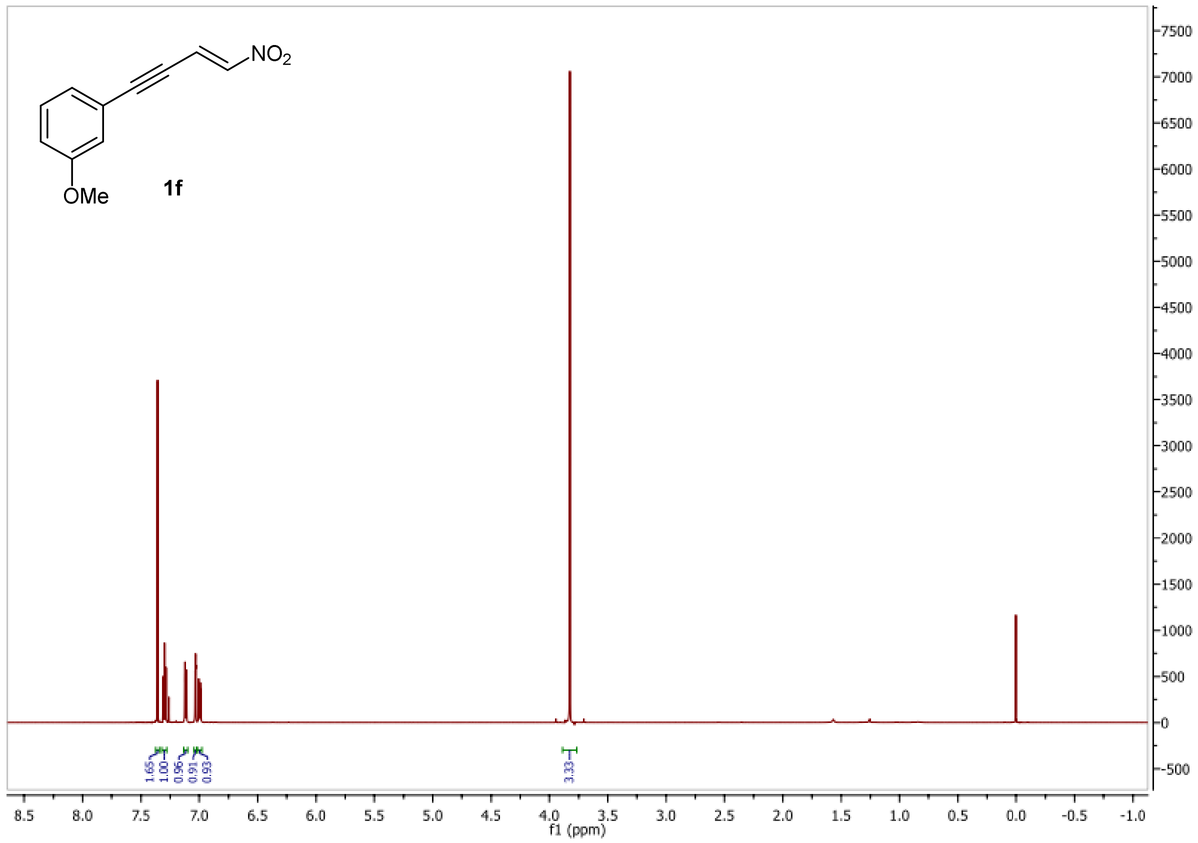
NO2
OMe
1f
1.65
1.00
0.96
0.91
0.93
3.33
f1 (ppm)

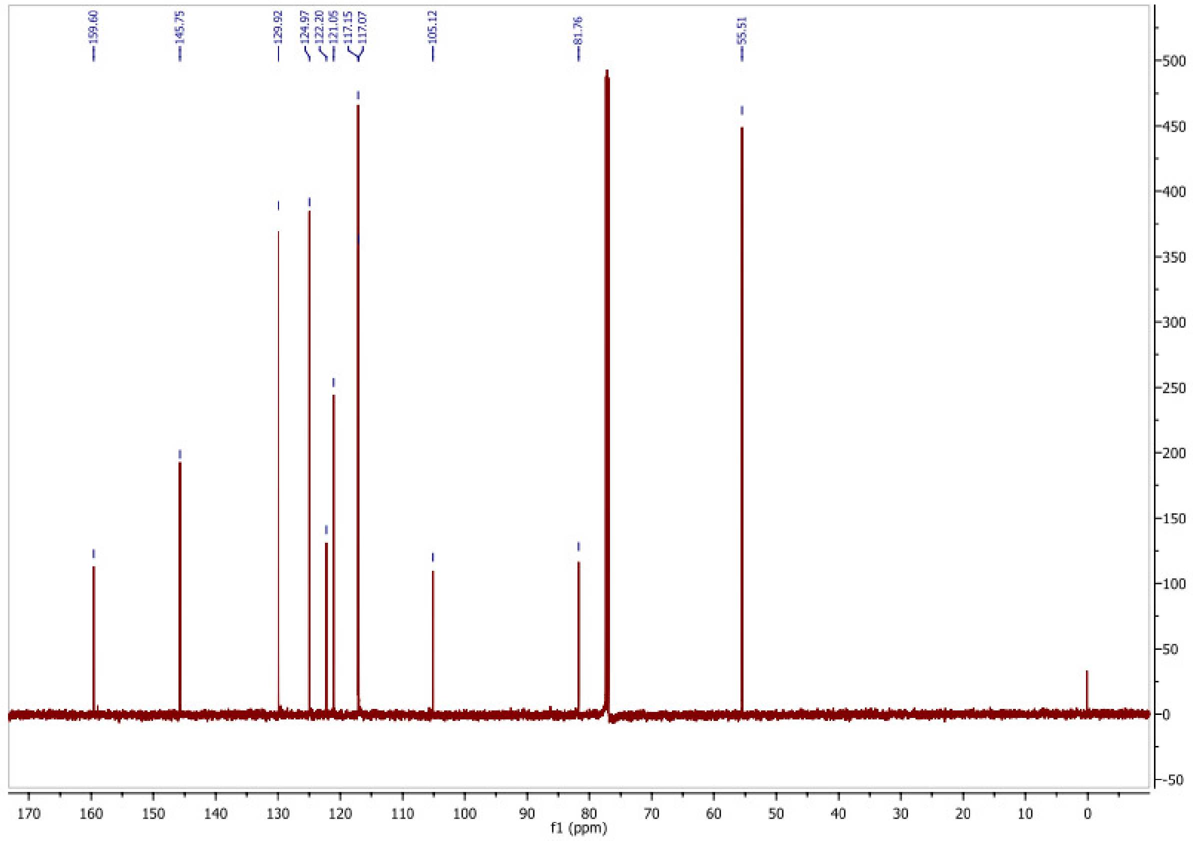

159.60
145.75
129.92
124.97
122.20
121.05
117.15
117.07
105.12
81.76
55.51
f1 (ppm)

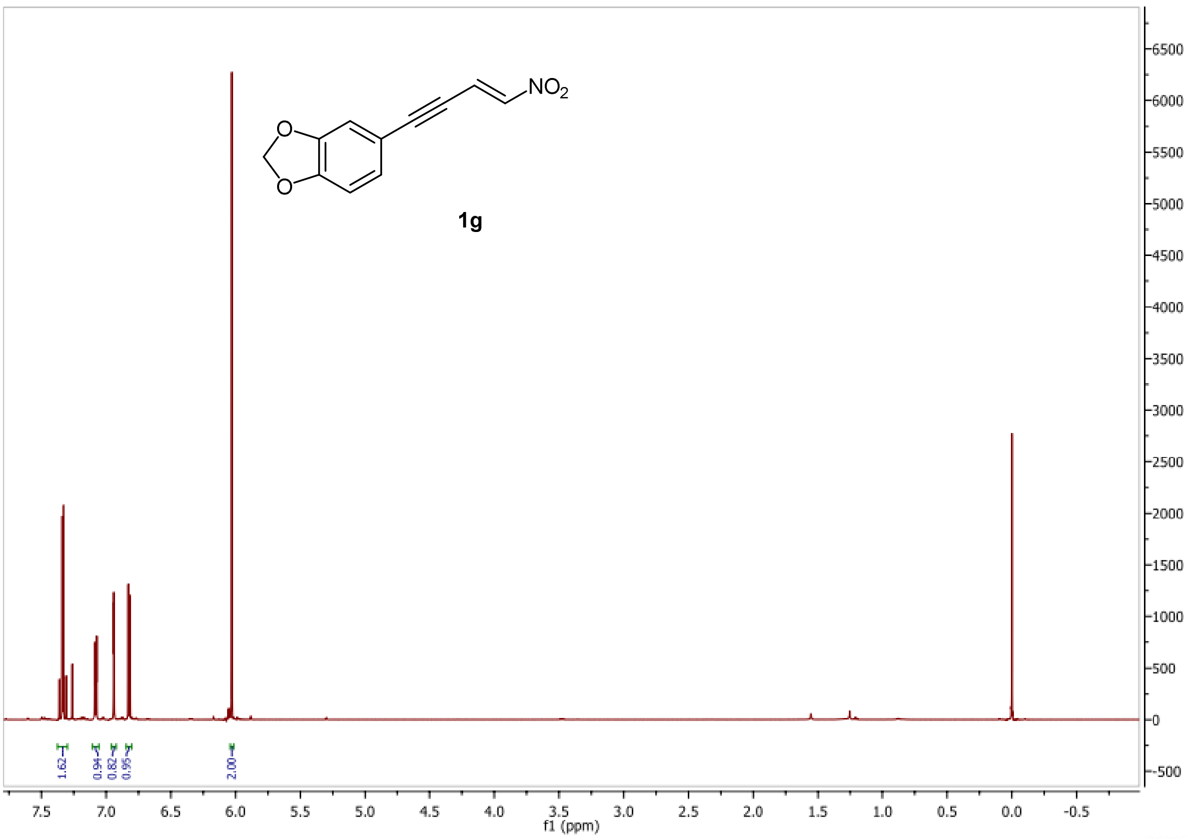
NO2
O
O
1g
1.62
0.94
0.82
0.95
2.00
f1 (ppm)

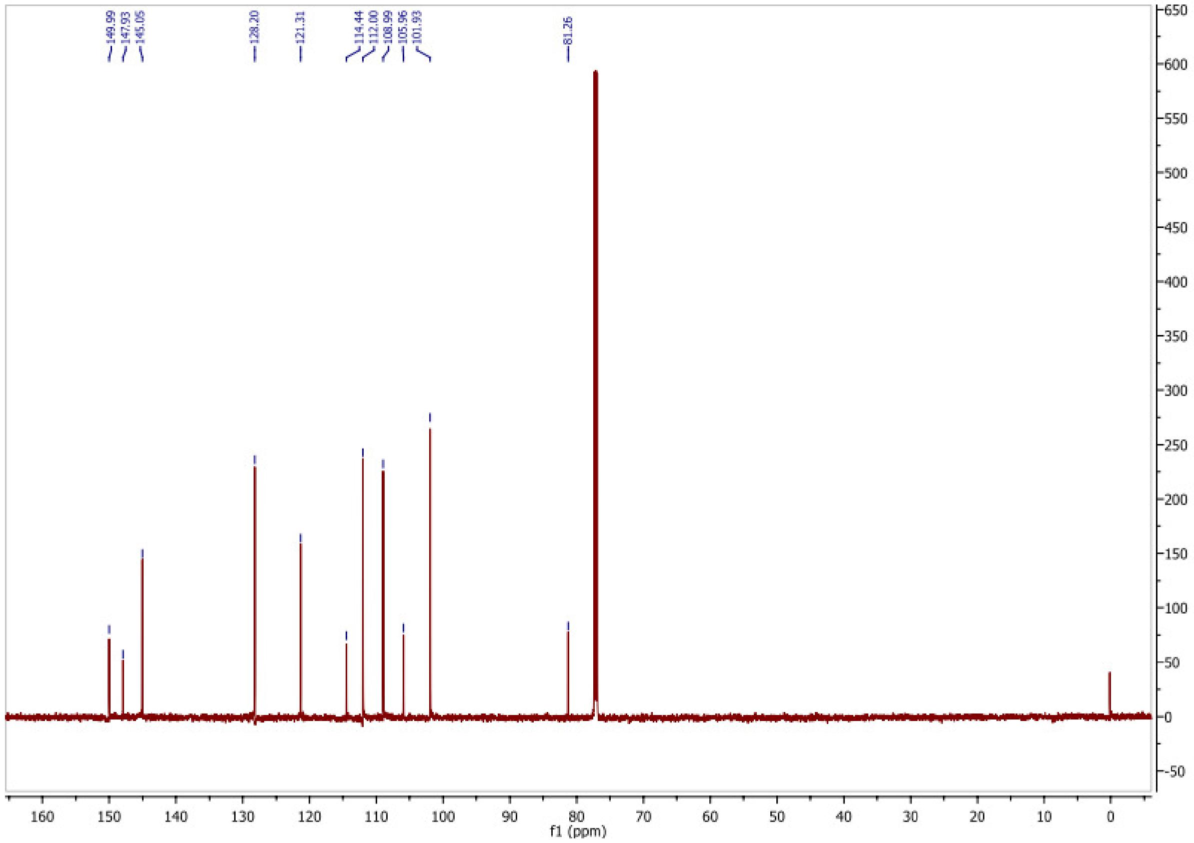
149.99
147.93
145.05
128.20
121.31
114.44
112.00
108.99
105.96
101.93
81.26
f1 (ppm)

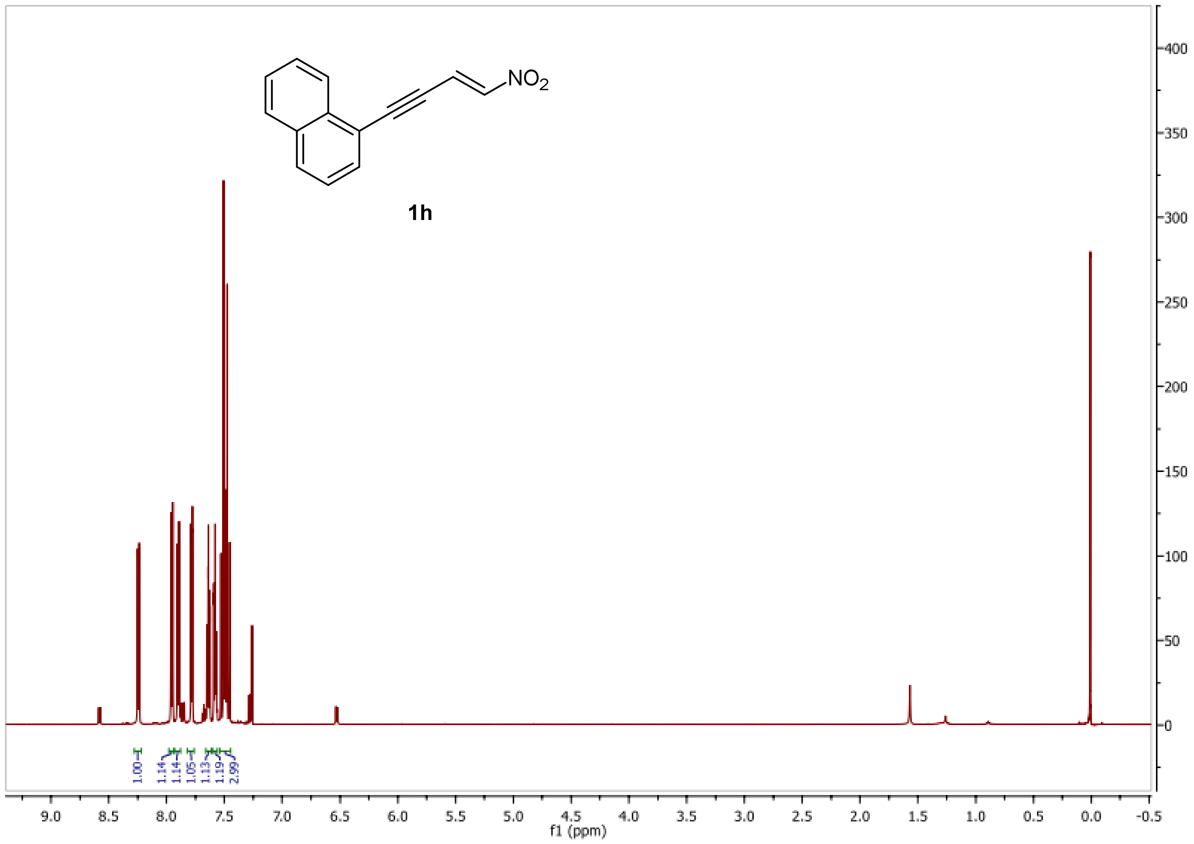

NO2
1h
1.00
1.14
1.14
1.05
1.13
1.19
2.99
f1 (ppm)

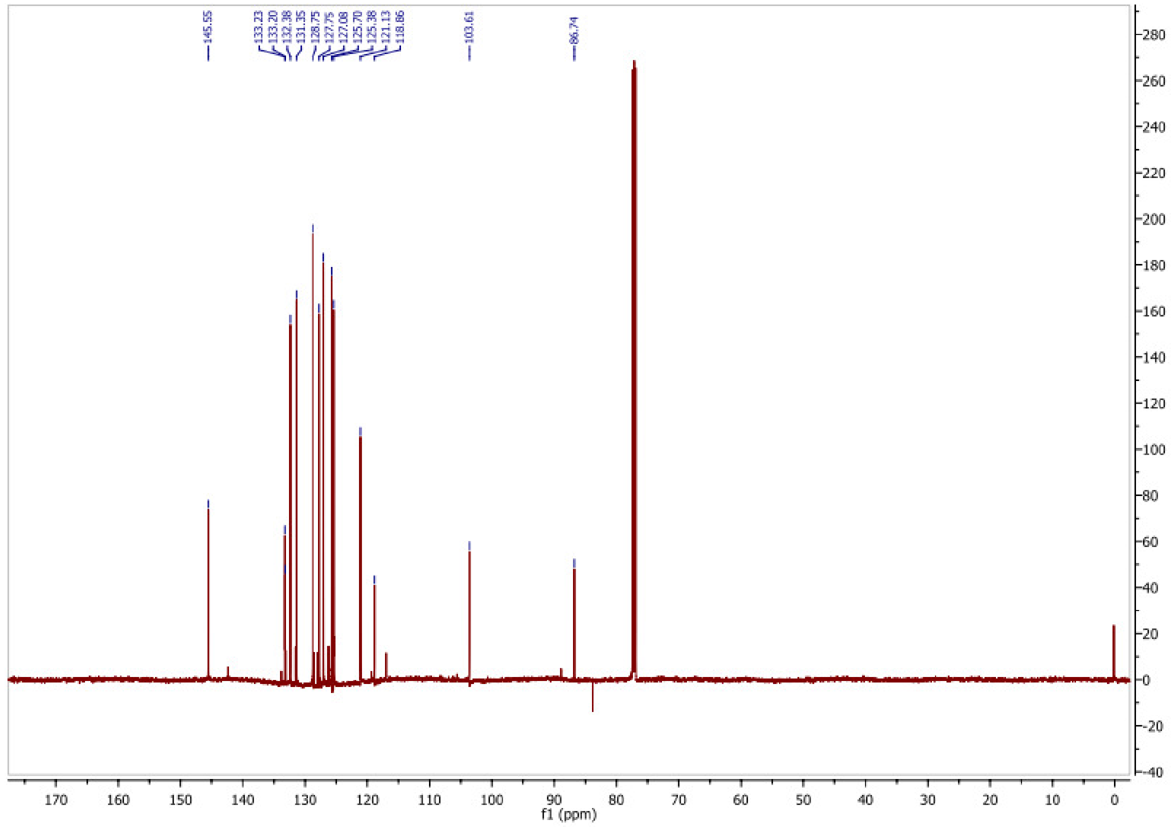

145.55
133.23
133.20
132.38
131.35
128.75
127.75
127.08
125.70
125.38
121.13
118.86
103.61
86.74
f1 (ppm)

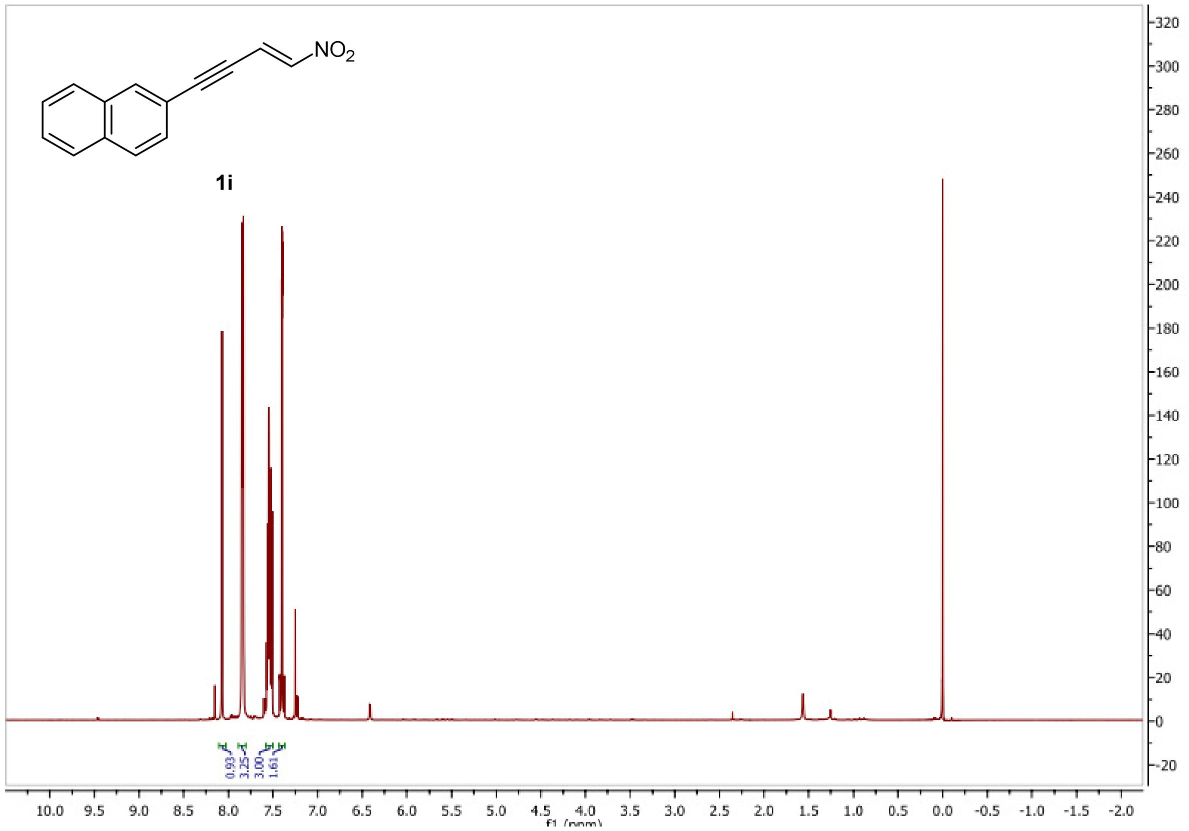

NO2
1i
0.93
3.25
3.00
1.61
f1 (ppm)

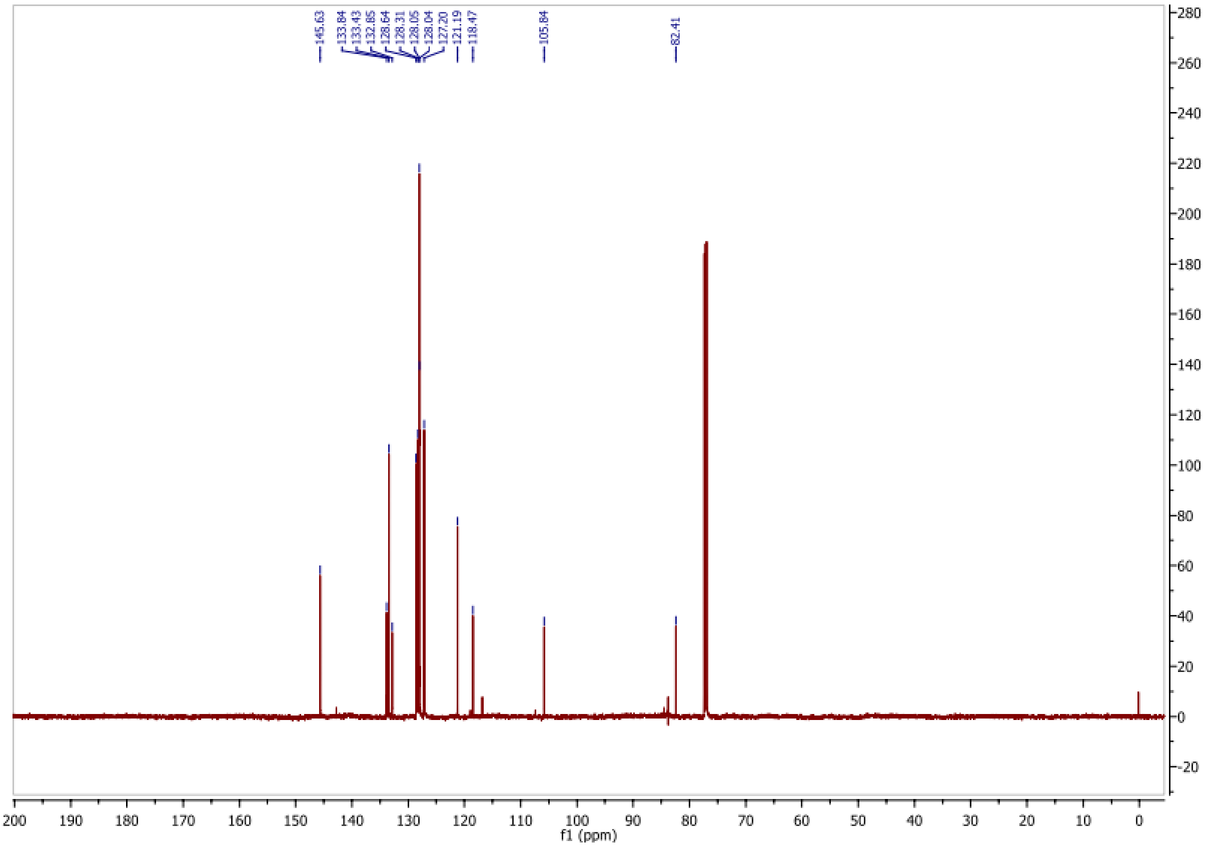

145.63
133.84
133.43
132.85
128.64
128.31
128.05
128.04
127.20
121.19
118.47
105.84
82.41
f1 (ppm)

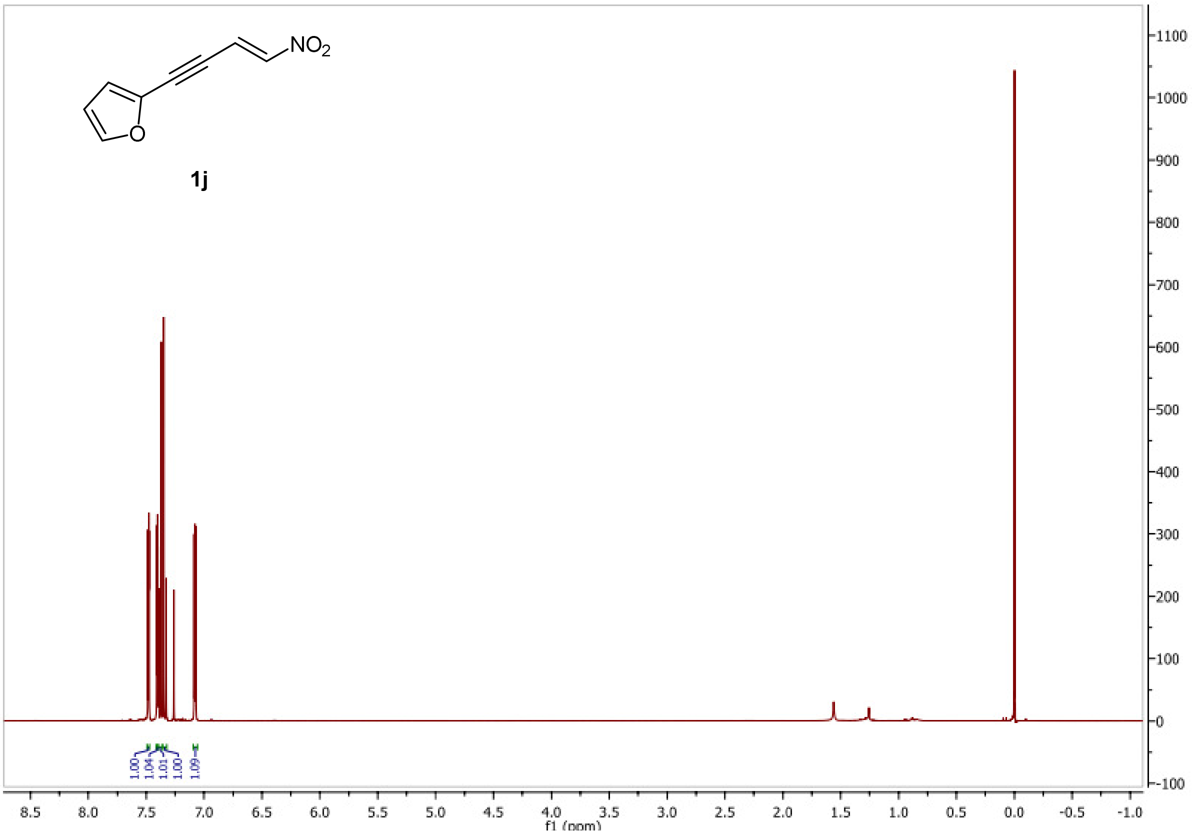

NO2
O
1j
1.00
1.04
1.01
1.00
1.09
f1 (ppm)

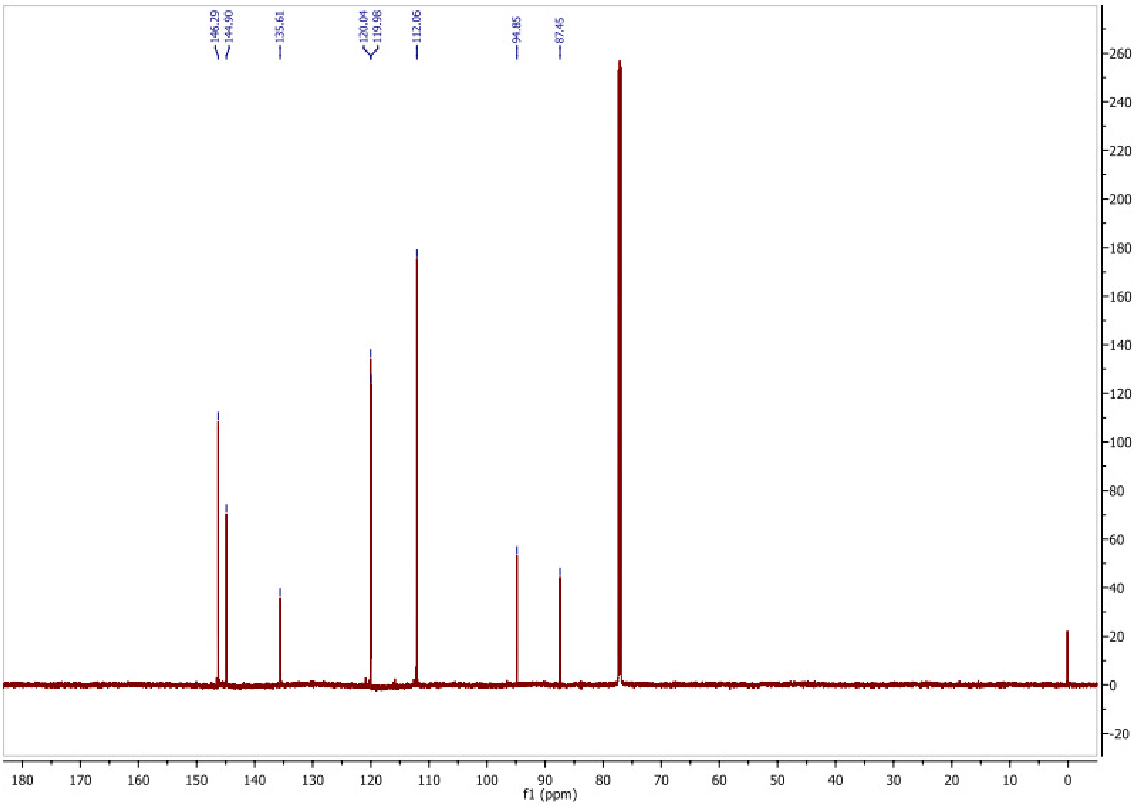

146.29
144.90
135.61
120.04
119.98
112.06
94.85
87.45
f1 (ppm)

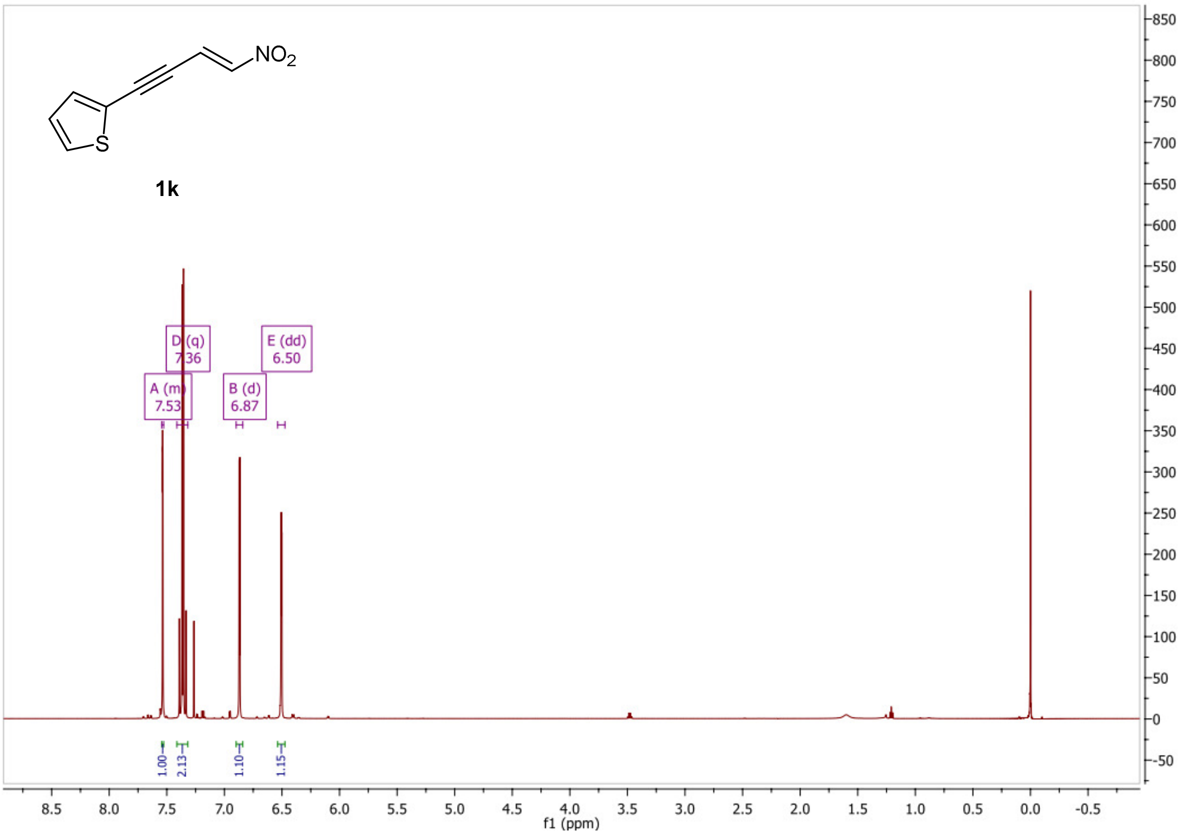
NO2
S
1k
A (m)
7.53
D (q)
7.36
B (d)
6.87
E (dd)
6.50
1.00
2.13
1.10
1.15
f1 (ppm)

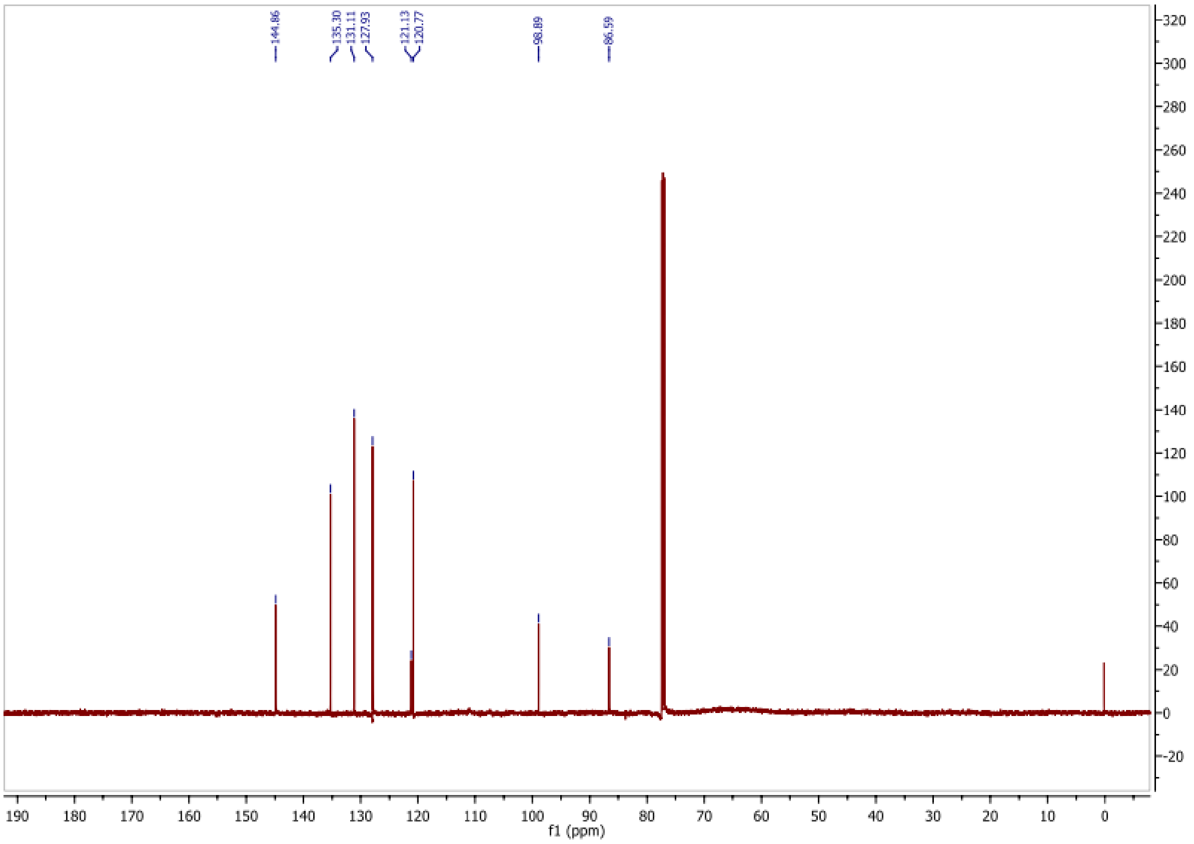
144.86
135.30
131.11
127.93
121.13
120.77
98.89
86.59
f1 (ppm)

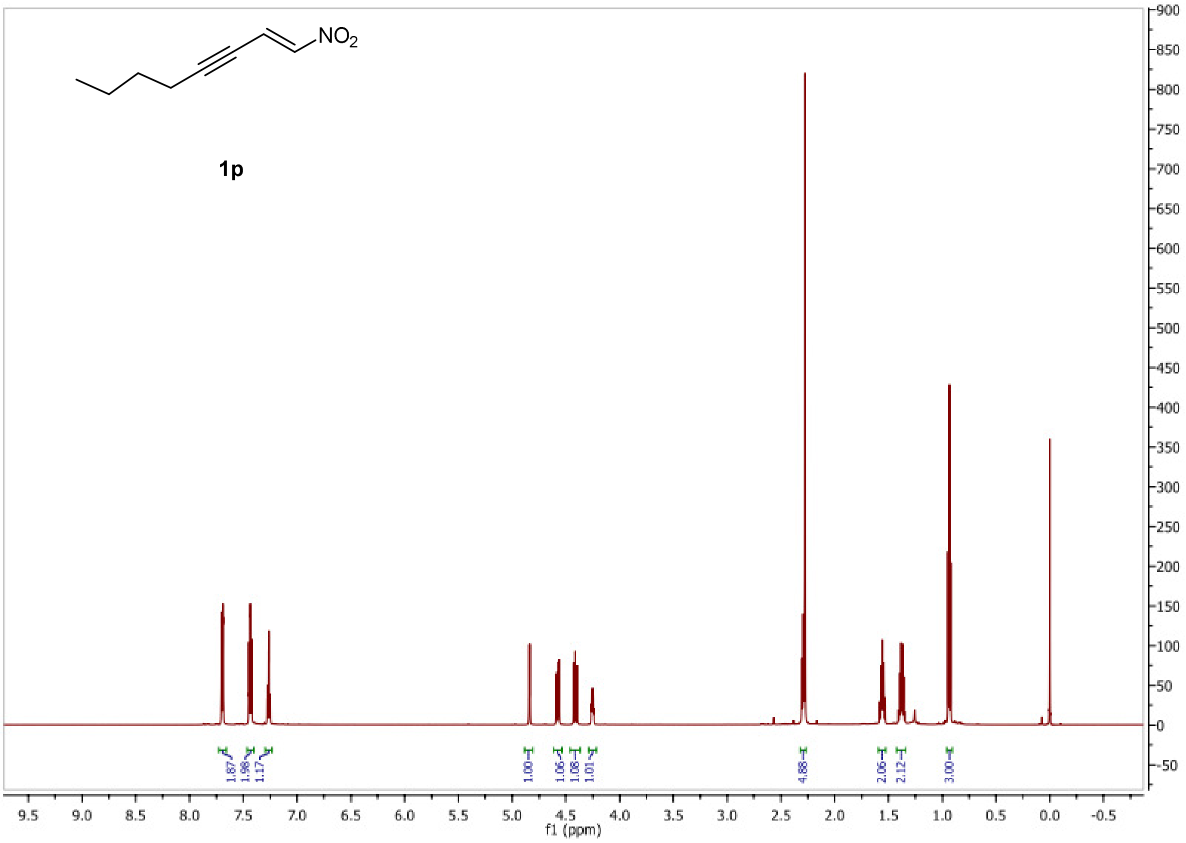
NO2
1p
1.87
1.98
1.17
1.00
1.06
1.08
1.01
4.88
2.06
2.12
3.00
f1 (ppm)

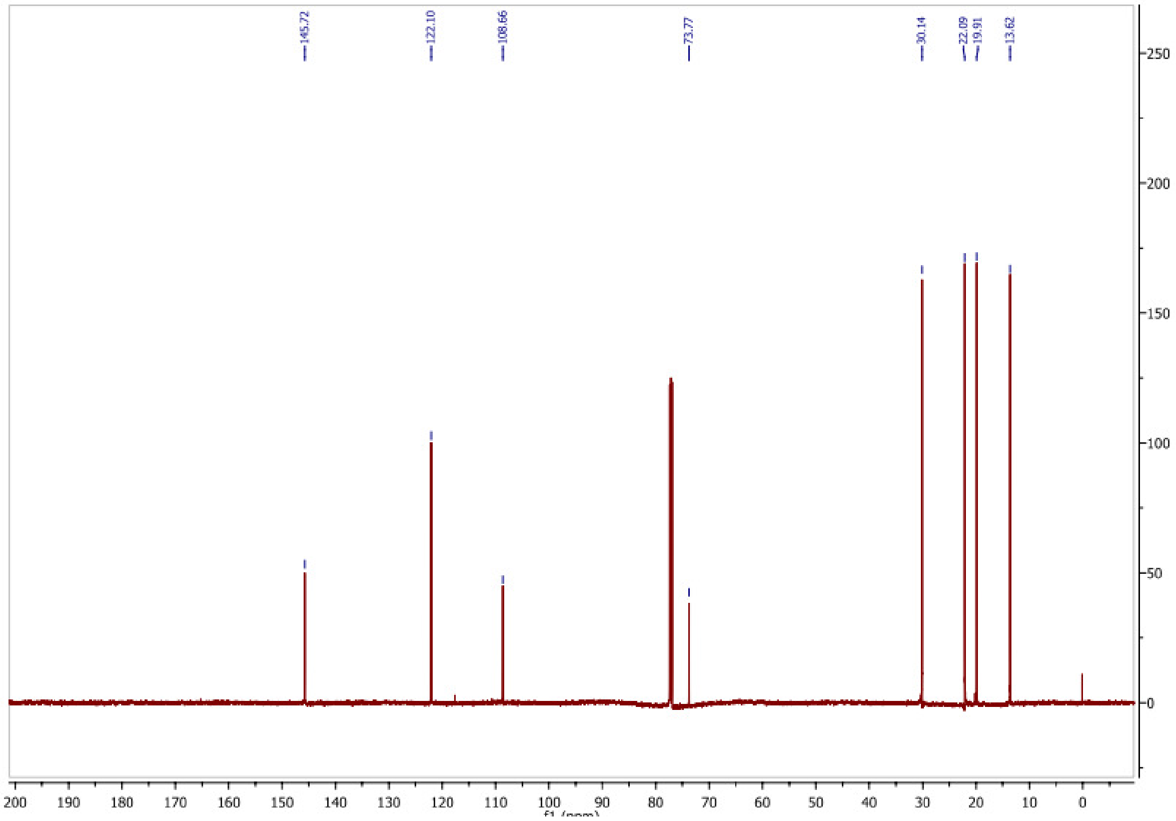
145.72
122.10
108.66
73.77
30.14
22.09
19.91
13.62
f1 (ppm)

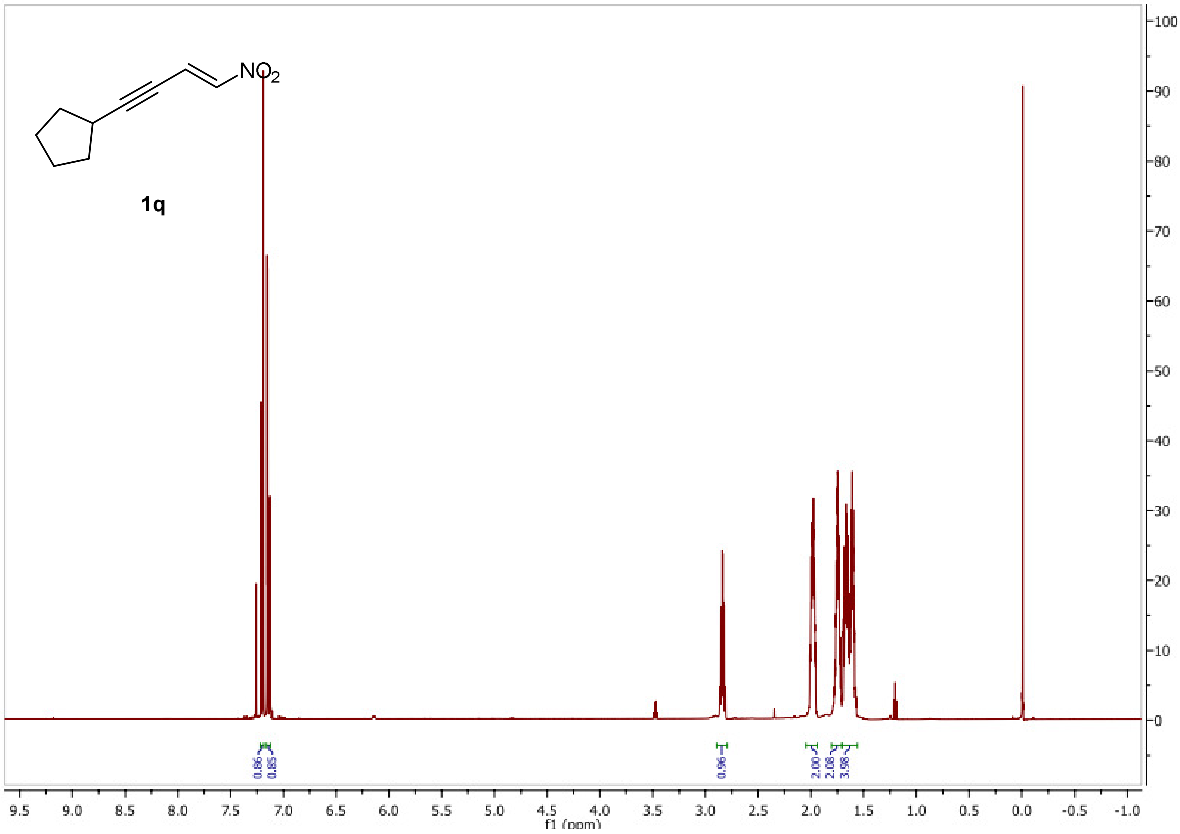

NO2
1q
0.86
0.85
0.96
2.00
2.08
3.98
f1 (ppm)

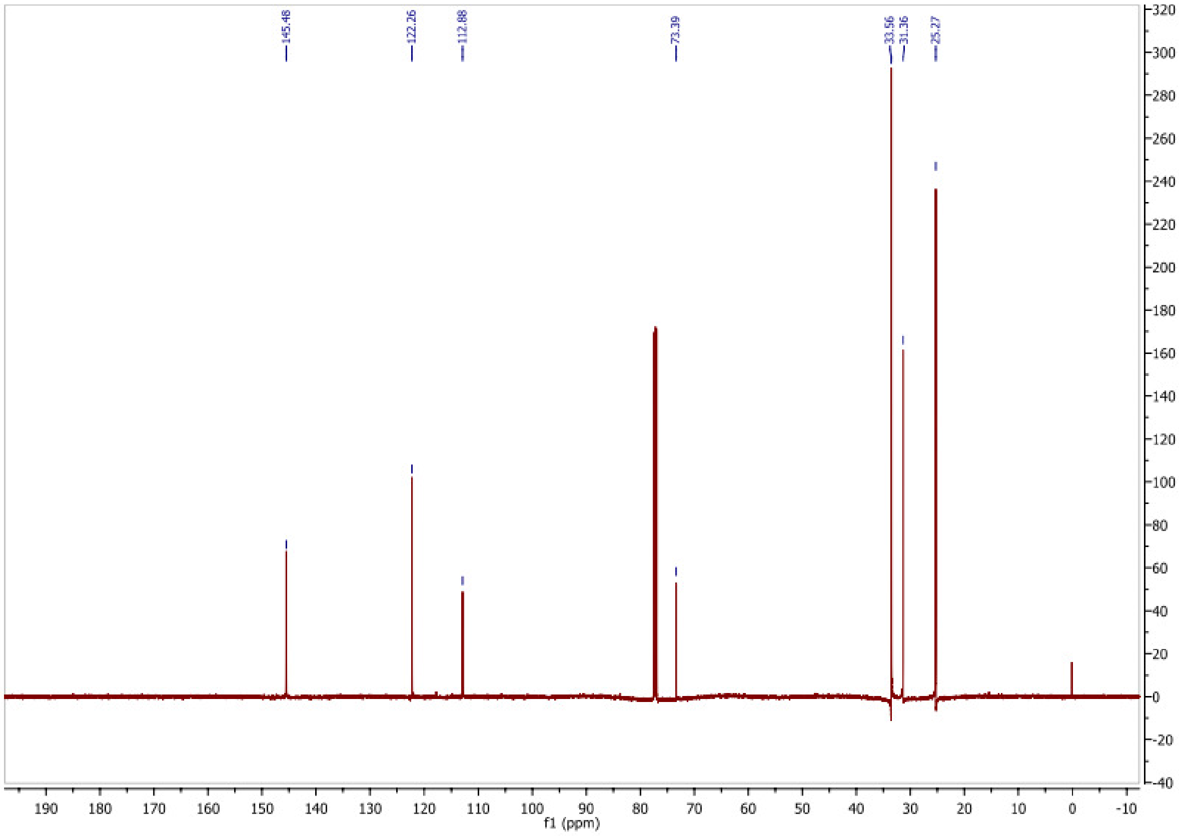

145.48
122.26
112.88
73.39
33.56
31.36
25.27
f1 (ppm)

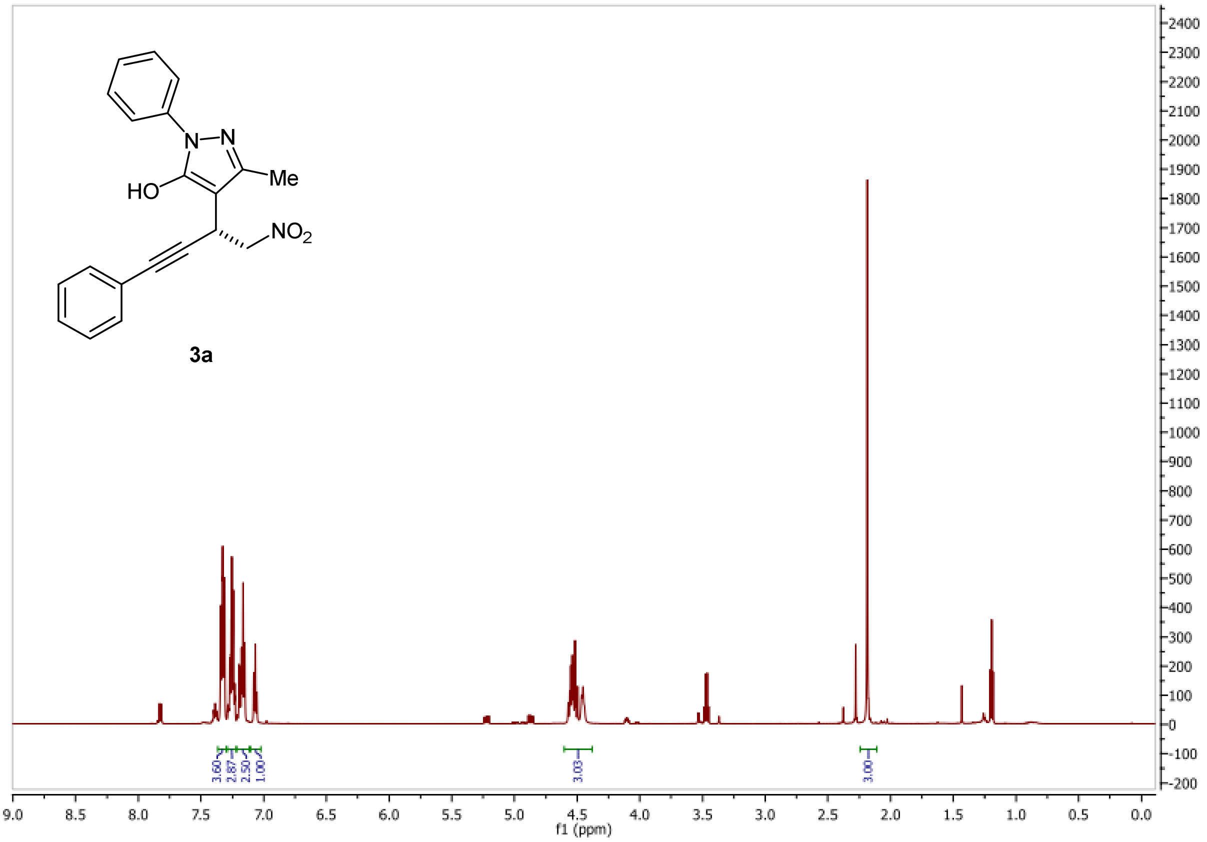
N
N
HO
Me
NO2
3a
3.60
2.87
2.50
1.00
3.03
3.00
f1 (ppm)

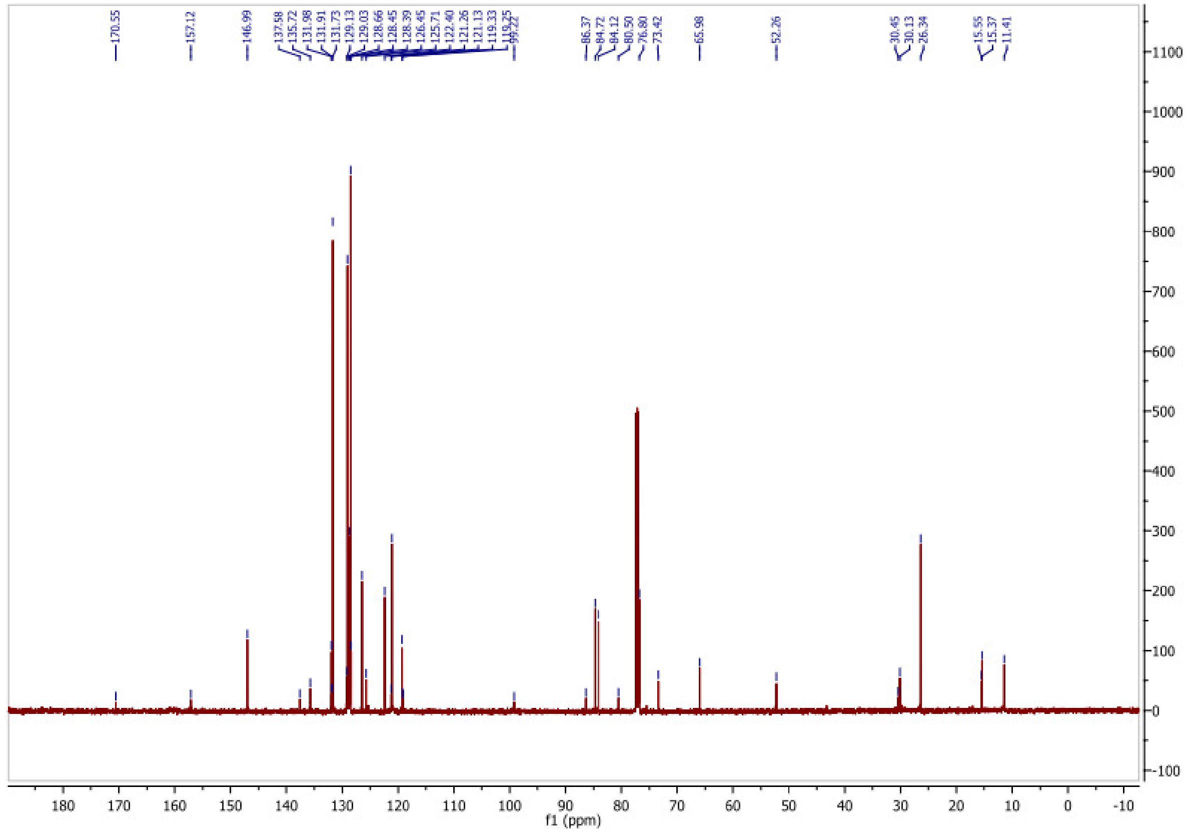
170.55
157.12
146.99
137.58
135.72
131.98
131.91
131.73
129.13
129.03
128.66
128.45
128.39
126.45
125.71
122.40
121.26
121.13
119.33
119.25
99.22
86.37
84.72
84.12
80.50
76.80
73.42
65.98
52.26
30.45
30.13
26.34
15.55
15.37
11.41
f1 (ppm)

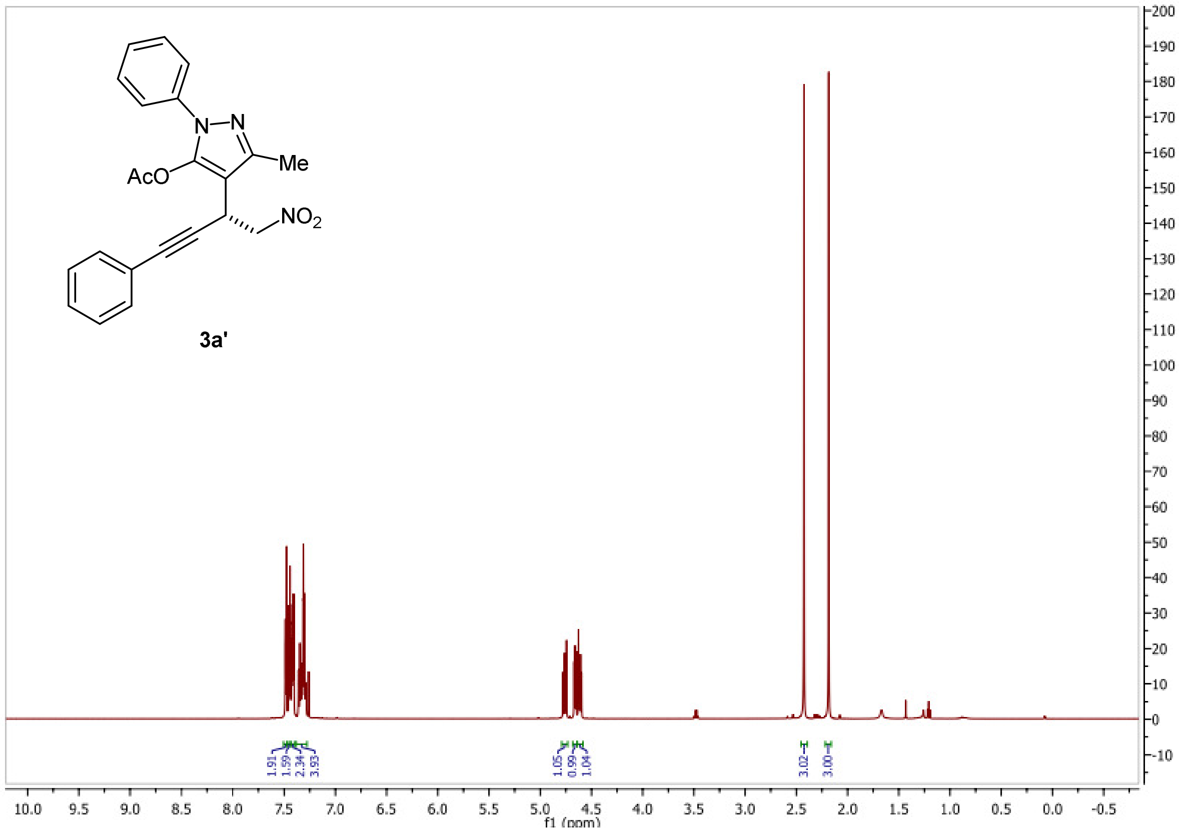
AcO
N
N
Me
NO2
3a'
1.91
1.59
2.34
3.93
1.05
0.99
1.04
3.02
3.00
f1 (ppm)

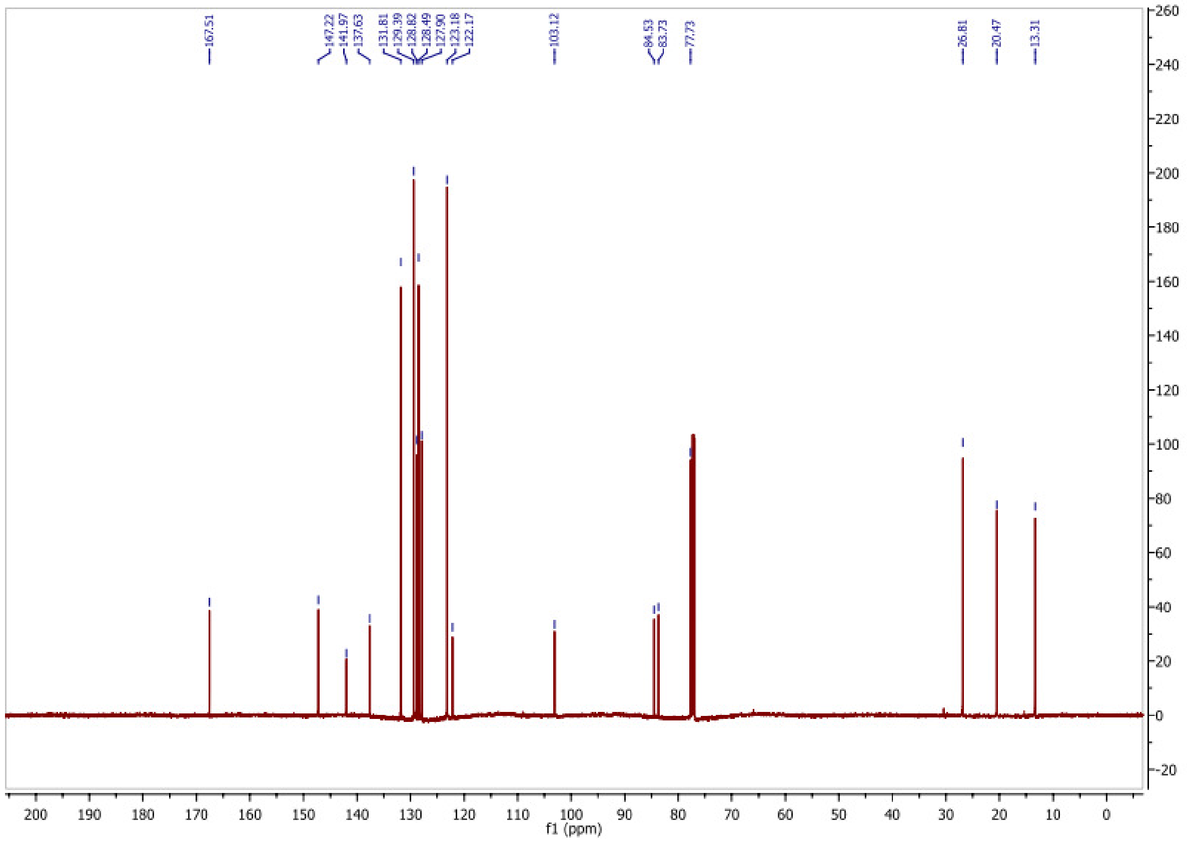
167.51
147.22
141.97
137.63
131.81
129.39
128.82
128.49
127.90
123.18
122.17
103.12
84.53
83.73
77.73
26.81
20.47
13.31
f1 (ppm)

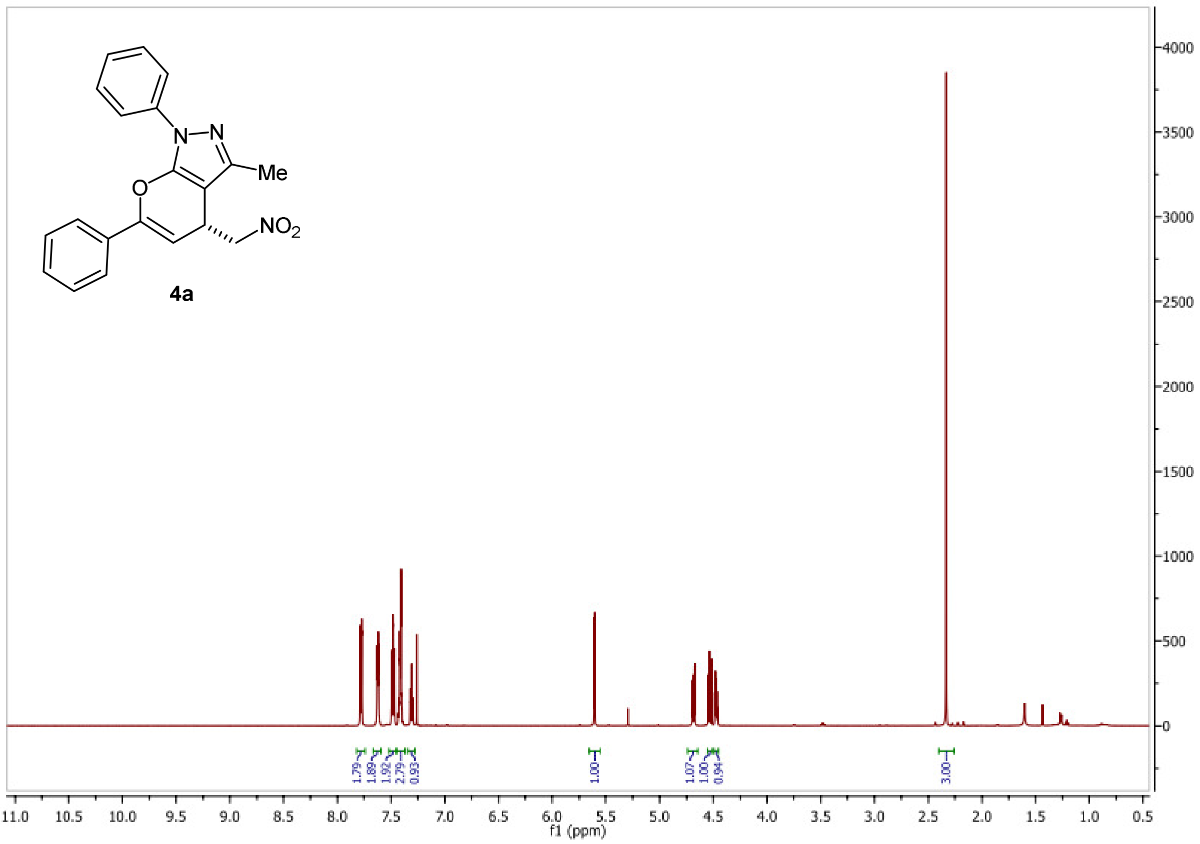

N
N
Me
O
NO2
4a
1.79
1.89
1.92
2.79
0.93
1.00
1.07
1.00
0.94
3.00
f1 (ppm)

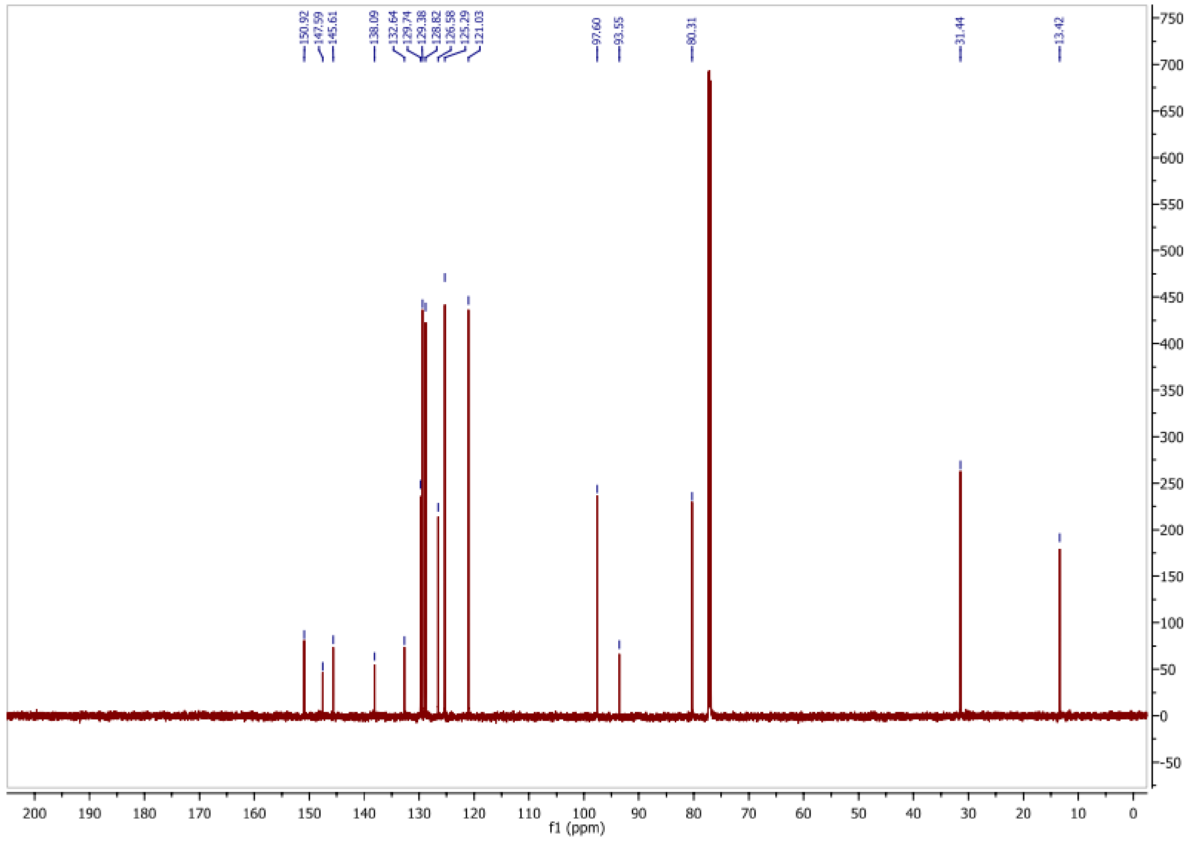

150.92
147.59
145.61
138.09
132.64
129.74
129.38
128.82
126.58
125.29
121.03
97.60
93.55
80.31
31.44
13.42
f1 (ppm)

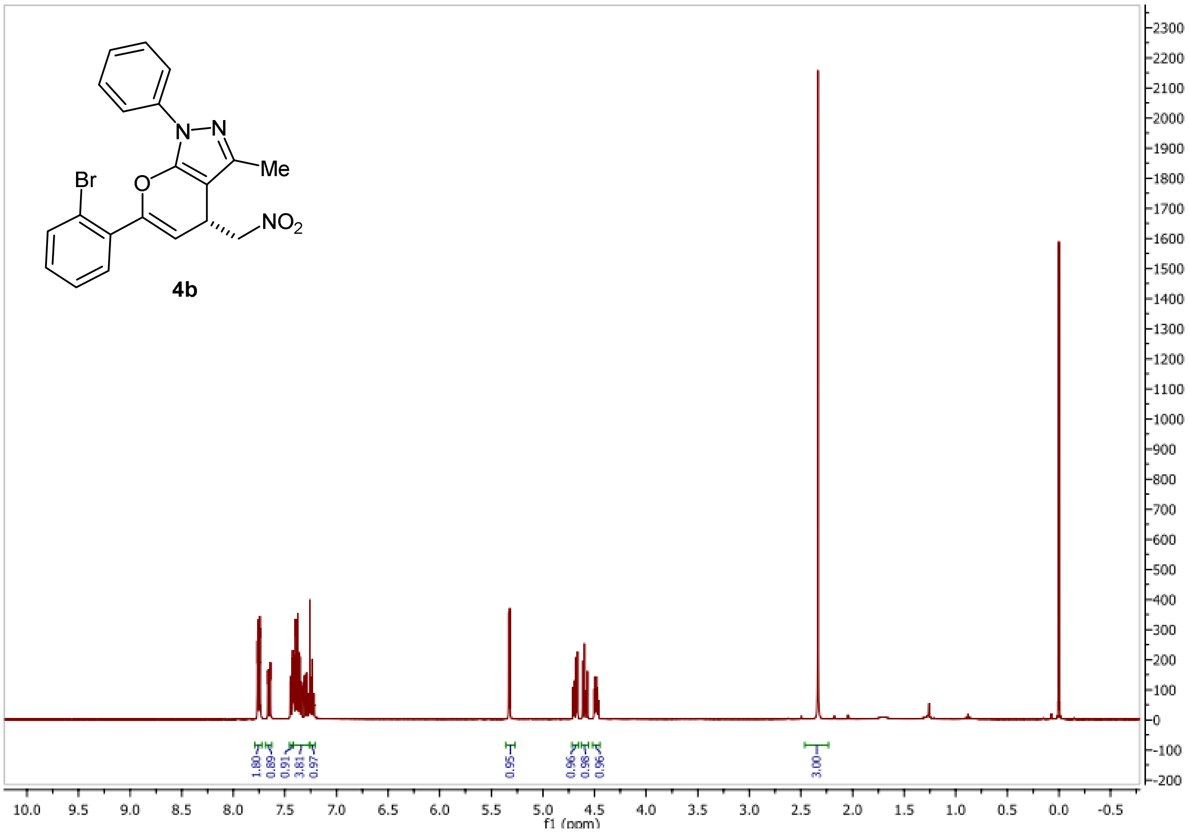
Br
N
N
O
Me
NO2
4b
1.80
0.89
0.91
3.81
0.97
0.95
0.96
0.98
0.96
3.00
f1 (ppm)

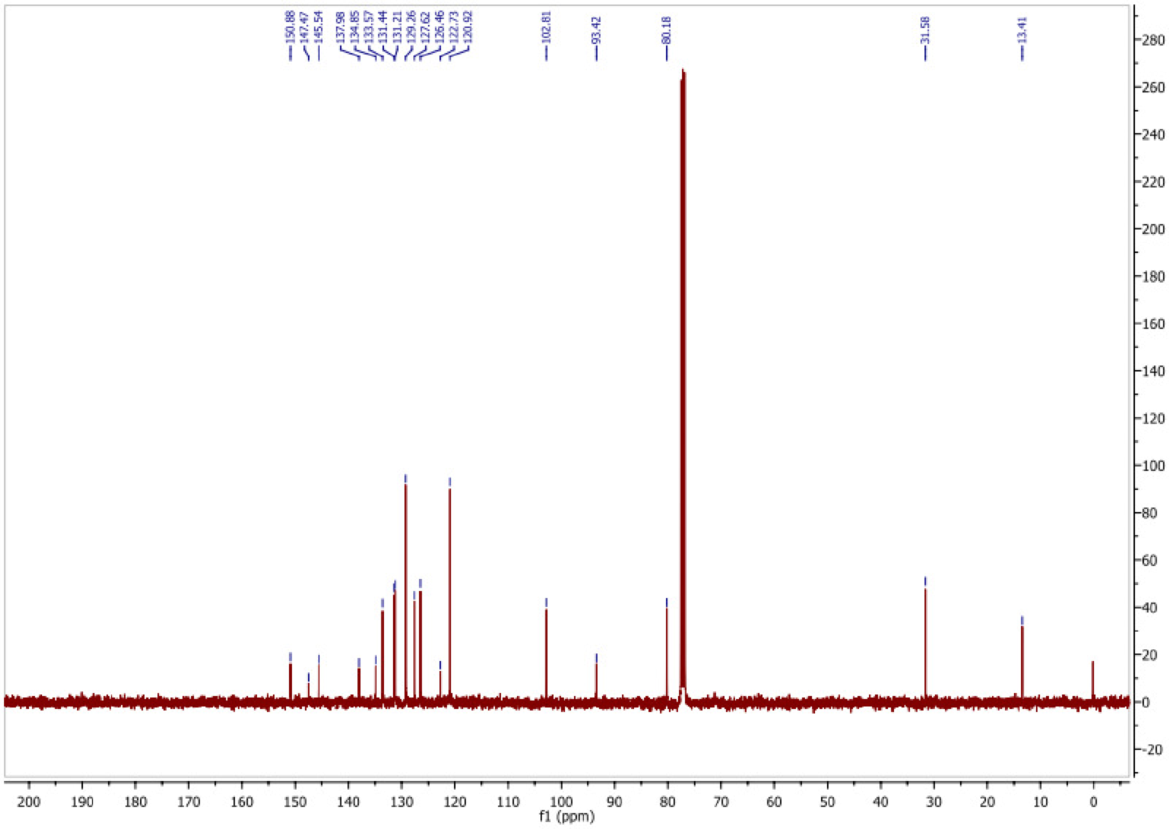
150.88
147.47
145.54
137.98
134.85
133.57
131.44
131.21
129.26
127.62
126.46
122.73
120.92
102.81
93.42
80.18
31.58
13.41
f1 (ppm)

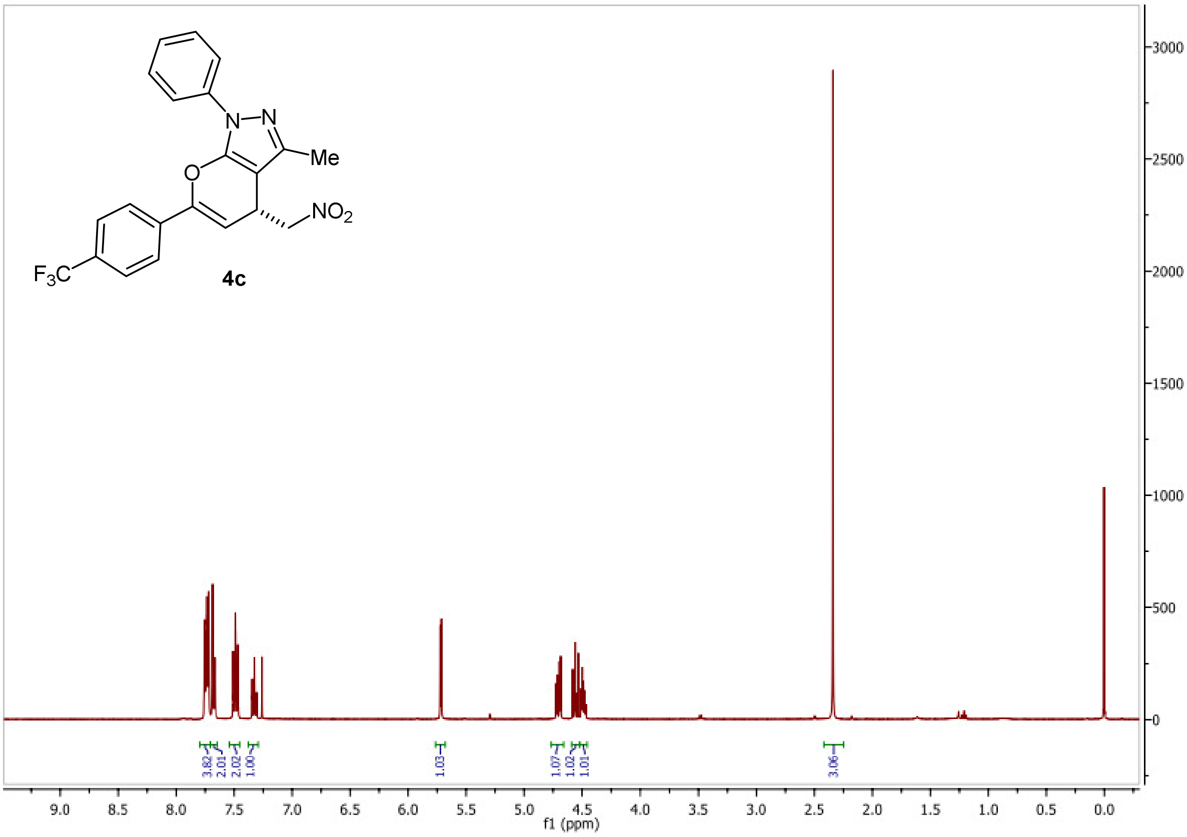
N–N
Me
O
NO2
F3C
4c
3.82
2.01
2.02
1.00
1.03
1.07
1.02
1.01
3.06
9.0 8.5 8.0 7.5 7.0 6.5 6.0 5.5 5.0 4.5 4.0 3.5 3.0 2.5 2.0 1.5 1.0 0.5 0.0
f1 (ppm)
3000 2500 2000 1500 1000 500 0

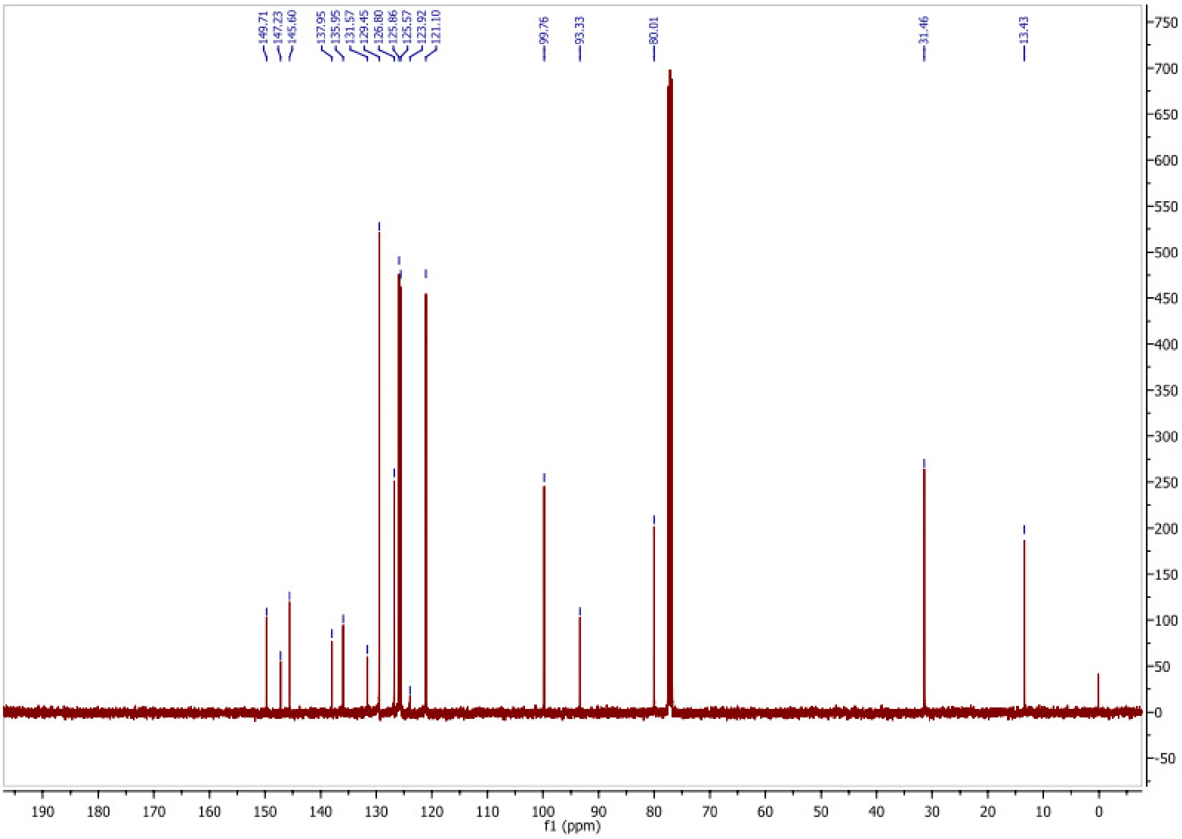
149.71
147.23
145.60
137.95
135.95
131.57
129.45
126.80
125.86
125.57
123.92
121.10
99.76
93.33
80.01
31.46
13.43
190 180 170 160 150 140 130 120 110 100 90 80 70 60 50 40 30 20 10 0
f1 (ppm)
750 700 650 600 550 500 450 400 350 300 250 200 150 100 50 0 -50

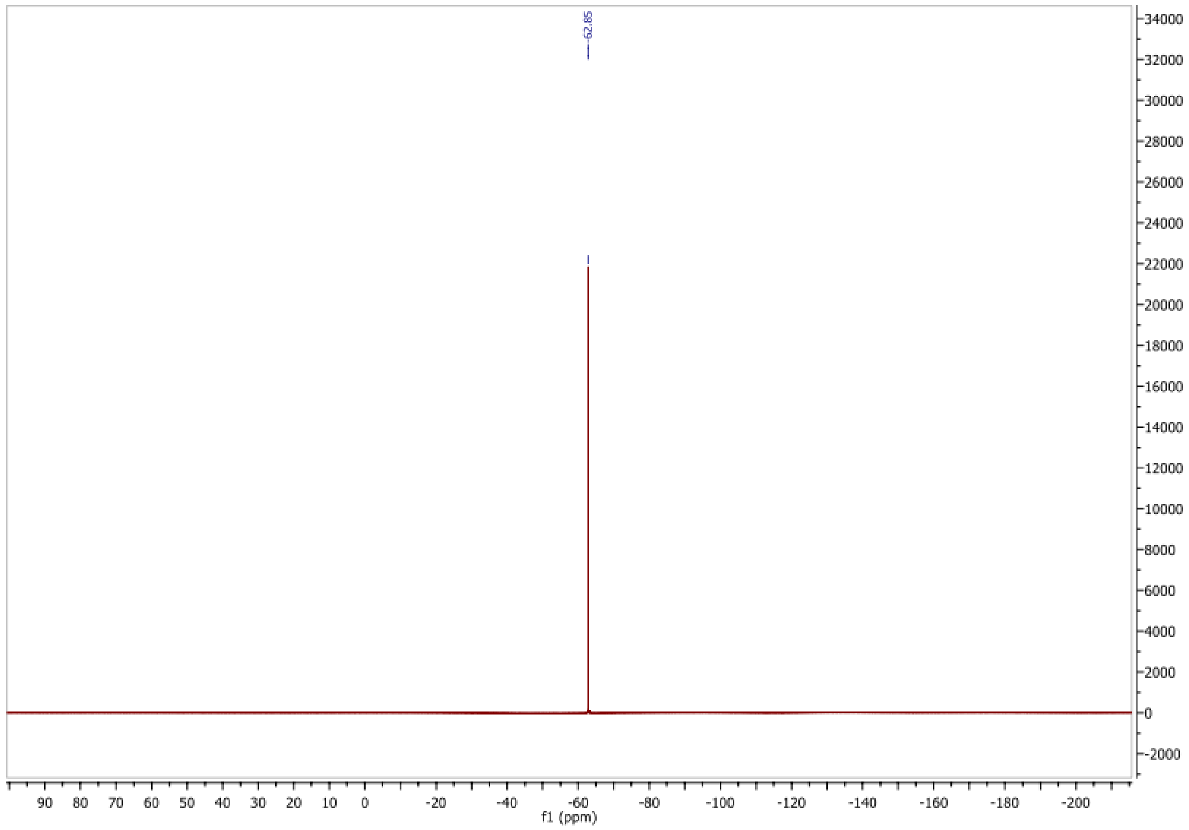
-62.85
f1 (ppm)

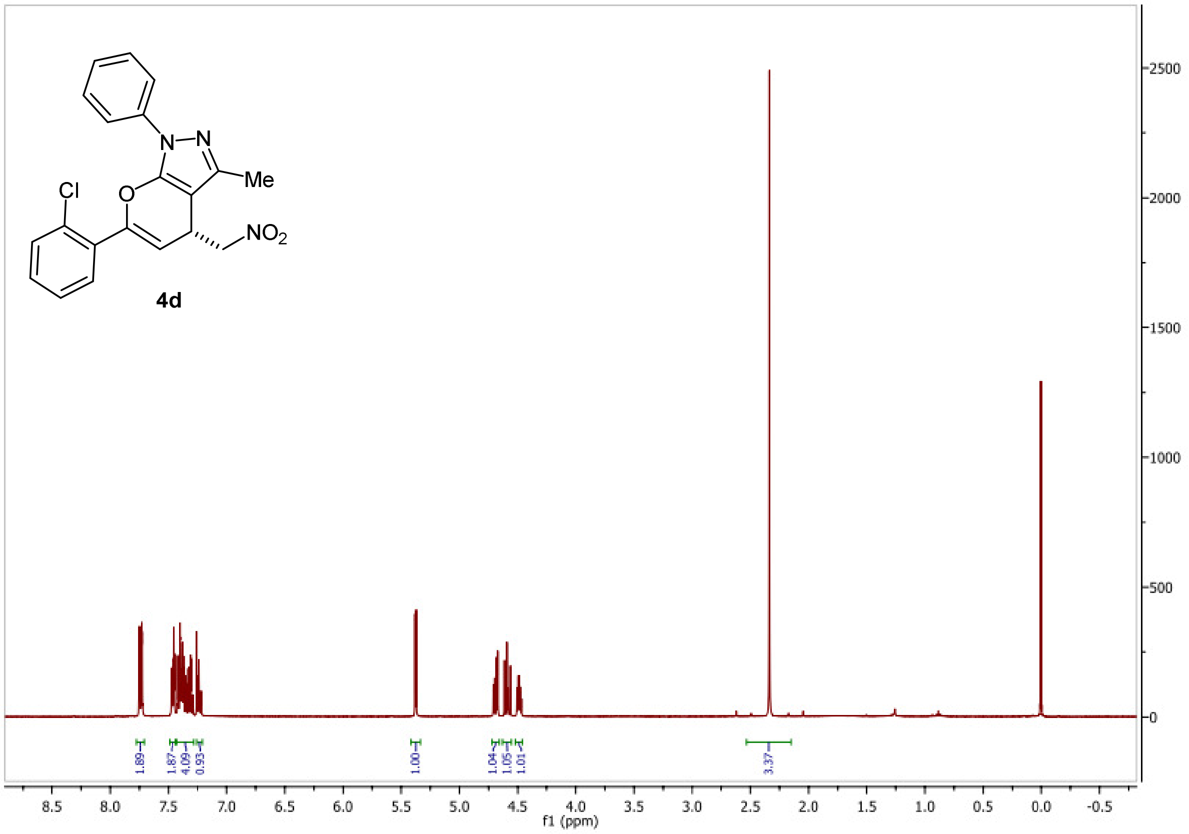
Cl
O
N
N
Me
NO2
4d
1.89
1.87
4.09
0.93
1.00
1.04
1.05
1.01
3.37
f1 (ppm)

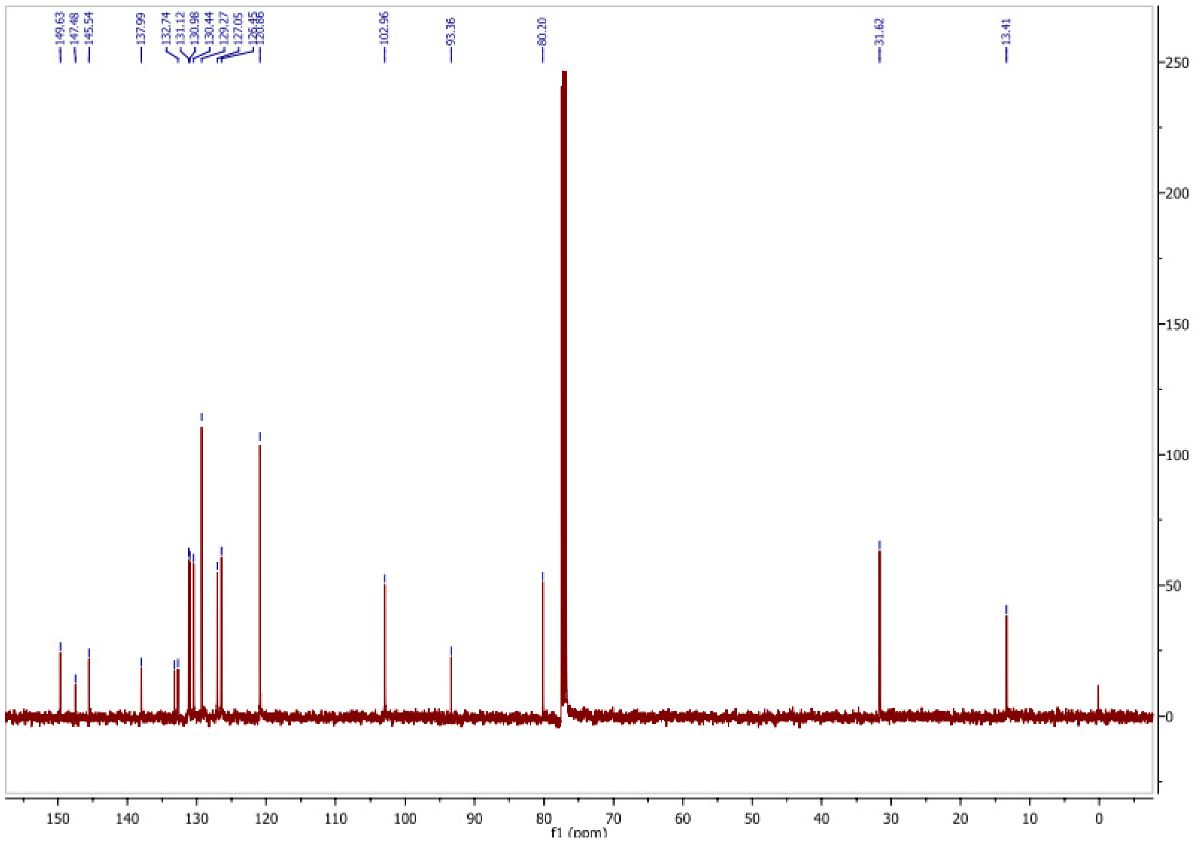

149.63
147.48
145.54
137.99
132.74
131.12
130.98
130.44
129.27
127.05
126.45
120.86
102.96
93.36
80.20
31.62
13.41
f1 (ppm)

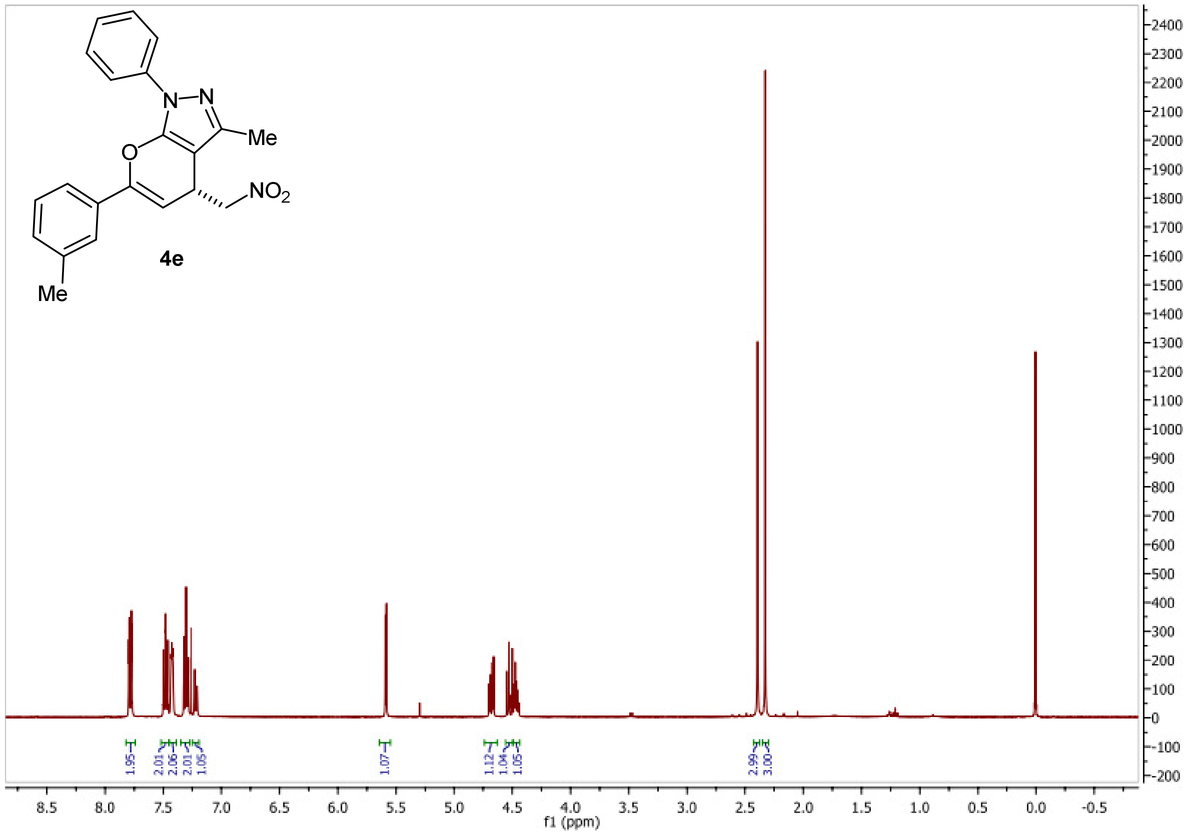

N
N
O
Me
NO2
Me
4e
1.95
2.01
2.06
2.01
1.05
1.07
1.12
1.04
1.05
2.99
3.00
f1 (ppm)

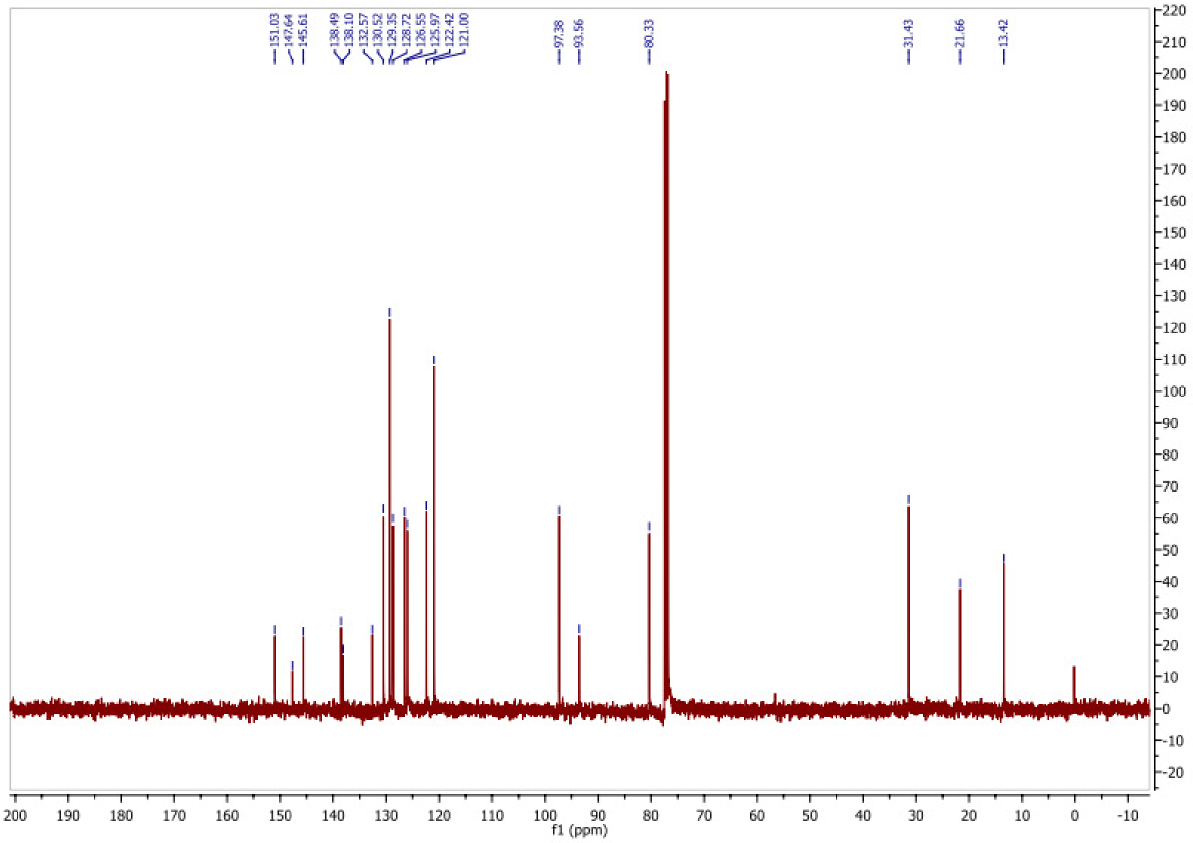
151.03
147.64
145.61
138.49
138.10
132.57
130.52
129.35
128.72
126.55
125.97
122.42
121.00
97.38
93.56
80.33
31.43
21.66
13.42
f1 (ppm)

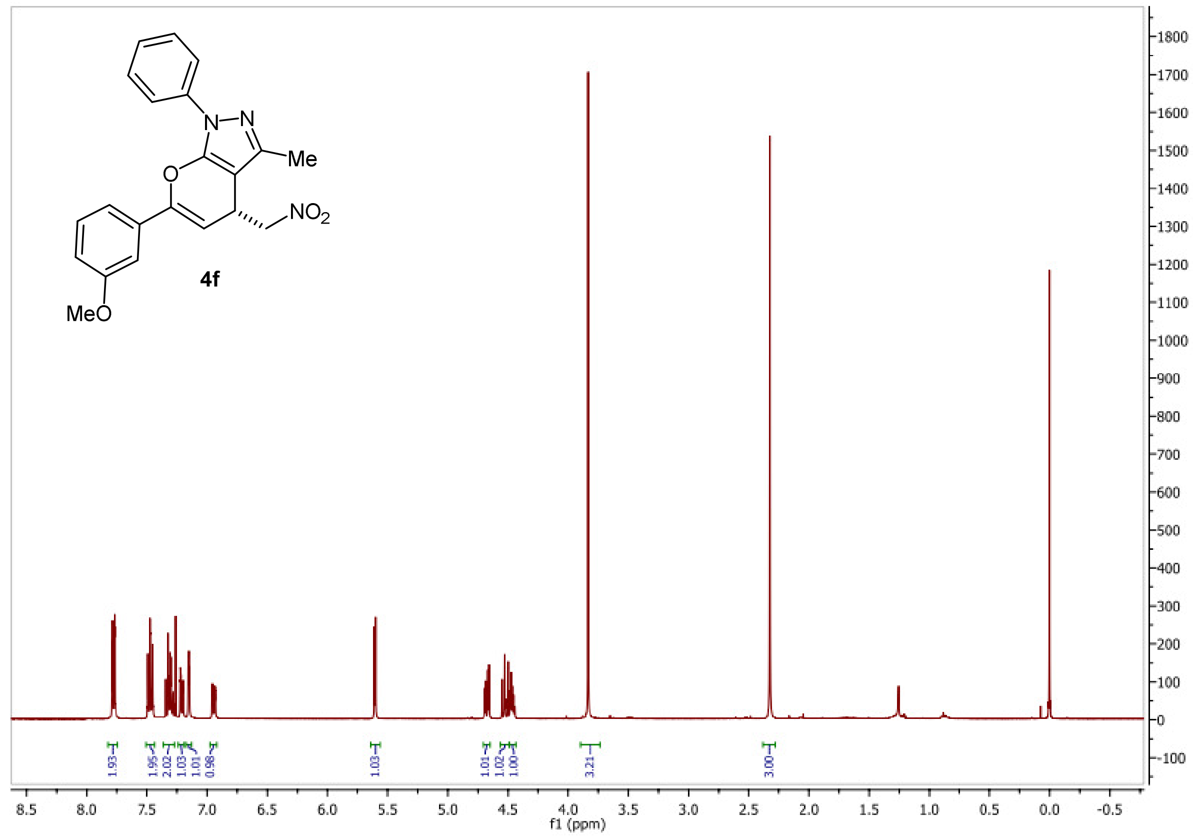
N
N
Me
O
NO2
MeO
4f
1.93
1.95
2.02
1.03
1.01
0.98
1.03
1.01
1.02
1.00
3.21
3.00
f1 (ppm)

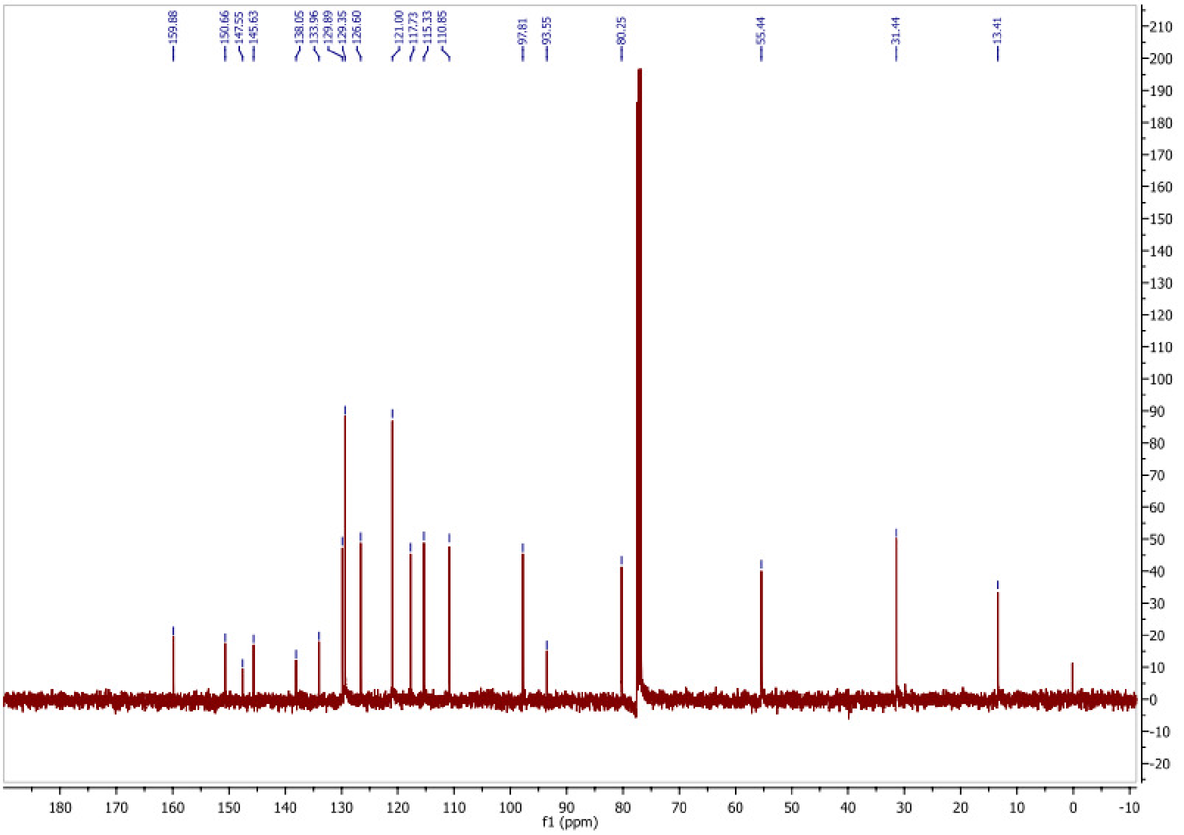
159.88
150.66
147.55
145.63
138.05
133.96
129.89
129.35
126.60
121.00
117.73
115.33
110.85
97.81
93.55
80.25
55.44
31.44
13.41
f1 (ppm)

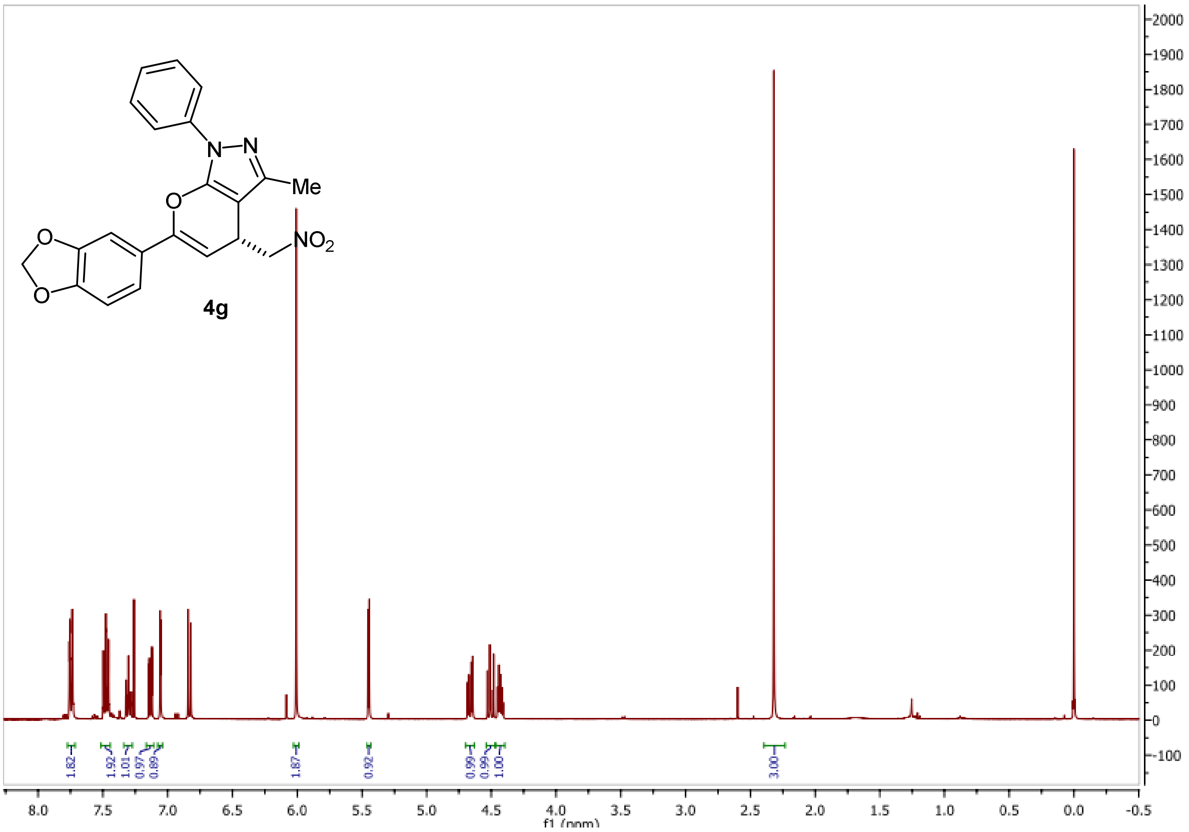
N
N
Me
O
O
O
NO2
4g
1.82
1.92
1.01
0.97
0.89
1.87
0.92
0.99
0.99
1.00
3.00
f1 (ppm)

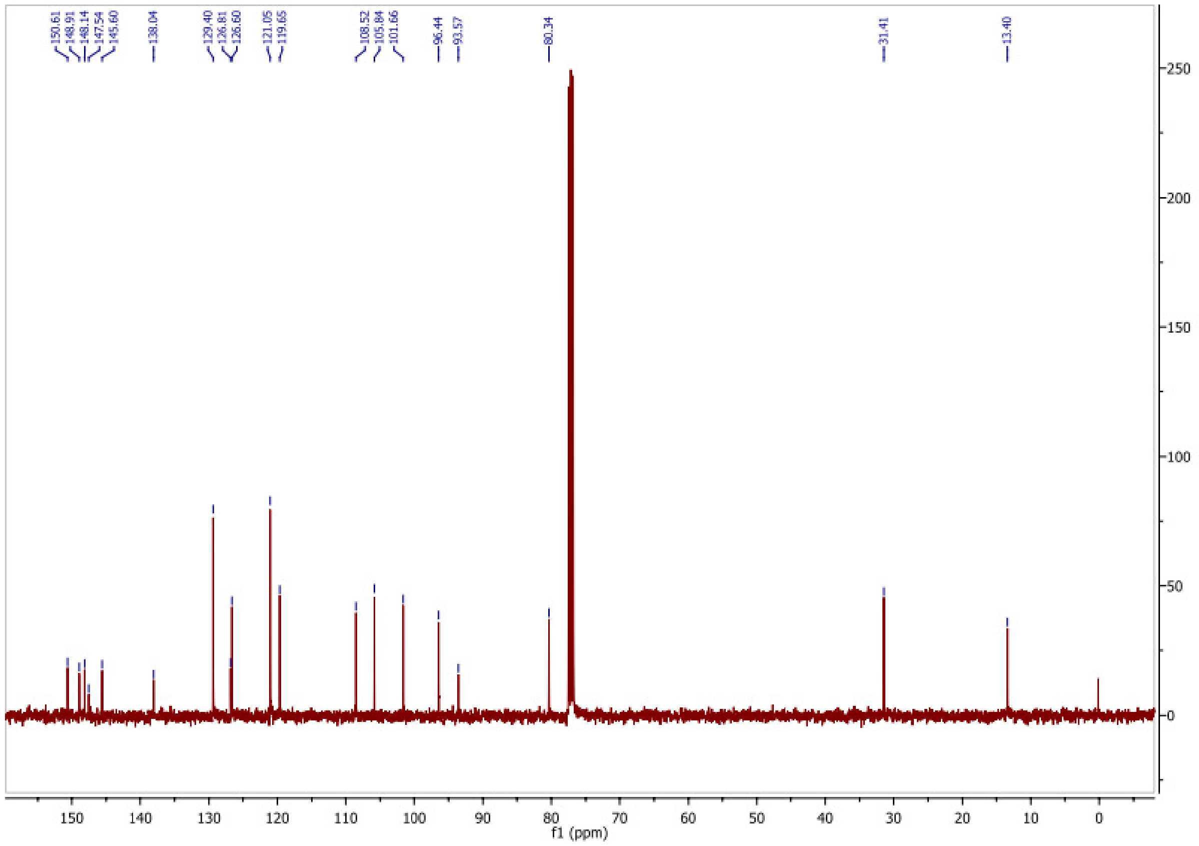
150.61
148.91
148.14
147.54
145.60
138.04
129.40
126.81
126.60
121.05
119.65
108.52
105.84
101.66
96.44
93.57
80.34
31.41
13.40
f1 (ppm)

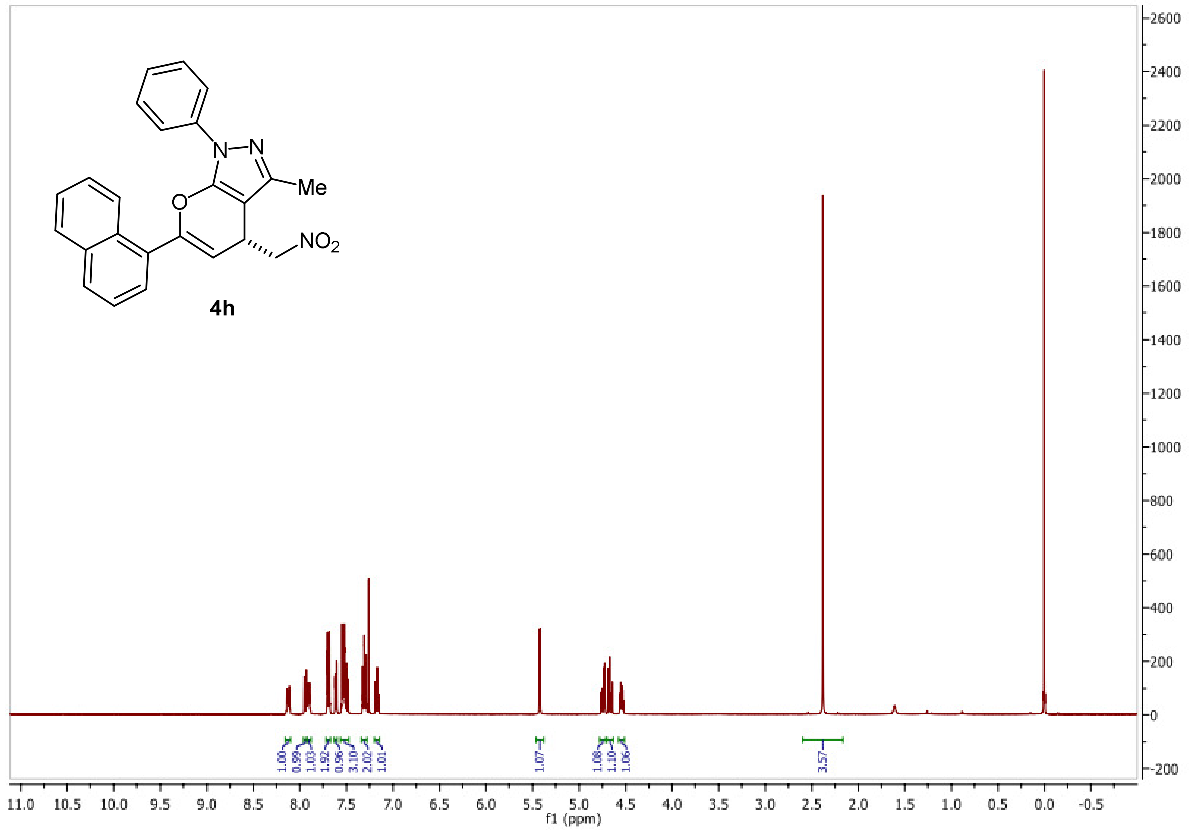
N
N
Me
O
NO2
4h
1.00
0.99
1.03
1.92
0.96
3.10
2.02
1.01
1.07
1.08
1.10
1.06
3.57
f1 (ppm)

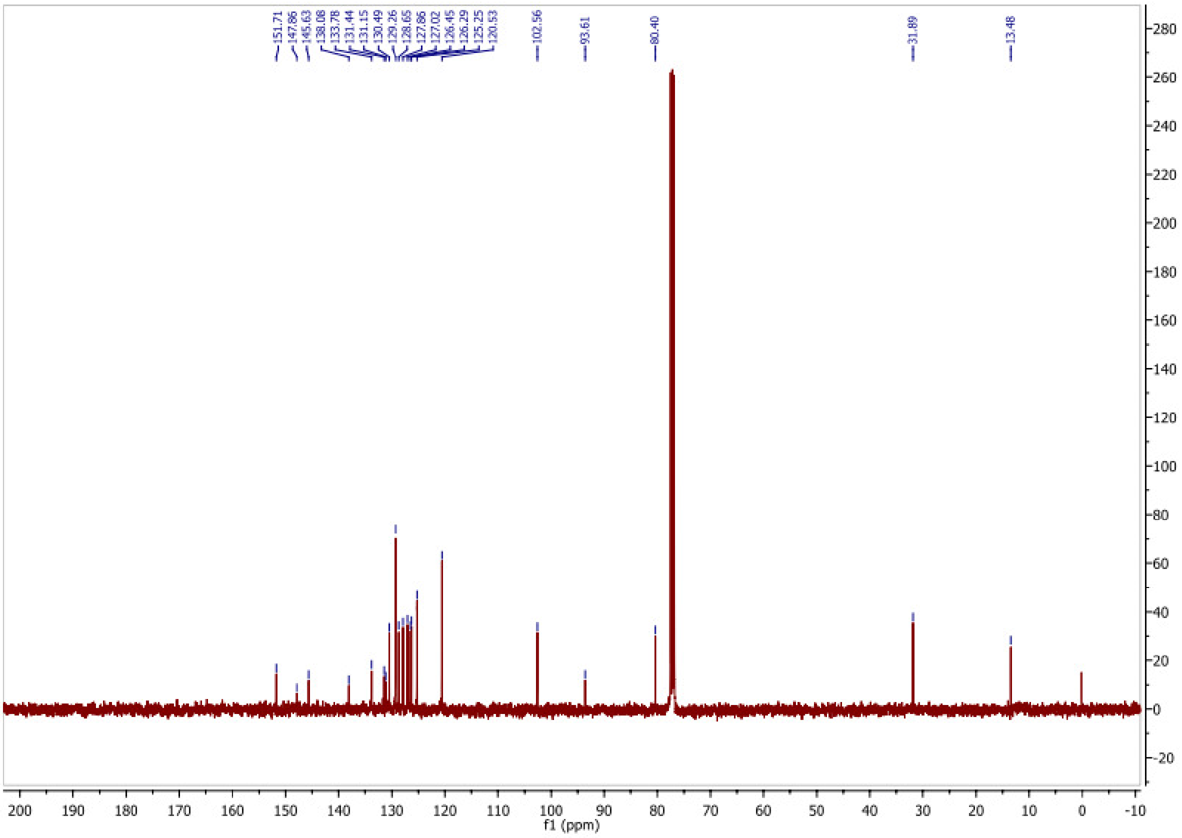
151.71
147.86
145.63
138.08
133.78
131.44
131.15
130.49
129.26
128.65
127.86
127.02
126.45
126.29
125.25
120.53
102.56
93.61
80.40
31.89
13.48
f1 (ppm)

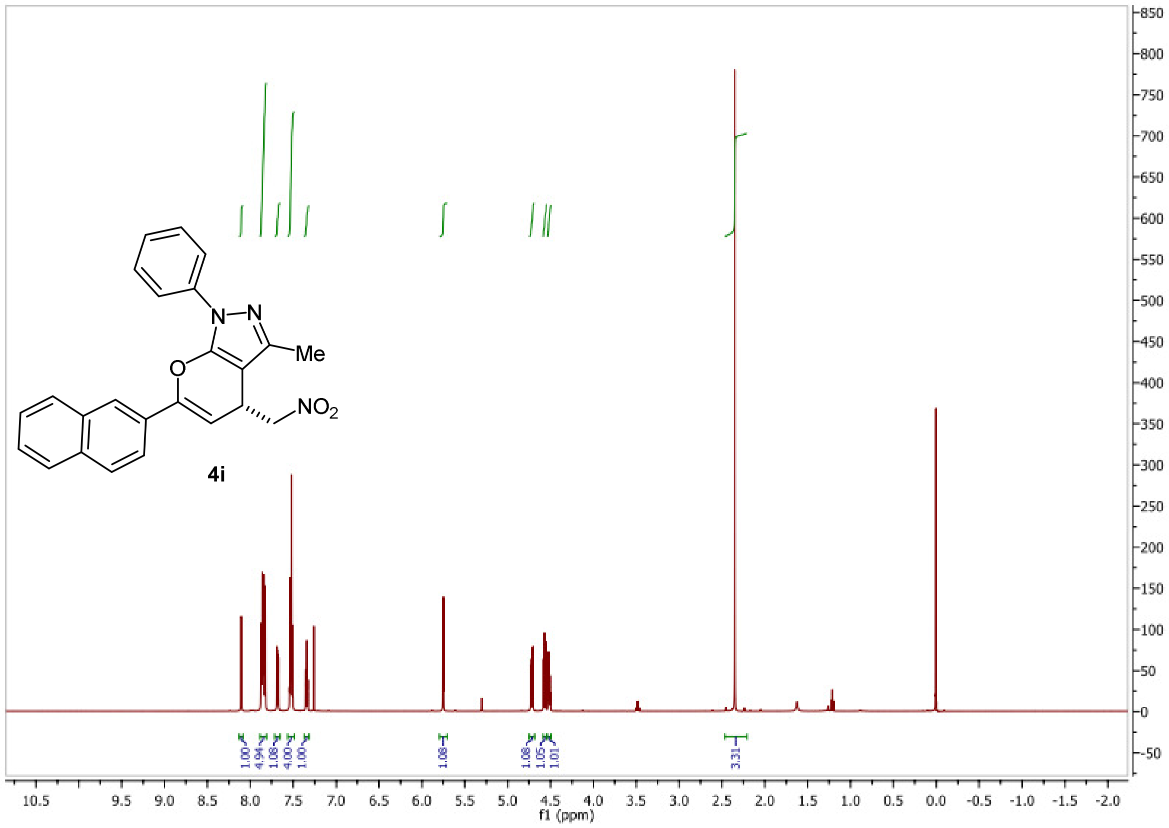
N
N
Me
O
NO2
4i
1.00
4.94
1.08
4.00
1.00
1.08
1.08
1.05
1.01
3.31
f1 (ppm)

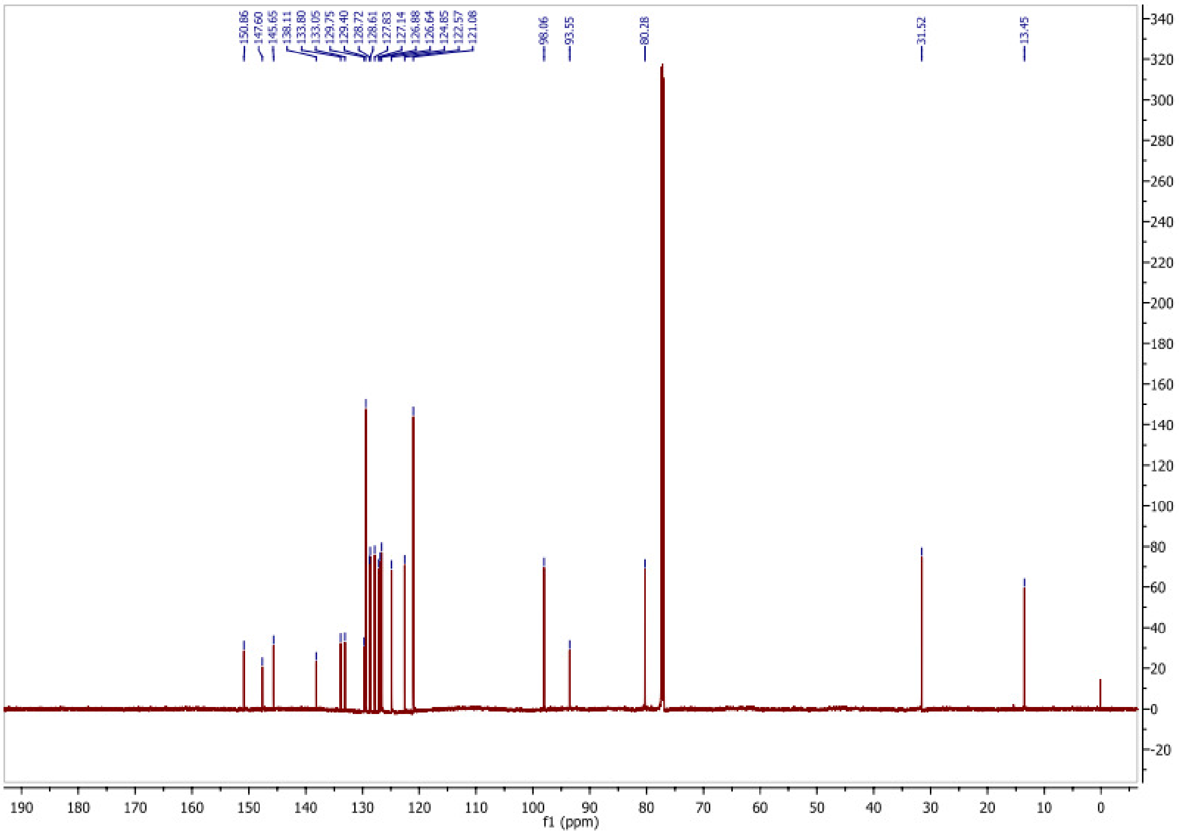
150.86
147.60
145.65
138.11
133.80
133.05
129.75
129.40
128.72
128.61
127.83
127.14
126.88
126.64
124.85
122.57
121.08
98.06
93.55
80.28
31.52
13.45
f1 (ppm)

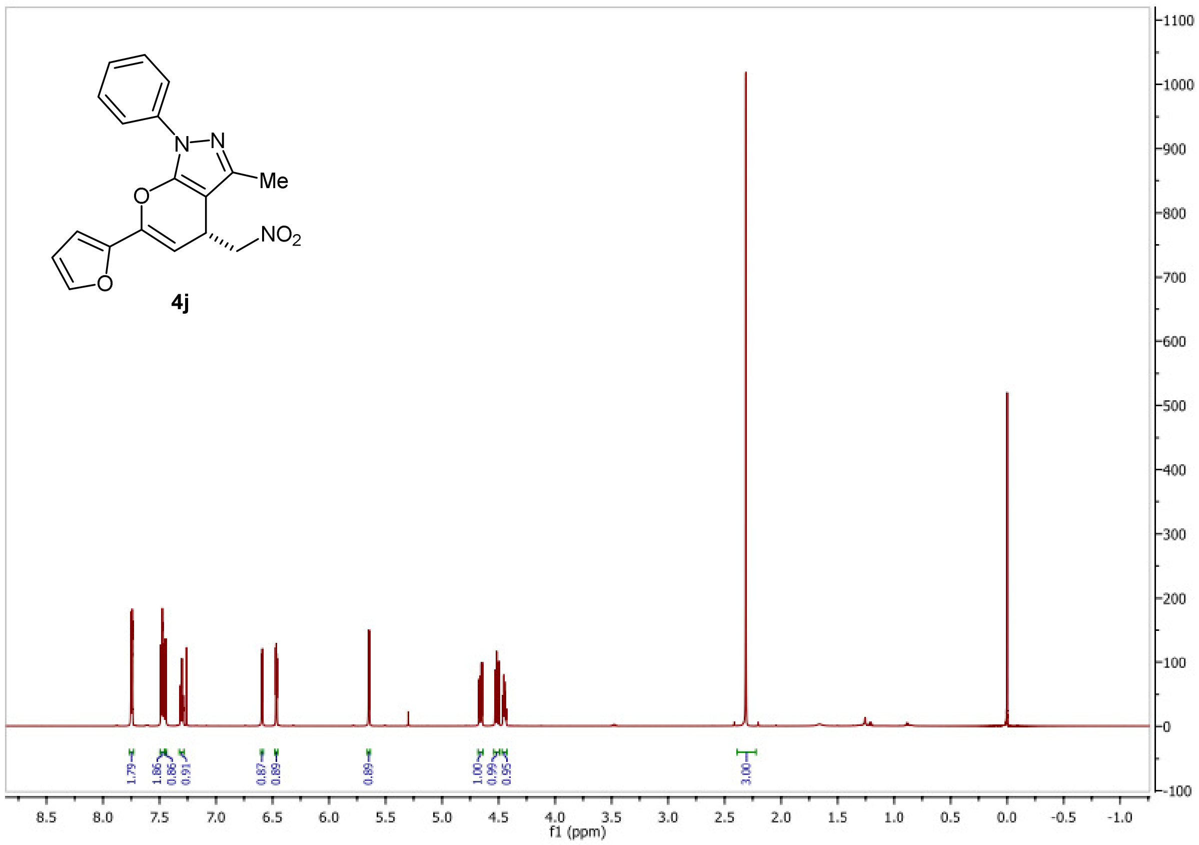
N
N
Me
O
NO2
O
4j
1.79
1.86
0.86
0.91
0.87
0.89
0.89
1.00
0.99
0.95
3.00
f1 (ppm)

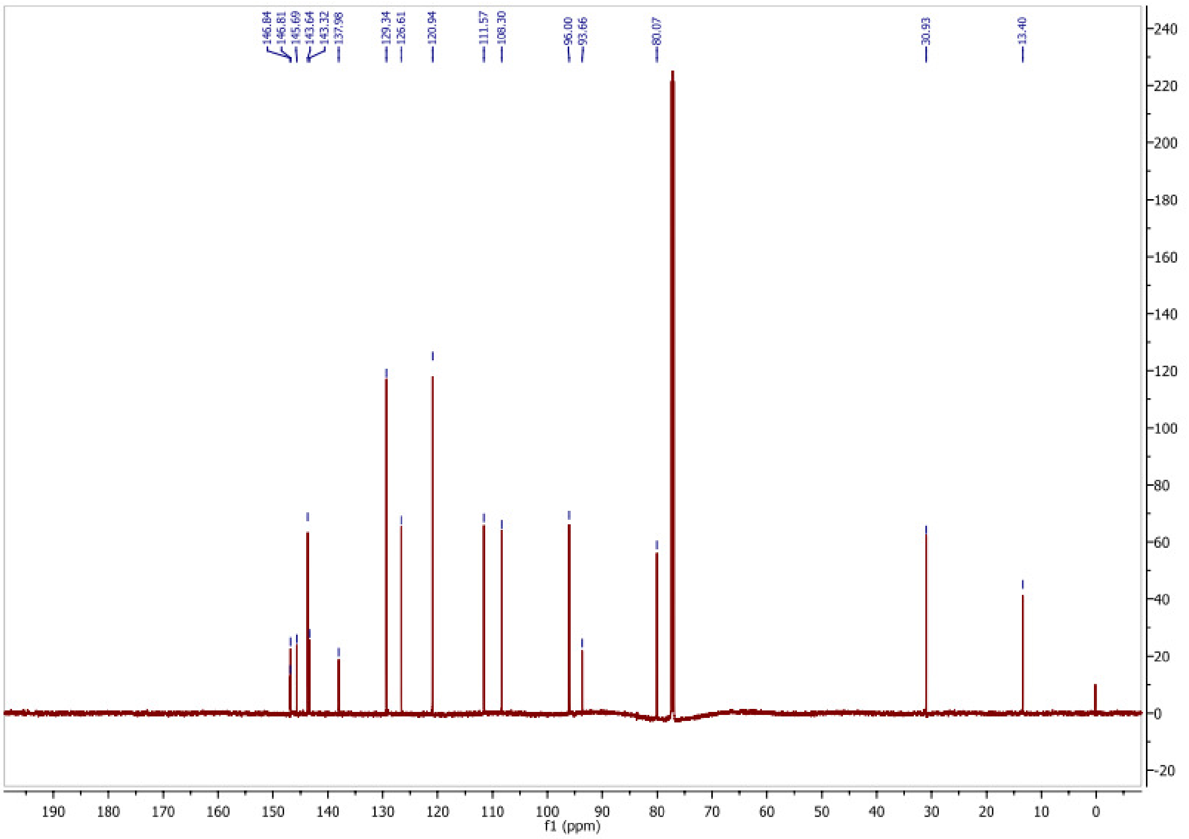
146.84
146.81
145.69
143.64
143.32
137.98
129.34
126.61
120.94
111.57
108.30
96.00
93.66
80.07
30.93
13.40
f1 (ppm)

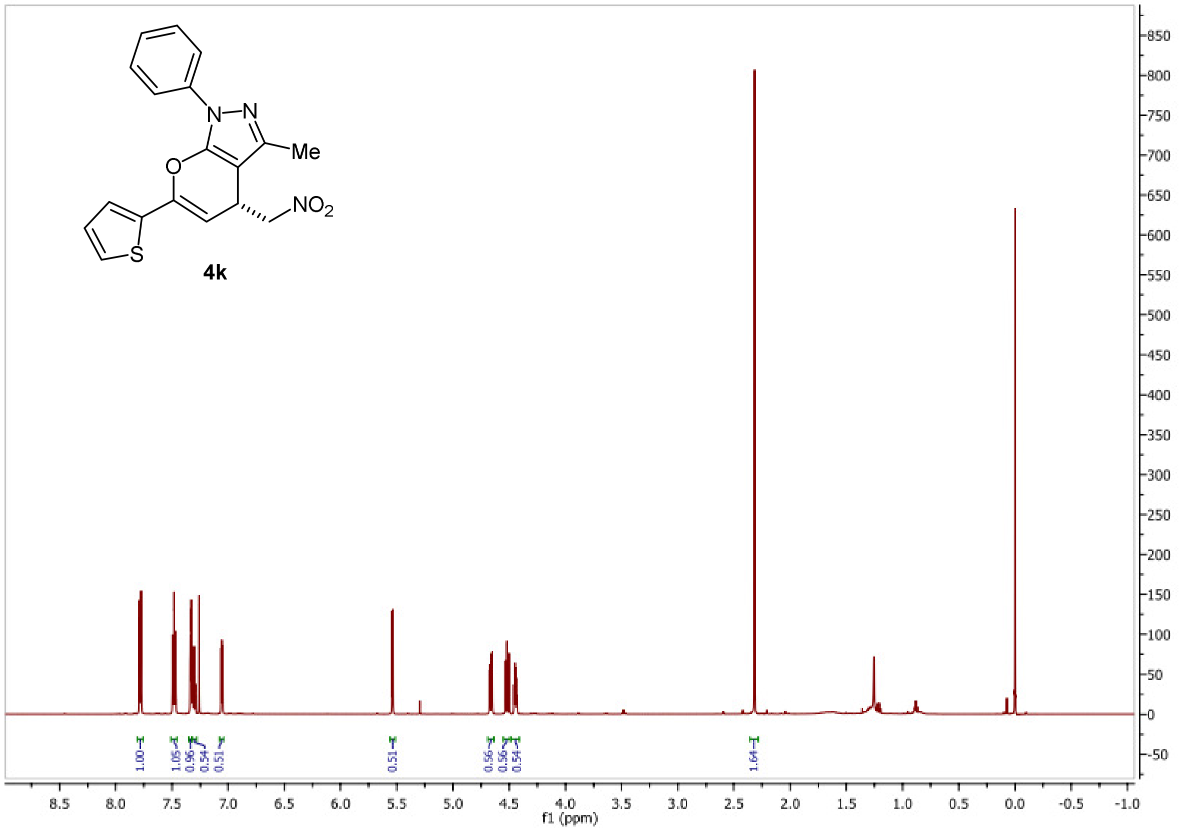
N
N
Me
O
NO2
S
4k
1.00
1.05
0.96
0.54
0.51
0.51
0.56
0.56
0.54
1.64
f1 (ppm)

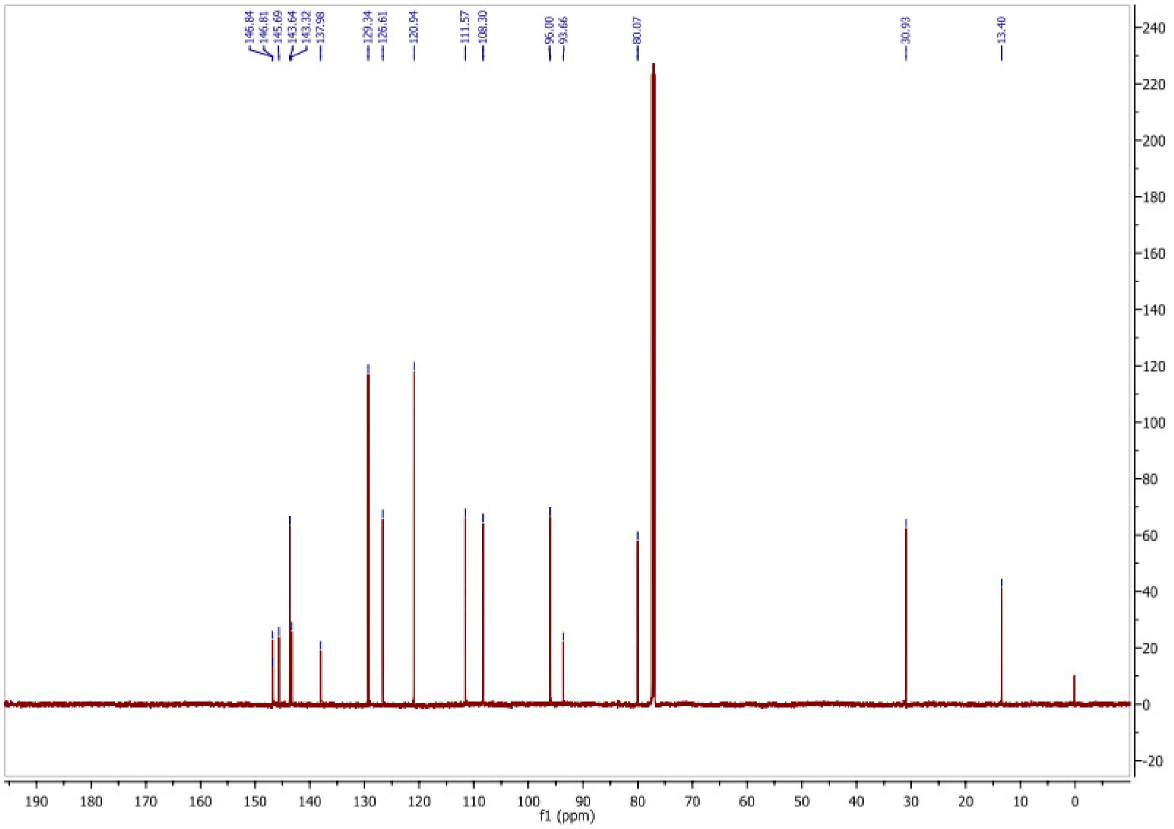

146.84
146.81
145.69
143.64
143.32
137.98
129.34
126.61
120.94
111.57
108.30
96.00
93.66
80.07
30.93
13.40
f1 (ppm)

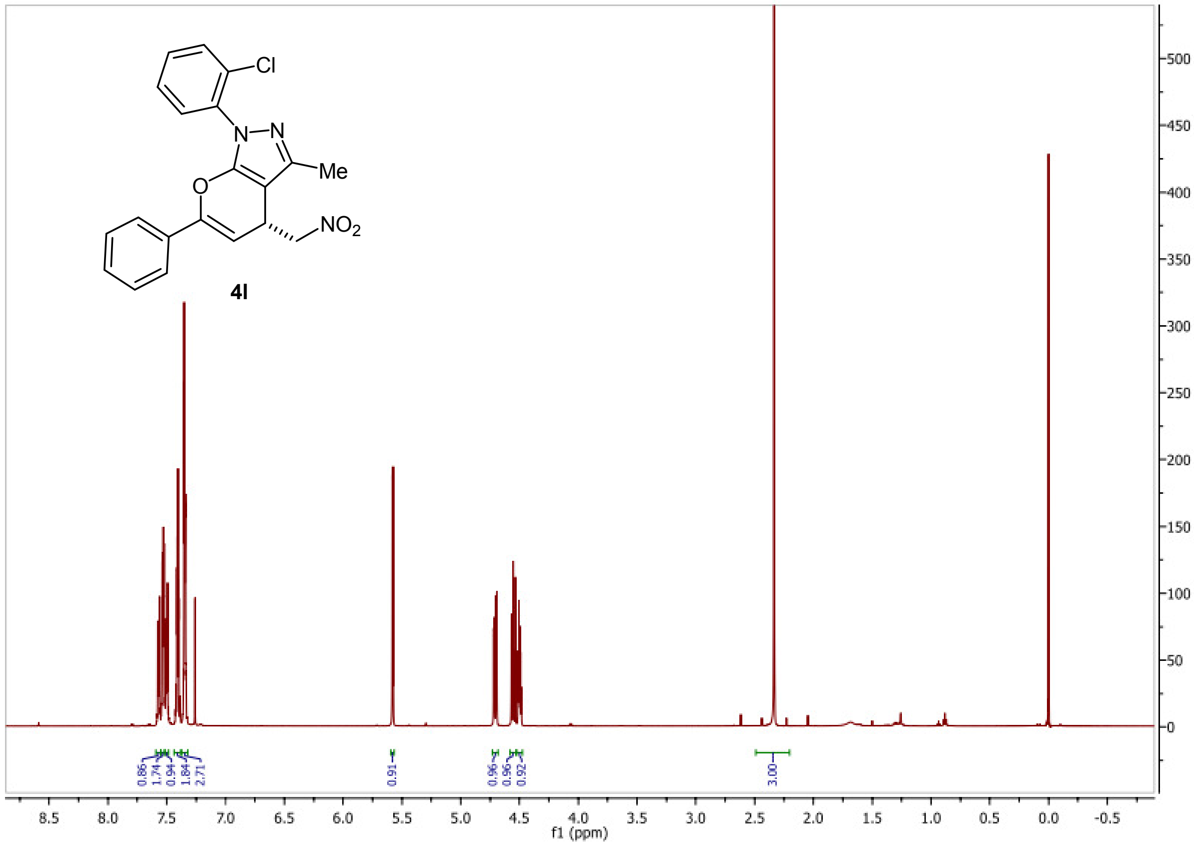

Cl
N
N
Me
O
NO2
4l
0.86
1.74
0.94
1.84
2.71
0.91
0.96
0.96
0.92
3.00
f1 (ppm)

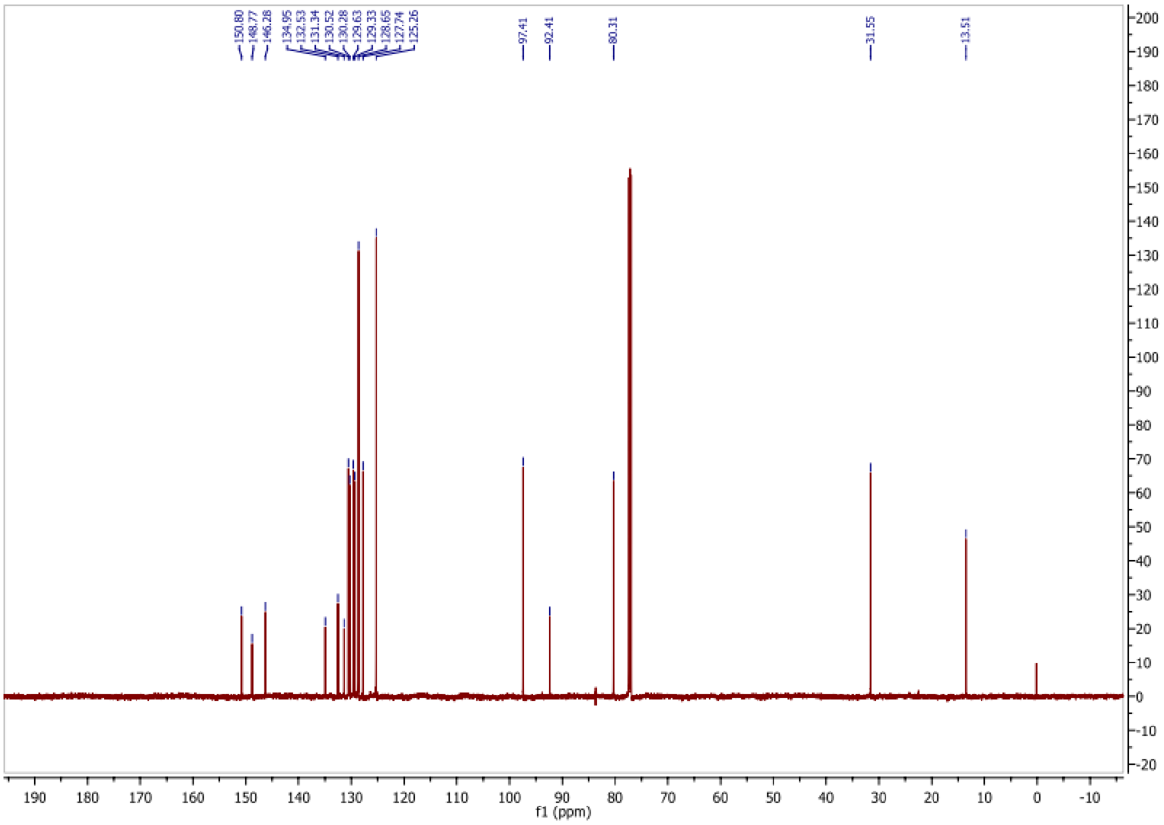
150.80
148.77
146.28
134.95
132.53
131.34
130.52
130.28
129.63
129.33
128.65
127.74
125.26
97.41
92.41
80.31
31.55
13.51
f1 (ppm)

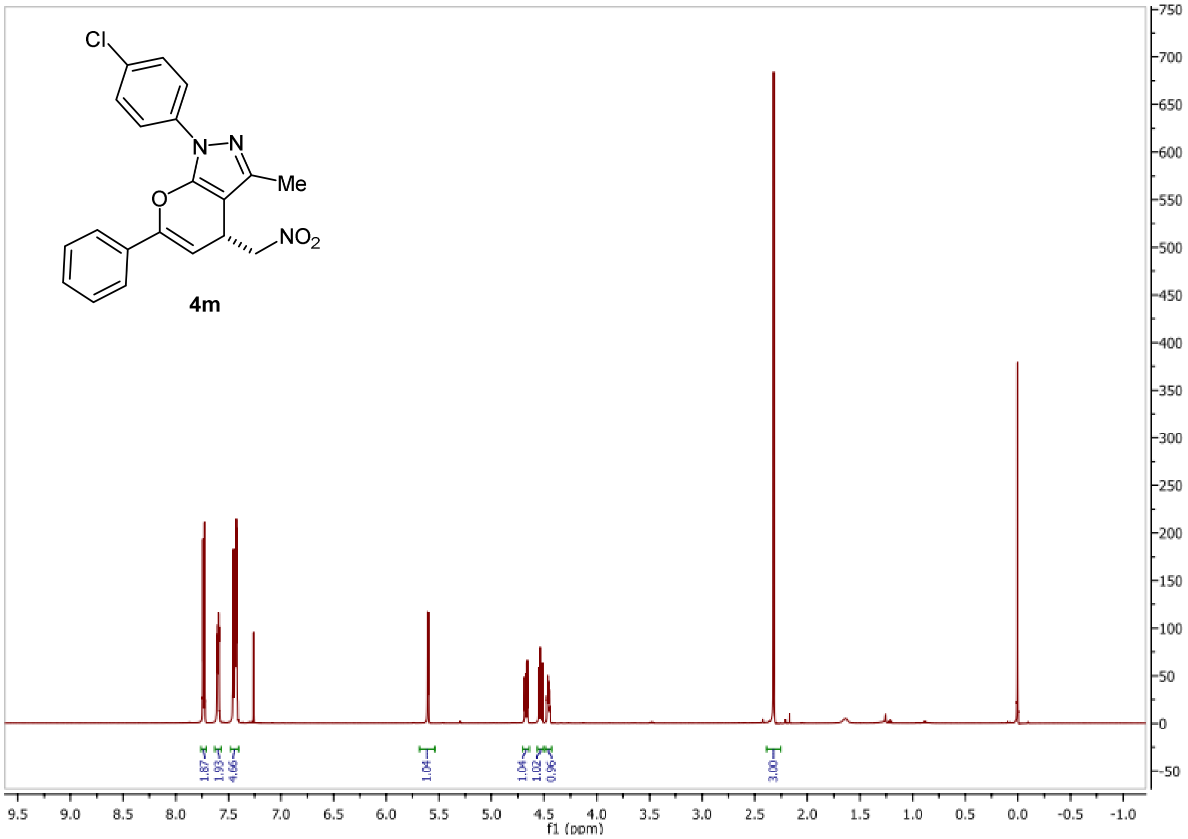
Cl
N
N
Me
O
NO_2
4m
1.87
1.93
4.66
1.04
1.04
1.02
0.96
3.00
f1 (ppm)

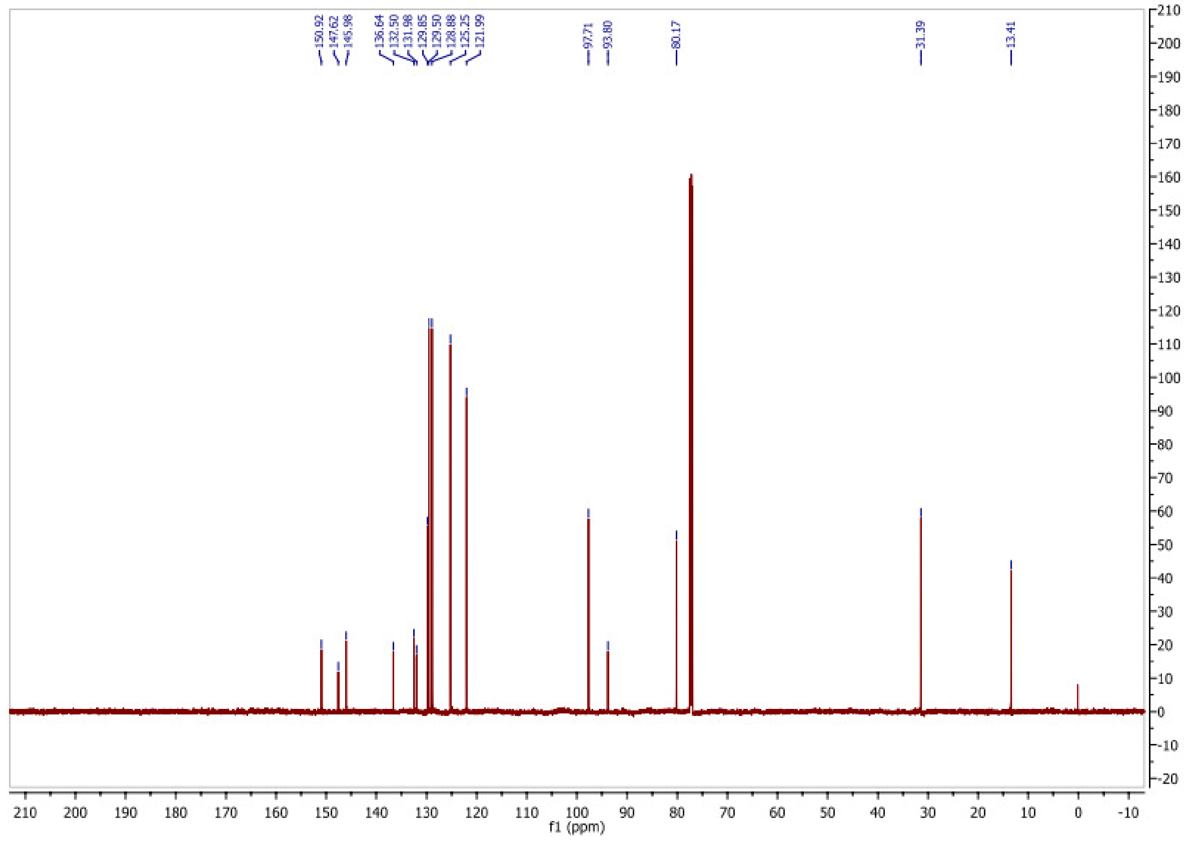
150.92
147.62
145.98
136.64
132.50
131.98
129.85
129.50
128.88
125.25
121.99
97.71
93.80
80.17
31.39
13.41
f1 (ppm)

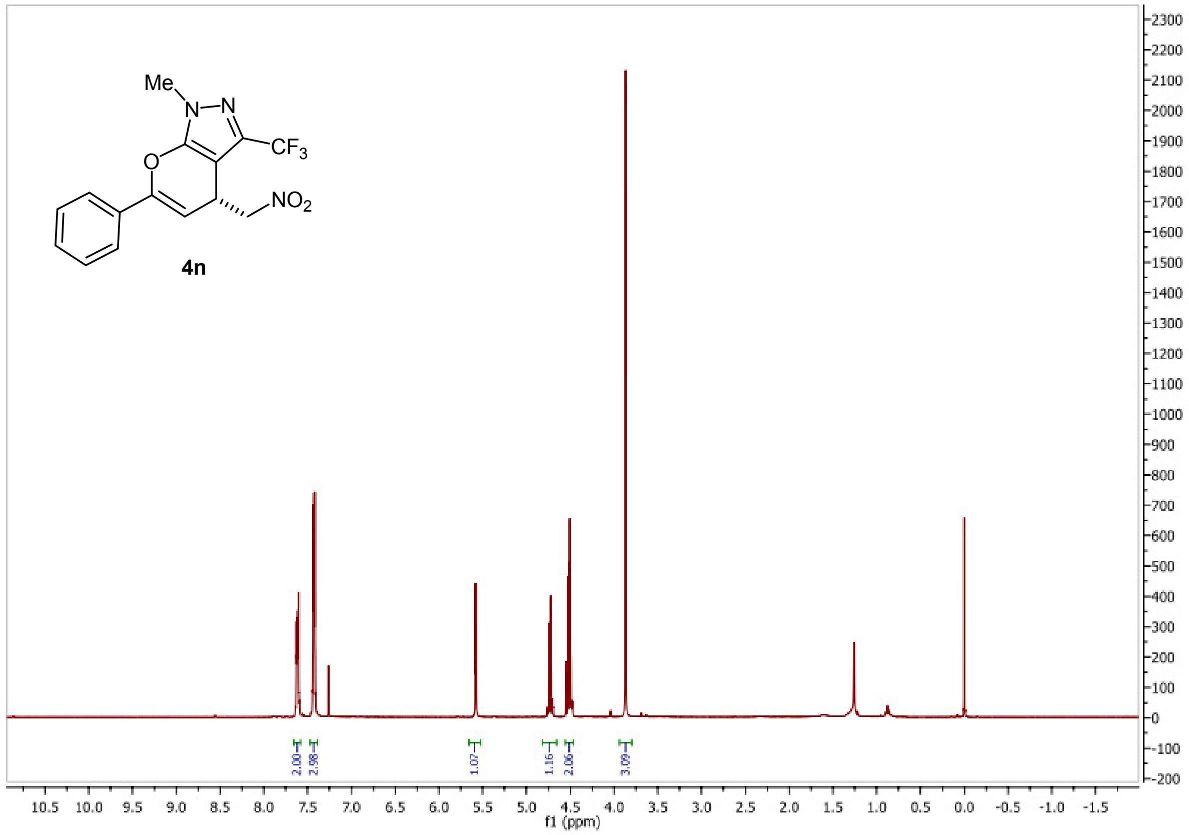
Me
N
N
CF3
O
NO2
4n
2.00
2.98
1.07
1.16
2.06
3.09
f1 (ppm)

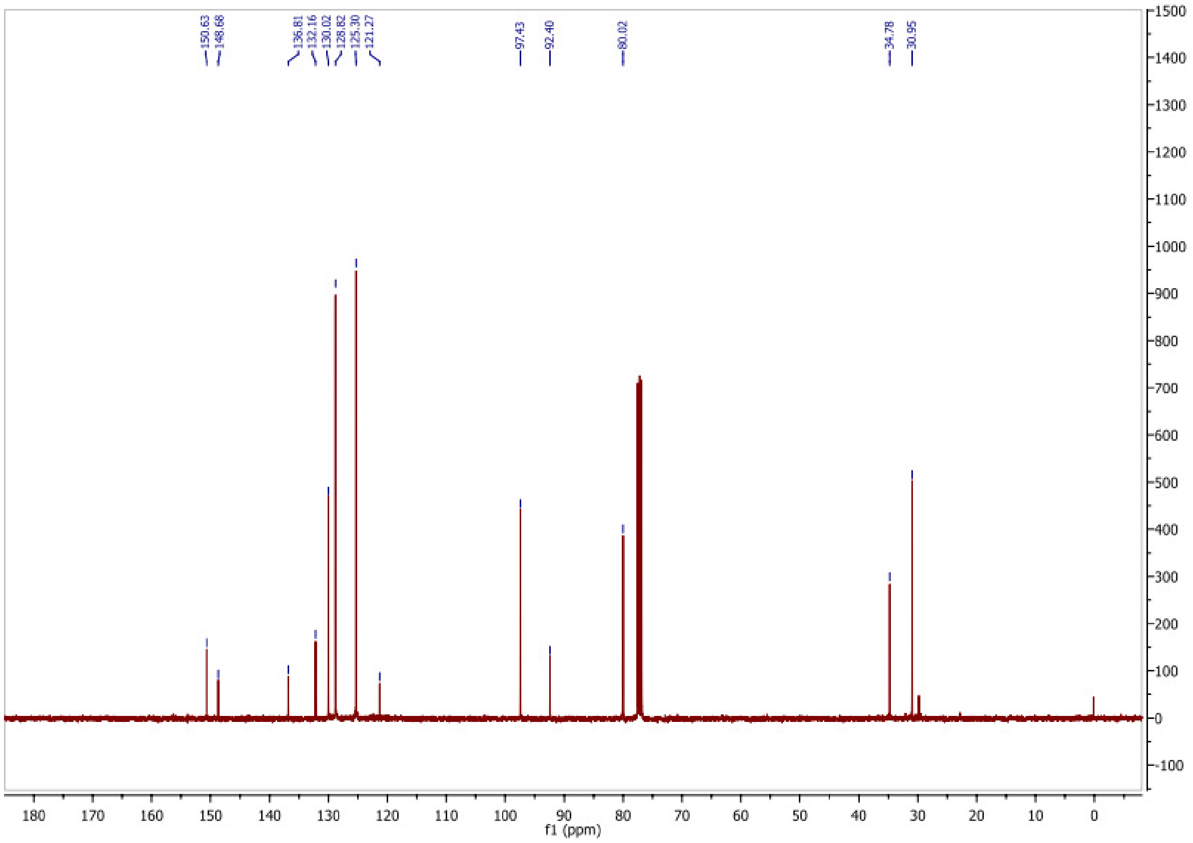
150.63
148.68
136.81
132.16
130.02
128.82
125.30
121.27
97.43
92.40
80.02
34.78
30.95
f1 (ppm)

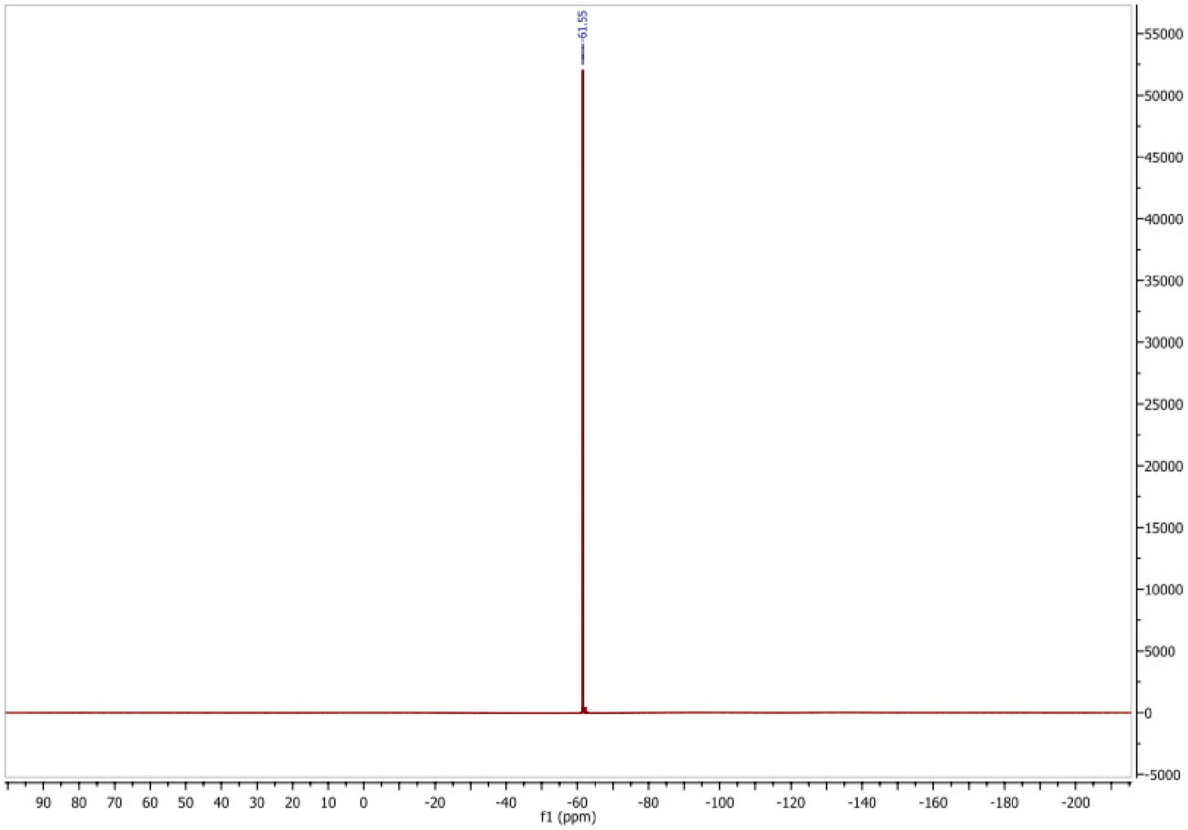
-61.55
f1 (ppm)

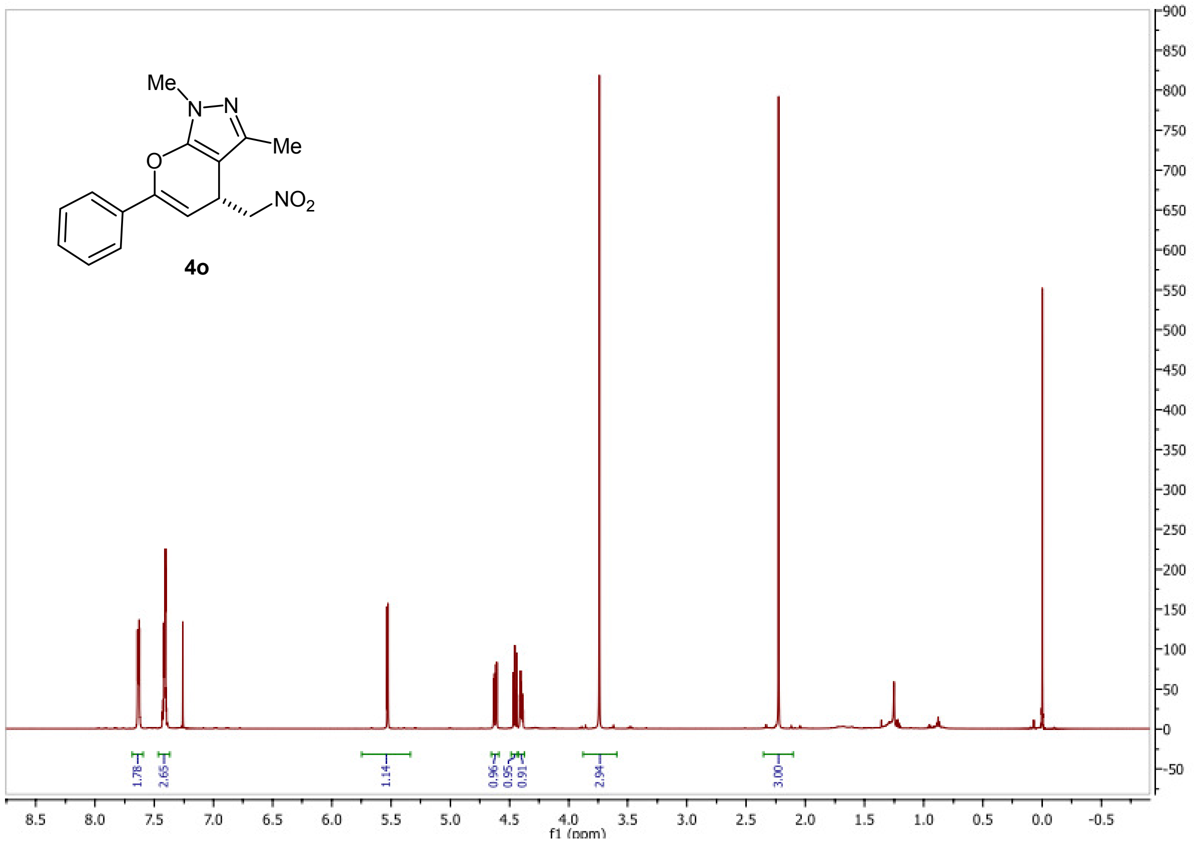
Me
N
N
Me
O
NO2
4o
1.78
2.65
1.14
0.96
0.95
0.91
2.94
3.00
f1 (ppm)

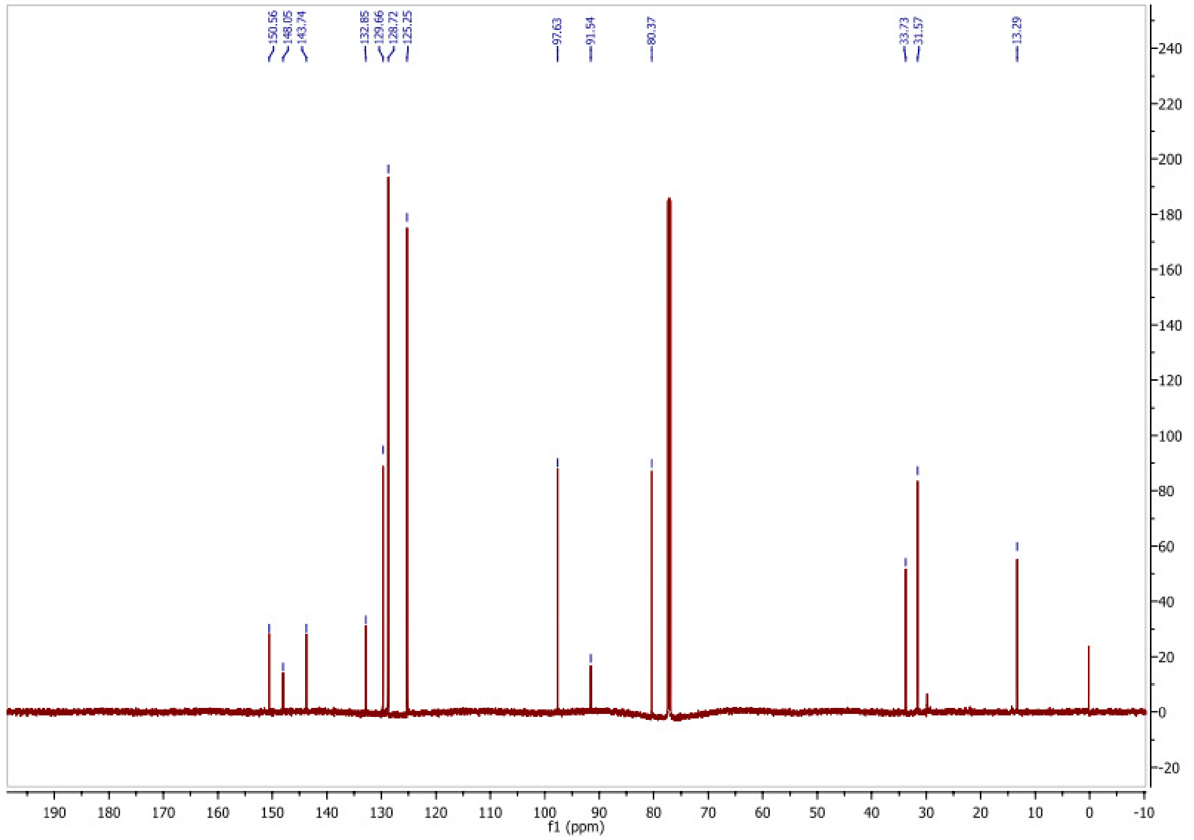
150.56
148.05
143.74
132.85
129.66
128.72
125.25
97.63
91.54
80.37
33.73
31.57
13.29
f1 (ppm)

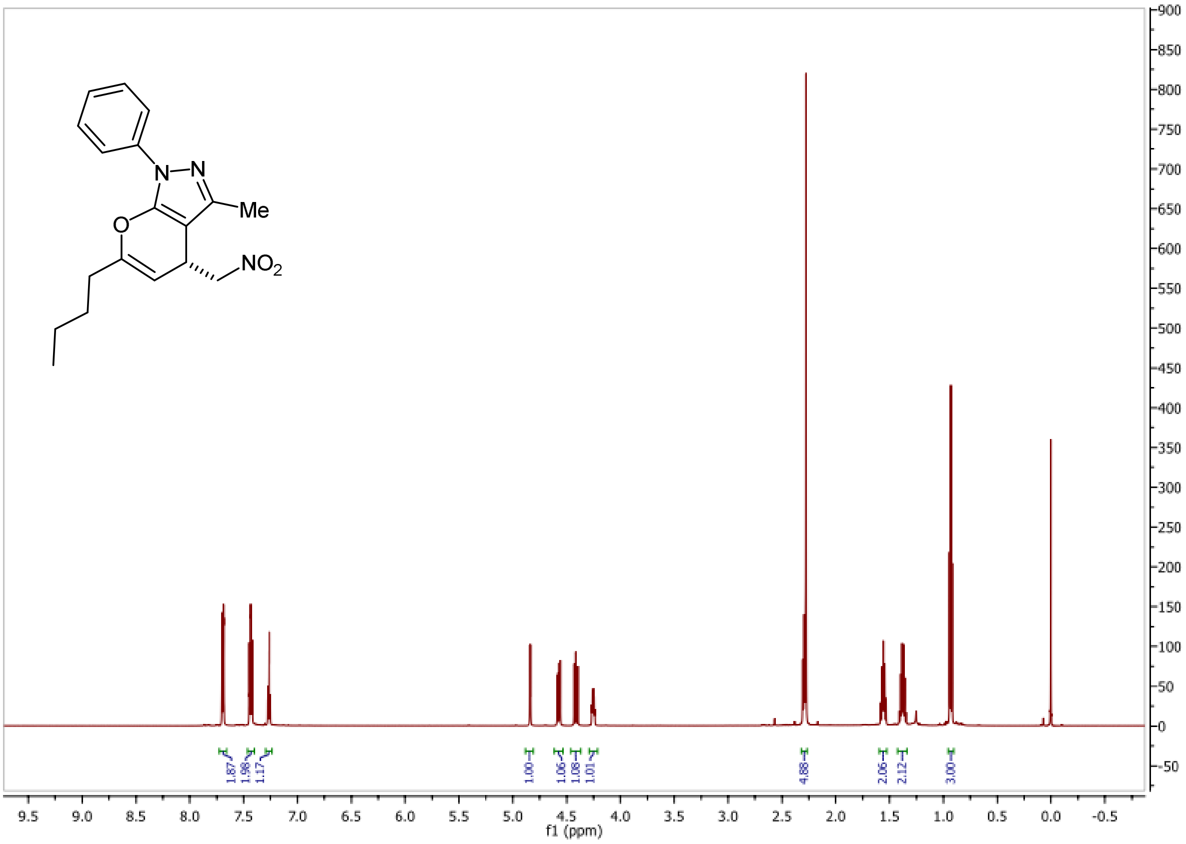
N
N
Me
O
NO2
1.87
1.98
1.17
1.00
1.06
1.08
1.01
4.88
2.06
2.12
3.00
f1 (ppm)

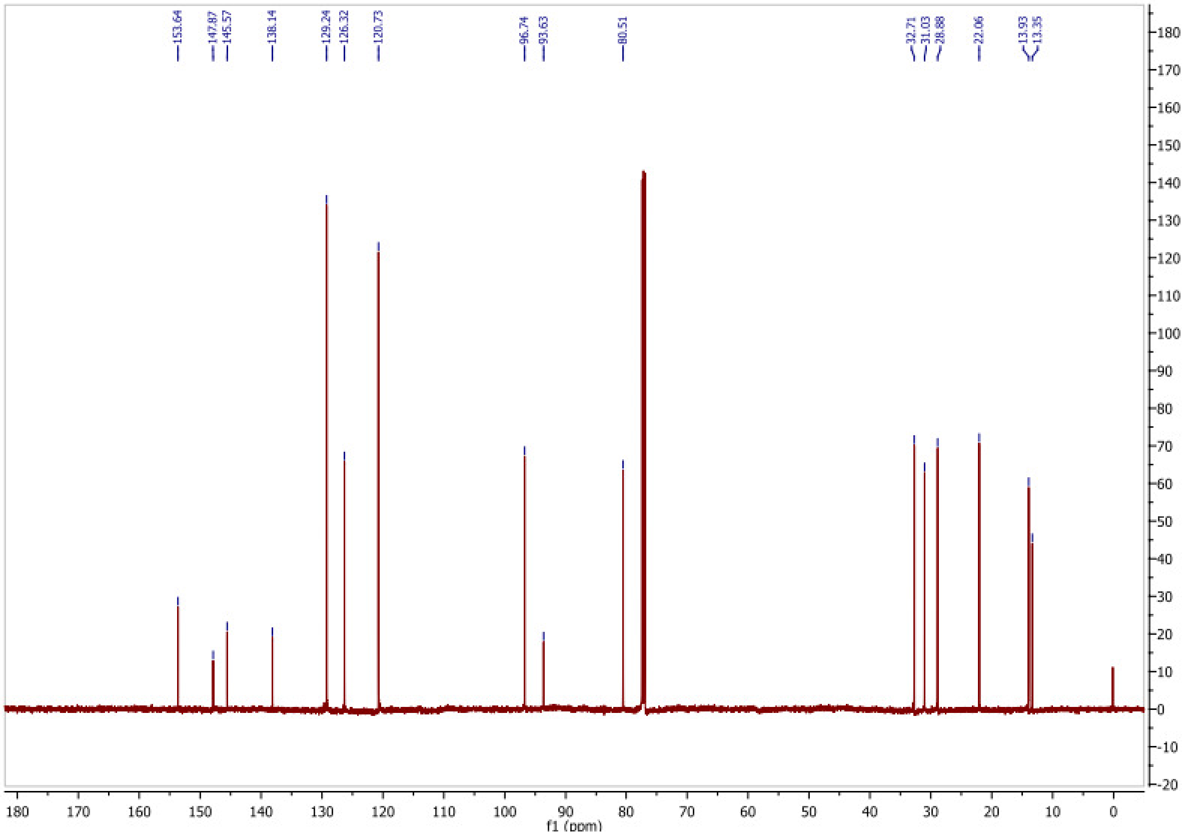
153.64
147.87
145.57
138.14
129.24
126.32
120.73
96.74
93.63
80.51
32.71
31.03
28.88
22.06
13.93
13.35
f1 (ppm)

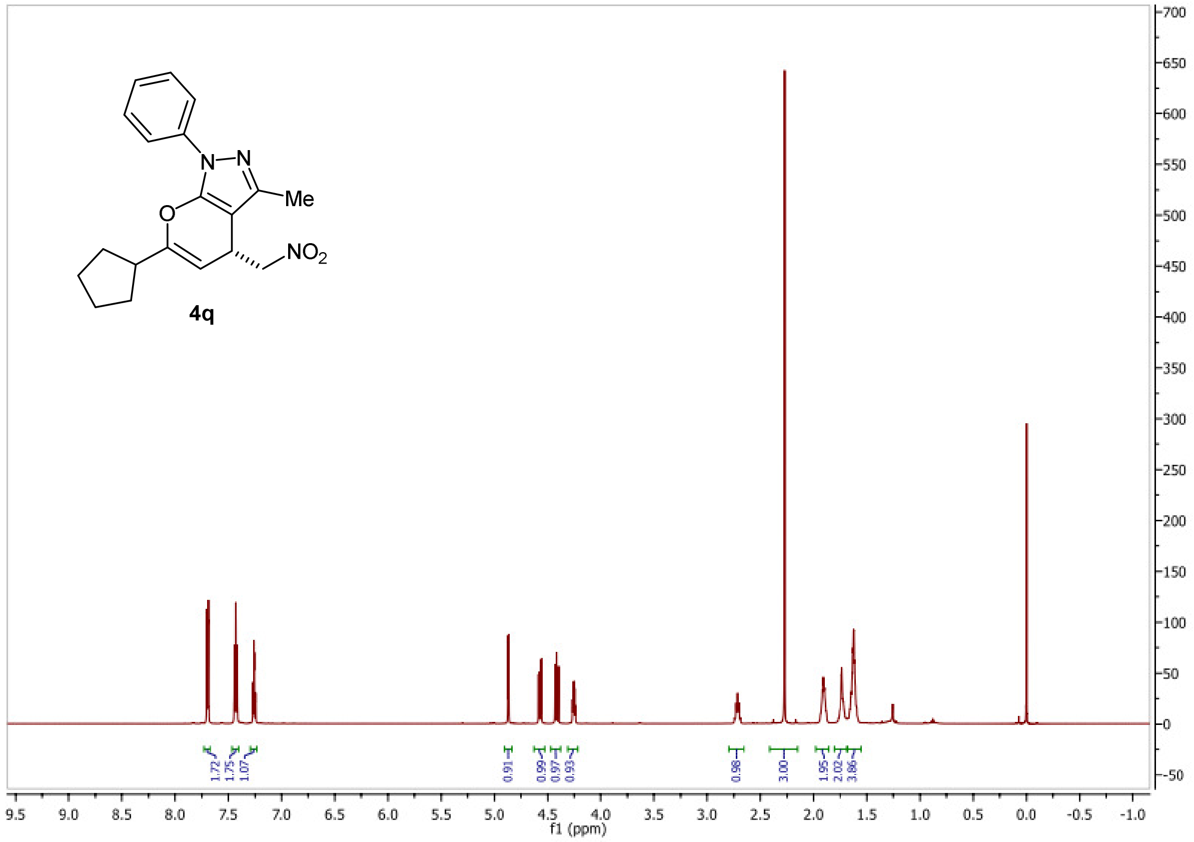

N
N
Me
O
NO2
4q
1.72
1.75
1.07
0.91
0.99
0.97
0.93
0.98
3.00
1.95
2.02
3.86
f1 (ppm)

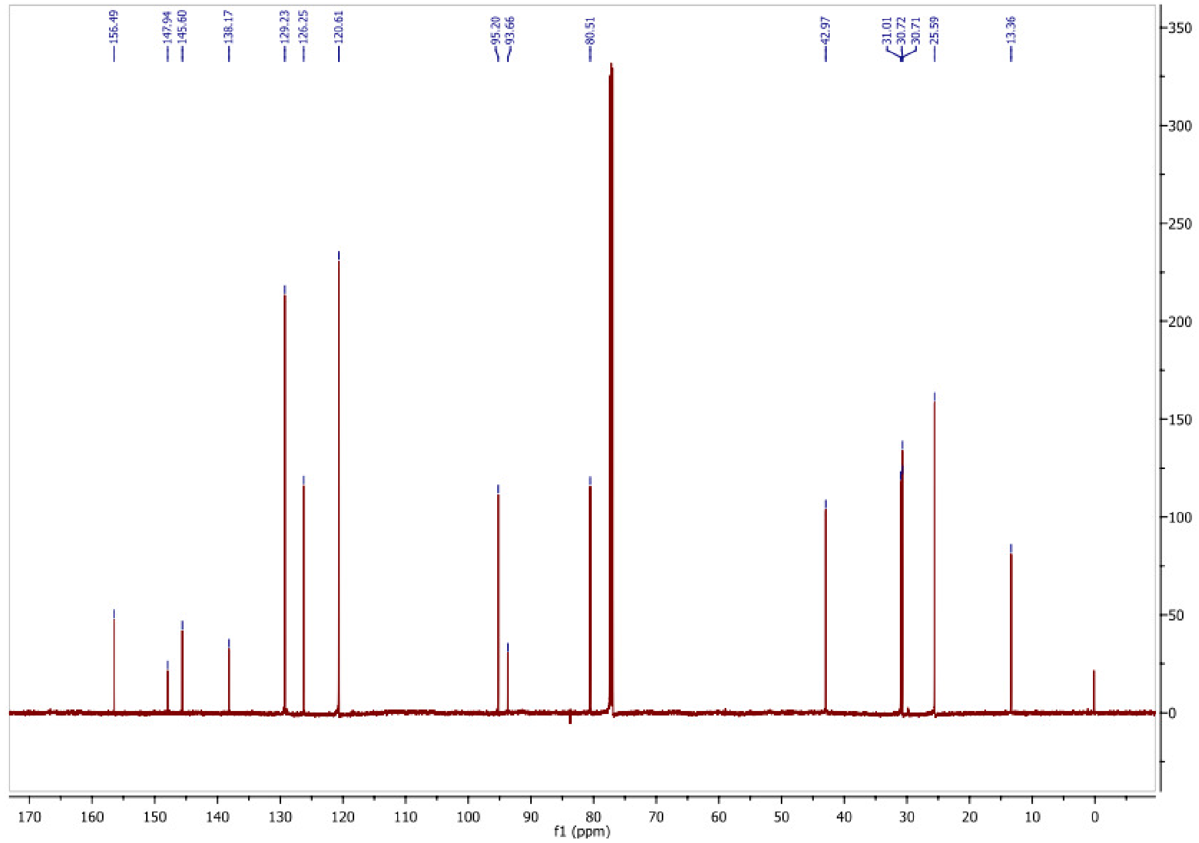

156.49
147.94
145.60
138.17
129.23
126.25
120.61
95.20
93.66
80.51
42.97
31.01
30.72
30.71
25.59
13.36
f1 (ppm)
